# Solar UV and X-ray spectral diagnostics

**DOI:** 10.1007/s41116-018-0015-3

**Published:** 2018-08-31

**Authors:** Giulio Del Zanna, Helen E. Mason

**Affiliations:** DAMTP, Centre for Mathematical Sciences, University of Cambridge, Wilberforce Road, Cambridge, CB3 0WA UK

**Keywords:** Atomic processes, Sun: corona, Atomic data, Line: formation, Techniques: spectroscopic, Sun: abundances

## Abstract

X-ray and ultraviolet (UV) observations of the outer solar atmosphere have been used for many decades to measure the fundamental parameters of the solar plasma. This review focuses on the optically thin emission from the solar atmosphere, mostly found at UV and X-ray (XUV) wavelengths, and discusses some of the diagnostic methods that have been used to measure electron densities, electron temperatures, differential emission measure (DEM), and relative chemical abundances. We mainly focus on methods and results obtained from high-resolution spectroscopy, rather than broad-band imaging. However, we note that the best results are often obtained by combining imaging and spectroscopic observations. We also mainly focus the review on measurements of electron densities and temperatures obtained from single ion diagnostics, to avoid issues related to the ionisation state of the plasma. We start the review with a short historical introduction on the main XUV high-resolution spectrometers, then review the basics of optically thin emission and the main processes that affect the formation of a spectral line. We mainly discuss plasma in equilibrium, but briefly mention non-equilibrium ionisation and non-thermal electron distributions. We also summarise the status of atomic data, which are an essential part of the diagnostic process. We then review the methods used to measure electron densities, electron temperatures, the DEM, and relative chemical abundances, and the results obtained for the lower solar atmosphere (within a fraction of the solar radii), for coronal holes, the quiet Sun, active regions and flares.

## Introduction

The solar corona is the tenuous outer atmosphere of the Sun, revealed in its full glory during a total solar eclipse. The visible spectrum of the solar corona has two major components: the continuum (the K-corona) due to Thomson scattering of photospheric light by the free electrons in the corona; and weak absorption lines (corresponding to the Fraunhofer lines—the F-corona) superimposed on the continuum emission. The latter is due to scattering by interplanetary dust particles in the immediate vicinity of the Sun. From white light coronagraph observations, and using a model for the distribution of electrons in the corona (van de Hulst 1950), it is possible to estimate the electron number density, which has a value of the order of $10^8\,\hbox {cm}^{-3}$ in the inner corona.

In addition, strong *forbidden* emission lines of highly-ionised atoms formed around 1–2 MK (e.g., the green and red coronal lines Fe XIV 5303 Å and Fe X 6374 Å) are also observed during eclipses. The forbidden lines allow measurements of electron densities and also of chemical abundances.

The solar corona is a very hot plasma (1 MK or more) that is mostly optically thin. The emission is due to highly-ionised atoms, which emit principally in the X-rays (5–50 Å), soft X-rays (50–150 Å), extreme ultra-violet (EUV, 150–900 Å) or far ultra-violet (UV, 900–2000 Å) region of the spectrum. Since radiation at these wavelengths cannot penetrate to the Earth’s surface, most of the observations and spectral diagnostics have been obtained from XUV (5–2000 Å) observations from space. These observations and associated spectroscopic diagnostics are the main focus for this review.

When imaged in the EUV at 1 MK, the solar corona shows a wide range of different structures that are magnetically linked to the underlying and cooler regions of the solar atmosphere, the chromosphere and the photosphere. Between the chromosphere and the corona there is a thin but highly complex region, the ‘transition region’ (TR, see e.g., Gabriel 1976), where the temperature dramatically increases. In addition we know, from both a theoretical and observational perspective, that there is a multitude of cooler loops at transition region temperatures that are not connected with the corona, as discussed e.g., by Feldman (1983), Antiochos and Noci (1986), Landi et al. (2000), Hansteen et al. (2014) and Sasso et al. (2015). The transition region emission is highly dynamic and very complex to interpret, with the likelihood of non-equilibrium and high-density effects that are normally not considered when studying the solar corona.

**Fig. 1:**
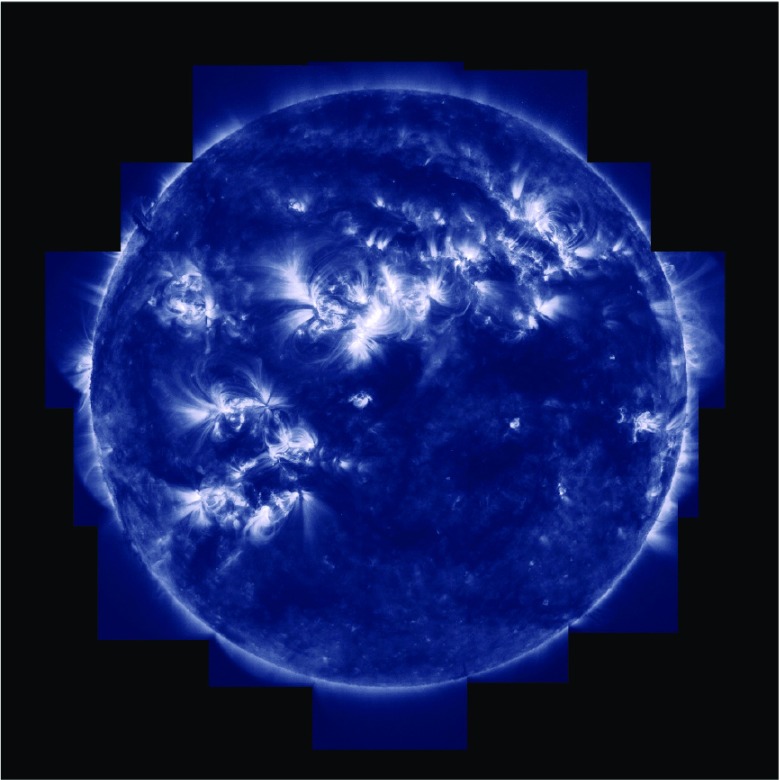
A TRACE composite image of the solar corona at 171 Å, formed at around 1 MK (courtesy of the TRACE consortium, NASA)

Much of the corona appears to have a diffuse nature (at modest spatial resolutions) and is referred to as ‘quiet Sun (QS)’. This quiet corona, which corresponds to a mixed-polarity magnetic field, is spattered with small bipolar regions which give rise to ‘bright points (BP)’ in the EUV and X-ray wavelength ranges. Then, in regions with enhanced magnetic field (which in the corresponding visible photosphere appear as sunspots), bright ‘active regions (AR)’ form, with a multitude of extended loop structures (see Fig. 1).

The other large-scale features of the solar outer atmosphere are the ‘coronal holes (CH)’, which appear as dark areas in EUV and soft X-ray images. At the photospheric level, they correspond to a prevalence of unipolar magnetic fields, corresponding to open magnetic field lines extending out into space. Inside polar coronal holes, large-scale ray-like extended features are usually observed, at various wavelengths (see e.g., DeForest et al. 2001). Due to their appearance these are named coronal hole plumes.

Remote-sensing XUV spectroscopy allows detailed measurements of plasma parameters such as electron temperatures and densities, the differential emission measure (DEM), the chemical abundances, Doppler and non-thermal motions, etc.

These topics have a vast literature associated with them. The present review aims to provide a synthetic up-to-date summary of some of the spectral diagnostics that have been used with data from recent missions or are currently routinely used, focusing on measurements of electron temperatures, electron number densities and chemical abundances in the lower solar corona. The diagnostic techniques used to study the plasma thermal emission measure (EM) distribution are only briefly described, as more emphasis is given to direct measurements of electron temperatures from ratios of lines from the same ion.

Although there are no current or planned instruments which will observe the X-rays with high-resolution spectrometers, we briefly discuss the rich set of diagnostics that in the past were available using satellite lines, i.e., lines formed by inner-shell excitation or dielectronic recombination.

We review the main processes underlying the formation of spectral line emission but we do not intend to replace in-depth presentations of basic material that can be found in books such as that one on solar UV and X-ray spectroscopy by Phillips et al. (2008) or specialised ones, such as the older but very good review on the transition region by Mariska (1992).

We provide a short review on atomic data, with emphasis on the most recent results. We also briefly describe some of the commonly-used atomic codes to calculate atomic data, and mention some of the issues related to uncertainties and line identifications, again not providing in-depth details on each of these topics, which were developed over more than 40 years. This review does not replace standard textbooks of atomic spectroscopy such as Condon and Shortley (1935), Grant (2006) and Landi Degl’Innocenti (2014), nor standard books and articles on atomic calculations in general.

The material presented here builds on and updates the useful review articles on atomic processes and spectroscopic diagnostics for the solar transition region and corona that have been previously written by Dere and Mason (1981), Gabriel and Mason (1982), Doschek (1985), Mason and Monsignori Fossi (1994), Del Zanna and Mason (2013) and Bradshaw and Raymond (2014).

We briefly discuss non-equilibrium effects such as time-dependent ionization and non-thermal distributions, two areas that have recently received more attention. Processes relating to hard X-ray emission from e.g., solar flares, as observed by e.g., RHESSI are not discussed in this review. Recent reviews on this topic have been provided by Benz (2017) and Krucker et al. (2008).

Plasma processes such as radiative transfer, relevant to the lower solar atmosphere (e.g., chromosphere), are not covered in this review. Such information can be found in standard textbooks such as Athay (1976) and Mihalas (1978). In the future, this Living review will be extended to cover the diagnostics of the outer solar corona, where densities become so low that photo-excitation and resonance excitation from the disk radiation need to be included in the modelling.

## The solar XUV spectrum

We first briefly review some of the main spectrometers which have been used to observe the Sun from the X-rays to the UV. In this review, we do not discuss hard X-ray spectrometers, nor spacecrafts which carried spectrometers, but ultimately did not produce spectra. The emphasis is on high-resolution spectra. For most diagnostic applications, having an accurate radiometric calibration is a fundamental requirement, but particularly difficult to achieve, especially in the EUV and UV. Significant degradation typically occurs in space, due to various effects (see, e.g., the recent review of BenMoussa et al. 2013). We also mention some EUV imaging instruments which have been used extensively.

### Historic perspective

The solar corona has been studied in detail since the early 1960s using data from a number of rocket flights. Some of them produced the best XUV spectra of the solar corona and transition region to date. An overview of these early days can be found in Doschek (1985), Mason and Monsignori Fossi (1994) and Wilhelm et al. (2004a).

#### Rocket flights

The X-rays are mostly dominated by L-shell ($n=2,3,4 \rightarrow 2$) emission from highly ionised atoms. Early (but excellent) X-ray spectra of the Sun were obtained by a large number of rocket flights, see for example Evans and Pounds (1968) and Davis et al. (1975), and the reviews of Neupert (1971b) and Walker Jr (1972).

The best X-ray spectrum of a quiescent active region was obtained with an instrument, built by the University of Leicester (UK), which consisted of Bragg crystal spectrometers with a collimator having a FOV (FWHM) of $3{^{\prime }}$, and flown on a British Skylark sounding rocket on 1971 November 30 (Parkinson 1975). The instrument had an excellent spectral resolution, was radiometrically calibrated and the whole spectral region was observed simultaneously, unlike many other X-ray instruments which scanned the spectral regions.

The soft X-ray (50–170 Å) spectrum of the quiet and active Sun is rich in $n=4 \rightarrow n=3$ transitions from highly ionised iron ions, from Fe vii to Fe xvi (see, e.g., Fawcett et al. 1968). Manson (1972) provided an excellent list of calibrated soft X-ray irradiances observed in quiet and active conditions in the 30–130 Å range by two rocket flights, on 1965 November 3 and 1967 August 8. The spectral resolution was moderate, about 0.23 Å (FWHM) for the quiet Sun, and 0.16 Å for the active Sun observation.

Behring et al. (1972) published a line list from a high resolution (0.06 Å) spectrum in the 60–385 Å region of a moderately active Sun. The instrument was built at the Goddard Space Flight Center (GSFC) and flown on an Aerobee 150 rocket flight on 1969 May 16. A similar line list for the EUV was produced by Behring et al. (1976). Behring et al. (1972, 1976) represent the best solar EUV line lists in terms of accuracy of wavelength measurements and spectral resolution. Unfortunately, these EUV spectra were not radiometrically calibrated.

Malinovsky and Heroux (1973) presented an integrated-Sun spectrum covering the 50–300 Å range with a medium resolution (0.25 Å), taken with a grazing-incidence spectrometer flown on a rocket on 1969 April 4. The photometric calibration of the EUV part of the spectrum was exceptionally good (about 10–20%), but the soft X-ray part was recently shown to be incorrect by a large factor (Del Zanna 2012a).

Acton et al. (1985) published a high-quality solar spectrum recorded on photographic film during the rocket flight on 1982 July 13, 2 min after the GOES X-ray peak emission of an M1-class flare. The instrument was an X-ray spectrometer/spectrograph telescope (XSST).

The spectrum was radiometrically calibrated, and it provided accurate line intensities from 10 Å up to about 77 Å. The spectral resolution was excellent, clearly resolving lines only 0.04 Å apart. Excellent agreement between predicted and measured line intensities has been found (see, e.g., the recent study of Del Zanna 2012a).

At longer wavelengths, the best EUV spectra have been obtained by the series of GSFC Solar Extreme Ultraviolet Rocket Telescope and Spectrograph (SERTS) flights. The one flown in 1989 (SERTS-89) (Thomas and Neupert 1994; hereafter TN94) observed the 235–450 Å range in first order. The SERTS-95 covered shorter wavelengths (Brosius et al. 1998). The SERTS-97 (Brosius et al. 2000) covered the 300–353 Å spectral region. Both SERTS-89 and SERTS-97 were radiometrically calibrated, although the calibration of the SERTS-89 spectra has been questioned (Young et al. 1998). Other SERTS and EUNIS (see, e.g., Wang et al. 2010, 2011) sounding rockets built at GSFC have served for the calibration of in-flight EUV spectrometers of several satellites, but have also returned many scientific results (Table 1).

**Table 1:** Some of the earlier space-borne spectroscopic instruments that observed the solar corona in the XUV

Instrument	Dates	$\lambda$ (Å)	$\delta \lambda \,(\AA )$	Min $\delta S$	$\delta t$	FOV	Calibrated
OSO-4	1968	300–1400	3.2	60	900 full scan	$1\times 1$	
Rocket	1969	50–300	0.25	–	–	Integrated Sun	
GSFC rocket	1969	60–385					
OSO-5	1969	25–400	$\sim$ 0.4		900 full scan	Integrated Sun	
OSO-5	1969	280–370	Integrated	None	2	integrated Sun	
		465–630					
		760–1030					
OSO-6	1969	280–1390	3.2	35	900 full scan	$1\times 1$	
OSO-7	1972	150–400	0.85	20	120	$5\times 5$	
OSO-8 UV spectrometer	1975–1978	1200–2000	0.02	$2.5{^{\prime \prime }}$	20–50 s	Variable	Yes
OSO-8 UV/vis. polychromator	1975–1978	Lyman $\alpha ,\beta$	2–10 pm	$\simeq$ 2	Variable	Variable	Yes
		Mg ii, Ca ii					
OSO-8 graphite crystals	1975–1978	1.5–6.7	0.03–0.06	–	10 s	Full-Sun	Yes (10%)
OSO-8 PET crystals	1975–1978	5.13–7.18		–	10 s	Full-Sun	Lab (30%)
Skylab SO55 (HCO)	1973–1974	296–1340	1.6	$5\times 5{^{\prime \prime }}$	$\sim$ 330	Variable	Yes (35%)
Skylab SO82A (NRL)	1973–1974	171–630	$\ge$ 0.03	$\ge 2 {^{\prime \prime }}$ slit-less		Full-Sun	
Skylab SO82B (NRL)	1973–1974	970–3940	0.04–0.08	$2\times 60{^{\prime \prime }}$			Yes
P78-1 SOLEX A	1979–	7.8–25	0.02 at 16 Å	$20{^{\prime \prime }}$	56 s	Variable	
P78-1 SOLEX B	1979–	3–10	0.001 at 8 Å	$60{^{\prime \prime }}$	56 s	Variable	
P78-1 SOLFLEX	1979–	1.82–8.53	0.00024–0.001	–	56 s	Full-Sun	
HRTS (8 rockets)	1975–1992	1170–1710	0.05	$1{^{\prime \prime }}$			Yes (some)
CHASE	1985	160–1344	0.25–0.4	$15{^{\prime \prime }}$	Few second	$3\times 1{^{\prime }}\,\hbox {max}$	No
SMM XRP FCS	1980–1989	1.8–25		$15{^{\prime \prime }}\times 14{^{\prime \prime }}$	$\simeq$ 10 m	Variable	Yes
SMM XRP BCS	1980–1989	1.7–3.2	$\simeq$ 0.0005	$6'\times 6'$	$\le$ 1 s		Yes
SMM UVSP	1980–1981	1750–3600	0.04 (0.02 IIo)	3	Minutes	Variable	Yes
Hinotori SOX1,2	1981–1982	1.7–1.95	0.00015	–	$\simeq$ 1	Full-Sun	Yes
XSST	1982 Jul 12	10–77	0.04	–	145 s	625 arcsec$^2$	Yes
Yohkoh BCS	1991–2001	1.8–5.0	0.0004–0.002	–	$\simeq$ 1	Full-Sun	Yes
SERTS	1989–	235–450	0.06	6	None	$5\times 8$	

### OSO

After the early rocket flights, the first series of small satellites were the Orbiting Solar Observatories (OSO). The first observations of $n=3 \rightarrow 2$ lines in solar flares were made with the OSO-3 satellite in the 1.3–20 Å region (Neupert et al. 1967). OSO-5 produced spectra of solar flares in the 6–25 Å region (Neupert et al. 1973), and the first solar-flare spectra containing the $n=2 \rightarrow 2$ L-shell iron emission, in the 66–171 Å range (Kastner et al. 1974a). OSO-6 also provided solar flare spectra (see the Doschek 1972; Doschek et al. 1973 line lists). OSO-7 produced EUV spectra in the 190–300 Å range of the coronal lines (Kastner et al. 1974b), later studied in detail by Kastner and Mason (1978).

OSO-8 (1975–1978) obtained the first high spatial ($2{^{\prime \prime }}$) and spectral observations of the chromosphere and the transition region with an UV spectrometer which operated in the 1200–2000 Å range (Bruner 1977). In its scanning mode, a line profile would typically be scanned across 1 Å with very high spectral resolution (0.02 Å) but low cadence (30–50 s). OSO-8 also carried a UV/visible polychromator which observed the H i Lyman $\alpha ,\beta$, the Mg ii H, K, and Ca ii H, K lines. Results from these instruments are reviewed by Bonnet (1981). Unfortunately, the instruments suffered a drop in sensitivity very early on during the mission. The degradation was so dramatic that measures were put in place by Bonnet and others for a strict cleanliness program for the SOHO spacecraft. This cleanliness program was an overall success, as most instruments suffered little degradation, compared to other missions (see below).

OSO-8 also carried two X-ray spectrometers, with co-aligned graphite and PET crystals (see Parkinson et al. 1978, and references therein). The graphite had a large geometrical area (100 cm$^2$) but lower spectral resolution than the PET system. The spectrometers were uncollimated so viewed the whole Sun. The overlapping of spectra from active regions located in different parts of the solar surface was therefore a problem. The great advantages of the OSO-8 spectrometers over previous ones were the high sensitivity and the fact that the crystals were fixed, i.e., the entire wavelength ranges were observed with a high cadence, about 10 s. The graphite crystal spectrometer was well calibrated (10%) in the laboratory and in flight (Kestenbaum et al. 1976).

### Skylab

More detailed studies of the solar corona from space started in May 1973, when Skylab, the first NASA space station, was launched. The Apollo Telescope Mount (ATM) on Skylab carried several solar instruments, observing from the UV to the X-rays between June 1973 and February 1974. Three successful series of Skylab workshops held in Boulder, Colorado, summarised the main results: coronal hole and high speed wind streams (Zirker 1977), solar flares (Sturrock 1980) and solar active regions (Orrall 1981).

The Harvard College Observatory (HCO) EUV spectrometer SO55 (Reeves et al. 1977a) on the ATM had a good spatial resolution in the 296–1340 Å range, but low spectral resolution ($\simeq$ 1.6 Å or more, depending on the spectral range). In the standard grating position, the instrument scanned with a $5{^{\prime \prime }}\times 5{^{\prime \prime }}$ slit covering six wavelengths typical of chromospheric to coronal temperatures: Ly$\alpha$ (1216 Å, $T \simeq 2 \times 10^4$ K); C II (1336 Å, $T \simeq 3.5 \times 10^4$ K); C III (977 Å, $T \simeq 7 \times 10^4$ K); O IV (554 Å, $T \simeq 1.5 \times 10^5$ K); O VI (1032 Å, $T \simeq 3 \times 10^5$ K) and Mg X (625 Å, $T \simeq 1.1 \times 10^6$ K). With other grating positions, spectroheliograms of the lines Ne VII (465 Å, $T \simeq 5 \times 10^5$ K) and Si XII (521 Å, $T \simeq 1.8 \times 10^6$ K) were also recorded. The good spatial resolution of the HCO spectrometer enabled (Vernazza and Reeves 1978) to produce a list of line intensities for different solar regions, which was a standard reference for many years. The radiometric calibration of the instrument (about 35% uncertainty) is described by Reeves et al. (1977b). This instrument suffered severe in-flight degradation.

**Fig. 2:**
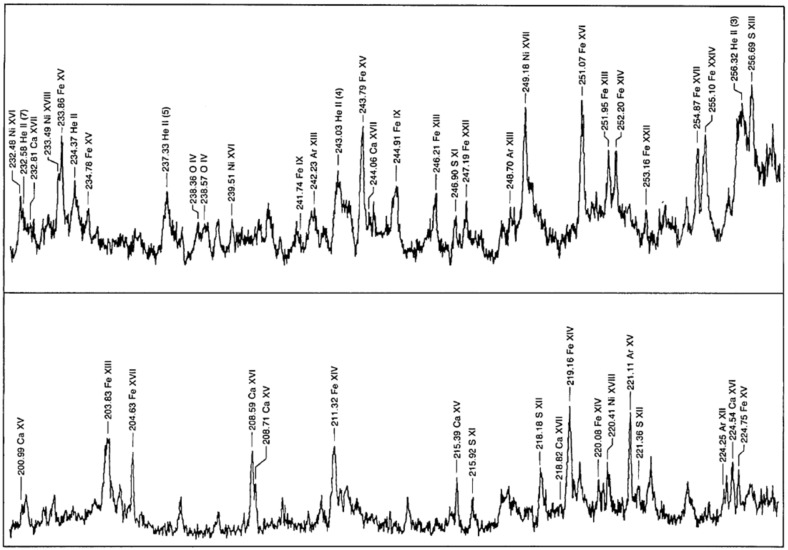
A portion of the NRL S082A slitless spectrum of a solar flare Image reproduced with permission from Feldman et al. (1988), copyright by OSA

The Naval Research Laboratory (NRL) S082A slitless spectroheliograph on the ATM (Tousey et al. 1977) had a very good wavelength coverage (170–630 Å), with a spatial resolution reaching $2''$ for small well defined features. The instrument obtained 1023 spectroheliograms of the whole Sun. However, the dispersion direction coincided with one spatial dimension (normally oriented E–W), so the images of the solar disk in the nearby spectral lines were overlapped (the instrument was fondly called the ‘overlappograph’). For this reason, most of scientific results have been obtained from spectra emitted by small well defined regions, such as compact flares, active region loop legs, the limb brightening, etc. For the smallest features, the instrument achieved an excellent spectral resolution of about 0.03 Å. As an example of the excellent quality of the instrument, a portion of a flare spectrum is shown in Fig. 2. A complete list of lines observed during flares was produced by Dere (1978b).

**Fig. 3:**
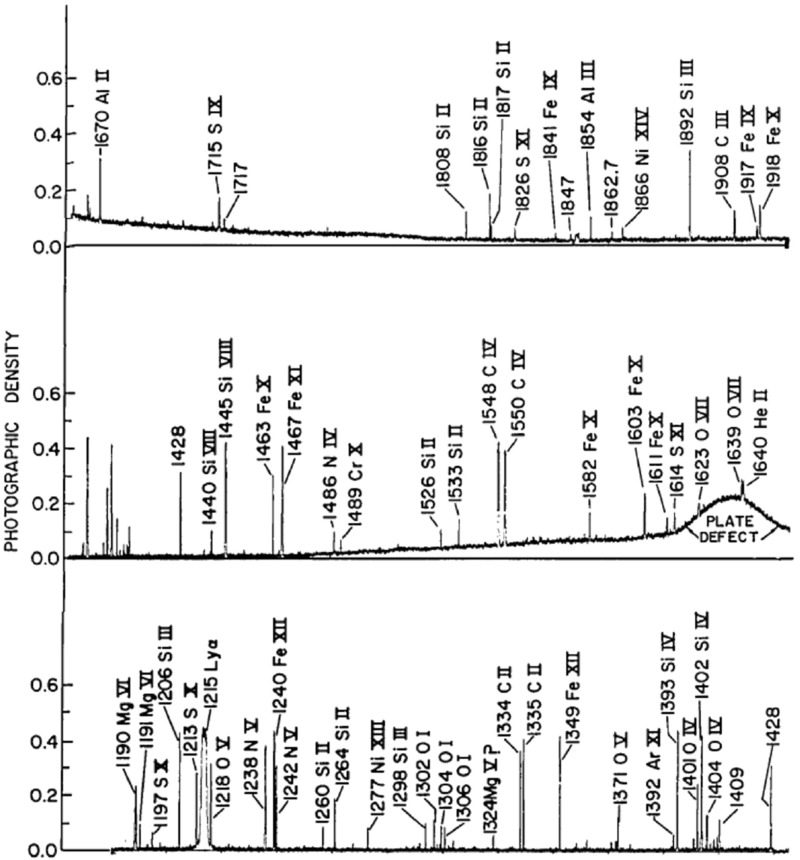
A portion of the NRL S082B spectrum taken about $30{^{\prime \prime }}$ off the solar limb, showing several coronal forbidden lines Image reproduced with permission from Feldman et al. (1988), copyright by OSA

The NRL Skylab SO82B (Bartoe et al. 1977) had a $2{^{\prime \prime }}\times 60{^{\prime \prime }}$ non-stigmatic slit and an excellent spectral resolution (0.04–0.08 Å) over the 970–3940 Å spectral range. Sandlin et al. (1977) and Sandlin and Tousey (1979) produced lists of coronal forbidden lines. Figure 3 shows a spectrum taken about $30{^{\prime \prime }}$ off the solar limb, showing several coronal forbidden lines (Feldman et al. 1988). Line lists of chromospheric lines were provided by Doschek et al. (1977a) and Cohen (1981). Many excellent papers using the S082A and S082B instruments were produced by the groups at NRL and collaborators.

### P78-1

Significant improvements in terms of spectral resolution were achieved in the X-rays with the SOLEX and SOLFLEX crystal spectrometers aboard the US P78-1 satellite, launched in 1979 (for a description of the solar instruments on the P78-1 spacecraft see Doschek 1983).

The SOLEX crystals scanned the 3–25 Å spectral region with a resolution of $10^{-3}$ Å at 8.2 Å and two multi-grid collimators of 20 or $60{^{\prime \prime }}$. For more details, see Landecker et al. (1979). The SOLFLEX crystals observed the full Sun and covered four spectral bands in the 1.82–8.53 Å range (1.82–1.97; 2.98–3.07; 3.14–3.24; 8.26–8.53 Å) with a resolution varying from $2.4 \times 10^{-4}$ Å at 1.9 Å to $10^{-3}$ Å at 8.2 Å. The bands were chosen to observe the strong resonance lines from Fe xxv, Ca xx, and Ca xix with their associated satellite lines, as well as several other lines from high-temperature ions. The spectral ranges were scanned by rotating the crystals, with a typical cadence of 56 s.

These spectrometers allowed seminal discoveries related to solar flares. During the rise (impulsive) phase of solar flares, strong blue asymmetries in the resonance lines were observed, interpreted as upflows during the chromospheric evaporation. The crystals also enabled the observation of non-thermal broadening and the measurement of temperature and emission measure variations during flares. For a review of the SOLEX results see, e.g., McKenzie et al. (1980a, b, 1985) and Doschek et al. (1981b). For a review of SOLFLEX spectra and their interpretation see, e.g., Doschek et al. (1979, 1981a).

### HRTS

Superb UV solar spectra were obtained by the series of High Resolution Telescope and Spectrometer (HRTS) instruments, developed at the Naval Research Laboratory (NRL) (Brueckner and Bartoe 1983). HRTS was flown eight times on sounding rockets between 1975 and 1992 which enabled many results. It was also flown on Spacelab 2, together with the CHASE instrument (see below) in 1985. HRTS covered the 1170–1710 Å spectral region with very high spectral (0.05 Å) and spatial ($1{^{\prime \prime }}$) resolution. HRTS had a stigmatic slit and also provided slit-jaw images. Each flight was unique, in that different slits, wavelength ranges or pointing were chosen. A good review of the flights can be found in http://wwwsolar.nrl.navy.mil/hrts_hist.html, written by K. Dere.

Sandlin et al. (1986) published a well-known list of HRTS observations of different regions on the Sun, with accurate wavelengths and line intensities, in the 1175–1710 Å spectral range. This list represents the most complete coverage in this wavelength range. A modification of the HRTS was flown on Spacelab 2 (Brueckner et al. 1986).

**Fig. 4:**
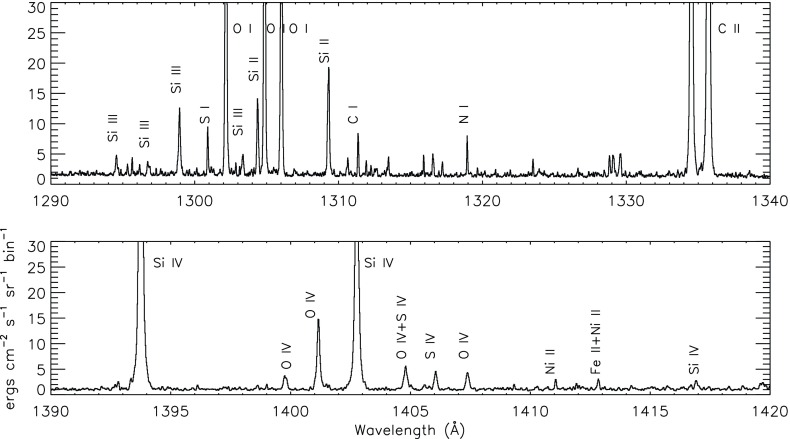
An averaged spectrum of an active region at the limb from HRTS, in two spectral regions, dominated by the important UV lines from Si iii, C ii, Si iv, O iv and S iv Data from Brekke et al. (1991)

Brekke et al. (1991) published excellent HRTS spectra, obtained during the second rocket flight in February 1978. The spectrum was radiometrically calibrated by matching the quiet Sun intensities with those measured by the Skylab S082B calibration rocket flight CALROC. The long stigmatic slit of the HRTS instrument covered many solar regions. Figure 4 shows an averaged spectrum of an active region at the limb from HRTS.

### CHASE

The Coronal Helium Abundance Spacelab Experiment (CHASE, see Breeveld et al. 1988) on the Spacelab 2 Mission (1985) was specifically designed to determine the helium abundance from the ratio of He ii 304 Å to Lyman-$\alpha$ 1218 Å, on the disc and off the limb. A value of $0.079\pm 0.011$ for the quiet corona was obtained by Gabriel et al. (1995). However, the instrument also recorded several spectral lines, used by Lang et al. (1990) to describe the temperature structure of the corona. One limiting aspect of CHASE was the lack of a specific radiometric calibration for the flight instrument.

### SMM

The Solar Maximum Mission (SMM) was dedicated to the study of active regions and solar flares. SMM was launched in February 1980 but encountered some problems. It was repaired in-orbit by the NASA Space Shuttle in April 1984, and then resumed full operations until December 1989. It carried several instruments.

**Fig. 5:**
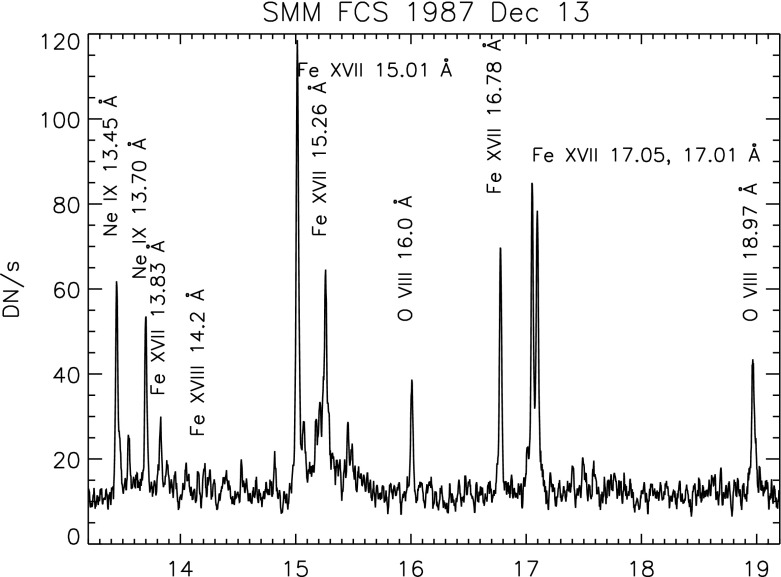
The SMM/FCS AR spectrum of 1987 December 13 Image reproduced with permission from Del Zanna and Mason (2014), copyright by ESO

The SMM X-ray polychromator (XRP) Flat and Bent Crystal Spectrometers (FCS and BCS) instruments (Acton et al. 1980) produced excellent X-ray spectra of the solar corona, active regions and solar flares. The XRP/FCS had a collimator of about $15{^{\prime \prime }}\times 14{^{\prime \prime }}$, so had a limited spatial resolution, although it could raster an area as large as $7'\times 7'$ with $5{^{\prime \prime }}$ steps. The FCS crystals could be rotated to provide the seven detectors access to the spectral range 1.40–22.43 Å. The FCS 5–25 Å spectral region is dominated by Fe xvii, O viii, Ne ix lines. A sample spectrum of a quiescent active region is shown in Fig. 5. The sensitivity of the FCS instrument decreased significantly early on in the mission, in particular at the longer wavelengths, so the O vii lines around 22 Å became very weak. The in-flight radiometric calibration was based on the assumption of carbon deposition on the front filter (see, e.g., Saba et al. 1999). The FCS data allowed measurements of the temperature distribution and electron density of solar active regions and flares together with measurements of relative coronal abundances of various elements. A complete list of lines observed with the SMM FCS during flares was published by Phillips et al. (1982). Another excellent SMM/FCS solar flare spectrum, this time with $n=4 \rightarrow 2$ calibrated line intensities, was published by Fawcett et al. (1987). A significant limitation of these solar observations was that the spectral range of each detector was scanned, hence lines within the same channel were not observed simultaneously. This considerably complicated the analysis (cf. Landi and Phillips 2005), particularly for solar flares.

The XRP/BCS, with a collimator field of view of about $6'\times 6'$ (the size of a large active region), was able to obtain spectra with eight position-sensitive proportional counters in the range 1.7–3.2 Å simultaneously, at a resolving power of about $10^4$. The XRP/BCS observed X-ray line complexes of high temperature (in excess of 10 MK) coronal lines: Fe xxvi, Fe xxv, Ca xix, and S xv. This spectral range allowed a wide range of plasma diagnostics to be applied. The He-like ions allowed measurements of electron densities and temperatures (Gabriel and Jordan 1969). The satellite lines also allowed some important diagnostic measurements (see, e.g., Gabriel 1972a, b; Gabriel and Phillips 1979; Doschek 1985; Phillips et al. 2008).

The Ultraviolet Spectrometer and Polarimeter (UVSP) on board SMM was used to observe many features, including solar active regions and flares in the 1750–3600 Å range in first order of diffraction and 1150–1800 Å in the second (Woodgate et al. 1980). The spatial resolution was very good, about $3{^{\prime \prime }}$, and the spectral resolution was excellent (0.04 Å in first order and 0.02 in second order). Various slits were available, from 1 to $15{^{\prime \prime }}$ wide. As with previous UV instruments, the UVSP also suffered severe degradation in orbit (Miller et al. 1981) during its 10 months of operations.

### Hinotori

Hinotori was a Japanese spacecraft in orbit between 1981 and 1982 which was used to observe high-energy X-ray emission produced by solar flares. The most important results were obtained with the Bragg spectrometers. The SOX2 scanning spectrometer had an excellent spectral resolution (0.15 mÅ) and produced excellent spectra of Fe xxvi and Fe xxv during large flares. Very high temperatures were measured. For a summary of the results from these spectrometers see Tanaka et al. (1982) and Tanaka (1986).

### Yohkoh

Yohkoh (Japanese for *sunbeam*) was used to observe the Sun in X-ray emission from 1991 to 2001. The Bragg Crystal Spectrometer (BCS, see Culhane et al. 1991) produced similar measurements to the SMM/BCS of the X-ray H- and He-like line complexes. It used 4 bent crystals which observed the X-ray lines of highly ionized S, Ca, and Fe produced by flares and active regions in the 1.76–5.1 Å wavelength range (1.76–1.80: Fe xxvi; 1.83–1.89: Fe xxv; 3.16–3.19: Ca xix; 5.02–5.11: S xv). Many scientific results have been obtained, in particular those regarding the temperatures during flares (Phillips and Feldman 1995; Feldman et al. 1996).

One potential problem with instruments such as XRP/BCS and the Yohkoh BCS is that sources in different spatial locations produce superimposed spectra at different wavelengths. For example, extended sources produced a broadening of the lines, and complex spectra could arise in the case of multiple flares occurring at the same time within the field of view. This was not normally an issue for XRP/BCS, given its field of view ($6\times 6$ arc minutes), but was occasionally more of a problem for Yohkoh/BCS which observed the full Sun.

### SoHO

The Solar and Heliospheric Observatory (SoHO), a joint NASA and ESA mission which was launched in December 1995 to the L1 position, is still operational, although the attitude loss in 1998 caused significant degradation to some of its instruments, many of which have now been switched off. First results from SoHO were published in a special issue of *Solar Physics* in 1997 (volume 170). With 24 h monitoring, SoHO has produced a wealth of data and has changed our view of the Sun. SoHO carried a suite of several instruments, performing in-situ and remote-sensing observations. The radiometric calibration of various instruments on-board SoHO during the first few years of the mission was discussed during two workshops held at the International Space Science Institute (ISSI), in Bern. The results were summarised in the 2002 ISSI Scientific Report SR-002 (Pauluhn et al. 2002).

Here, we summarise the spectroscopic instruments used to study the solar corona. The Coronal Diagnostic Spectrometer (CDS), a UK-led instrument (Harrison et al. 1995) was routinely operated from 1996 to 2014. It comprised of a Wolter–Schwarzschild type II grazing incidence telescope, a scan mirror, a set of different slits (2, 4, $90{^{\prime \prime }}$), and two spectrometers, a normal incidence spectrometer (NIS) and a grazing incidence spectrometer (GIS). The wavelength range covered by the two detectors (150–800 Å) contains many emission lines emitted from the chromosphere, the transition region and the corona. The NIS had two wavelength bands, NIS-1, 308–381 Å and NIS-2, 513–633 Å. To construct monochromatic images (rasters), a scan mirror was moved across a solar region to project onto the detectors the image of a stigmatic slit ($2''$ or $4''$). For the NIS instrument, the spectral resolution was about 0.3 Å before the SoHO loss of contact in 1998, then degraded to about 0.5 Å afterwards. The effective spatial resolution was about $4{^{\prime \prime }}$.

The in-flight radiometric calibration of the CDS instrument was found to be very different from that which had been measured on the ground (actually better than expected, for the NIS see, e.g., Landi et al. 1997; Del Zanna et al. 2001a; Lang et al. 2007, while for the GIS see Del Zanna et al. 2001a).

The CDS team believed that the variation in the long-term radiometric calibration of the NIS instrument was mainly caused by the degradation of the microchannel plate detectors following the use of the wide slit. A standard correction was implemented in the calibration software. However, Del Zanna et al. (2010a) showed that this standard correction was quite different from expectation, and overestimated by large factors (2–3) for the stronger lines. The NIS instrument only degraded by about a factor of two in 13 years, which is quite remarkable. The new calibration by Del Zanna et al. (2010a) was confirmed by sounding rocket flights (e.g., EUNIS-2007, see Wang et al. 2011) and was adopted for the final calibration of the instrument.

**Fig. 6:**
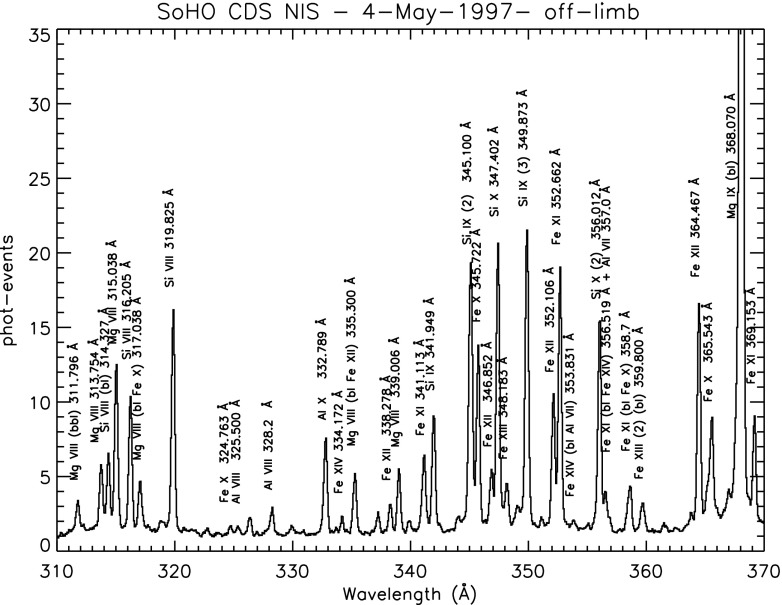
CDS NIS 1 averaged spectrum of the quiet Sun (off-limb) Image adapted from Del Zanna (1999)

**Fig. 7:**
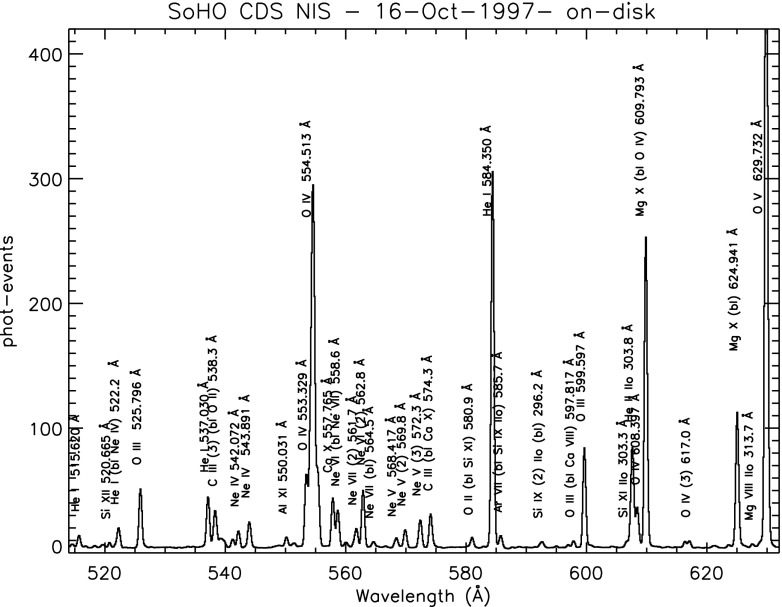
SoHO CDS NIS 2 averaged spectrum of the quiet Sun (on-disk) Image adapted from Del Zanna (1999)

The diagnostic potential of CDS was discussed by Mason et al. (1997). A NIS spectral line list for the quiet Sun can be found in Brooks et al. (1999), while more extended lists for different regions, with line identifications based on CHIANTI are given in Del Zanna (1999). Sample NIS spectra are given in Figs. 6 and 7.

The grazing incidence spectrometer used a grazing incidence spherical grating that disperses the incident light to four microchannel plate detectors placed along the Rowland Circle (GIS 1: 151–221 Å, GIS 2: 256–341 Å, GIS 3: 393–492 Å and GIS 4: 659–785 Å). The spectral resolution of the GIS detectors was about 0.5 Å. The GIS was astigmatic, focusing the image of the slit along the direction of dispersion but not perpendicular to it. The in-flight radiometric calibration of the GIS channels (only the pinhole $2{^{\prime \prime }}\times 2{^{\prime \prime }}$ and $4{^{\prime \prime }}\times 4{^{\prime \prime }}$ slits) is described in Del Zanna et al. (2001a) and Kuin and Del Zanna (2007). No significant degradation of the GIS sensitivity was found. The GIS suffered badly from ‘ghost lines’ due to the spiral nature of the detector. This made data analysis somewhat complicated. A full list of GIS spectral lines can be found in Del Zanna (1999).

**Fig. 8:**
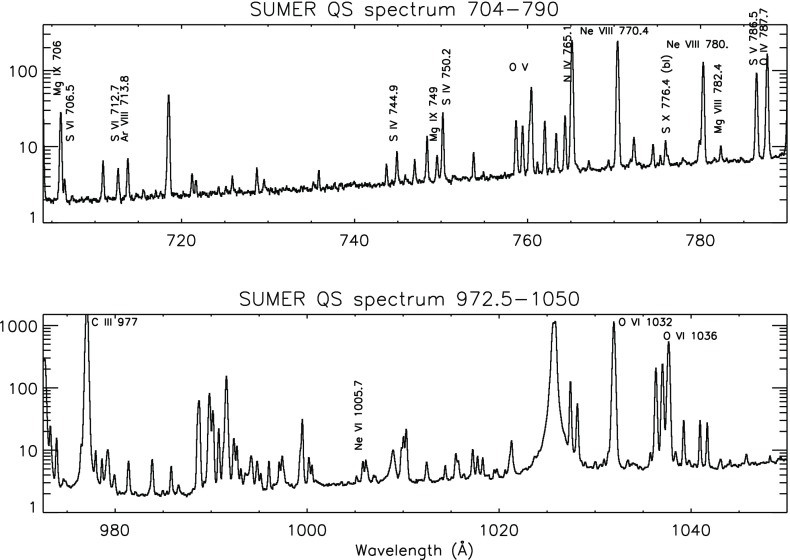
An on-disc quiet Sun SUMER spectrum in the two spectral ranges which will be covered by the Solar Orbiter SPICE spectrometer

The Solar Ultraviolet Measurement of Emitted Radiation (SUMER) was a joint German and French-led instrument (Wilhelm et al. 1995). It was a high-resolution ($1{^{\prime \prime }}$ spatial) spectrometer covering the wavelength range 450–1600 Å, dominated by lines emitted by the chromosphere and transition region. Detector A observed the 780–1610 Å range, while detector B covered the 660–1500 Å range. Second-order lines were superimposed on the first order spectra, however the second order sensitivity was such that only the strongest lines in the 450–600 Å range were observable.

SUMER had an excellent spectral resolution (${\lambda \over d \lambda } = 19{,}000$–40,000), and was able to measure Doppler motions (flows) with an accuracy better than 2 km/s, using photospheric lines as a reference. As in the case of the CDS, SUMER was able to scan solar regions to obtain monochromatic images in selected spectral lines. One main difference was that only lines within a wavelength range (typically 40 Å) could be recorded simultaneously by SUMER.

One disadvantage with SUMER was the amount of time it took to scan a spatial area. Some difficulties were encountered with the scanning mechanism, so it was used sparingly during the latter part of the mission. The SUMER radiometric calibration for the first few years of the mission was discussed at the ISSI workshops, see ISSI Scientific Report SR-002 (Pauluhn et al. 2002).

A spectral atlas of SUMER on-disk lines was published by Curdt et al. (2001). Figure 8 shows the spectrum in the two spectral ranges that will be observed with the Solar Orbiter SPICE instrument. Several strong transition region lines are present. A list of SUMER on-disk quiet Sun radiances in the 800–1250 Å range was published by Parenti et al. (2005), where radiances of a prominence were also provided. Within the SUMER spectral range, several coronal forbidden lines become visible off the solar limb, see e.g., the spectral atlases by Feldman et al. (1997) and Curdt et al. (2004). Some of the high-temperature forbidden lines become visible even on-disk in active regions and during flares (see, e.g., the lists of Feldman et al. 1998a, 2000). The last observations were carried out with SUMER in 2014, due to the significant degradation of the detectors.

The Ultraviolet Coronagraph Spectrometer (UVCS) was an instrument that was built and operated by a USA–Italy collaboration (Kohl et al. 1995). It observed the solar corona from its base out to 10 $R_\odot$. Its heritage was the Spartan Ultraviolet Coronagraph Spectrometer, which flew several times between 1993 and 1998 (Kohl et al. 2006). Most of the UVCS scientific results are based on the measurements of the strong H I Lyman-$\alpha$ and O vi (1032 and 1037 Å) lines, which are partly collisionally excited and partly resonantly scattered (Raymond et al. 1997). Several coronal lines such as Si xii (499 and 521 Å) and Fe xii (1242 Å) were also observed. UVCS produced measurements of chemical abundances, proton velocity distribution, proton outflow velocity, electron temperature, and ion outflow velocities and densities. Some of scientific results from UVCS have been reviewed by Kohl et al. (2006) and Antonucci et al. (2012). The instrument suffered a significant degradation (factor of ten) at first light, but continued to operate for a long time.

### CORONAS

CORONAS-F, launched in 2001, provided XUV spectroscopy with the SPIRIT (Russian-led, see Zhitnik et al. 2005) and RESIK (REntgenovsky Spektrometr s Izognutymi Kristalami, Polish-led, see Sylwester et al. 2005) instruments, especially of flares during solar maximum. The SPIRIT spectroheliograph had two wavelength ranges, 176–207 and 280–330 Å, and a relatively high spectral resolution of about 0.1 Å. The instrument was slitless. The solar light was deflected at a grazing angle of about $1.5^{\circ }$ by a grating, and then focused by a mirror coated with a multilayer with a high reflectivity in the EUV. This resulted in ‘overlappogram’ images of the spectral lines, highly compressed in the solar E–W direction, but with a good spatial resolution along the N–S direction. Most solar flares have a typical small spatial extension during the impulsive phase, so it was relatively straightforward to obtain flare spectra, only slightly contaminated by nearby emission at similar latitudes. The radiometric calibration was only approximate, obtained with the use of line ratios. SPIRIT observed several flares. For details and a line list see Shestov et al. (2014).

RESIK was a full-Sun spectrometer employing bent crystals to observe simultaneously four spectral bands within the range 3.4–6.1 Å, with a spectral resolution of about 0.05 Å. The instrumental fluorescence was a major limiting effect in the spectra, creating a complex background emission, which needed to be subtracted for continuum analysis, and to measure the signal from weaker lines.

**Fig. 9:**
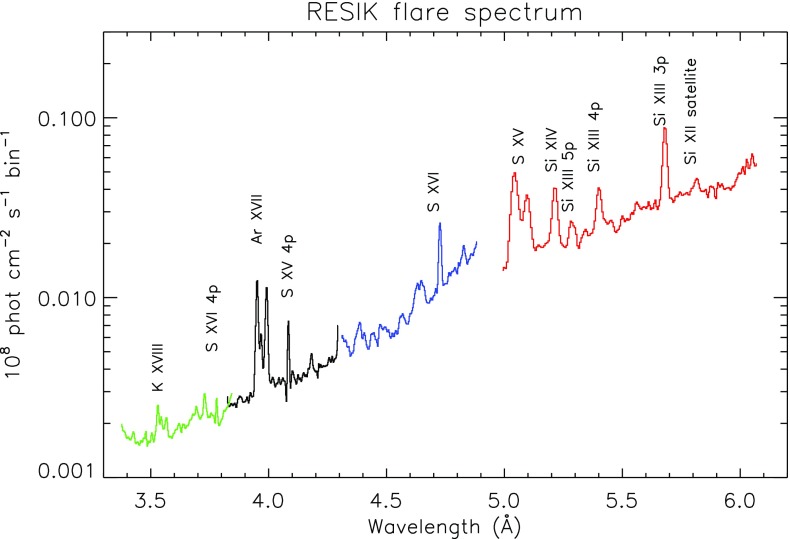
RESIK spectrum of a flare. Different colours indicate the four channels Image adapted from Chifor et al. (2007)

The RESIK wavelength range (see Fig. 9) is of particular interest because line-to-continuum measurements can be used for absolute abundance determinations (see, e.g., Chifor et al. 2007) and because of the presence of dielectronic satellite lines (see, e.g., Dzifčáková et al. 2008). RESIK observed a large number of flares during 2001–2003.

Some XUV spectroscopy was provided by CORONAS-Photon with the SPHINX (Polish-led) instrument (Sylwester et al. 2008), although the satellite, launched in 2009, unfortunately had a failure in 2010.

### RHESSI

The NASA *Reuven Ramaty High-Energy Solar Spectroscopic Imager* (RHESSI, Lin et al. 2002), despite not being a purely high-resolution spectrometer, provided nevertheless very important X-ray spectral observations since 2002 at high energies, above 3 keV. The instrument achieved spatial and spectral resolutions significantly higher than those of earlier missions. Depending on the signal, it was possible to obtain imaging in selected energy bands at about $2.5''$ resolution. The spectra have about 1 keV resolution, just allowing the Fe line complex to be resolved at 6.7 keV from the continuum emission.

### Hinode

Hinode (Japanese for *sunrise*) is a Japanese mission developed and launched in September 2006 by ISAS/JAXA, collaborating with NAOJ as a domestic partner, NASA and STFC (UK) as international partners. Scientific operation of the Hinode mission is conducted by the Hinode science team organized at ISAS/JAXA. This team mainly consists of scientists from institutes in the partner countries. Support for the post-launch operation is provided by JAXA and NAOJ (Japan), STFC (UK), NASA, ESA, and NSC (Norway).

Initial results from the Hinode satellite have been published in special issues of the PASJ, Science and A&A journals in 2007 and 2008. Hinode (Kosugi et al. 2007) carried 3 instruments, the Solar Optical telescope (SOT, see Tsuneta et al. 2008), the X-ray imaging telescope (XRT, see Golub et al. 2007), and the Extreme-ultraviolet Imaging Spectrometer (EIS, see Culhane et al. 2007). We focus here on the latter instrument.

**Fig. 10:**
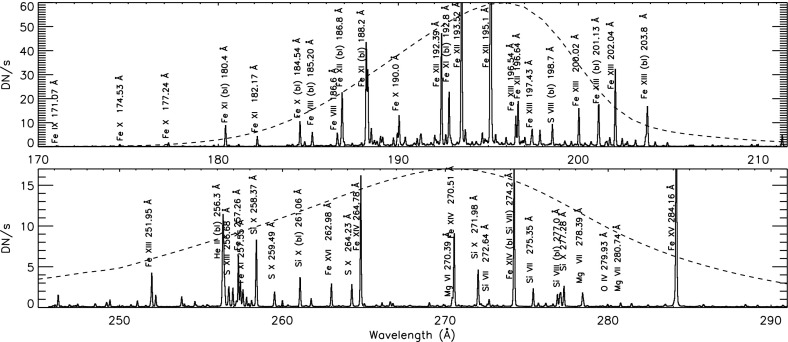
Hinode EIS spectrum of an active region. The dashed lines are the (scaled) effective areas of the two channels Image adapted from Young et al. (2007a)

EIS has two wavelength bands, 170–211 and 246–292 Å (see Fig. 10), which include spectral lines formed over a wide range of temperatures, from chromospheric to flare temperatures ($\log T ({\mathrm{MK}}) = 4.7{-}7.3$). The instrument has an effective spatial resolution of about 3–$4''$. The high spectral resolution (0.06 Å) allows velocity measurements of a few km/s. However with no chromospheric lines, these velocity measurements are difficult to calibrate. Rastering is normally obtained with the narrow slits ($1''$ or $2''$).

The ground radiometric calibration (Lang et al. 2006) was revised by Del Zanna (2013b). A significant degradation e of about a factor of two within the first 2 years was found in the longer-wavelength channel. This degradation was confirmed by Warren et al. (2014).

Earlier spectroscopic diagnostic applications were described in Del Zanna and Mason (2005b) and Young et al. (2007a, b). A tabulation of spectral lines observed by Hinode/EIS was provided by Brown et al. (2008). A more complete list of coronal lines with their identification based on CHIANTI was provided by Del Zanna (2012b). A comprehensive list with identifications of cool ($T \le 1\,\hbox {MK}$) emission lines can be found in Del Zanna (2009a) and Landi and Young (2009). A discussion of the high-temperature flare lines and their blends can be found in Del Zanna (2008b) and Del Zanna et al. (2011b).

### SDO

The NASA Solar Dynamics Observatory (SDO, see Pesnell et al. 2012) was launched in February 2010, carrying a suite of instruments. The Helioseismic and Magnetic Imager (HMI, see Schou et al. 2012), led from Stanford University in Stanford, California, measures the photospheric magnetic field. The Atmospheric Imaging Assembly (AIA, see Lemen et al. 2012), led from the Lockheed Martin Solar and Astrophysics Laboratory (LMSAL), provides continuous full-disk observations of the solar chromosphere and corona in seven extreme ultraviolet channels.

The only coronal spectrometer on SDO is the Extreme ultraviolet Variability Experiment (EVE) instrument (Woods et al. 2012). It provided solar EUV irradiance with an unprecedented wavelength range (1–1220 Å) and temporal resolution (10 s). The EVE spectra are from the Multiple EUV Grating Spectrographs (MEGS) and have about 1 Å spectral resolution. The MEGS A channel is a grazing incidence spectrograph for the 50–380 Å range. It ceased operations in May 2014. The MEGS B channel is a double pass normal incidence spectrograph for the 350–1050 Å range. A list of MEGS flare lines and their identifications is presented in Del Zanna and Woods (2013). Additionally, EVE has an EUV Spectrophotometer (ESP), a transmission grating and photodiode instrument similar to the SoHO Solar EUV Monitor (SEM). ESP has four first-order channels centered on 182, 257, 304, and 366 Å with approximately 40 Å spectral width, and a zero order channel covering the region 1–70 Å.

MEGS B suffered a significant degradation of its sensitivity from the beginning of the mission (factor of 10), while the degradation of MEGS A was more contained (see, e.g., BenMoussa et al. 2013). Various procedures such as the line ratio technique, previously applied to other instruments (as SoHO CDS, Del Zanna et al. 2001a), were used to obtain in-flight corrections. A recent evaluation of the EVE version 5 calibration showed relatively good agreement (to within 20%) with the SoHO CDS irradiance measurements for most lines (Del Zanna and Andretta 2015).

**Fig. 11:**
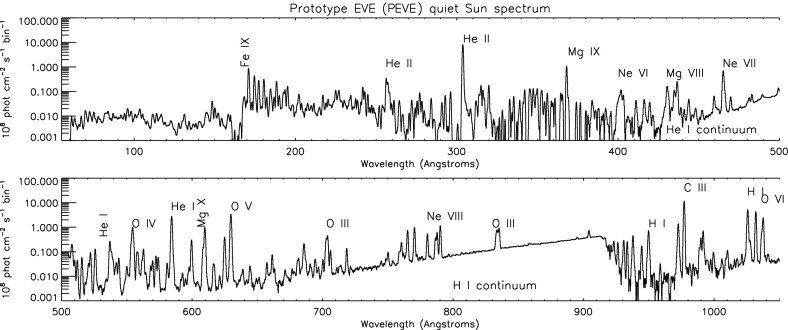
The EUV spectrum of the whole Sun, as measured by the prototype SDO/EVE instrument flown aboard a rocket in 2008 April 14

The combined spectral range of the two channels was observed with a sounding rocket in 2008 April 14 carrying a prototype EVE MEGS instrument (Woods et al. 2009; Chamberlin et al. 2009; Hock et al. 2010, 2012). The spectrum was radiometrically calibrated on the ground, and is shown in Fig. 11. On that day, and during the whole long deep solar minimum around 2008, the Sun was extremely quiet, so in principle the prototype EVE observation should represent the best EUV solar spectrum at minimum. However, significant discrepancies were found for several of the strongest lines when comparisons of the SoHO CDS and EVE observations were made (from May 2010, when the Sun started to be more active) as discussed in Del Zanna and Andretta (2015). The irradiances of the strongest lines appear to have been overestimated by 30–50%.

**Fig. 12:**
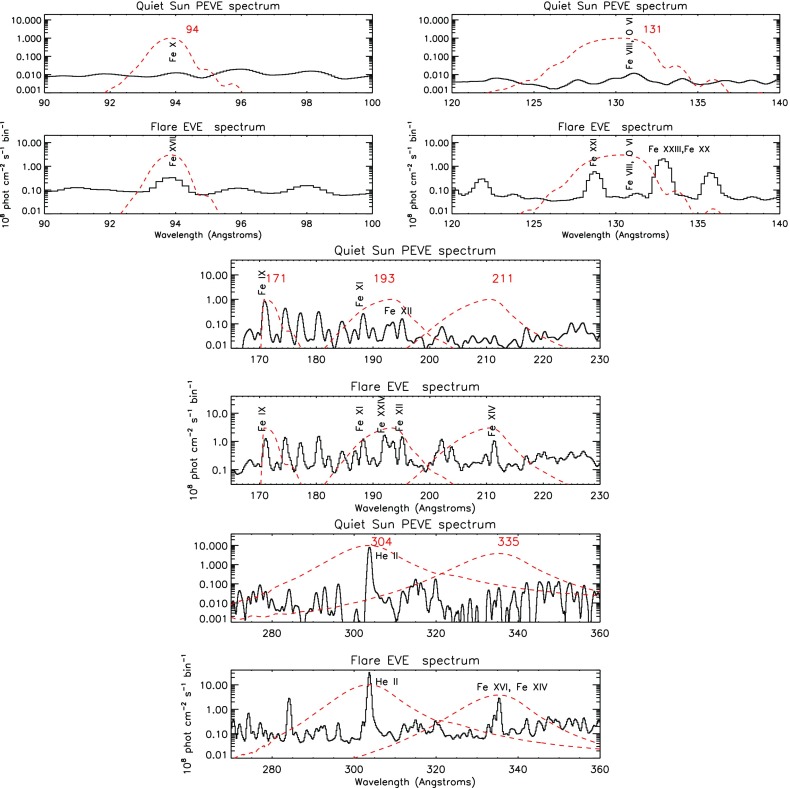
The EVE spectrum of the quiet Sun (above) and of an X-class flare (below, see Del Zanna and Woods 2013), in the spectral ranges covered by the six SDO/AIA EUV bands (their effective areas are shown, rescaled, with dashed lines)

It is worth noting that the EVE spectra vary a lot in some spectral ranges, depending on the level of activity on the Sun. An example is shown in Fig. 12, where the quiet Sun spectrum is shown together with an X-class flare spectrum in the spectral ranges covered by the six SDO AIA EUV bands. Even the coarse EVE spectral resolution clearly shows that each AIA band has contributions from several spectral lines, and that different spectral lines become dominant under different solar conditions.

### IRIS

The Interface Region Imaging Spectrograph (IRIS, De Pontieu et al. 2014) was launched in July 2013, and has been producing excellent spectra and images of the solar atmosphere at very high temporal (2 s) and spatial ($0.33{-}0.4{^{\prime \prime }}$) resolution, but with a limited field of view. The high resolution has enabled many new scientific results. A special section of *Science* in October 2014 (volume 346) was dedicated to the first results from IRIS.

As with earlier UV instruments, IRIS suffered significant in-flight degradation during the first few years of the mission.

**Fig. 13:**
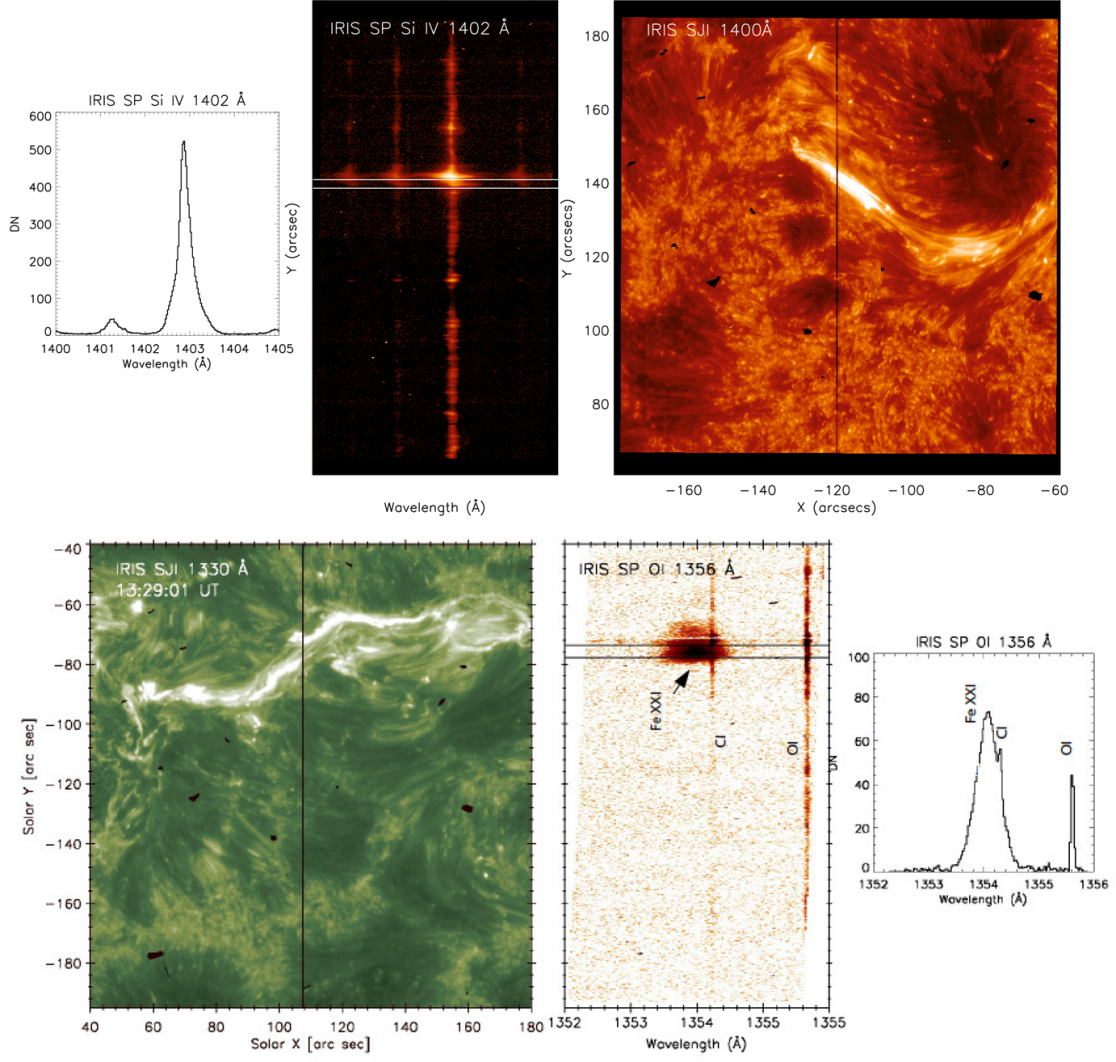
Top: IRIS spectra in the Si iv spectral band (courtesy of V. Polito). The right panel shows the Si iv slit-jaw image and the location of the slit. The middle panel shows the detector image in the Si iv spectral window, and the left panel shows the averaged spectrum from the region indicated by the two parallel lines in the middle panel. Bottom: IRIS spectra in the Fe xxi band during a flare. The left image shows the C ii slit-jaw image and the location of the IRIS slit across two ribbons during a flare. The middle image shows a detector image with the Fe xxi 1354.08 Å line and photospheric lines from C i and O i. The rightmost panel shows the spectrum averaged along the slit in between the two lines shown in the middle panel

The IRIS Slit Jaw Imager (SJI) provides high-resolution images in four different passbands (C ii 1330 Å, Si iv 1440 Å, Mg ii k 2796 and Mg ii wing at 2830 Å). The IRIS spectrograph (SP) observes spectra in the 1332–1358, 1389–1407, and 2783–2834 Å spectral regions, where there are several emission lines formed in the photosphere, chromosphere, as well as in the transition region (Si iv, O iv, S iv). The highest temperature line observed by IRIS is the Fe xxi 1354.08 Å line, formed at high temperatures (12 MK) typical of flares (Young et al. 2015; Polito et al. 2015, see Fig. 13). This interesting flare line was previously observed with Skylab SO82B (see, e.g., Doschek et al. 1975) and SMM UVSP (see, e.g., Mason et al. 1986).

The use of a slit together with a slit-jaw image enables the precise location of the spectra to be established, and in addition high-cadence observations can be obtained. HRTS also had a slit-jaw camera but lacked the high-cadence which IRIS has, used photographic plates, and was generally flown on a rocket (with relatively short duration). Nonetheless, HRTS provided some interesting observations which can now be explored in more detail with IRIS.

## The formation of the XUV spectrum

### Spectral line intensity in the optically thin case

In the majority of cases, the XUV emission from the solar corona and transition region is optically thin, i.e., all the radiation that we observe remotely has freely escaped the solar atmosphere. In this case, the observed intensity of a spectral line is directly related to its bound–bound emissivity. The radiance (or more simply intensity) $I(\lambda _{ji})$ of a spectral line of wavelength $\lambda _{ji}$ (frequency $\nu _{ji} = c/\lambda _{ji}$) is therefore:

1 $$\begin{aligned} {I(\lambda _{ji})} = {{{h \nu _{ji}\over {4\pi }}\;{\int \limits \,N_j(Z^{+r})\,A_{ji}\;ds}}} \quad (\mathrm{erg}\,\mathrm{cm}^{-2}\,\mathrm{s}^{-1}\,\mathrm{sr}^{-1}) \end{aligned}$$

where $Z^{+r}$ indicates the element of atomic number *Z* which is *r* times ionized, *i*, *j* are the lower and upper levels of the ion $Z^{+r}, h$ is Planck’s constant, $A_{ji}$^1^ is the transition probability for the spontaneous emission (Einstein’s A value), $N_j(Z^{+r})$ is the number density (i.e., the number of particles per unit volume in $\mathrm{cm}^{-3}$) of the upper level *j* of the emitting ion, and *s* is the line-of-sight coordinate.

The term

2 $$\begin{aligned} P_{ji} = h \nu _{ji}\;N_j(Z^{+r})\;A_{ji}. \end{aligned}$$

is the power (or *emissivity*) per unit volume ($\mathrm{erg}\,\mathrm{cm}^{-3}\,\mathrm{s}^{-1}$) emitted in the spectral line.

When the Sun is observed as a star, the flux (i.e., total irradiance in a line), $F(\lambda _{ji})$, for an optically thin line of wavelength $\lambda _{ij}$ is defined as:

3 $$\begin{aligned} F(\lambda _{ji}) = {1 \over 4 \pi d^2}~ \int _V P_{ji}~ dV \quad (\mathrm{erg}\,\mathrm{cm}^{-2}\,\mathrm{s}^{-1}) \end{aligned}$$

where *d* is the Sun/Earth distance.

There are many processes that affect the population of an upper level of an ion. Those processes occurring between the levels of an ion are, under normal conditions, much faster than those affecting the charged state of the ions, so the two groups of processes (described below) can usually be considered separately. For example, for C iv at $N_{\mathrm{e}}=10^{10}\,\hbox {cm}^{-3}$ and $T=10^5\,\hbox {K}$ we have Mariska (1992) for the allowed transition at 1548 Å the following time scales: collisional excitations and de-excitations: $2 \times 10^{-3}\,\mathrm{s}$; spontaneous radiative decay: $4 \times 10^{-9}\,\mathrm{s}$; radiative recombinations $+$ dielectronic recombinations: $88\,\mathrm{s}$; collisional ionization $+$ autoionization: $107\,\mathrm{s}$.

The population of a level is therefore normally calculated by separately calculating the excited level populations and the ion population. The radiance (intensity) of a spectral line (see Eq. 1) is therefore usually rewritten using the identity:

4 $$\begin{aligned} N_j(Z^{+r}) \equiv {N_j(Z^{+r})\over N(Z^{+r})}\,{N(Z^{+r}) \over N(Z)}\,{N(Z) \over N_{H}}\,{N_{H} \over N_{\mathrm{e}}}\,N_{\mathrm{e}} \end{aligned}$$

where the various terms are defined as follows:

- ${N_j(Z^{+r})/N(Z^{+r})}$ is the population of level *j* relative to the total $N(Z^{+r})$ number density of the ion $Z^{+r}$. As we shall see below, the population of level *j* is calculated by solving the statistical equilibrium equations for the ion $Z^{+r}$. It is a function of the electron temperature and density.
- ${N(Z^{+r})/N(Z)}$ is the ion abundance, and is predominantly a function of temperature, but also has some electron density dependence.
- ${N(Z)/N_{H}} \equiv Ab(Z)$ is the element abundance relative to hydrogen.
- ${N_{H}/N_{\mathrm{e}}}$ is the hydrogen abundance relative to the electron number density. This ratio is usually in the range $\sim 0.8{-}0.9$, since H and He, are fully ionized at coronal temperatures. If we neglect the contribution of the heavier elements to the electron density ($\le$ 1%), and assume fully ionized H and He, the only parameter that changes this ratio is the relative abundance of He, which is variable. If we assume e.g., the Meyer (1985) abundances, $N_{H}/N(\mathrm{He})=10$, then ${N_{H}/N_{\mathrm{e}}}=0.83$.

The intensity of a spectral line can then be written in the form:

5 $$\begin{aligned} I(\lambda _{ji})= \int _s G(N_{\mathrm{e}},T,\lambda _{ji})\,N_{\mathrm{e}}\,N_H\,\mathrm{d}s \end{aligned}$$

with the *contribution function* *G* given by:

6 $$\begin{aligned} G(N_{\mathrm{e}},T,\lambda _{ij}) = Ab(Z)~ A_{ji} ~{h \nu _{ij} \over {4\pi }} {N_j(Z^{+r})\over N_{\mathrm{e}} N(Z^{+r})} {N(Z^{+r}) \over N(Z)} \quad (\mathrm{erg~cm}^{3}~\mathrm{sr}^{-1} ~\mathrm{s}^{-1}) \end{aligned}$$

The contribution function contains all of the relevant atomic physics parameters and for most of the transitions has a narrow peak in temperature, and therefore effectively confines the emission to a limited temperature range. In the literature there are various definitions of contribution function, depending on which terms are included. Aside from constant terms, sometimes the elemental abundance and/or $N_{H}/N_{\mathrm{e}}=0.8$ and/or the term ${1/4 \pi }$ are included in the definition.

Sometimes, as in the CHIANTI package, the contribution function $C(N_{\mathrm{e}},T,\lambda _{ij})$ is calculated without the abundance factor:

7 $$\begin{aligned} G(N_{\mathrm{e}},T,\lambda _{ij}) = Ab(Z)~ C(N_{\mathrm{e}},T,\lambda _{ij}) \end{aligned}$$

Also note that sometimes (as in the CHIANTI *emiss_calc* program) the emissivity is defined as ${ Emiss} = h \nu _{ij}\,N_j(Z^{+r})/N(Z^{+r})\,A_{ji}$, i.e., with the fractional population of the upper level, in which case the emissivity is basically the contribution function without the elemental abundance *Ab*(*Z*) and the ion abundance $N(Z^{+r})/N(Z)$, and without dividing for the electron density.

### Collisional rates and Maxwellian distributions

The dominant mechanisms for the level populations in the low solar corona are collisional excitation and ionization of the ions by the free electrons. Considering excitation, the number of transitions in an ion from a state *i* to a state *j* due to electron collisions, per unit volume and time, is $N_i \sigma _{ij} N_{\mathrm{e}} v f(v) \mathrm{d}v$, where $N_i$ is the number density of the ion in the initial state, $\sigma _{ij}$ is the cross section for the process, *v* is the velocity (in absolute value) of the electron, and *f*(*v*) the distribution function of the electrons.

In general, the collisional excitation rates are proportional to the total number of transitions integrated over the free electron distribution, the so-called rate coefficients

8 $$\begin{aligned} C^{\mathrm{e}}_{ij} = \int _{v_0}^\infty v\,\sigma _{ij} (v)\,f(v)\,\mathrm{d}v \quad ({\mathrm{cm}}^3~\mathrm{s}^{-1}), \end{aligned}$$

where the limit of integration $v_0$ is the threshold velocity, i.e., the minimum velocity for the electron to be able to excite the atom from level *i* to *j*:

9 $$\begin{aligned} {1 \over 2}\,m\,{v_0}^2 = E_j - E_i \end{aligned}$$

where *m* is the mass of the electron.

It is normally assumed that the electrons have enough time to thermalise, i.e., follow a Maxwell–Boltzmann (thermal) distribution (but see Sect. 6.2) in the lower solar corona. In this case, the probability $f(v)\,\mathrm{d}v$ that the electron has a velocity (in the 3-D space) between *v* and $v+\mathrm{d}v$ is:

10 $$\begin{aligned} f(v) = 4\,\pi \,v^2 \left( {m \over 2\,\pi \,kT_{\mathrm{e}}} \right) ^{3/2}\,{\mathrm{e}}^{-{m v^2/2\,kT_{\mathrm{e}}}}. \end{aligned}$$

where *k* indicates Boltzmann’s constant, and $T_{\mathrm{e}}$ the electron temperature. On a side note, the most probable speed $v_p$, i.e., the maximum value of the distribution is found by imposing that ${\mathrm{d} f(v) \over \mathrm{d}v}=0$:

11 $$\begin{aligned} v_p = \left( {2\,k T_{\mathrm{e}} \over m}\right) ^{1/2}, \end{aligned}$$

while the average speed $\langle v\rangle$ is:

12 $$\begin{aligned} \langle v\rangle = \int v\,f(v)\,\mathrm{d}v = \left( {8\,k T_{\mathrm{e}} \over \pi m}\right) ^{1/2} \end{aligned}$$

The collisional excitation rates can then be written using the Maxwell–Boltzmann distribution. As a function of the kinetic energy of the incident electron $E = {1 \over 2}\,m\,{v}^2$, the rate coefficient can be written as:

13 $$\begin{aligned} C^{\mathrm{e}}_{ij}&= \left( {m \over 2 \pi \,k T_{\mathrm{e}}} \right) ^{3/2}\,4 \pi \,\int _{v_0}^\infty v^3\,\sigma _{ij} (v) \,{\mathrm{e}}^{-{m v^2/2\,kT_{\mathrm{e}}}}\,\mathrm{d}v \nonumber \\&= \left( {8 \over \pi \,m} \right) ^{1/2}\,(k T_{\mathrm{e}})^{-3/2} \int _{E_0}^\infty E\,\sigma _{ij} (E)\,{\mathrm{e}}^{-{E/kT_{\mathrm{e}}}}\,\mathrm{d}E \nonumber \\&= 5.287\times 10^{13}\,(k T_{\mathrm{e}})^{1/2} \int _{E_0}^\infty {E \over k T_{\mathrm{e}}}\,\sigma _{ij} (E)\,{\mathrm{e}}^{-{E/kT_{\mathrm{e}}}}\,\mathrm{d}\left( {E \over k T_{\mathrm{e}}}\right) , \end{aligned}$$

where $E_0$ is the threshold energy of the electron, i.e., $E_0 = E_j-E_i$, the energy difference between the ion states *i* and *j*.

In the case of collisional ionization of an atom or ion by a free electron, the expressions for the number of transitions are similar, as described below.

### Excitation and de-excitation of ion levels

Inspection of Eq. (13) indicates a way to simplify the expression, by introducing a dimensionless quantity, the collision strength for electron excitation:

14 $$\begin{aligned} \sigma _{ij} = \pi a_0^2\,\varOmega _{ij}(E)\,{I_{\mathrm{H}} \over g_i E}, \end{aligned}$$

where $g_i$ is the statistical weight of the initial level, $I_{\mathrm{H}}$ is the ionization energy of hydrogen, and $a_0$ the Bohr radius. The collision strength is a symmetrical quantity, such that $\varOmega _{ij}(E)=\varOmega _{ji}(E^{\prime })$, where $E^{\prime }=E-E_{ij}$ is the kinetic energy of the electron after the scattering.

The electron collisional excitation rate coefficient for a Maxwellian electron velocity distribution with a temperature $T_{\mathrm{e}}$ (K) is then obtained by integrating:

15 $$\begin{aligned} C^{\mathrm{e}}_{ij}&= a_0^2 \left( {8 \pi I_{\mathrm{H}} \over m} \right) ^{1/2} \left( {I_{\mathrm{H}} \over k T_{\mathrm{e}}} \right) ^{1/2}{\varUpsilon _{ij} \over g_i} \exp \left( - {E_{ij} \over k T_{\mathrm{e}}} \right) \nonumber \\&={8.63\times 10^{-6} \over T_{\mathrm{e}}^{1/2}} {\varUpsilon _{i,j}(T_{\mathrm{e}}) \over g_i} \exp \left( {- \varDelta E_{i,j} \over kT_{\mathrm{e}}} \right) ~ \mathrm{cm}^3~\mathrm{s}^{-1}, \end{aligned}$$

where *k* is the Boltzmann constant and $\varUpsilon _{i,j}$ is the thermally-averaged collision strength:

16 $$\begin{aligned} \varUpsilon _{i,j}(T_{\mathrm{e}}) = \int _0^\infty \varOmega _{i,j} \exp \left( -{E_{j} \over kT_{\mathrm{e}}}\right) d \left( {E_j \over kT_{\mathrm{e}}}\right) , \end{aligned}$$

where $E_j$ is the energy of the scattered electron relative to the final energy state of the ion. Some details on electron-ion scattering calculations are provided in Sect. 5.

The electron de-excitation rates $C_{j,i}^{\mathrm{d}}$ from the upper level *j* to the lower level *i* are obtained by applying the principle of detailed balance, assuming thermodynamic equilibrium, following Milne, who repeated Einstein’s reasoning on the radiative transition probabilities. In thermodynamic equilibrium, the processes of excitation and de-excitation must equal:

17 $$\begin{aligned} N_i N_{\mathrm{e}} C^{\mathrm{e}}_{i,j} = N_j N_{\mathrm{e}} C^{\mathrm{d}}_{j,i}, \end{aligned}$$

and the populations of the two levels are in Boltzmann equilibrium:

18 $$\begin{aligned} {N_i \over N_j} = {g_i \over g_j} \exp \left( {\varDelta E_{i,j} \over kT_{\mathrm{e}}} \right) , \end{aligned}$$

where $g_i, g_j$ are the statistical weights of the two levels. So we obtain

19 $$\begin{aligned} C_{j,i}^{\mathrm{d}} = {g_i \over g_j} C_{i,j}^{\mathrm{e}} \exp \left( {\varDelta E_{i,j} \over kT_{\mathrm{e}}} \right) . \end{aligned}$$

This relation holds also outside of thermodynamic equilibrium, as long as the plasma is thermal. If the electron distribution is not Maxwellian, the definitions of the excitation and de-excitation rates are somewhat different, as described below in Sect. 6.2.

### The ion level population and the metastable levels

The variation in $N_j$, the population of level *j* of the ion $Z^{+r}$, is calculated by solving the statistical equilibrium equations for the ion including all the important excitation and de-excitation mechanisms:

20 $$\begin{aligned} {{ dN}_j\over dt}&= \sum _{k>j} N_k A_{k,j} + \sum _{k>j} N_k N_{\mathrm{e}} C^{\mathrm{d}}_{k,j} + \sum _{i<j} N_i N_{\mathrm{e}} C^{\mathrm{e}}_{i,j} + \sum _{i<j} N_i B_{i,j}\,J_{\nu _{i,j}} \nonumber \\&\quad +\, \sum _{k>j} N_k B_{k,j}\,J_{\nu _{k,j}}- N_j \left( \sum _{i<j} A_{j,i} + N_{\mathrm{e}} \sum _{i<j} C^{\mathrm{d}}_{j,i} + N_{\mathrm{e}} \sum _{k>j} C^{\mathrm{e}}_{j,k}\right. \nonumber \\&\quad \left. +\, \sum _{i<j} B_{j,i}\,J_{\nu _{j,i}} + \sum _{k>j} B_{j,k}\,J_{\nu _{k,j}}\right) \end{aligned}$$

where the first five terms are processes which populate the level *j*: the first is decay from higher levels, the second is de-excitation from higher levels, the third is excitation from lower levels, and the other two are photo-excitation and de-excitation. The other five terms are the corresponding depopulating processes.

Note:

* $N_{\mathrm{e}}$ ($\hbox {cm}^{-3}$) is the electron number density.
* $C^{\mathrm{e}}, C^{\mathrm{d}}$ ($\hbox {cm}^{-3}\,\hbox {s}^{-1}$) are the electron collisional excitation and de-excitation rate coefficients defined above.
* $A_{j,i}$ ($\hbox {s}^{-1}$) are Einstein’s coefficients for spontaneous emission, also called transition probabilities or A-values.
* $B_{i,j}$ ($i<j$) are Einstein’s coefficients for absorption.
* $B_{k,j}$ ($k>j$) are Einstein’s coefficients for stimulated emission.
* $J_{\nu } = {1 \over 4 \pi } \int I_\nu ({\varvec{\Omega }})\,\mathrm{d}\varOmega ,$ i.e., is the average of the intensity of the radiation field over the solid angle.

Note that the terms associated with stimulated emission by radiation are normally negligible for the solar corona, while the terms associated with absorption of radiation are only important in the outer corona, where electron densities become sufficiently small. Also note that Einstein’s coefficient for stimulated emission is related to the A-value by:

21 $$\begin{aligned} B_{ul} = {c^2 \over 2\,h\,\nu _{ul}^3}\,A_{ul}, \end{aligned}$$

while Einstein’s coefficient for absorption is related to the other two coefficients by:

22 $$\begin{aligned} B_{lu} = {c^2 \over 2\,h\,\nu _{ul}^3}\,{g_u \over g_l}\,A_{ul} = {g_u \over g_l}\,B_{ul}, \end{aligned}$$

where we have indicated the lower level with *l* and the upper level with *u* for simplicity, and *g* indicates the statistical weight of the level. Typical $A_{j,i}$ values for transitions that are dipole-allowed are of the order of $10^{10}\,\hbox {s}^{-1}$, while those of forbidden transitions can be as low as $100\,\hbox {s}^{-1}$.

For most solar (and astrophysical) applications, the time scales of the relevant processes are so short that the plasma is normally assumed to be in a steady state (${{ dN}_j\over dt}=0$). The set of Eq. (20) is then solved for a number of low lying levels, with the additional requirement that the total population of the levels equals the population of the ion: $N(Z^r) =\sum _j N_j$.

In the simplified case of a two-level ion model (a ground state *g* and an excited state *j*) and neglecting other processes such as photoexcitation, we have:

23 $$\begin{aligned} N_{\mathrm{g}} N_{\mathrm{e}} C^e_{g,j} = N_j \left( N_{\mathrm{e}} C^e_{j,g} + A_{j,g}\right) \end{aligned}$$

so the relative population of the level *j* is

24 $$\begin{aligned} {N_j \over N_{\mathrm{g}}} = {N_{\mathrm{e}} C^e_{g,j} \over {N_{\mathrm{e}} C^e_{j,g} + A_{j,g}}} \end{aligned}$$

i.e., depends strongly on the relative values between the radiative rate $A_{j,g}$ and the collisional de-excitation term $N_{\mathrm{e}} C^e_{j,g}$. Levels that are connected to the ground state by a dipole-allowed transition have $A_{j,i}$ values typically several orders of magnitude larger than the de-excitation term (at typical coronal densities, $N_{\mathrm{e}}=10^8{-}10^{12}\,\hbox {cm}^{-3}$), so their population is negligible, compared to the population of the ground state. When all the upper levels of the ion are of this kind, the statistical equilibrium equations are simplified, so that only direct excitations from the ground state need to be included. This is the so called *coronal-model approximation*, where only the electron *collisional excitation* from the ground state of an ion and the *spontaneous radiative decay* are competing.

However, ions often have so called *metastable levels*, *m*, which have a small radiative decay rate (e.g., corresponding to intersystem or forbidden transitions), so that collisional de-excitation starts to compete with radiative decay as a depopulating process at sufficiently high electron densities ($A_{m,g}~\simeq ~N_{\mathrm{e}}~ C^e_{m,g}$). In such cases, the population of the metastable levels becomes comparable to that of the ground state. Whenever ions have metastable levels, it is necessary to include all collisional excitation and de-excitation rates to/from the metastable levels when solving the statistical equilibrium equations. All the ions that have more than one level in the ground configuration have metastable levels (i.e., all the excited levels within the ground configuration), because the transitions within a configuration are forbidden, i.e., they have small radiative decay rates. The ion population is shifted from the ground level into the metastable(s) as the electron density of the plasma increases.

#### Proton excitation

Proton collisions become non-negligible when excitation energies are small, $\varDelta E_{i,j} \ll kT_{\mathrm{e}}$. This occurs, for example, for transitions between fine structure levels, as in the Fe XIV transition in the ground configuration ($3{s}^23{p}\,{}^2\hbox {P}_{1/2}$–$^2\hbox {P}_{3/2}$), as discussed in Seaton (1964a). Normally, only the fine structure levels within the ground configuration of an ion have a significant population, so only the proton collisions among such levels are important. The inclusion of proton excitation has some effects on the relative population of the levels. Proton collisional excitation and de-excitation are easily included as additional terms $C^{\mathrm{p}}$ ($\hbox {cm}^{3}\,\hbox {s}^{-1}$) in the level balance equations.

#### Photoexcitation

Photoexcitation is an important process which needs to be included in the level balance equations when electron densities are sufficiently low. Photoexcitation is the process by which the excitation of an ion from a level i to a level j is caused by absorption of a photon. For this to occur, the photon has to have the same energy of the transition from i to j. From the statistical equilibrium equations (Eq. 20) and considering the relations between the Einstein coefficients, it is obvious that photoexcitation and de-excitation can easily be included as additional terms which modify the A-value. These terms are proportional to $J_{\nu }$. It is common to assume that the intensity of the radiation field originating from the solar photosphere $I_\nu$ does not vary with the solid angle (no limb brightening/darkening), in which case we have:

25 $$\begin{aligned} J_{\nu } = {\varDelta \,\varOmega \over 4 \pi }\,\overline{I_\nu } = W(r)\,\overline{I_\nu } \end{aligned}$$

where *W*(*r*) is the *dilution factor* of the radiation, i.e., the geometrical factor which accounts for the weakening of the radiation field at a distance *r* from the Sun, and $\overline{I_\nu }$ is the averaged disk radiance at the frequency $\nu$.

Assuming spherical symmetry (i.e., the solar photosphere a perfect sphere), and indicating with *r* the distance from Sun centre $R_{\odot }$ the solar radius, we have:

26 $$\begin{aligned} W(r) = {1 \over 4 \pi } \int _0^{2 \pi } \int _0^{\theta _0} { sin}\;\theta \,d\theta \,d\phi = {1 \over 2}\,(1- { cos}\;\theta _0) = {1 \over 2} \left( 1- \left[ 1- \left( {R_{\odot } \over r}\right) ^2\right] ^{1/2} \right) \nonumber \\ \end{aligned}$$

where $\theta _0$ is the angle sub-tending $R_{\odot }$ at the distance *r*, i.e., ${ sin }\theta _0 = R_{\odot }/r$.

In terms of the energy density per unit wavelength, $U_\lambda$, the photoexcitation rate for a transition $i\rightarrow j$ is:

27 $$\begin{aligned} P_{ij}=A_{ji}\,W(r)\,{g_j \over g_i}\,{\lambda ^5 \over 8\pi hc} U_\lambda \end{aligned}$$

where $A_{ji}$ is the Einstein coefficient for spontaneous emission from *j* to $i, g_j$ and $g_i$ are the statistical weights of levels *j* and *i*, and *W*(*r*) is the radiation dilution factor.

Metastable levels affect the population of an ion, in particular those of the ground configuration. The transitions between these levels are normally in the visible and infrared parts of the spectrum, where almost all the photons are emitted by the solar photosphere. Therefore, the main contributions of photoexcitation to the level population is due to visible/infrared photospheric emission. A reasonable approximation for the photospheric radiation field at visible/infrared wavelengths is a black-body of temperature $T_*$, for which the photoexcitation rate becomes:

28 $$\begin{aligned} P^{\mathrm{bb}}_{ij}=A_{ji} W(r) {g_j \over g_i} {1 \over \exp (E/kT_*) -1} \end{aligned}$$

The inclusion of photoexcitation can simply be carried out by replacing the ${A}_{ji}$ value in the statistical equilibrium equations with a generalized radiative decay rate (as coded in the CHIANTI atomic package), which in the black-body case is:

29 $$\begin{aligned} \mathcal{A}_{ij} = \left\{ \begin{array}{l@{\quad }l} W(r) A_{ji} {g_j\over g_i} {1 \over \exp ({\varDelta E}/kT_*) -1} &{} i<j \\ \\ A_{ji} \left[ 1 + W(r) {1 \over \exp ({\varDelta E}/kT_*) -1} \right] &{} i > j \end{array} \right. \end{aligned}$$

Clearly, the photospheric radiation field might depart significantly from black-body radiation, for example with absorption lines, so an observed solar spectrum should be used for more accurate calculations. Photoexcitation typically becomes a significant process at densities of about $10^8\,\hbox {cm}^{-3}$ and below. It therefore becomes a non-negligible effect for off-limb observations of the corona above a fraction of the solar radius, where electron densities (hence electron excitations) decrease quasi-exponentially.

By affecting the populations of the levels of the ground configuration, photoexcitation has a direct effect on the intensities of the (visible and infrared) forbidden lines which are emitted by these levels. One typical example is for the Fe xiii infrared forbidden lines (see, e.g., Chevalier and Lambert 1969; Flower and Pineau des Forets 1973; Young et al. 2003, Fig. 14). These lines are used to measure electron densities (see, e.g., Fisher and Pope 1971), the orientation and strength of the magnetic field and small Doppler shifts in the solar corona. Such measurements are currently being made with the coronal multi-channel polarimeter (CoMP) instrument, now located at Mauna Loa (see, e.g., Tomczyk et al. 2007).

**Fig. 14:**
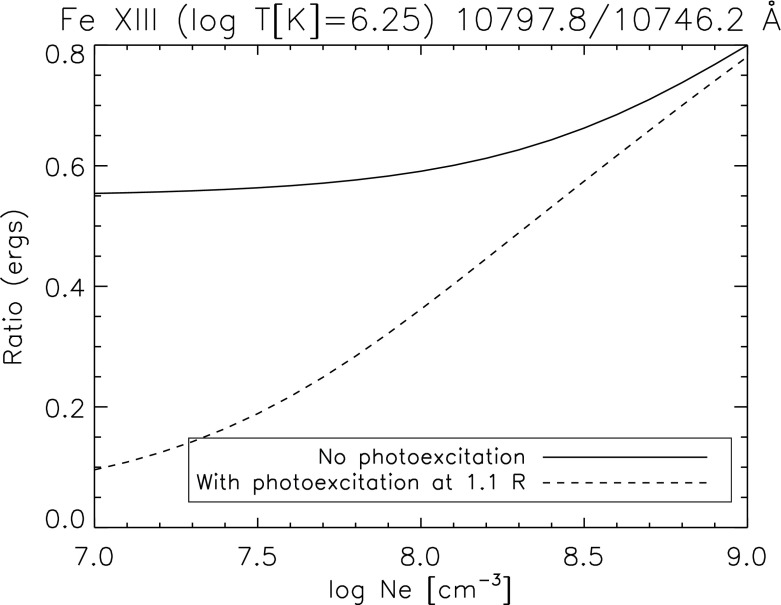
Ratios of the two infrared forbidden Fe xiii lines, as a function of density, without and with photoexcitation (assuming a blackbody spectrum seen at 1.1 $R_\odot$). CHIANTI version 8 was used. The wavelengths indicated are the wavelengths in air

By changing the population of the metastable levels of the ground configuration, photoexcitation also indirectly affects some EUV/UV spectral lines, in particular those connected with the ground configuration metastable levels.

### Atomic processes affecting the ion charge state

Various processes can affect the ionisation state of an element. If we consider two ionization stages, we have the following processes, denoting for simplicity the population of the ion *r*-times ionised with $N_{r}=N(Z^r)$:

1. radiative recombination, induced by the radiation field $\sim N_{r+1} N_{\mathrm{e}} \alpha ^I_{r+1}$;
2. radiative recombination, spontaneous $\sim N_{r+1} N_{\mathrm{e}} \alpha ^R_{r+1}$;
3. photoionisation, induced by the radiation field $\sim N_{r} \alpha ^{\mathrm{PI}}_{r}$;
4. collisional ionization by direct impact of free electrons $\sim N_{r} N_{\mathrm{e}} C^{\mathrm{I}}_{r}$;
5. dielectronic recombination $$\sim N_{r+1} N_{\mathrm{e}} \alpha ^{\mathrm{D}}_{r+1}$$,

where $C^{\mathrm{I}}_{r}$ ($\mathrm{cm}^{3}\,\mathrm{s}^{-1}$) are the rate coefficients for collisional ionization by electrons, and $\alpha$ ($\mathrm{cm}^{3}\,\mathrm{s}^{-1}$) are the various recombination coefficients.

#### Collisional ionisation by electron impact and three-body recombination

The cross section for collisional ionization of an atom or ion from an initial state *i* to a final state *j* by a free electron, differential in the energy $E_1$ of the incident and that of the ejected electron $E_2$ can be expressed, as in the excitation process, in terms of a collision strength $\varOmega$:

30 $$\begin{aligned} \sigma (E_1, E_2) = \frac{1}{k_1^2 g_i} \varOmega _{ij}, \end{aligned}$$

where $g_i$ is the statistical weight of the initial state $i, k_1$ is the kinetic momentum of the incident electron, while the collision strength $\varOmega _{ij}$ can be calculated by replacing the bound orbital in the final state with the free orbital of the ejected electron and summing over its angular momentum (see, e.g., Gu 2008). The total ionization cross section is obtained by integrating over the energy $E_2$ of the ejected electron:

31 $$\begin{aligned} \sigma _{ij}(E_1) = \int \nolimits _0^{\frac{E_1-I}{2}} \sigma _{ij}(E_1, E_2) \mathrm{d}E_2\,, \end{aligned}$$

where *I* is the ionization energy. Note that $$E_1 =I + E_s + E_2$$, where $$E_s$$ is the energy of the scattered electron. By energy conservation, indicating with $$E_i$$ the energy of the *i* state of the ion, we have: $$E_j - E_i = E_1 - E_2 - E_s$$. Also note that the energy of the incident electron must be above threshold: $$E_1 > E_j - E_i $$, and for the ejected electron to exist it must have an energy $$E_2 > E_1 - (E_j - E_i)$$.

The total number of ionisations is found by integrating over the distribution of the incident (free) electron. The total ionisation rate between two ions can be obtained by summing the rate coefficients for each initial state *i* over the final states *j*, and then over all the initial states, although the main contribution to the total is typically the ionisation between the two ground states of the ions.

Note that ionization by direct impact (DI) is the main process, although for some isoelectronic sequences additional non-negligible ionisation can occur via inner-shell excitation into a state above the ionization threshold which then auto-ionizes. This is referred as excitation–autoionization (EA). Goldberg et al. (1965) were among the first to point out the importance of this process, which was later confirmed with experiments (see, e.g., Crandall et al. 1979). The EA provides additional contributions at higher energies of the incident electron, and increases with charge. This contribution is calculated by multiplying the inner-shell excitation cross section with the branching ratios associated with the doubly-excited level.

The main DI process occurs via neighboring ionisation stages, however double ionisation processes can sometimes be non-negligible, as e.g., shown by Hahn et al. (2017). More details and references about these processes can be found below in the atomic data section.

The study by Bell et al. (1983) presented a review of calculated and measured cross sections between ground states of the main ions relevant for astrophysics. This was a landmark paper which formed a reference for a long time. A significant revision of the collisional ionization by direct impact was produced by Dere (2007), where most of the DI and EA cross sections between ground states were recalculated and compared to experimental data, whenever available. Urdampilleta et al. (2017) recently also provided a review of ionisation rates, but without providing new calculations.

Assuming a Maxwellian distribution, and indicating for simplicity with *E* the energy of the incident electron, we have, as in Eq. (13), that the rate coefficient for collisional ionization is:

32 $$\begin{aligned} C^{\mathrm{I}}&= \left( {8 \over \pi \,m} \right) ^{1/2}\,(k T)^{-3/2} \int _{I}^\infty E\,\sigma _{ij} (E)\,{\mathrm{e}}^{-{E/kT}} \mathrm{d}E \nonumber \\&= \left( {8 \over \pi \,m} \right) ^{1/2}\,(k T)^{1/2}\,{\mathrm{e}}^{-{I/kT}} \int _0^\infty (k T x + I)\,\sigma _{ij} (k T x + I)\,{\mathrm{e}}^{-x}\,\mathrm{d}x, \end{aligned}$$

where we have applied the substitution $E = k T x + I$.

The function in the integrand is very steep. For typical temperatures where the ions are formed in the solar corona in equilibrium, the dominant values the cross section to the integral are those from threshold until the peak. The integral in Eq. (32) is of the type

33 $$\begin{aligned} \int _0^\infty f(x)\,{\mathrm{e}}^{-x}\,\mathrm{d}x, \end{aligned}$$

and is therefore often evaluated using a Gauss–Laguerre quadrature: $\sum _i w_i f(x_i)$, where $x_i$ is the root of a Laguerre polynomial, and $w_i$ is the weight:

34 $$\begin{aligned} C^{\mathrm{I}} = \left( {8 \over \pi \,m}\right) ^{1/2}\,(k T)^{1/2}\,{\mathrm{e}}^{-{I/kT}} \sum _i w_i\,\left( x_i + {I\over k T} \right) \,\sigma _{ij} (k T x_i + I). \end{aligned}$$

The numerical factor $\sqrt{8/(\pi \,m)} = 5.287 \times 10^{13}$ as before in the case of excitation. Also note that the analogous expression in the landmark paper by Bell et al. (1983) (their Eq. 8), is incorrect (surprisingly).

Three body recombination is the inverse process of collisional ionization. If we indicate with $C^{\mathrm{I}}_{ij}$ the collisional rate coefficient for ionisation by direct impact of the ion $Z^{+r}$ in its state *i* to the ion $Z^{+r+1}$ in its state *j*, the rate coefficient for the three body recombination $C^{\mathrm{3B}}_{ji}$ can be obtained by applying the principle of detailed balance:

35 $$\begin{aligned} N_{\mathrm{e}}\,N_i(Z^{+r})\,C^{\mathrm{I}}_{ij} = N_{\mathrm{e}}\,N_j(Z^{+r+1})\,C^{\mathrm{3B}}_{ji}, \end{aligned}$$

which leads to

36 $$\begin{aligned} C^{\mathrm{3B}}_{ji} = C^{\mathrm{I}}_{ij} = {g_i \over g_j} {N_{\mathrm{e}} \over 2} \left( {h^2 \over 2\,\pi \,m\,k T_{\mathrm{e}}} \right) ^{3/2}\,{\mathrm{e}}^{-{(E_i -E_f)/kT_{\mathrm{e}}}}\,\end{aligned}$$

in the case of a Maxwellian electron distribution. In the more general case of non-Maxwellian distributions, a simple relation between the rates does not hold. The detail balance applied to the two processes leads to the Fowler relation between the differential cross-sections and the three body recombination rate involves an integral over the energies of the electrons involved in the process.

#### Photoionisation and radiative recombination

Photoionisation is the process by which a photon of energy higher than the ionisation threshold is absorbed by an ion, leaving the ion in the next ionisation stage. For the solar corona, photoionisation is normally a negligible process. For this reason, it is not discussed in detail here.

We note, however, that photoionisation can become important in a number of cases, for example for cool prominence material in the corona, and for low-temperature lines formed in the chromosphere/transition-region, especially during flares, when the photoionising coronal radiation can become significant. This particularly affects lines from H I and He I, He II, as photoionisation is followed by recombination into excited levels, which can then affect the population of lower levels via cascading. For a discussion of this photoionisation–recombination process for He see e.g., Zirin (1975) and Andretta et al. (2003).

The photoionisation rate coefficient from a bound level *i* to the continuum *c* is:

37 $$\begin{aligned} \alpha ^{\mathrm{PI}}_{ic} = 4 \pi \,\int \limits _{\nu _0}^\infty {\sigma ^{\mathrm{(bf)}}_{ic}(\nu ) \over h\nu }\,J_\nu \,\mathrm{d}\nu , \end{aligned}$$

where $\nu _0$ is the threshold frequency below which the bound–free cross section $\sigma ^{\mathrm{(bf)}}_{ic}(\nu )$ is zero.

The photoionisation cross-sections increase roughly as the cube of the wavelength, until threshold. For H-like ions, the modified Kramers’ semi-classical expression is often used:

38 $$\begin{aligned} \sigma ^{\mathrm{(bf)}}_\nu = {64 \pi ^4 m e^{10} \over 3 \sqrt{3} c h^6 }\,{Z^4\,g^{\mathrm{(bf)}} \over n^5\,\nu ^3} = 2.815 \times 10^{-29} {Z^4\,g^{\mathrm{(bf)}} \over n^5\,\nu ^3} \quad \nu \ge \nu _0, \end{aligned}$$

where *n* is the principal quantum number of the level from which the ion of charge *Z* is ionised. $g^{\mathrm{(bf)}}$ is the dimensionless bound–free Gaunt factor (which is close to 1.), introduced as a correction. Values of the bound–free Gaunt factor for H-like ions are tabulated by Karzas and Latter (1961).

Quantum–mechanical calculations of photoionisation cross-sections for ions in general are quite complex. There are two main approaches required to generate opacities: the R-matrix method (Berrington et al. 1987) and the perturbative, or “distorted wave” (DW) method (see, e.g., Badnell and Seaton 2003). The R-matrix method is accurate but computationally expensive. The perturbative approach is much faster but approximates resonances with symmetric line profiles and neglects their interaction with the direct background photoionization. There is generally good agreement between the DW cross sections and the background *R*-matrix photoionization cross sections.

The Opacity Project (OP, see Seaton et al. 1994) involved many researchers under the coordination of M.J. Seaton (UCL) for the calculations of the cross-sections with the R-matrix method. A significant improvement was the inclusion of inner-shell data calculated with the DW method, which formed the Updated OP data (UOP Badnell et al. 2005). Further updates are made available via the web site: http://opacity-cs.obspm.fr/opacity/index.html maintained by F. Delahaye at the Paris Meudon Observatory. On a side note, there is an extended recent literature where other groups used similar approaches but found discordant results. Work within several groups is ongoing, to try and resolve the various issues, as they are of fundamental importance to calculate the opacities for stellar interiors.

Photoionisation cross-sections for transitions from the ground level are often available in the literature as analytic fits to the slow-varying component of the cross-sections (see, e.g., Verner and Yakovlev 1995).

Radiative recombination is the inverse process of photoionisation, i.e., when a free electron recombines with the ion and a photon is emitted. The cross-sections for recombination from a level *f* to a level *i* are normally calculated from the photoionisation cross-sections using the principle of detailed balance in thermodynamic equilibrium, which leads to the Einstein–Milne relation:

39 $$\begin{aligned} \sigma ^{\mathrm{(RR)}}_{fi} (E) = {g_i \over g_f} {(h \nu )^2 \over 2 m E c^2} \sigma ^{\mathrm{(bf)}}_{if}(E). \end{aligned}$$

Radiative recombination rates for all ions of astrophysical interest have recently been obtained by Badnell (2006b) using the photoionisation cross-sections calculated with the DW method (Badnell and Seaton 2003) and the principle of detail balance (i.e., for Maxwellian electron distributions). Finally, we note that the process of radiative recombination, induced by the radiation field, should also be included, although it is normally a negligible process for the solar corona.

#### Dielectronic recombination

Dielectronic recombination occurs when a free electron is captured into an autoionization state of the recombining ion. The ion can then autoionize (releasing a free electron) or produce a radiative transition into a bound state of the recombined ion. The transition can only occur at specific wavelengths. The process of dielectronic recombination was shown by Burgess (1964a, 1965) and Seaton (1964b) to be very important for the solar corona.

By applying the principle of detailed balance, the rate for dielectronic capture should equal the spontaneous ejection of the captured electron, i.e., autoionization. If we indicate with $C^{\mathrm{dc}}_{nj}$ the rate coefficient for the capture of the free electron by the ion $Z^{r+1}$ in the state *n* into a doubly-excited state *j* of the recombined ion $Z^{+r}$, we have

40 $$\begin{aligned} N_n(Z^{r+1}) N_{\mathrm{e}} C^{\mathrm{dc}}_{nj} = N_j(Z^{+r}) A^{\mathrm{auto}}_{jn} \end{aligned}$$

where $A^{\mathrm{auto}}_{jn}$ is the autoionization probability for the transition from the doubly-excited state *j* to the state *n*.

By applying the Saha equation for thermodynamic equilibrium, we obtain

41 $$\begin{aligned} C^{\mathrm{dc}}_{nj} = {h^3 \over (2\pi m kT)^{3/2}} {g_j \over 2 g_n} A^{\mathrm{auto}}_{jn} \exp \left( - {E_j - E_n \over kT} \right) \end{aligned}$$

which is a general formula that also holds outside of thermodynamic equilibrium (as long as the electrons have a Maxwellian distribution), and relates the rate for dielectronic capture to the autoionisation rate (the two inverse processes). The dielectronic capture can then be followed by a radiative stabilization into a bound state of the recombined ion. For coronal plasmas, this normally occurs with a decay of the excited state within the recombining ion.

To calculate the dielectronic recombination coefficient $C^{\mathrm{d}}_{p,u,l}$ of an ion in a state *p* that captures an electron to form a state *u* of the recombined ion, which then decays to a stable state *l* we have

42 $$\begin{aligned} C^{\mathrm{d}}_{p,l,u} (T) = C^{\mathrm{dc}}_{pu}(T) \left[ {A_{ul} \over \sum _k A_{uk} + \sum _q A^{\mathrm{a}}_{uq}} \right] \end{aligned}$$

where the sum over *k* is over all states in the recombined ion $Z^{r}$ that are below *u*, and the sum over *q* is over all possible states of the ion $Z^{r+1}$ and the free electron associated with the autoionization of the doubly-excited state *u*.

Burgess (1964a, 1965) showed that for coronal ions the main DR process is capture into high-lying levels ($n\approx 10$–100), and obtained a general formula for the DR rates that provides a good approximation at low densities and has been used extensively in the literature. More recent DR rates have been computed from the autoionization rate for the reverse process, as described in the atomic data section.

Dielectronic recombination for ions of several isoelectronic sequences have been calculated in a series of papers by Badnell and colleagues (see e.g., Badnell et al. 2003). More details on these atomic data can be found in Sect. 5.

#### Charge transfer

Charge transfer is also an efficient ionisation/recombination process, but only at very low temperatures, and is normally expected to be negligible in the solar corona. In principle, in the low transition region, charge transfer could be an important process for some ions. For example, Baliunas and Butler (1980) discuss the effect of charge transfer between H, He and low charge states of Si, finding that, for example, the Si iii ion population becomes broader in temperature, with a peak around 30,000 K instead of 50,000 K. Such an effect could be significant on a range of spectroscopic diagnostic applications. Such charge transfer effects were included in Arnaud and Rothenflug (1985) but not in subsequent tabulations of ion abundances. In principle, it would be possible to estimate these effects. For example, Yu et al. (1986) used Si iii line ratios to obtain a temperature of about 70,000 K in a fusion device. The temperature of low-Z ions in fusion devices is normally close to that of ionization equilibrium, although particle transport effects could shift the ion populations towards higher temperatures.

#### Charge state distributions

In the case of local thermodynamic equilibrium (LTE), if we write the detailed balance of processes 1., 2., 3., we obtain the Saha equation. At low densities, plasma becomes optically thin and most of radiation escape, therefore processes 1. and 3. are attenuated and the plasma is no longer in LTE. In this condition, the degree of ionization of an element is obtained by equating the total ionization and recombination rates that relate successive ionization stages:

43 $$\begin{aligned} {1 \over N_{\mathrm{e}}} {{ dN}_{r}\over dt} =N_{r-1} S_{r-1} - N_{r} (S_{r} +\alpha _{r}) + N_{r+1}\alpha _{r+1} \end{aligned}$$

for transitions of ion $Z^r$ from and to higher and lower stages, obtaining a set of coupled equations with the additional condition $N(Z)=\sum _r N_{r}$. Here, $S_{r}$ and $\alpha _{r}$ are the total ionization and recombination rates, i.e., those that include all the relevant processes.

**Fig. 15:**
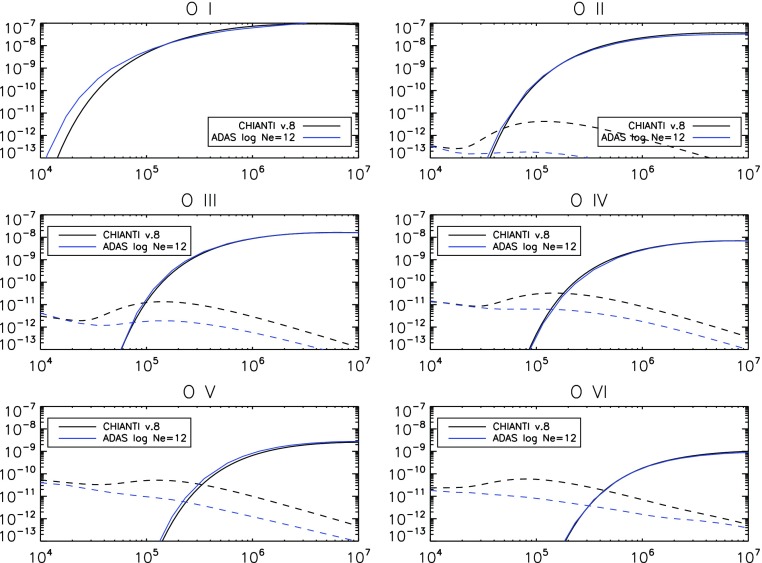
Ionization and recombination (total) rates for a few oxygen ions, as a function of temperature. The first plot in the top left (O i) shows the total ionization rate for neutral oxygen. The second plot (O ii) shows the total ionization rate for O ii, and the total recombination rate from O ii to O i, and so on. The full curves are the ionization rates, while the dashed lines are the recombination rates. The black curves are the CHIANTI v.8 rates (zero density), while the blue ones are the effective rates obtained from OPEN-ADAS, and calculated at a density $N_{\mathrm{e}}=10^{12}\,\hbox {cm}^{-3}$

Figure 15 shows as an example the total ionization and recombination rates for a few oxygen ions, as available with CHIANTI version 8 (black). Note that in addition the plots also show the ionization and recombination effective rates calculated at a density $N_{\mathrm{e}}=10^{12}\,\hbox {cm}^{-3}$, as available in OPEN-ADAS (see next section).

Whenever the time scales of the observed phenomena are less than those for ionization and recombination, we can assume that the population of ions lying in a given state is constant $\left( {{ dN}_{r}\over { dt}} =0\right)$ and so the number of ions leaving this state per unit time must exactly balance the number arriving into that state. This is the so called collisional ionization equilibrium (CIE), which is normally assumed for the solar corona (for very low densities, as in the case of planetary nebulae, photoionisation becomes important and dominates the ion charge state distribution). In the case of two successive stages:

44 $$\begin{aligned} {N_{r+1}\over N_{r}} = {S_{r} \over \alpha _{r+1}}. \end{aligned}$$

Many ionization equilibrium calculations have been published, perhaps the most significant for the iron ions was that one from Burgess and Seaton (1964), where the newly discovered dielectronic recombination was included. Later ones include: Jordan (1969), Arnaud and Rothenflug (1985), Landini and Monsignori Fossi (1991), Arnaud and Raymond (1992) and Mazzotta et al. (1998). Other calculations which also included density effects are those from Summers (1972, 1974).

**Fig. 16:**
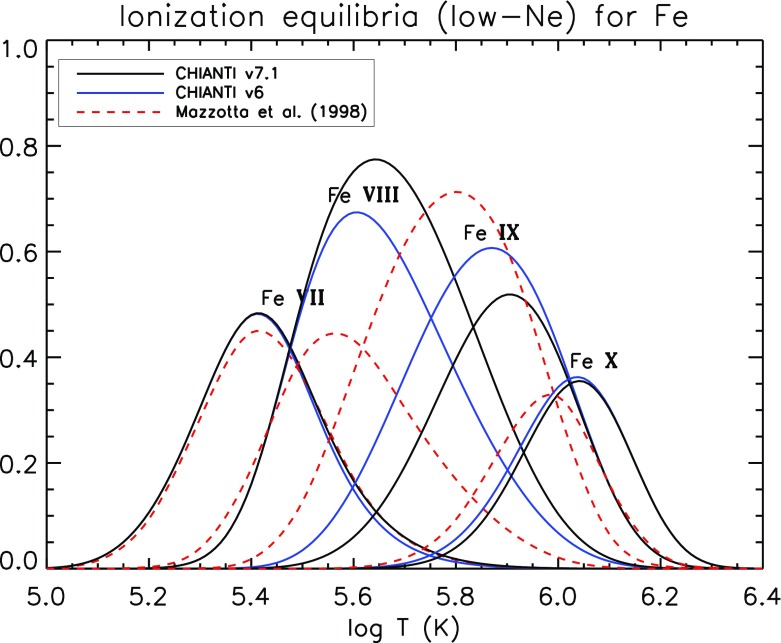
Ion populations of some important coronal ions in equilibrium in the low-density limit. Results from the tabulations of CHIANTI v.6, v.7.1 and Mazzotta et al. (1998) are shown

The improvements in the rates as recalculated in recent years have led to a revision of the ion populations in equilibrium, published by Dere et al. (2009) for CHIANTI version 6. In some cases, significant differences with previous ionisation tables were present. An example is given in Fig. 16. Clearly, such large differences affect any measurements that rely on the ion abundances, such as temperatures from DEM analyses, or relative elemental abundances. Bryans et al. (2009) ion populations are based on almost the same atomic data and rates as used in CHIANTI v.6, so are very similar.

Finally, a few remarks about the formation temperature of a spectral line. This can be quite different from the temperature $T_{\mathrm{max}}$, corresponding to the peak charge state of an ion, a common assumption in the past. This is often found especially in transition region lines, where the temperature gradient of the solar atmosphere is very steep. As we shall see below, there are indications that several structures in the solar corona are nearly isothermal, with temperatures typically very different from $T_{\mathrm{max}}$. This issue is particularly relevant when lines from different ions are used for diagnostic applications such as measuring relative elemental abundances (see Sect. 14).

As a first approximation, an effective temperature $T_{\mathrm{eff}}$ (cf. Eq. 93), where most of the line is formed, can be defined when a continuous distribution of temperatures in the plasma volume is present, as described below in Sect. 7.

Diffusion processes within the transition region, where strong flows and strong temperature gradients exist can also affect the temperature of formation of a line, see e.g., Tworkowski (1976).

#### Density-dependent effects on the ion balances

Most ionization equilibrium calculations are in the so-called *low-density limit* (*the coronal approximation*), where all the population in an ion is assumed to be in the ground state and all the rates are calculated at low densities.

However, as shown by Burgess and Summers (1969), the dielectronic recombination rates decrease significantly at high densities. This is caused by the fact that the intermediate excited states below the ionization limit can easily be re-ionized via electron impact, since this ionization increases linearly with the electron density. When this re-ionization occurs, recombination does not happen. The effect is particularly strong for lower charge states, i.e., transition-region ions, since at typical TR densities the dielectronic recombination becomes suppressed significantly. As a consequence, these ions become populated at progressively lower temperatures. To estimate this suppression, collisional-radiative (CR) modelling was carried out by Summers and Burgess, as described in Summers (1972, 1974), and further refined in following studies.

Recently, Nikolić et al. (2013) suggested an empirical formula to reproduce the suppression factors as calculated by Summers (1974) as a function of the ion charge, isoelectronic sequence, electron density, and temperature. The principle idea was that one would apply these suppression factors to the most recent dielectronic recombination results from the DR project (Badnell et al. 2003), to assess for the importance of this effect for a particular application. If the effect is found to be important, appropriate CR modelling should be carried out. We note that such suppression should only be applied to the total DR rates. One problem with the approach is that the recombination rates calculated by Summers (1974) were actually effective rates, i.e., included many other density effects in addition to the DR suppression, such as the three body recombination, and the changes related to the presence of metastable levels. Another problem is that the Nikolić et al. (2013) formulae were trying to reproduce the Summers (1974) tables which neglected secondary autoionization, with the result that the suppression would be over-estimated, if those factors were applied to the DR project rates, as they included secondary autoionization. Indeed for the boron, carbon, aluminium and silicon sequences Summers (1974) produced tables with secondary autoionization which show less suppression with density. A revision of the Nikolić et al. (2013) formulae is underway.

The effect of including the metastable levels in the ion balance calculations for transition region ions can be as important as the suppression of dielectronic recombination. An approximate calculation for C iv was carried out by Vernazza and Raymond (1979) using a rough estimate of the DR suppression based on Summers (1974) (assuming that it would be the same for all fine-structure levels), and adding collisional ionisation from the metastable levels in C iii. The two effects appeared equally important.

One way to estimate these effects is the generalised collisional-radiative (GCR) modelling (McWhirter and Summers 1984; Summers et al. 2006) which has been implemented within the Atomic Data and Analysis Structure (ADAS). Once the level-resolved ion balance equations are solved, *effective* ionisation and recombination rates can be obtained. These rates are currently available via OPEN-ADAS.^2^

Figure 15 shows as an example the total effective ionization and recombination rates (blue curves) for a few oxygen ions, calculated at a density $N_{\mathrm{e}}=10^{12}\,\hbox {cm}^{-3}$, as available in OPEN-ADAS (1996 version), compared to the CHIANTI v.8 rates (black curves, zero density). The effect of the suppression in the DR rates is clearly present in the ADAS effective recombination rates.

The effect of these processes on diagnostic ratios and predicted radiances has been discussed in the literature (cf. Vernazza and Raymond 1979; Del Zanna et al. 2002; Doyle et al. 2005). Generally, the predicted radiances increase by factors of 2–3, thus reducing the discrepancies with observation for the so-called anomalous ions, i.e., those that have predicted radiances typically a factor of 5–10 lower than observed, as discussed below in Sect. 7.3.

**Fig. 17:**
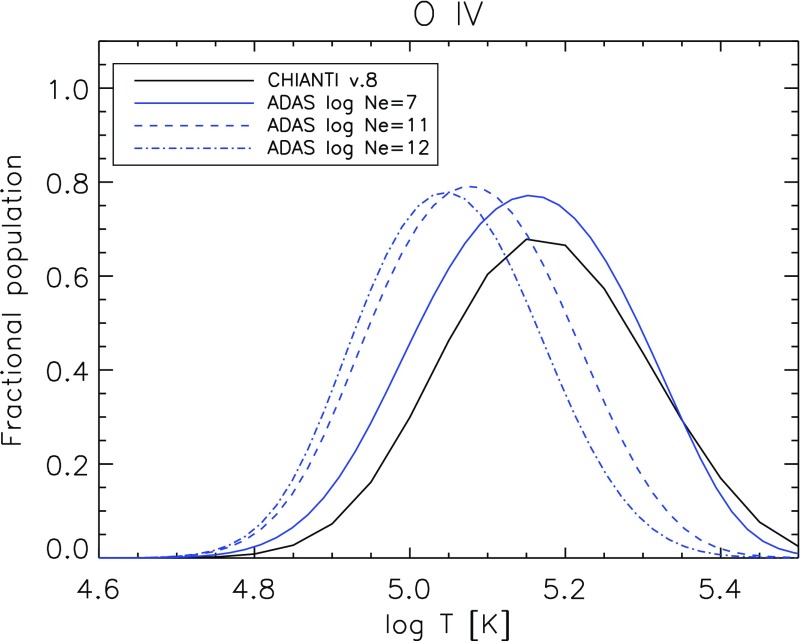
Fractional O iv abundances in equilibrium at different electron densities (blue, from OPEN-ADAS), and the low-density values as in CHIANTI version 8 (black)

The density effect on the ion charge state distribution are particularly important for the IRIS O iv and S iv lines, as shown by Polito et al. (2016a). Figure 17 shows as an example the fractional O iv abundance in equilibrium at different electron densities (blue) as calculated with the OPEN-ADAS rates and the low-density value as in CHIANTI version 8.

### Optical depth effects

In most cases, spectral lines in the XUV originating from the solar corona and transition region are optically thin, i.e., the emitted photons freely escape. There are however several cases, especially for strong transition-region lines, where lines could be optically thick. One question naturally arises: how can we check if a spectral line is optically thin? There are various ways. One obvious effect of optical depth effects is a flattening, widening (or even self-reversal of the core) of the line profile. Estimates of the optical depth of lines from their width and their centre-to-limb variation have been obtained by e.g., Roussel-Dupre et al. (1979) and Doschek and Feldman (2004). However, a shape that is for example non-Gaussian does not necessarily mean that a line is not optically thin. In fact, a non-Gaussian shape can be produced by a superposition of Doppler motions. We further discuss such effects together with other broadening mechanisms below in Sect. 13.

**Table 2:** Some UV line ratios that can be used to investigate optical depth effects

Ion	Terms	Wavelengths (Å)	Ratio
C II	$^2\hbox {P}_{3/2}{-}{}^2\hbox {S}_{1/2}/{}^2\hbox {P}_{1/2}{-}{}^2\hbox {S}_{1/2}$	1037.02/1036.33	2
Si II	$^2\hbox {P}_{3/2}{-}{}^2\hbox {S}_{1/2}/{}^2\hbox {P}_{1/2}{-}{}^2\hbox {S}_{1/2}$	1533.43/1526.71	2
C III	$^3\hbox {P}_{0}{-}{}^3\hbox {P}_{1}/^3\hbox {P}_{2}{-}{}^3\hbox {P}_{1}$	1175.26/1176.37	0.8
Si III	$^3\hbox {P}_{0}{-}{}^3\hbox {P}_{1}/^3\hbox {P}_{2}{-}{}^3\hbox {P}_{1}$	1296.73/1303.32	0.8
C III	$^3\hbox {P}_{2}{-}{}^3\hbox {P}_{2}/{}^3\hbox {P}_{1}{-}{}^3\hbox {P}_{2}$	1175.71/1174.93	3
Si III	$^3\hbox {P}_{2}{-}{}^3\hbox {P}_{2}/{}^3\hbox {P}_{1}{-}{}^3\hbox {P}_{2}$	1298.94/1294.54	3
Si IV	$^2\hbox {S}_{1/2}{-}{}^2\hbox {P}_{3/2}/{}^2\hbox {S}_{1/2}{-}{}^2\hbox {P}_{1/2}$	1393.75/1402.77	2
C IV	$^2\hbox {S}_{1/2}{-}{}^2\hbox {P}_{3/2}/{}^2\hbox {S}_{1/2}{-}{}^2\hbox {P}_{1/2}$	1548.20/1550.77	2
N V	$^2\hbox {S}_{1/2}{-}{}^2\hbox {P}_{3/2}/{}^2\hbox {S}_{1/2}{-}{}^2\hbox {P}_{1/2}$	1238.82/1242.80	2
O VI	$^2\hbox {S}_{1/2}{-}{}^2\hbox {P}_{3/2}/{}^2\hbox {S}_{1/2}{-}{}^2\hbox {P}_{1/2}$	1031.91/1037.61	2
Ne VIII	$^2\hbox {S}_{1/2}{-}{}^2\hbox {P}_{3/2}/{}^2\hbox {S}_{1/2}{-}{}^2\hbox {P}_{1/2}$	770.41/780.32	2

The last column indicates the theoretical value in the optically thin case

A direct way to estimate if lines are optically thin is to consider the ratios of lines that originate from a common upper level (a branching ratio). In the optically thin case, the ratios are equal to the ratios of the A-values (see, e.g., Jordan 1967), which are normally known to a good accuracy. If opacity effects are present, the ratio would be different. Table 2 lists a few commonly used ratios from C II, Si II, Si III, and C III (see, e.g., Doyle and McWhirter 1980; Keenan and Kingston 1986; Brooks et al. 2000; Del Zanna et al. 2002).

Another direct method applies to the doublets of the Li-like ions. In the optically thin case, the ratios of the two lines of the doublet should be equal to the ratio of the oscillator strengths, i.e., two. Due to opacity effects, the intensity of the brightest component, the $^2\hbox {S}_{1/2}{-}{}^2\hbox {P}_{3/2}$ typically decreases (relative to the weaker one), because it has a larger oscillator strength.

From the observed departure of a ratio from the optically thin case, the optical depths of the lines and the path lengths of the emitting layer can be obtained. The process is not straightforward though. Various approximations and approaches exist in the literature, involving the probability that a photon emitted from a layer of certain optical depth will escape along the line of sight. For details, see, e.g., Holstein (1947), Jordan (1967), Irons (1979), Doyle and McWhirter (1980), Kastner and Kastner (1990) and Brooks et al. (2000) and references therein.

We now introduce a simplified discussion of optical depth. The one-dimensional radiative transfer equation for the specific intensity of the radiation field that propagates, at frequency $\nu$ along the direction ${\varvec{\Omega }}$ inside a plasma is:

45 $$\begin{aligned} {\mathrm{d} \over \mathrm{d}s} I_\nu ({\varvec{\Omega }}) = -k_\nu ^{(\mathrm{a})}\,I_\nu ({\varvec{\Omega }})\,+k_\nu ^{(\mathrm{s})} I_\nu ({\varvec{\Omega }})\,+\epsilon _\nu , \end{aligned}$$

where *s* is the spatial coordinate measured along the direction of propagation, and the three quantities $k_\nu ^{(\mathrm{a})}, k_\nu ^{(\mathrm{s})}$, and $\epsilon _\nu$, are the absorption coefficient, the coefficient of stimulated emission, and the emission coefficient, respectively. The equation is often written in this form:

46 $$\begin{aligned} {\mathrm{d} \over \mathrm{d}s} I_\nu ({\varvec{\Omega }}) = -k_\nu \,[I_\nu ({\varvec{\Omega }}) - S_\nu ], \end{aligned}$$

where the source function $S_\nu = \epsilon _\nu /k_\nu$ and $k_\nu = k_\nu ^{(\mathrm{a})} - k_\nu ^{(\mathrm{s})}$.

The specific optical depth

47 $$\begin{aligned} \mathrm{d} \tau _\nu = - k_\nu \,\mathrm{d}s \end{aligned}$$

is defined in the direction opposite to that of the propagation of the radiation, which reflects the point of view of an observer receiving the radiation. The optical thickness at frequency $\nu$ of a plasma slab of geometrical thickness *D* is defined as:

48 $$\begin{aligned} \tau _\nu = \int _0^D k_\nu (s)\,\mathrm{d}s. \end{aligned}$$

A plasma is optically thick when $\tau _\nu \gg 1$, i.e., when the photon has a probability practically equal to unity to be absorbed within the slab.

Considering a simple two-level atom with lower and upper level populations $N_l, N_u$, it is possible to show that these monochromatic coefficients are related to Einstein’s coefficients:

49 $$\begin{aligned} k_\nu ^{(\mathrm{a})}= & {} {h\,\nu \over 4 \pi }\,N_l\,B_{lu}\,\phi (\nu - \nu _0); \quad k_\nu ^{(\mathrm{s})} = - {h\,\nu \over 4 \pi } \,N_u B_{ul}\,\chi (\nu - \nu _0);\quad \nonumber \\ \epsilon _\nu= & {} {h\,\nu \over 4 \pi }\,N_u\,A_{ul}\,\psi (\nu - \nu _0) \end{aligned}$$

where $\phi (\nu - \nu _0), \chi (\nu - \nu _0), \psi (\nu - \nu _0)$ are the area-normalised line profiles for the extinction, induced emission, and spontaneous emission, respectively. $\nu _0$ indicates the line centre frequency.

When the incident (exciting) and emitted photons are not correlated, we have *complete redistribution* and the three local line profiles are equal, in which case the source function $S_\nu$ becomes frequency independent:

50 $$\begin{aligned} S_{\nu _0} = {N_u\,A_{ul} \over N_l\,B_{lu} - N_u B_{ul}}, \end{aligned}$$

and the bound–bound opacity of a line can be expressed in terms of the A-value as:

51 $$\begin{aligned} \tau _\nu = \int _0^D {c^2 \over 8\,\pi \,\nu ^2}\,{g_u \over g_l} \,N_l\,A_{ul}\,\left\{ 1- {N_u g_l \over N_l g_u} \right\} \,\phi (\nu - \nu _0) \mathrm{d}s, \end{aligned}$$

which could also be written in terms of the oscillator strength $f_{lu}$ of the transition, using the relation:

52 $$\begin{aligned} g_u\,A_{ul} = {8 \pi ^2\,e^2\,\nu _{ul}^2 \over m\,c^3}\,g_l \,f_{lu}. \end{aligned}$$

The line centre optical thickness (or opacity) is an useful quantity to assess if a spectral line is optically thin:

53 $$\begin{aligned} \tau _{\nu _0} = \int k_{\nu _0} N_l \mathrm{d}s, \end{aligned}$$

where $k_{\nu _0}$ is the absorption coefficient at line center frequency $\nu _0$ which can be written, neglecting the induced emission and assuming a Doppler line profile:

54 $$\begin{aligned} k_{\nu _0} = {h\,\nu _0 \over 4 \pi }\,B_{lu} {1 \over \pi ^{1/2} \,\varDelta \nu _D}, \end{aligned}$$

where $\varDelta \nu _D$ is the Doppler width of the line, in frequency (for more details on Doppler widths see Sect. 13).

Considering the relations between Einstein’s coefficients and the oscillator strength, and the expression for the population of the lower level (cf. previous notation):

$$\begin{aligned} N_l = {N_l \over N(Z^{+r})}\,{N(Z^{+r}) \over N(Z)}\,Ab(X)\,\frac{N_{\mathrm{H}}}{N_{\mathrm{e}}}\,N_{\mathrm{e}}, \end{aligned}$$

the opacity at line centre can be written as:

55 $$\begin{aligned} \tau _{0} = {\pi \,e^2\,\over m\,c} {1 \over \pi ^{1/2}\,\varDelta \nu _D} f_{lu}\,D = {\pi ^{1/2}\,e^2\,\over m\,c\,\varDelta \nu _D} f_{lu}\,D\,{N_l \over N(Z^{+r})}\,{N(Z^{+r}) \over N(Z)}\,Ab(X)\,\frac{N_{\mathrm{H}}}{N_{\mathrm{e}}}\,N_{\mathrm{e}}, \nonumber \\ \end{aligned}$$

where $N_{\mathrm{e}}$ is the average electron density of the plasma emitting the line, and *D* is the path length along the line of sight through the source. This is a very useful definition commonly used, and easy to estimate, once the various factors are known. If $\tau _{0}$ is of the order of one or less, opacity effects can be neglected. Even if $\tau _{0}$ is much larger, it does not mean that photons do not escape. For example, values of $\tau _{0} \simeq 10^4$ are needed for no photons to escape (Athay 1971).

With the above definition, we can show that even the strongest lines in the transition region are not affected by opacity in typical quiet Sun regions. Following Mariska (1992), we consider one of the strongest lines, the C iv 1548 Å resonance transition, which has $f=0.2$. Assuming typical quiet Sun values of $N_{\mathrm{e}}=10^{10}\,\hbox {cm}^{-3}$ and $T=10^5\,\hbox {K}$ and observed values of the line width we have $\tau _{0} \simeq 10^{-8}\,D$ as an order of magnitude (this value can differ depending on the chosen element and ion abundances). Therefore, a slab thickness larger than $10^{8}\,\hbox {cm}$ would be needed before opacity effects come into play. This is more than the typical size of the region where C iv is emitted. Indeed opacity effects are typically seen in lower temperature lines formed in the chromosphere or occasionally in active regions.

Another effect that often decreases the intensity of a spectral line is absorption by cool plasma that is along the line of sight. The main absorption is by neutral hydrogen (with an edge at 912 Å), neutral helium (with an edge at 504 Å) and ionised helium (with an edge at 228 Å). Such effects were noticed long ago from Skylab observations (see e.g., Orrall and Schmahl 1976). They are particularly evident when surges or filaments are present.

From the observed absorption at different wavelengths and some assumptions about the underlying emission, it is possible to estimate the column density of the hydrogen and helium absorbing plasma. Kucera et al. (1998) used SOHO CDS while Del Zanna et al. (2004b) combined SOHO CDS with SUMER. Others have used the absorption seen in EUV images, such as SDO AIA (see, e.g., Williams et al. 2013).

### Continuum radiation

*Free–free* emission is produced when an electron interacts with a charged particle *Z* and looses its kinetic energy $E= m v^2/2$ releasing a photon of energy $h\nu$: $Z + e(E) \Longrightarrow Z + e(E') + h\nu$. The process is called *bremsstrahlung* (‘braking radiation’). The emission is a continuum. The calculation of the free–free emission in the classical way can be found in textbooks, and is based on the radiation emitted by a charged particle in the Coulomb field of the ion, with an impact parameter *b*. Classically, the minimum impact parameter is set so the kinetic energy of the free electron is greater than the binding energy: $E > {e^2 Z \over b}$, otherwise we would have recombination. With a quantum–mechanical treatment, a correction factor needs to be introduced. This need to take into account that $m v^2/2 \ge h\nu$, otherwise a photon could not be created. The correction is the free–free Gaunt factor $g_{\mathrm{ff}}(\nu v)$ which is close to unity and has a weak dependence on the frequency $\nu$ and the electron velocity/energy. The energy emitted per unit time, volume and frequency is

56 $$\begin{aligned} \frac{dW}{dt dV d\nu }= \frac{16 \pi }{3^{3/2}} \frac{Z^{2}e^{6}}{m^{2} v c^{3}} N_{\mathrm{e}} N_{\mathrm{i}} g_{\mathrm{ff}} \end{aligned}$$

where $N_{\mathrm{e}} N_{\mathrm{i}}$ are the electron and ion number densities. We note that slight different definitions are found in the literature, depending on how the Gaunt factor is defined. We follow Rybicki and Lightman (1979).

For a Maxwellian velocity distribution of electrons the process is called *thermal bremsstrahlung*. The calculation of the emitted energy per unit of time and volume requires an integration over the electron velocity distribution. The integral with the Gaunt factor produces the mean Gaunt factor $\langle g_{\mathrm{ff}}\rangle$ (see, e.g., Karzas and Latter 1961). We obtain:

57 $$\begin{aligned}&\displaystyle \frac{dW}{dt dV d\nu } = \frac{32 \pi e^6}{3 m c^3} \Big (\frac{2 \pi }{3 k m}\Big )^{1/2}~\frac{Z^{2} N_{\mathrm{e}} N_{\mathrm{i}}}{T_{\mathrm{e}}^{1/2}}~{\mathrm{e}}^{-{h\nu /kT_{\mathrm{e}}}}~\langle g_{\mathrm{ff}}\rangle \end{aligned}$$

58 $$\begin{aligned}&\displaystyle \frac{dW}{dt dV d\nu } = 6.8\times 10^{-38} \frac{Z^{2} N_{\mathrm{e}} N_{\mathrm{i}}}{T_{\mathrm{e}}^{1/2}}~{\mathrm{e}}^{-{h\nu /kT_{\mathrm{e}}}}~\langle g_{\mathrm{ff}}\rangle \,\,\, \mathrm{erg\,cm}^{3}\,\mathrm{s}^{-1}\,\mathrm{Hz}^{-1} \end{aligned}$$

where *k* is Boltzmann constant and *h* is the Planck constant.

Free–free emission is the main radiative loss mechanism for low density plasmas at $T>10^7\,\hbox {K}$.

*Free–bound* emission is produced when a free electron of energy *E* is captured by an ion ($Z^{r+1}$) into a bound state of $Z^{r}$:

$$\begin{aligned} Z^{r+1} + e(E) \Rightarrow Z_{n}^{r} + h \nu \end{aligned}$$

a photon of energy $h \nu = E + I_{n}$ is emitted and $I_n$ is the ionization energy of the bound state *n*. For a Maxwellian electron velocity distribution, the continuum emission is characterized by discontinuities at the ionization thresholds.

The free–bound continuum emissivity produced from recombination onto an ion of charge *Z* can be written as

59 $$\begin{aligned} P_{\mathrm{fb},\lambda }= & {} 3.0992 \times 10^{-52} N_{\mathrm{e}} N_{Z+1} {E_\lambda ^5 \over T^{3/2}} \sum _i {\omega _i \over \omega _0} \sigma ^{\mathrm{bf}}_i \exp \left( - {E_\lambda - I_i \over kT} \right) \quad \nonumber \\&\times (\mathrm{erg}~\mathrm{cm}^{-3}~\mathrm{s}^{-1}~\mathrm{~\AA }^{-1}) \end{aligned}$$

where $N_{\mathrm{e}}$ and $N_{Z+1}$ are the number densities of electrons and recombining ions, respectively, in units of $\hbox {cm}^{-3}; E_\lambda$ is the energy in $\hbox {cm}^{-1}$ of the emitted radiation; *T* is the plasma temperature in K; $\omega _i$ is the statistical weight of the level *i* in the recombined ion; $\omega _0$ is the statistical weight of the ground level of the recombining ion; $\sigma ^{\mathrm{bf}}_i$ is the photoionization cross-section from the level *i* in the recombined ion to the ground level of the recombining ion, in units of Mb ($=10^{-18}\,\hbox {cm}^2$); $I_i$ is the ionization energy in units of $\hbox {cm}^{-1}$ from the level *i* in the recombined ion, and the sum is over all levels *i* below the recombined ion’s ionization limit.

Finally, there is another process which produces continuum radiation, the so-called *Two-photon* continuum. It is caused by two-photon decay processes in H-like and He-like ions. Compared to the free–free and the bound–free, this continuum is nearly negligible, except at low temperatures $\hbox {T}\le 3 \times 10^4\,\hbox {K}$. However, it is important in the population modelling of such ions. The transition from the metastable $1s\,2s\, ^1\hbox {S}_0$ state of Helium-like ions to their ground state $1{s}^2\,{}^1\hbox {S}_0$ is strictly forbidden, hence the two-photon process becomes an important depopulating process. The same occurs in H-like ions, where the transition from the metastable $2s\,{}^2\hbox {S}_{1/2}$ state to the ground state $1s\,{}^2\hbox {S}_{1/2}$ is also strictly forbidden. Calculations of the rates for these processes have been carried out by several authors, see e.g., Drake (1986) for He-like and Parpia and Johnson (1982) for the H-like ions.

**Fig. 18:**
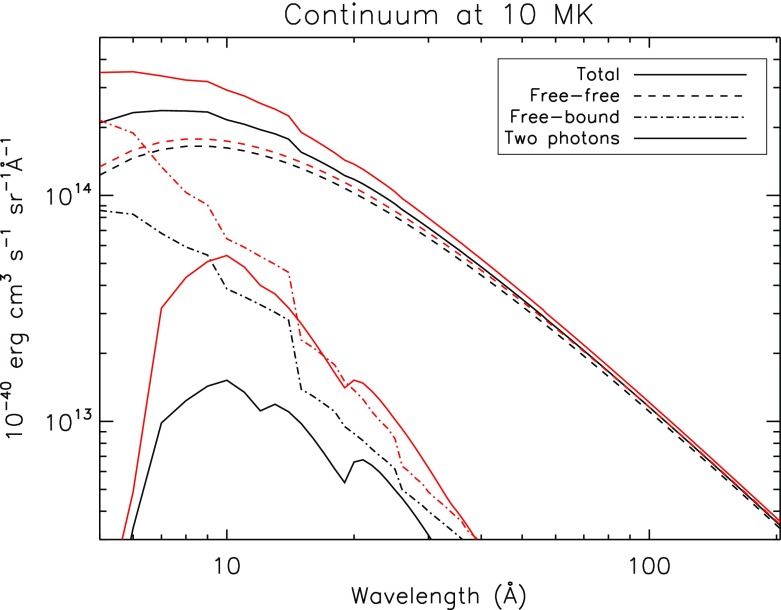
The continuum calculated with CHIANTI version 8 at a temperature of 10 MK, from 5 to 200 Å. The black curves are calculated with the photospheric abundances recommended by Asplund et al. (2009), while the red ones with the ‘coronal’ abundances of Feldman (1992a)

The available atomic data (including relativistic effects) for the continuum were assessed for CHIANTI version 3 by Young et al. (2003). Figure 18 shows the continuum calculated with CHIANTI version 8 at a temperature of 10 MK, from 5 to 200 Å. The black curves are calculated with the photospheric abundances recommended by Asplund et al. (2009), while the blue ones with the ‘coronal’ abundances of Feldman (1992a). Note that at the EUV wavelengths, most of the continuum is free–free radiation. Also note the significant variation with elemental abundances in the X-rays. This means that when chemical abundances are estimated from line-to-continuum measurements (see below in Sect. 14), a self-consistent approach needs to be adopted.

## The satellite lines

Satellite lines were discovered by Edlén and Tyrén (1939) in laboratory vacuum spark spectra of the He-like carbon. They were called satellites because they were close to the resonance line, mostly at longer wavelengths, although some were also present at shorter wavelengths. They were correctly interpreted as $1{s}^2\,nl$–$1s\,2p\,nl$ transitions, satellites of the He-like resonance transition $1{s}^2{-}1{s}\,2p$ (the so-called *parent line*). They also observed the $1{s}^2\,nl$–$1s\,3p\,nl$ transitions, satellites of the He-like $1{s}^2$–$1s\,3p$ transition, and the $1s\,nl{-}2{p}\,nl$ satellites of the H-like 1*s*–2*p* resonance line.

Satellite lines have later been observed in solar spectra, and soon it was recognised that they have many important and unique diagnostic applications.

**Fig. 19:**
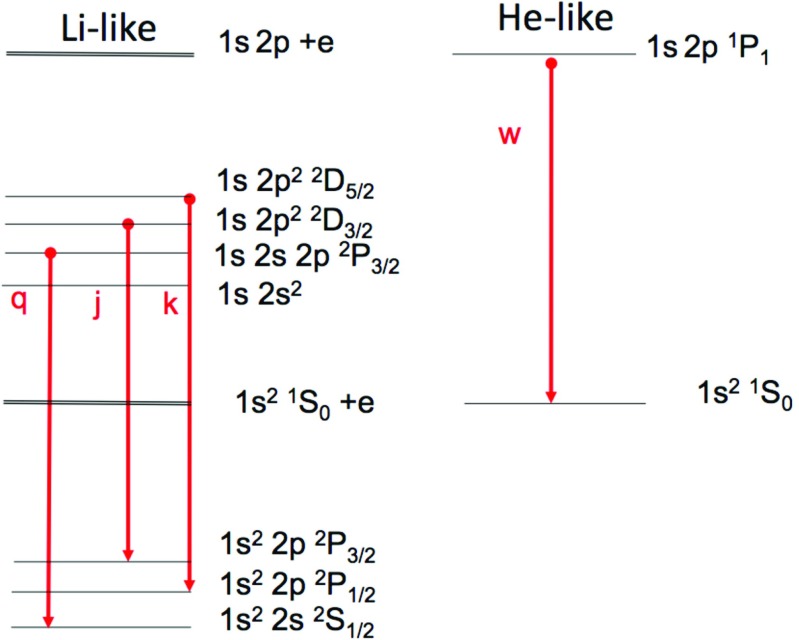
A sketch of a few of the main satellite lines of the He-like resonance line *w*

The satellite lines of the He-like ions involve the presence of doubly-excited states of the Li-like ions, which can then auto-ionize or produce the satellite line (see Fig. 19). The doubly excited states exist because of the interaction with at least one continuum, in this case, the continuum of the ground state of the He-like ions. The formation of the doubly-excited state follows the usual selection rules in that the autoionising state and the final state plus free electron have to have the same parity and total angular momentum *J* (and *L* and *S* in case of LS coupling).

In principle the doubly excited states can be formed by inner-shell electron impact ionisation of the Be-like ion or by two-electron impact excitation, but these processes are normally negligible, compared to the inner-shell excitation of one electron in the Li-like ion (which can subsequently auto-ionize or produce the satellite line), or dielectronic capture, the inverse process of autoionisation. Both processes need to be calculated. The relative importance of the two then depends on the relative values of the relevant rates, as well as the status of the ion (i.e., if it is out of equilibrium, ionising or recombining).

Dielectronic capture occurs when a free electron is captured into an autoionization state of the recombining Li-like ion. The ion can then autoionize (releasing a free electron) or produce a satellite line, i.e., a transition into a bound state of the recombined ion. The transitions occur at specific wavelengths, close to the wavelength of the He-like resonance line. As we have mentioned in Sect. 3.5, Burgess with Seaton developed the theory of dielectronic recombination, however those calculations were focused on the states where the spectator electron is in a highly excited state with *n* typically around 100, as these give the highest contribution to the total dielectronic recombination of an ion.

On the other hand, for spectral diagnostic purposes, the transitions that are important and are observed are those arising from lower autoionising levels.

Early theories and computations for these satellite lines were initially carried out by Gabriel and colleagues in the late 1960s and early 1970s, and later by a number of researchers, in Europe, Russia and USA. Notable papers, where the main diagnostics and notation were developed for the He-like lines are Gabriel and Paget (1972) and Gabriel (1972a). Several excellent laboratory observations of the satellite lines exist, taken by Doschek, Feldman, Presnyakov, Boiko, Aglitskii and several other colleagues (see, e.g., Aglitskii et al. 1974; Feldman et al. 1974; Boiko et al. 1977, 1978a, b). There are several excellent in-depth reviews on the satellite lines, such as Dubau and Volonte (1980), Doschek (1985, 1990), Mewe (1988) and Phillips et al. (2008).

Below, we summarise the main processes and highlight some of the main diagnostic applications for the solar plasma. Many more diagnostic applications are available for other types of plasma.

### Inner-shell of Li-like ions

We consider first the inner-shell excitation. The simultaneous excitation of two electrons has a low probability and therefore the $1s\,2{p}^2$ population comes mainly from excitation of the excited state $1{s}^2\,2p$. On the other hand, the $1s\,2s\, 2p$ can be excited from either $1{s}^2\,2s$ or $1{s}^2\,2p$, although the first one usually dominates. Following Gabriel (1972a), the intensity of the satellite line from level *s* to the final level *f* produced by inner-shell excitation is (in photon units):

60 $$\begin{aligned} I^{\mathrm{inner}}_{{ sf}} = \beta \,N_{\mathrm{Li}{\text {-}}\mathrm{like}}\,N_{\mathrm{e}}\,C^e_{{ is}} \,{A_{{ sf}} \over \left( \sum _k A^{\mathrm{auto}}_{sk} + \sum _{f<s} A_{{ sf}} \right) } \sim \beta N_{\mathrm{Li}{\text {-}}\mathrm{like}}\,N_{\mathrm{e}}\,C^e_{{ is}}\,{A_{{ sf}} \over A^{{ tot}}_s}, \end{aligned}$$

where $$ N_{\mathrm{Li}{\text {-}}\mathrm{like}}$$ is the abundance of the Li-like ion; $$ C^e_{{ is}}$$ is the electron impact excitation rate coefficient by inner-shell; $$A_{{ sf}}$$ the transition probability by spontaneous emission from level *s* to the final level $$f; \sum _k A^{\mathrm{auto}}_{{ sk}}$$ is the total decay rate via autoionisation from level *s* to into all available continua *k* in the He-like ion; and $$\sum _{f<s} A_{{ sf}} $$ is the total decay rate by spontaneous radiative transitions to all possible final states *f*. For He-like ions, the main autoionisation is to the ground configuration of the recombining ion (which is a single level), so the $$\sum _k A^{\mathrm{auto}}_{sk}$$ is normally replaced by $$A^{\mathrm{auto}}_s$$, the total autoionisation rate into the He-like ground-level continua. The ratio of A values $$A_{{ sf}}/A_s^{{ tot}}$$ on the right of the equation is basically a branching ratio. This is normally small, meaning that the majority of inner-shell excitations decay by autoionisation, rather than by emission of the satellite line.

$\beta \,N_{\mathrm{Li}{\text {-}}\mathrm{like}}$ is the population of the lower level which populates the level *s* by collisional excitation. The value of $\beta$ is obtained by solving the level population for the Li-like ion. At coronal densities, the population of the Li-like ions is all in the ground state, so $\beta =1$ for the $1s\,2s\, 2p$, and $\beta =0$ for the $1s\,2p^2$. At increasing densities, $\beta$ varies but can easily be calculated.

The ratio of the satellite line produced by inner-shell excitation to the resonance line *w* in the He-like ion is an excellent diagnostic for measuring the relative population of the Li-like versus the He-like ion, i.e., to assess if departures from ionisation equilibrium are present. In fact, since most of the population of the He-like ion $N_{\mathrm{He}{\text {-}}\mathrm{like}}$ is in the ground state *g*, the intensity of the resonance line *w* can be approximated with

61 $$\begin{aligned} I_w = N_u A_{ug} \simeq N_{\mathrm{He}{\text {-}}\mathrm{like}} N_{\mathrm{e}} C^e_{{ gu}} \end{aligned}$$

where $C^e_{{ gu}}$ is the excitation rate from the ground state. Therefore, the ratio of the satellite line with the resonance is approximately

62 $$\begin{aligned} {I^{\mathrm{inner}}_{{ sf}} \over I_w} = {\beta N_{\mathrm{Li}{\text {-}}\mathrm{like}} \over N_{\mathrm{He}{\text {-}}\mathrm{like}}} {C^e_{{ is}} \over C^e_{{ gu}}} {A_{{ sf}} \over A^{\mathrm{tot}}_s} \end{aligned}$$

which is independent of electron density. We recall that the electron collisional excitation rate $C^e_{ij} \sim T_{\mathrm{e}}^{-1/2} {\varUpsilon _{i,j}(T_{\mathrm{e}}) \over \omega _i} \exp \left( {- \varDelta E_{i,j} \over kT_{\mathrm{e}}} \right)$, so the ratio does not depend much on electron temperature, because the excitation energies $\varDelta E_{i,j}$ of the two transitions are very similar, so also the exponential factors are very similar. In summary, the ratio of the satellite line with the resonance line depends directly on the relative population of the Li-like versus the He-like ion, aside from some factors which depend on the atomic data. In the original study by Gabriel (1972a), the actual collision strengths were approximated with effective oscillator strengths.

The above ratio is usually small, because the branching ratio $A_{{ sf}}/A^{\mathrm{tot}}_s$ is small, and because the $N_{\mathrm{Li}{\text {-}}\mathrm{like}}$ abundance is normally lower than $N_{\mathrm{He}{\text {-}}\mathrm{like}}$. Finally, we note that a correction factor to the resonance line intensity needs to be included. This factor should take into account the increase in the line intensity due to $n>3$ satellite lines formed by dielectronic recombination (see below). Once the ratio of the satellite line with the resonance is measured, the relative population of the Li-like versus the He-like ion can be obtained, and compared to the values predicted by assuming ionisation equilibrium. In this way, one can assess if the plasma is ionising or recombining.

### He-like satellite lines formed by dielectronic capture

As mentioned earlier, the dielectronic capture of a free electron colliding with the He-like ion can produce an autoionising state *s* of the Li-like ion, which can then produce a satellite line. The intensity of the satellite line, decay to the final level *f* is

63 $$\begin{aligned} I^{\mathrm{dr}}_{{ sf}} = N_s A_{{ sf}} \end{aligned}$$

where as before $A_{{ sf}}$ the transition probability by spontaneous emission from level *s* to the final level *f*, and $N_s$ is the population of the autoionising state, which is determined by the balance between the dielectronic capture (with rate $C^{\mathrm{dc}}$), autoionisation and radiative decay to all possible lower levels:

64 $$\begin{aligned} N_{\mathrm{He}{\text {-}}\mathrm{like}} N_{\mathrm{e}} C^{\mathrm{dc}} = N_s \left( \sum _k A^{\mathrm{auto}}_{sk} + \sum _{f<s} A_{{ sf}} \right) \end{aligned}$$

where we have seen that the sum of autoionising rates in this case reduces to a single term $A^{\mathrm{auto}}_s$, the total autoionisation rate into the He-like ground-level continua. The intensity of the satellite line can therefore be written as

65 $$\begin{aligned} I^{\mathrm{dr}}_{{ sf}} = N_{\mathrm{He}{\text {-}}\mathrm{like}} N_{\mathrm{e}} C^{\mathrm{dc}} {A_{{ sf}} \over A^{\mathrm{auto}}_s + \sum _{f<s} A_{{ sf}}}. \end{aligned}$$

We have seen in Sect. 3.5 that, by applying the Saha equation for thermodynamic equilibrium we obtain a direct relation between the rates of the two inverse processes of dielectronic capture and autoionisation (Eq. 41), which in our case is

66 $$\begin{aligned} C^{\mathrm{dc}} = {h^3 \over (2\pi \,m\,kT_{\mathrm{e}})^{3/2}} {\omega _s \over \omega _1} \exp \left( - {E_s - E_1 \over k T_{\mathrm{e}}} \right) A^{\mathrm{auto}}_{s} \end{aligned}$$

where $\omega _s, \omega _1$ are the statistical weights of the level *s* and the ground state of the He-like ion, and $E_s - E_1$ is the energy difference between the two states.

The intensity of the satellite line formed by dielectronic recombination can therefore be written as

67 $$\begin{aligned} I^{\mathrm{dr}}_{{ sf}} = 3.3\times 10^{-24} N_{\mathrm{He}{\text {-}}\mathrm{like}} N_{\mathrm{e}} {I_H \over (kT_{\mathrm{e}})^{3/2}} {\omega _s \over \omega _1} \exp \left( - {E_s - E_1 \over k T_{\mathrm{e}}} \right) {A_{{ sf}} A^{\mathrm{auto}}_s \over A^{\mathrm{auto}}_s + \sum _{f<s} A_{{ sf}}}\nonumber \\ \end{aligned}$$

where $I_H$ is the ionisation energy of neutral hydrogen (13.6 eV).

The ratio of the intensities of the satellite line formed by dielectronic recombination with the parent line is therefore

68 $$\begin{aligned} {I^{\mathrm{dr}}_{{ sf}} \over I_w} \simeq 3.3\times 10^{-24} {I_H \over (kT_{\mathrm{e}})^{3/2} C^e_{{ gu}}} {\omega _s \over \omega _1} \exp \left( - {E_s - E_1 \over kT_{\mathrm{e}}} \right) {A_{{ sf}} A^{\mathrm{auto}}_s \over A^{\mathrm{auto}}_s + \sum _{f<s} A_{{ sf}}}\qquad \end{aligned}$$

i.e., is independent of the population of the ions and the electron density. We recall the electron collisional excitation rate $C^e_{{ gu}}$ depends on the electron temperature, so in effect the above ratio depends only on $T_{\mathrm{e}}$, aside from the transition probabilities. In other words, the ratio is an excellent temperature diagnostic. It depends on $T_{\mathrm{e}}$ as

69 $$\begin{aligned} {I^{\mathrm{dr}}_{{ sf}} \over I_w} \sim T_{\mathrm{e}}^{-1/2} \exp \left( {E_0 - E_s + E_1 \over kT_{\mathrm{e}}} \right) \end{aligned}$$

where $E_0$ is the energy of the resonance transition. In normal coronal conditions, $E_0 - E_s + E_1 < kT_{\mathrm{e}}$ so the exponential factor is a slowly varying function of $T_{\mathrm{e}}$, so effectively the intensities of the satellite lines increase, with respect to the resonance line, when the electron temperature is lower. Indeed, observations show that during the gradual phase of solar flares, when the temperature decreases, the satellite lines become prominent.

When considering ions along the isoelectronic sequence, since $A^{\mathrm{auto}}_s$ does not depend much on *Z*, the main dependence with *Z* comes from the transition probability $A_{{ sf}}$, which scales as $Z^4$. Therefore, while the intensities of the satellite lines of say oxygen are weak, they become significant in heavier elements like iron. Indeed the best measurements of the satellite lines are from iron. The literature usually follows the notation from Gabriel (1972a), which is noted here in Table 3.

**Table 3:** List of the main He-like satellite lines, following the notation of Gabriel (1972a)

Key	Transition	
a	$1{s}^2\,2{p}\,{}^{2}\hbox {P}_{3/2}{-}1{s}\,2{p}^{2}\,{}^{2}\hbox {P}_{3/2}$	
b	$1{s}^2\,2{p}\,{}^{2}\hbox {P}_{1/2}{-}1{s}\,2{p}^{2}\,{}^{2}\hbox {P}_{3/2}$	
c	$1{s}^2\,2{p}\,{}^{2}\hbox {P}_{3/2}{-}1{s}\,2{p}^{2}\,{}^{2}\hbox {P}_{1/2}$	
d	$1{s}^2\,2{p}\,{}^{2}\hbox {P}_{1/2}{-}1{s}\,2{p}^{2}\,{}^{2}\hbox {P}_{1/2}$	
e	$1{s}^2\,2{p}\,{}^{2}\hbox {P}_{3/2}{-}1{s}\,2{p}^{2}\,{}^{4}\hbox {P}_{5/2}$	
f	$1{s}^2\,2{p}\,{}^{2}\hbox {P}_{3/2}{-}1{s}\,2{p}^{2}\,{}^{4}\hbox {P}_{3/2}$	
g	$1{s}^2\,2{p}\,{}^{2}\hbox {P}_{1/2}{-}1{s}\,2{p}^{2}\,{}^{4}\hbox {P}_{3/2}$	
h	$1{s}^2\,2{p}\,{}^{2}\hbox {P}_{3/2}{-}1{s}\,2{p}^{2}\,{}^{4}\hbox {P}_{1/2}$	
i	$1{s}^2\,2{p}\,{}^{2}\hbox {P}_{1/2}{-}1{s}\,2{p}^{2}\,{}^{4}\hbox {P}_{1/2}$	
j	$1{s}^2\,2{p}\,{}^{2}\hbox {P}_{3/2}{-}1{s}\,2{p}^{2}\,{}^{2}\hbox {D}_{5/2}$	
k	$1{s}^2\,2{p}\,{}^{2}\hbox {P}_{1/2}{-}1{s}\,2{p}^{2}\,{}^{2}\hbox {D}_{3/2}$	
l	$1{s}^2\,2{p}\,{}^{2}\hbox {P}_{3/2}{-}1{s}\,2{p}^{2}\,{}^{2}\hbox {D}_{3/2}$	
m	$1{s}^2\,2{p}\,{}^{2}\hbox {P}_{3/2}{-}1{s}\,2{p}^{2}\,{}^{2}\hbox {S}_{1/2}$	
n	$1{s}^2\,2{p}\,{}^{2}\hbox {P}_{1/2}{-}1{s}\,2{p}^{2}\,{}^{2}\hbox {S}_{1/2}$	
o	$1{s}^2\,2{p}\,{}^{2}\hbox {P}_{3/2}{-}1{s}\,2{s}^{2}\,{}^{2}\hbox {S}_{1/2}$	
p	$1{s}^2\,2{p}\,{}^{2}\hbox {P}_{1/2}{-}1{s}\,2{s}^{2}\,{}^{2}\hbox {S}_{1/2}$	
q	$1{s}^{2}\,2{s}\,{}^2\hbox {S}_{1/2}{-}1{s}\,2{p}\,2{s}\,(^1\hbox {P})\,{}^{2}\hbox {P}_{3/2}$	Inner-shell
r	$1{s}^{2}\,2{s}\,{}^2\hbox {S}_{1/2}{-}1{s}\,2{p}\,2{s}\,(^1\hbox {P})\,{}^{2}\hbox {P}_{1/2}$	Inner-shell
s	$1{s}^{2}\,2{s}\,{}^2\hbox {S}_{1/2}{-}1{s}\,2{p}\,2{s}\,(^3\hbox {P})\,{}^{2}\hbox {P}_{3/2}$	Inner-shell
t	$1{s}^{2}\,2{s}\,{}^2\hbox {S}_{1/2}{-}1{s}\,2{p}\,2{s}\,(^3\hbox {P})\,{}^{2}\hbox {P}_{1/2}$	Inner-shell
u	$1{s}^{2}\,2{s}\,{}^2\hbox {S}_{1/2}{-}1{s}\,2{p}\,2{s}\,{}^{4}\hbox {P}_{3/2}$	Inner-shell
v	$1{s}^{2}\,2{s}\,{}^2\hbox {S}_{1/2}{-}1{s}\,2{p}\,2{s}\,{}^4\hbox {P}_{1/2}$	Inner-shell

### Satellite lines of other sequences

The process of formation of satellite lines is a general one, and satellite lines are present also for ions of other sequences. We refer to the above-cited reviews for more details, but would like to mention two important sets of satellites. The most important ones are those of H-like ions.

**Fig. 20:**
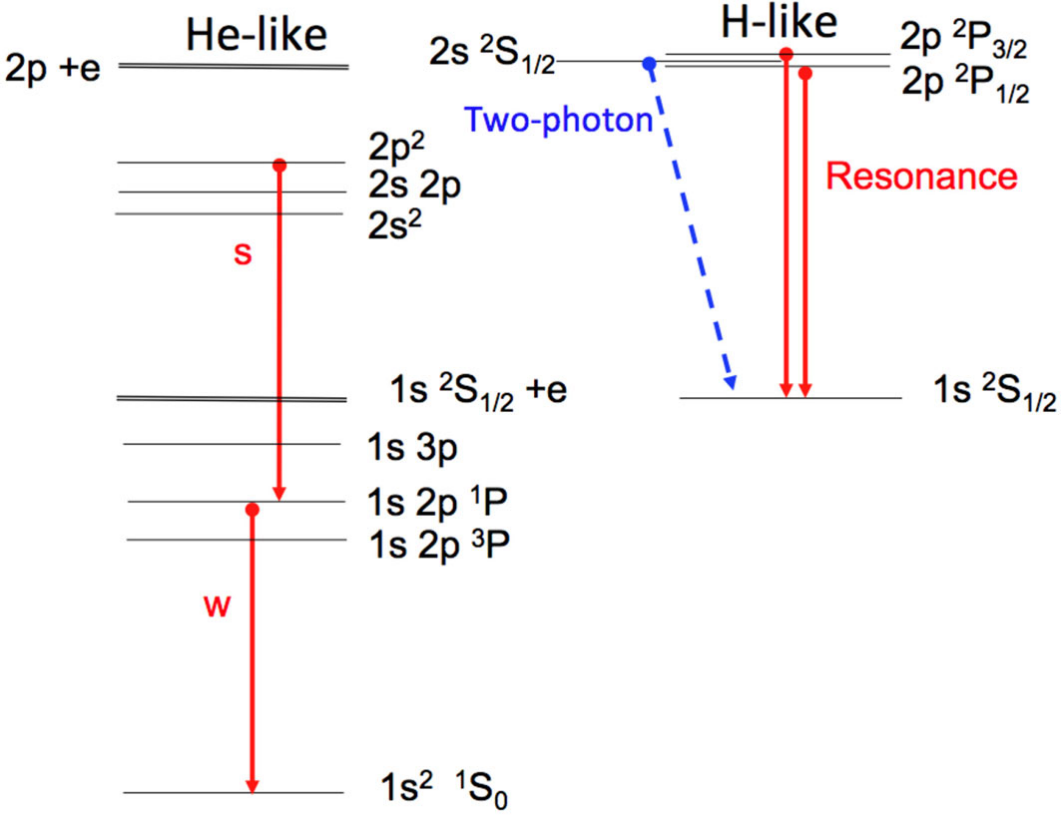
A sketch of the process of formation of satellite lines of the H-like $n=2$ resonance line

H-like satellite lines have some similarities and differences with the He-like ones. The main difference is that the only process of formation is dielectronic capture. The main satellites have, as in the He-like case, the $n=2$ parent line, the $\hbox {L-}\alpha$ doublet, the decay of the $2p\,^2\hbox {P}_{3/2,1/2}$ to the ground state (see Fig. 20).

The $2p\,{}^2\hbox {P}_{3/2,1/2}$ and $2s\,{}^2\hbox {S}_{1/2}$ are mainly collisionally excited from the ground state. The 2*s* $^2\hbox {S}_{1/2}$ decays mainly via a two-photon emission at low densities (even for solar flare conditions), and collisional transfer between the $2s\,{}^2\hbox {S}_{1/2}$ and $2p\,{}^2\hbox {P}$ is negligible. For a list of the main transitions and description of the calculations see, e.g., Boiko et al. (1977, 1978b).

Satellite lines in ions of other sequences are also important at some wavelengths. Most notably, the region between 1.85 and 1.9 Å (cf. Fig. 22) is reach in satellite lines from Fe xx, Fe xxi, Fe xxii, and Fe xxiii, as discussed e.g., in Doschek et al. (1981a), where a detailed list of transitions is provided. The most notable transition is the Fe xxiii inner-shell $\beta$ line, which can be used, in conjunction with the He-like Fe xxv, to assess for departures from ionisation equilibrium (Gabriel 1972b).

### Measuring departures from Maxwellian distributions

The fact that the satellite lines can only be excited by free electrons having specific energies means that, if one could measure the intensities of several satellites, information on the energy distribution of the free electrons could be obtained. A method to assess if non-Maxwellian distributions exist during solar flares was suggested by Gabriel and Phillips (1979). It relies on the measurement of the *j* and $n=3$ complex of satellites in the He-like Fe, of which the transition *d*13 is the main one.

**Fig. 21:**
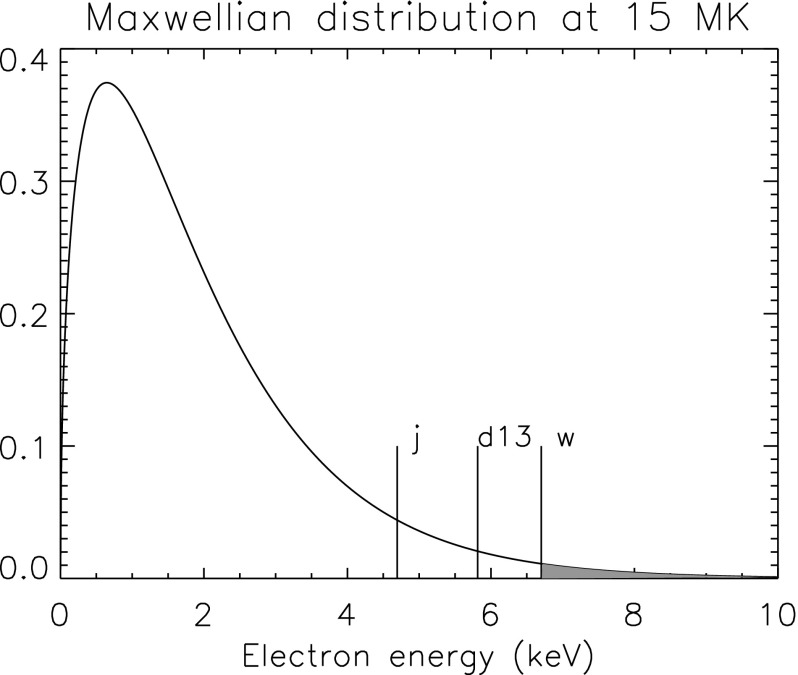
A Maxwellian distribution of electrons at 15 MK, with the energies of the satellite lines *j* and *d*13, and the threshold energy to excite the resonance line *w*

The *j* and $n=3$ complex are produced by electrons having two specific energies, shown in Fig. 21 with two vertical lines, superimposed on a Maxwellian distribution of a plasma with 15 MK, a temperature typical of moderately-large solar flares.

On the other hand, any electron with energy above the resonance *w* threshold can excite the line. Those energies are indicated with the grey area under the Maxwellian curve. If the electron distribution is Maxwellian, then the temperatures obtained from the observed *j* / *w* and *d*13 / *w* and the above prescriptions should be consistent, assuming that all the atomic data are accurate. On the other hand, if a tail of high-energy electrons is present (as it is known to be present for large flares from hard X-ray bremsstrahlung observations), then the resonance line would be greatly enhanced, as all the electrons above the threshold would increase the collisional population of the upper level of the resonance line. The temperatures obtained (with the Maxwellian assumption) from the observed *j* / *w* and *d*13 / *w* should therefore be different.

The method does not allow one to infer the complete electron distribution, but just to confirm if the distribution is Maxwellian or not. Several measurements of satellite lines that can only be excited at progressively higher energies would be needed to describe the electron distribution.

The d13 line complex has been resolved in laboratory plasmas, and the effects of non-thermal electrons on the spectra have been detected (see, e.g., Bartiromo et al. 1983, 1985; Lee et al. 1985). Satellite lines have also been observed in solar spectra (see next section), and some applications of the present diagnostics have been published. They are summarised later in Sect. 6, where we discuss non-equilibrium effects.

### Observations

Among the earliest observations, there are those reported by Neupert and Swartz (1970) and Neupert (1971a) obtained with the OSO-5, where the satellite lines from the $1s\,2s\,2p$ configuration in the He-like Si, S, Ar, and Fe ions were observed. Walker and Rugge (1971) observed several satellites of the H- and He-like Mg, Al, Si and S ions, form the OV1-17 satellite. Acton et al. (1972) observed the satellites of the He-like O and Ne using a rocket, while Parkinson (1971, 1972) and Parkinson et al. (1978) provided excellent spectra of He-like satellites of Ne, Mg and Si obtained with Skylark rockets and with OSO-8 observations of active regions and flares. Parkinson et al. (1978) also reported observations of the H-like Si xiv with OSO-8.

Doschek et al. (1971a, b) presented an analysis of the observations of the He-like Ca and Fe obtained with the NRL Bragg crystal spectrometer on-board OSO-6. Further earlier observations were those of the He-like Fe obtained by the Intercosmos-4 Satellite and the ‘Vertical-2’ Rocket (Grineva et al. 1973), and of the H-like lines by the same rocket and Intercosmos-4 and Intercosmos-7 (Aglitskii et al. 1978).

Later, several observations were obtained with the excellent NRL SOLFLEX instrument (cf. Doschek et al. 1980, 1981a; Feldman et al. 1980) and then from the crystals on-board the Hinotori, Yohkoh and SMM satellites.

**Fig. 22:**
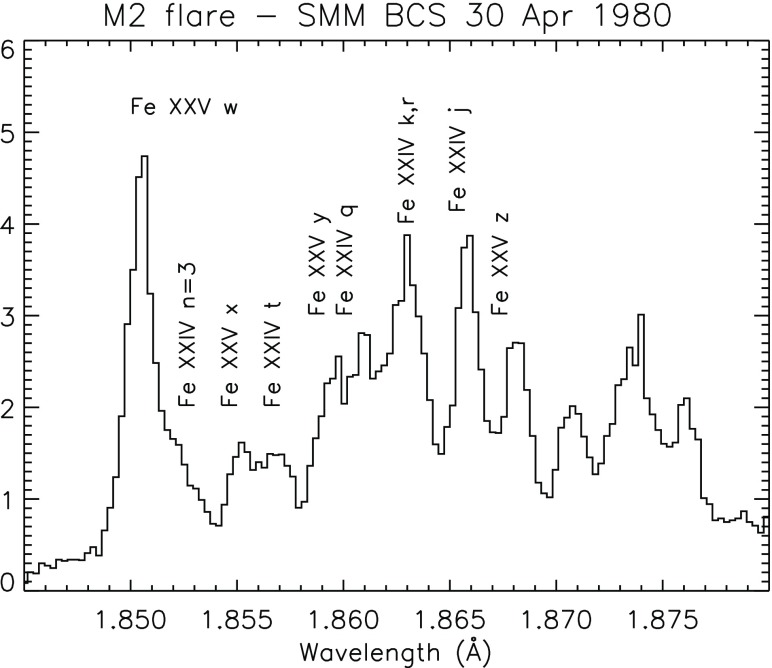
An SMM BCS spectrum during the peak phase of a flare, containing the He-like lines from iron and many satellites, some of which are labelled

**Fig. 23:**
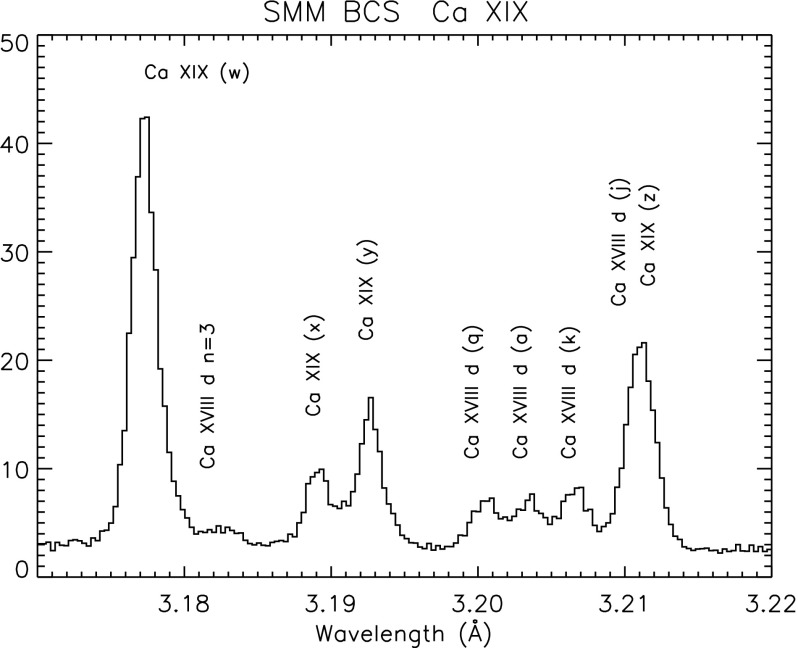
An SMM BCS spectrum during the peak phase of a flare, containing the Ca He-like lines and associated satellites

**Fig. 24:**
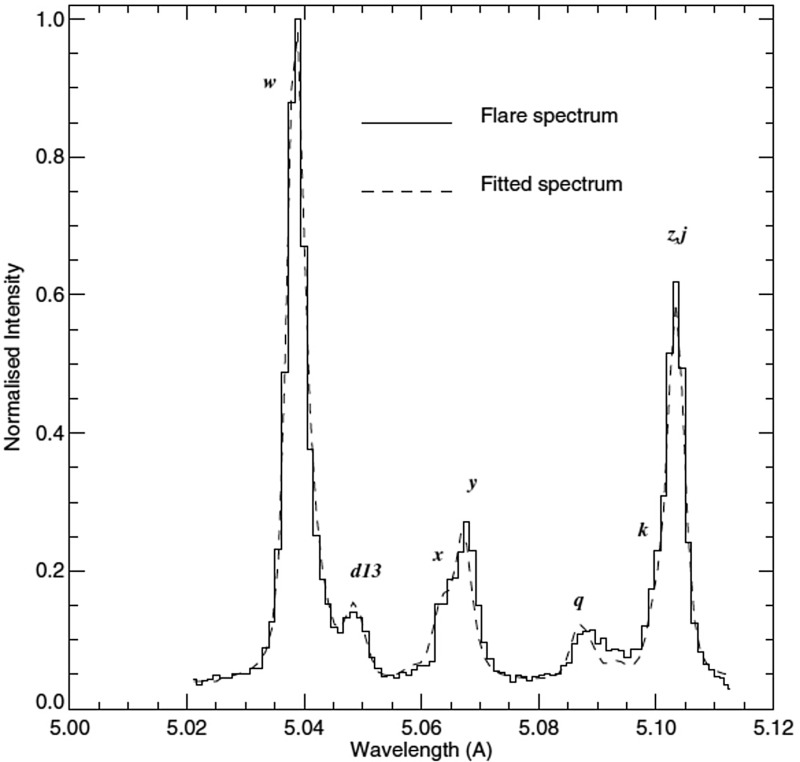
A Yohkoh BCS spectrum of the He-like lines from S and associated satellites. The dashed line is a theoretical spectrum Figure from Phillips et al. (2004)

Figure 22 shows as an example a spectrum obtained by SMM BCS, the He-like lines from iron, while Fig. 23 shows the corresponding spectrum of the He-like Ca lines. In addition, Fig. 24 shows a Yohkoh BCS spectrum of the sulphur He-like lines and associated satellites during a flare.

## Atomic calculations and data

This review is primarily concerned with diagnostics of electron densities and temperatures from spectral lines of the same ion. In this case, the primary atomic data are the cross-sections for electron-ion collisions and the radiative data (A-values), with their uncertainties. We therefore mainly review the latest work on these atomic data, and where references and data can be obtained, with emphasis on the CHIANTI database. We also introduce to the unfamiliar reader a few basic aspects about the relevant atomic calculations for such data. Finally, we also briefly review ionization and recombination data, line identification and benchmarking studies.

### The CHIANTI atomic package

The CHIANTI package consists of a critically evaluated set of atomic data for a large number of ions of astrophysical interest. It also includes a quantity of ancillary data and a suite of Interactive Data Language (IDL) programs to calculate optically thin synthetic spectra (including continuum emission) and to perform general spectral analysis and plasma diagnostics, from the X-rays to the infrared region of the spectrum.

The CHIANTI database started in 1995 as a collaboration between K. P. Dere (NRL, USA), H. E. Mason (University of Cambridge UK), and B. Monsignori Fossi (Arcetri Observatory, Italy). Sadly, the latter died prematurely in 1996. M. Landini (University of Florence, Italy) then worked with the CHIANTI team for several years. Currently, the project involves K. P. Dere and P. R. Young (George Mason University, USA), E. Landi (University of Michigan, USA), and G. Del Zanna and H. E. Mason (University of Cambridge, UK).

The database relies almost entirely on the availability of published atomic data. Each version release is accompanied by a publication that discusses the new atomic data and any other new features of CHIANTI. References to the original calculations, line identifications, wavelength measurements and benchmark work are provided in the published papers and throughout the database. It is very important to acknowledge the hard work of the atomic data providers.

The CHIANTI package is freely available at the web page: http://www.chiantidatabase.org/ and within SolarSoft, a programming and data analysis environment written in IDL for the solar physics community. There is also a Python interface to some of the CHIANTI routines (CHIANTIPy: http://chiantipy.sourceforge.net/).

The first version of the CHIANTI database was released in 1996 and is described in Dere et al. (1997). v.2 is described in Landi et al. (1999). In v.3 (Dere et al. 2001) the database was extended to wavelengths shorter than 50 Å, while v.4 (Young et al. 2003) included proton excitation data, photoexcitation, and new continuum routines. v.5 (Landi et al. 2006) included new datasets, while v.6 (Dere et al. 2009) included a complete database of ionization and recombination coefficients. v.7 (Landi et al. 2012) improved ions important for the EUV and the UV, while v.7.1 (Landi et al. 2013) included new DW atomic data for the soft X-ray lines and new identifications from Del Zanna (2012a). V.8 (Del Zanna et al. 2015b) included new atomic data for several isoelectronic sequences and for a few coronal ions from iron and nickel.

As with any atomic data package, CHIANTI has been developed to suit some specific applications in solar physics and astrophysics, although with time it has become more generally used and is often used as a reference atomic database rather than a modelling code. CHIANTI basic data are in fact included in many other atomic databases and modelling packages, for example:

- VAMDC (http://portal.vamdc.eu),
- CLOUDY (http://www.nublado.org),
- MOCASSIN (http://mocassin.nebulousresearch.org),
- XSTAR (http://heasarc.gsfc.nasa.gov/docs/software/xstar/xstar.html),
- ATOMDB (http://www.atomdb.org),
- PINTofALE (http://hea-www.harvard.edu/PINTofALE/),
- XSPEC (http://heasarc.gsfc.nasa.gov/docs/xanadu/xspec/index.html).

The main assumptions within the CHIANTI codes are that the plasma is optically thin and in thermal equilibrium. The plasma ionization is dominated by collisions (i.e., no photo-ionization is included). Tabulations of ion populations in equilibrium are provided, in the low-density limit.

Line emissivities are reliable only within given temperature and density ranges. The maximum densities for an ion model depend on which excitations/de-excitations are included. Normally, all those from the ground configuration and the main metastable levels are included, although for most ions the complete set of excitation rates has been included in v.8 (Del Zanna et al. 2015b). This allows the use of the CHIANTI data for higher densities. The electron collision strengths were, until v.8, interpolated over a fixed temperature grid in the scaled domain as formulated by Burgess and Tully (1992). However, this occasionally reduced the accuracy at lower temperatures, typical of photoionised plasma. The rates for the new ions in v.8 were normally released as published, without interpolation.

Electrons and protons have Maxwellian distribution functions. Indeed CHIANTI data include Maxwellian-averaged electron and proton collision strengths. However, it is possible to study the effects of particle distributions that are linear combinations of Maxwellians of different temperatures. An implementation of the CHIANTI programs that calculates line emissivities assuming $\kappa$ distributions has recently been made available (Dzifčáková et al. 2015), based on v.7.1.

Finally, various other databases of atomic data exist, e.g., ADAS (http://open.adas.ac.uk, primarily for fusion work) and ATOMDB (http://www.atomdb.org, primarily for high energies).

### Atomic structure calculations

We refer the reader to standard textbooks of atomic spectroscopy such as Condon and Shortley (1935), Grant (2006) and Landi Degl’Innocenti (2014), the various articles cited below, and the *Springer Handbook of Atomic and Molecular Physics* (Drake 2006), in particular Chapter 21 on atomic structure and multi-configuration Hartree–Fock theories by Charlotte Froese Fischer, and Chapter 22 by Ian Grant on relativistic atomic structure. For recent reviews of multiconfiguration methods for complex atoms see Bieroń et al. (2015) and Froese Fischer et al. (2016).

The atomic structure of an ion is obtained by solving the time-independent Schrödinger equation:

70 $$\begin{aligned} H\varPsi _i = E_i \varPsi _i \end{aligned}$$

where $\varPsi _i$ are the wavefunctions of the system, $E_i$ are the eigenvalues, and *H* is the Hamiltonian.

For lighter elements of astrophysical importance, the approach that is often adopted is to describe the system with a non-relativistic Hamiltonian, and then add relativistic corrections using nonrelativistic wavefunctions and the Breit–Pauli approximation. For heavier elements, a fully relativistic approach is usually needed. This involves solving the Dirac equation and taking into account the Breit interaction (see, e.g., Grant 2006).

The non-relativistic Hamiltonian describing an ion with *N* electrons and a central nucleus having charge number *Z* can be written in the form

71 $$\begin{aligned} {H} = \sum _{i=1}^N \left( {p_i^2 \over 2\,m} - {Z e^2 \over r_i} \right) + \sum ^{N-1}_{i < j} {e^2 \over r_{ij}}, \end{aligned}$$

where $\mathbf {r}_i$ is the position vector of the *i*th electron (relative to the nucleus), $\mathbf {p}_i$ is its momentum, and $r_{ij}$ is the distance between the *i* and *j* electrons: $r_{ij} = r_{ji} = \left| \mathbf {r}_i - \mathbf {r}_j \right|$. The terms in the Hamiltonian are the total kinetic and potential energy of the electrons in the field of the nucleus, and the repulsive Coulomb energy between the electrons. This equation neglects the spin of the electron.

It can be shown that this Hamiltonian commutes with the total angular momentum *L* and the total spin *S* of the electrons. Additionally, it commutes with the parity operator. This allows the usual description of the wavefunction in terms of the associated good quantum numbers LSp.

For complex highly-ionised atoms, it is common to assume that the electron repulsions are small perturbations relative to the much stronger nuclear central potential, and to simplify the equation by assuming that each electron experiences a central potential, caused by the electrostatic interaction with the nucleus, screened by the other electrons. In this way, the zero-order Hamiltonian is first solved:

72 $$\begin{aligned} {H}_0 = \sum _{i=1}^N \left( {p_i^2 \over 2\,m} + V_{\mathrm{c}}(r_i) \right) \end{aligned}$$

where $V_{\mathrm{c}}(r_i)$ is the potential energy of each electron in the central field. The first-order Hamiltonian

73 $$\begin{aligned} {H}_1 = \sum _{i=1}^N \left( -V_{\mathrm{c}}(r_i) -{Z e^2 \over r_i} \right) + \sum _{i<j} {e^2 \over r_{ij}}. \end{aligned}$$

is then considered as a perturbation. There are several approximate numerical methods to find a solution for the central potential and to evaluate $V_{\mathrm{c}}(r)$. For example, the Thomas–Fermi statistical method and the Hartree–Fock self-consistent method using the variational principle.

When configuration interaction (CI) is considered, one atomic state is described by a linear combination of eigenfunctions of different configurations (but with the same parity, as $H_1$ commutes with the parity operator). Configuration interaction becomes increasingly important for excited states whenever the difference in energy between the states becomes small. The Hamiltonian ${H}_1$ commutes with the operators $L^2$ and $S^2$, so the total *L* and *S* values are still good quantum numbers representing the state.

There are several relativistic corrections (see, e.g., Bethe and Salpeter 1957), some of which arise directly from the solution of the Dirac equation. They are normally grouped into two classes, one-body and two-body operators (see, e.g., Badnell 1997). The non-fine-structure operators commute with the operators associated with the total angular momentum *L* and the total spin *S* of the electrons (plus their azimuthal components) so they do not break the *LS* coupling and are often included as an additional term in the Hamiltonian $H_0$. On the other hand, the fine-structure operators only commute with the operators associated with the total angular momentum *J* and its azimuthal component. The nuclear spin–orbit interaction, i.e., the interaction between the orbital angular momentum in the field of the nucleus and the intrinsic spin of the electron, is the operator causing the main splitting of the *J*-resolved levels. Normally, atomic structure calculations have to be carried out in intermediate coupling, where the atomic states, characterized by the quantum numbers *J* and *M* are expressed as linear combinations of the form (in Dirac notation)

74 $$\begin{aligned} |\alpha J M \rangle = \sum _{{ LS}} \mathcal{C}_{{ LS}}\,|\alpha L S J M \rangle , \end{aligned}$$

where the sum is extended to all the *L* and *S* values that are compatible with the configuration $\alpha$ and with the value of *J*. The $\mathcal{C}_{{ LS}}$ are the coefficients of the expansion, obtained by diagonalising the Hamiltonian of the perturbations.

Once the Schrödinger equation is solved and the eigenfunctions and eigenvalues are calculated, it is relatively straightforward to calculate the radiative data such as line strengths and A values for the different types of transitions.

The first requirement in any structure calculation is a good representation of the target, i.e., accurate wavefunctions for the target ion. Such representation requires the inclusion of configuration interaction (CI) with a large set of configurations (see, e.g., Layzer 1954).

Often, significant discrepancies between the experimental energies and those calculated ab-initio have been found. Various semi-empirical corrections based on the observed energies have therefore often been applied to the calculations. This improves the oscillator strengths. One example is the term energy correction (TEC) to the Hamiltonian matrix, introduced by Zeippen et al. (1977) and Nussbaumer and Storey (1978) within the superstructure program to improve the description of the spin–orbit mixing between two levels, which requires their initial term separation to be accurate. Semi-empirical corrections are often applied within the other atomic structure programs, see e.g., B. C. Fawcett work using the Cowan’s Hartree–Fock code (see the Fawcett 1990 review).

Traditionally, there have been two sets of atomic structure calculations that have been carried out in the literature. The first one is to provide the wavefunctions to be used for the scattering calculation. For such calculations, the emphasis is to include in the CC expansion all the levels that are deemed important (e.g., by cascading or by adding resonances in the excitation cross sections) for the spectroscopically-relevant levels.

The second set of calculations normally include a large set of CI and are focused on obtaining the most accurate energies and A-values for the lower levels in an ion, especially for the ground configuration and in general for the metastable levels. In fact, such sets of A-values establish the population of the lower levels, so it is important to obtain accurate values.

#### Codes

Over the years, many codes have been developed to calculate the atomic structure and associated radiative data. Among the most widely used earlier codes are Cowan’s Hartree–Fock (Cowan 1967, 1981), the superstructure program (Eissner et al. 1974), the CIV3 program (Hibbert 1975), and the HULLAC code (Bar-Shalom et al. 1988).

Autostructure (Badnell 2011), originally a development of the superstructure program, has now become a multi-purpose program for the calculations of a wide range of atomic processes. The code and relative information are available at http://www.apap-network.org/codes.html. Ian Grant’s general-purpose relativistic atomic structure program (GRASP) (Grant et al. 1980; Dyall et al. 1989), based on multiconfiguration Dirac–Hartree–Fock theory, was significantly modified and extended (GRASP2K, see Jönsson et al. 2007, 2013c). Among other features, the new version implements a bi-orthogonal transformation method that allows initial and final states in a transition array to be optimized separately. This often leads to an improvement in the accuracy of the rates. The multi-configuration Hartree–Fock (MCHF) (Froese Fischer 1991) code has also been improved and modified over the years. The most up-to-date version is called ATSP2K. As in GRASP2K, to calculate transition probabilities the orbitals of the initial and final state do not need to be orthogonal. The international collaboration on computational atomic structure (COMPAS) has a useful description of various codes (especially GRASP2K and ATSP2K) and their applications. The codes can be downloaded from their website: ddwap.mah.se/tsjoek/compas/software.php. The Flexible Atomic Code (FAC) (Gu 2004) is available at GitHub: https://github.com/fnevgeny/fac/.

### Uncertainties in atomic structure calculations

The accuracy of a particular atomic structure calculation depends on several factors. One important factor is the representation which is used for the target wavefunctions. The target must take into account configuration interaction and allow for intermediate coupling for the higher stages of ionization (cf. Mason 1975a).

It is customary to assess the accuracy of the target wavefunctions by comparing the level energies with the experimental ones. This is a good zero-order approach, although there are cases when, despite relatively good agreement between theoretical and experimental energies, significant problems are still present.

In the literature, the studies which focus on the atomic structure of an atom normally perform a series of calculations, where the size of the CI (that is the number of configurations) is increased until some convergence is achieved for the (lower) levels of spectroscopic importance. It is also common to study the effects of which electrons are allowed to be promoted/excited. One recent example is given by Gustafsson et al. (2017a), where the MCDHF method, as implemented in the GRASP2K program, was used to study correlation effects for Mg-like iron.

**Fig. 25:**
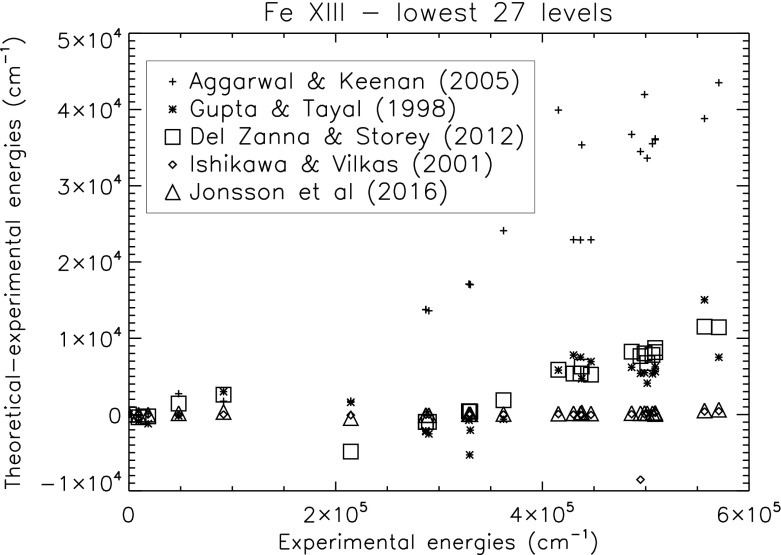
A comparison between experimental and ab-initio energies for the lowest 27 levels ($3{s}^2\,3{p}^2, 3{s}\,3{p}^3, 3{s}^2\,3{p}\,3{d}$) in Si-like Fe xiii with different target wavefunctions (see text)

As an example of how different calculated energies can be, we consider here the ab-initio energies for the lowest 27 levels ($3{s}^2\,3{p}^2, 3{s}\,3{p}^3, 3{s}^2\,3{p}\,3{d}$) as calculated by different authors with different codes in recent years for Si-like iron (Fe xiii). These are plotted in Fig. 25, relative to the experimental energies (all known) as available in CHIANTI v.8. Gupta and Tayal (1998) used the CIV3 computer code and considered for the CI expansions one- and two-electron excitations. Aggarwal and Keenan (2005) used the GRASP code to calculate the ab-initio energies for the lowest 97 levels. Del Zanna and Storey (2012) used the autostructure code and the Thomas–Fermi–Amaldi central potential, with scaling parameters, including in the CI expansion all the configurations up to $n=4$, giving rise to 2186 fine-structure levels. Vilkas and Ishikawa developed a multiconfiguration Dirac–Fock–Breit self-consistent field plus state-specific multireference Møller–Plesset (MR–MP) perturbation procedure to obtain very accurate level energies for open-shell systems. They applied this procedure to Si-like ions as described in Vilkas and Ishikawa (2004). Recently, Jönsson et al. (2016) applied the GRASP2K code to carry out large-scale calculations for Si-like ions, including correlations up to $n=8$. It is clear that the GRASP2K and MR–MP calculations are outperforming the other codes, achieving spectroscopic accuracy, i.e., deviations from the experimental energies of a few hundred Kaysers. However we should note that the other calculations were mostly concerned in defining the wavefunctions for the scattering calculations. For this relatively simple ion, the autostructure energies are close to those from CIV3, while larger deviations are clear for the GRASP calculations. In turn, such deviations often affect the oscillator strengths to the levels, and as a consequence the cross-sections for electron excitations, as pointed out by Del Zanna (2011a) for this particular ion, and also as discussed below.

**Fig. 26:**
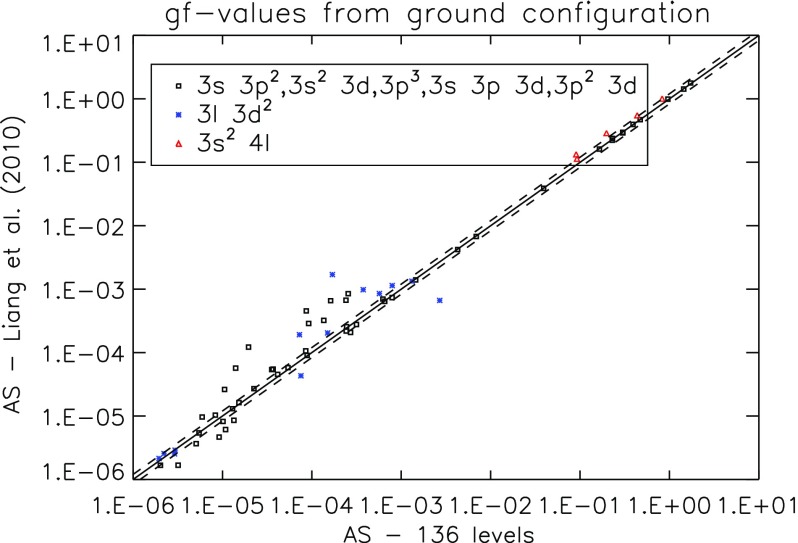
Oscillator strengths (*gf* values) for all transitions from the ground configuration of Fe xiv as calculated with a smaller CI (136 levels) versus those calculated with a much larger CI (2985 levels) by Liang et al. (2010). The dashed lines indicate $\pm \,20\%$ Image adapted from Del Zanna et al. (2015a)

Another way to assess the accuracy of the target wavefunctions is to compare oscillator strengths (*gf* values) for the dipole-allowed transitions as computed with different sets of configurations or parameters. Similarly, A-values for the forbidden transitions are usually compared. Typically, the *gf* values for transitions to lower levels do not vary significantly, while it is always the transitions to the highest levels in the calculations that are more uncertain. They are usually also the weakest. Figure 26 shows as an example the *gf* values for transitions from the ground configuration of Fe xiv as calculated with a set of configurations giving rise to 136 fine-structure levels (Del Zanna et al. 2015a), and a much larger CI (2985 levels) as calculated by Liang et al. (2010). There are some clear discrepancies for transitions to the higher levels, despite the fact that the same code (autostructure) and approximations were used.

Generally, larger CI calculations lead to more accurate oscillator strengths. However, for complex ions (e.g., the coronal iron ions) a large calculation does not necessarily provide accurate values. For example, the spin–orbit mixing among fine-structure levels of the same J and parity is very sensitive to the difference between energy level values, and small variations can lead to large differences in oscillator strengths. This can even occur for some of the strongest transitions to low-lying levels, as shown, e.g., for Fe xi (Del Zanna et al. 2010b) and Fe viii (Del Zanna and Badnell 2014).

**Fig. 27:**
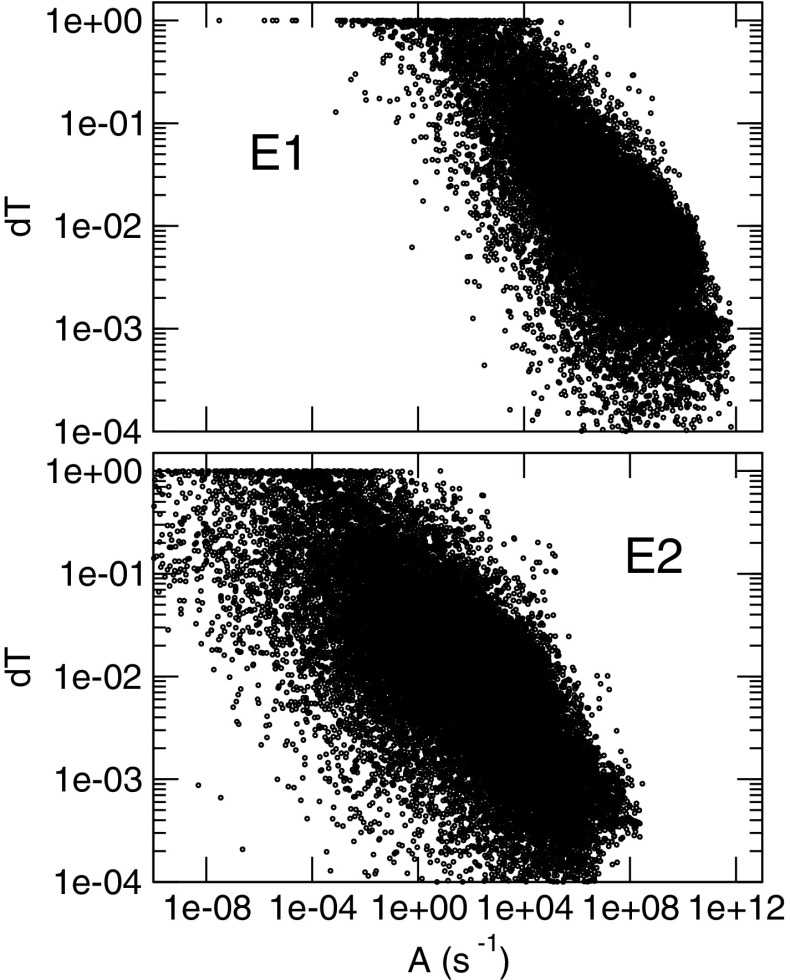
Scatter plots of accuracy estimates ${ dT}$ versus A-value for E1 and E2 transitions in Fe xiv Image reproduced with permission from Ekman et al. (2018), copyright by Elsevier

Any difference in the *gf* values is directly reflected in differences in the A values, which directly affect the line intensity. One way to assess the accuracy in the oscillator strengths (or A values) is to compare the values as computed in the Babushkin (length) and the Coulomb (velocity) gauges. A recent example is from the Fe xiv GRASP2K calculations by Ekman et al. (2018), where the accuracy was estimated from

75 $$\begin{aligned} { dT} = \frac{|A_{B}-A_{C}|}{\max (A_{B},A_{C})}, \end{aligned}$$

where $A_{B}$ and $A_{C}$ are the transition rates obtained in the Babushkin and Coulomb gauge, respectively (Fig. 27).

**Fig. 28:**
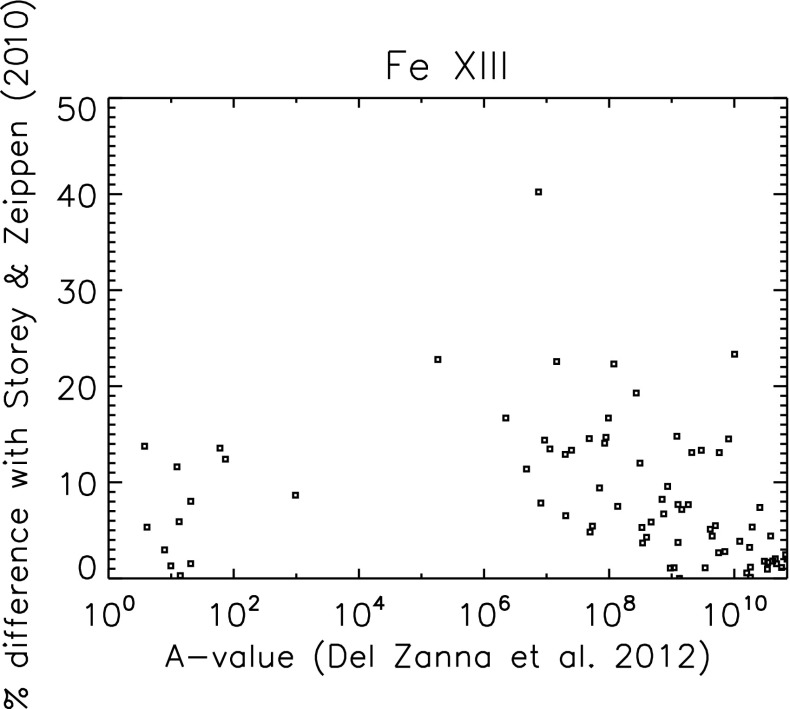
Percentage difference in the A values of Fe xiii transitions within the energetically lowest 27 levels, as obtained with two different calculations

One other way to assess the accuracy of the A-values is to compare the results of different calculations, where e.g., the size of the CI is different, but the same methods and codes are used. Figure 28 shows as an example the percentage difference in the A values of Fe xiii transitions within the energetically lowest 27 levels, as obtained by Storey and Zeippen (2010) and Del Zanna and Storey (2012). In both cases, the AUTOSTRUCTURE code was used. In Storey and Zeippen (2010), 114 fine-structure levels within the $n=3$ complex were included in the CI expansion. In Del Zanna and Storey (2012), 2985 $n=3,4$ levels were included. Clearly, very good agreement within 5–10% is present for the strongest transitions. A similar agreement is found with the A-values calculated by Young (2004) with SUPERSTRUCTURE.

### Measurements of level lifetimes

Laboratory measurements of radiative lifetimes of atomic levels have been very important in order to assess the accuracy of the theoretical radiative rates. Even with large computations, it is often found that theoretical values disagree significantly with the observed ones, especially for forbidden and intersystem transitions.

Many results have been obtained using beam-foil spectroscopy. Indeed there is a large body of literature on beam-foil measurements of level lifetimes. For recent reviews see, e.g., Träbert (2005, 2008). Beam-foil spectroscopy is based on a production of beams of fast ions that target a thin foil, where they are excited by the collisions with the electrons that are present in the foil (at the solid density). As a result, the ions reach a status where high-lying levels (with large values of the quantum numbers *n*, *l*, *J*) are multiply excited (i.e., several electrons are excited). The experimental setup is such that the ions travel through the thin foil and continue to travel in a low-density environment. During this travel, the various excited levels decay to the ground state in a complex way. The allowed transitions quickly depopulate most of the levels, while the metastable levels last much longer. In the mean time, the high-lying levels tend to decay via $\varDelta n=1$ transitions, and maintain the populations of the low-lying levels for a while via cascading. The beam-foil technique consists in measuring the transitions at varying distance from the foil. The decay curves provide measurements of the lifetimes of the levels, while the spectra at larger distances have been extremely useful to identify the transitions from metastable levels.

**Fig. 29:**
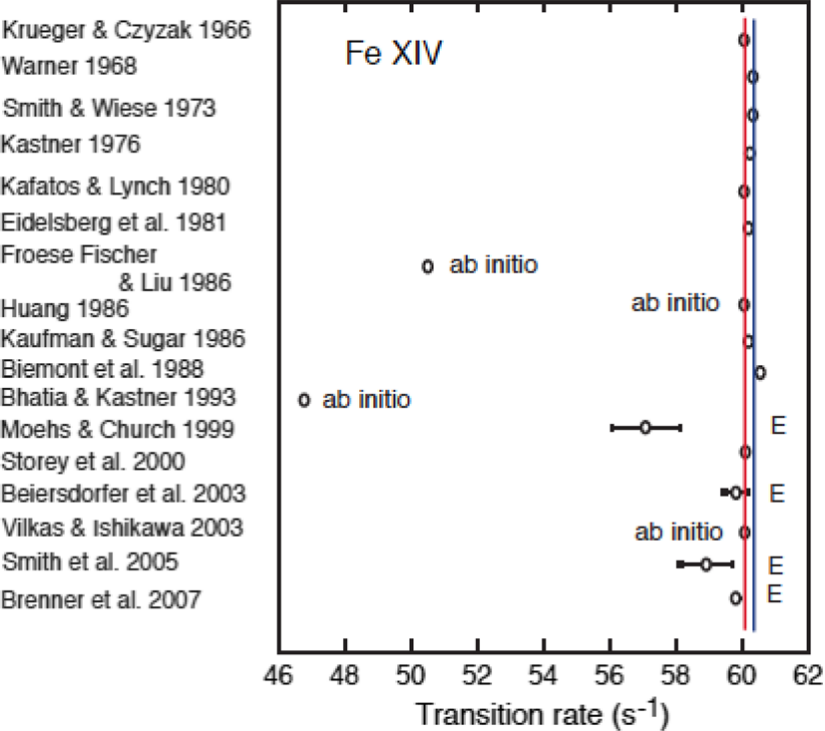
A comparison of theoretical and experimental A-value measurements for the green coronal line in Fe xiv. The red line indicates the value obtained from ab-initio calculations, adjusted to the experimental transition energy. The blue line indicates the value obtained by additionally taking into account the anomalous magnetic moment of the electron Image reproduced with permission from Träbert (2014a)

From the lifetimes of the levels, direct measurements of the A-values are sometimes possible. There is an extensive literature on theoretical and experimental lifetime measurements for important metastable levels, mostly in the ground configurations of abundant ions. Such levels typically have large quantum numbers *J*. Figure 29 shows as an example the results for the A-value measurements for the famous *green coronal line* in Fe xiv, obtained directly from the lifetime of the first excited level within the ground configuration of this ion. Theoretical values are also plotted in the figure. We can see good agreement between most calculations and experimental data, although a scatter in the values is present. Note that Brenner et al. (2007) used the Heidelberg EBIT to obtain a lifetime of 16.73 ms (equivalent to an A-value of $59.8\,\hbox {s}^{-1}$) with a very small uncertainty, which would indicate disagreement with theory (blue line, taking into account the experimental transition energy and the anomalous magnetic moment of the electron). However, this small uncertainty has been questioned (Träbert 2014b).

Quite often, however, levels decay via multiple branching pathways, so in the literature it is common to compare level lifetimes as calculated by theory (taking into account the branching ratios) and by experiment. This offers an excellent way to benchmark/validate the atomic structure calculations. For general reviews of how atomic structure calculations can be assessed by, e.g., lifetime measurements see, e.g., Träbert (2010, 2014a). Träbert (2005) reviewed lifetime measurements for the important iron ions. Table 4 shows as an example the lifetime measurements and predictions for Fe xii, as described in Träbert et al. (2008).

**Table 4:** Fe xii level lifetimes in the millisecond range and known or predicted principal decay channels (M1, E2, or M2) Adapted from Träbert et al. (2008)

Upper level	$\lambda \,(\hbox {nm})^{\mathrm{a}}$	Lifetime $\tau$ (ms)	
		Experiment	Theory
$3s^{2}\,3p^{3}\,{}^{2}\hbox {D}^{\mathrm{o}}_{3/2}$	240.641	$20.35 \pm 1.24^{\mathrm{b}}$	$18.9^{\mathrm{e}}, 18.9^{\mathrm{f}}, 5.0^{\mathrm{g}}, 16.8^{\mathrm{h}}$, $16.0^{\mathrm{i}}, 20.8^{\mathrm{j}}, 18.4^{\mathrm{k}}, 22.57^{\mathrm{b}}$, $18.0/18.0^{\mathrm{l}}, 17.7^{\mathrm{a}}$
		$$18.0 \pm 0.1^{\mathrm{c}}$$	
$3s^{2}3p^{3}\,{}^{2}\hbox {D}^{\mathrm{o}}_{5/2}$	216.976	$306 \pm 10^{\mathrm{c}}$	$324^{\mathrm{e}}, 115^{\mathrm{f}}, 326^{\mathrm{g}}, 294^{\mathrm{h}}$, $313^{\mathrm{i}}, 544^{\mathrm{j}}, 314^{\mathrm{k}}, 323/323^{\mathrm{l}}$, $311^{\mathrm{a}}$
$3s^{2}\,3p^{3}\,{}^{2}\hbox {P}^{\mathrm{o}}_{1/2}$	307.206, 356.6	$4.38 \pm 0.42^{\mathrm{b}}$	$3.84^{\mathrm{e}}, 3.84^{\mathrm{f}}, 3.84^{\mathrm{g}}$, $3.64^{\mathrm{h}}, 3.58^{\mathrm{i}}, 4.05^{\mathrm{j}}$, $3.81^{\mathrm{k}}, 3.59/3.79^{\mathrm{l}}, 3.8^{\mathrm{a}}$
		$$4.10 \pm 0.12^{\mathrm{c}}$$	
$3s^{2}\,3p^{3}\,{}^{2}\hbox {P}^{\mathrm{o}}_{3/2}$	256.677, 290.470	$1.85 \pm 0.24^{\mathrm{b}}$	$1.61^{\mathrm{e}}, 1.61^{\mathrm{f}}, 2.39^{\mathrm{g}}$, $1.55^{\mathrm{h}}, 1.53^{\mathrm{i}}, 1.67^{\mathrm{j}}$, $1.59^{\mathrm{k}}, 1.59/1.59^{\mathrm{l}},1.6^{\mathrm{a}}$
		$$1.70 \pm 0.08^{\mathrm{c}}$$	
$3s^{2}\,3p^{2}\,(^{3}\hbox {P})\,3d\,^{4}\hbox {F}_{9/2}$	25.187, 59.2600, 1421.868	$11 \pm 1^{\mathrm{d}}$	$11.6/9.2^{\mathrm{l}}, 9.7^{\mathrm{a}}, 9.56^{\mathrm{m}}$
$3s^{2}\,3p^{2}\,(^{1}\hbox {D})\,3d\,^{2}\hbox {G}_{9/2}$	184.723, 163.484, 64.292	$4 \pm 1^{\mathrm{d}}$	$4.00/4.27^{\mathrm{l}}, 4.03^{\mathrm{a}}, 4.43^{\mathrm{m}}$
	22.167		

$^{\mathrm{a}}$Del Zanna and Mason (2005a), $^{\mathrm{b}}$Moehs et al. (2001), $^{\mathrm{c}}$Träbert et al. (2002), $^{\mathrm{d}}$Träbert et al. (2008), $^{\mathrm{e}}$Garstang (1972), $^{\mathrm{f}}$Smith and Wiese (1973), $^{\mathrm{g}}$Smitt et al. (1976), $^{\mathrm{h}}$Mendoza and Zeippen (1982), $^{\mathrm{i}}$Huang (1984), $^{\mathrm{j}}$Kaufman and Sugar (1986), $^{\mathrm{k}}$Biémont and Hansen (1985), $^{\mathrm{l}}$Biémont et al. (2002) (two approximations), $^{\mathrm{m}}$Froese Fischer et al. (2006)

**Fig. 30:**
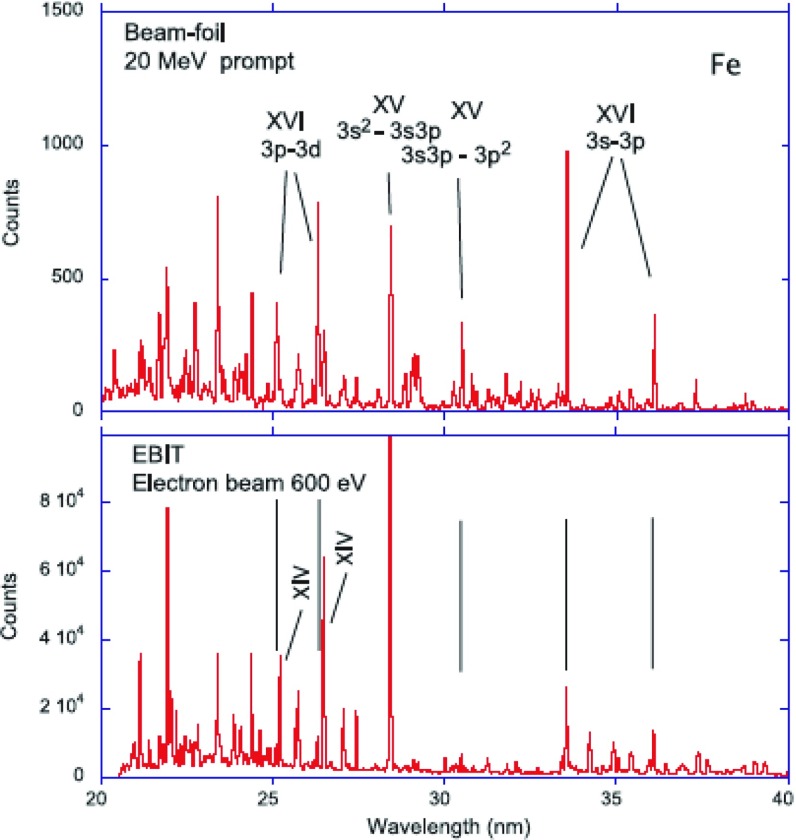
A beam-foil spectrum of iron ions (top), compared to an EBIT spectrum Image reproduced with permission from Träbert (2017), copyright by CSP

The ion beam energy determines the average charge state reached. Therefore, by tuning the energy, it is possible to obtain spectra where particular charge state ions are enhanced. Beam-foil spectroscopy has therefore been fundamental for the identification of spectral lines, in particular those arising from levels with long lifetimes. However, the spectra always contain a mixture of charge states. A sample spectrum is shown in Fig. 30.

Many other methods and devices have been used to measure atomic lifetimes, mostly with heavy-ion storage rings and electron beam ion trap (EBIT) devices. EBIT devices are extremely useful for assessing solar spectra because the plasma density is normally in the range $10^{11}{-}10^{13}\,\hbox {cm}^{-3}$, i.e., not far from typical densities found in active regions and flares. The beam energy can be adjusted so only a range of charge states from an element (all those below the threshold for ionization of the highest charge) are observed. This aids the identification of the spectral lines. Figure 30 (below) shows a spectrum obtained with an electron beam ion trap (EBIT), as an example.

For a review of measured lifetimes for iron ions using heavy-ion storage rings see Träbert et al. (2003), while Träbert (2002) also discusses EBIT measurements.

### Magnetically-induced transitions

We note here a relatively new and potentially interesting diagnostic to measure solar magnetic fields using XUV lines. A few cases have been discovered where transitions which are strictly forbidden are actually observed in laboratory plasma, being induced by very strong magnetic fields. They are called magnetically-induced transitions (MIT). For sufficiently strong magnetic fields and atomic states which are nearly degenerate and have the same magnetic quantum number and parity, a strong external field could break the atomic symmetry (by mixing the atomic states), and induce new magnetically-induced transitions.

**Fig. 31:**
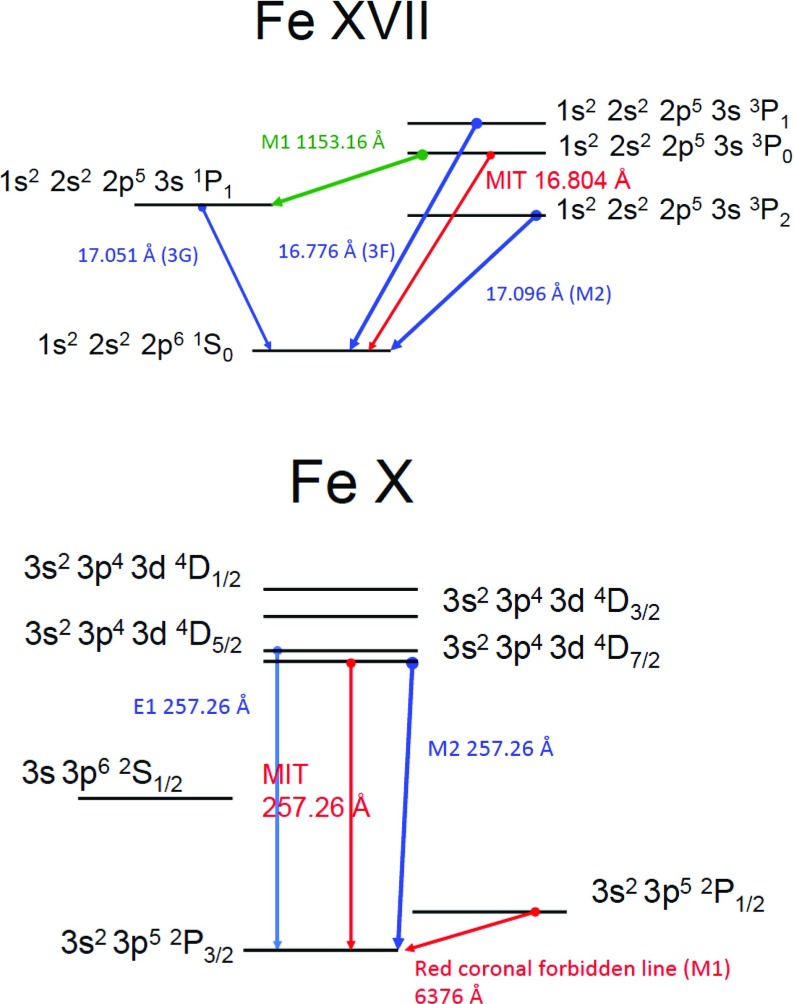
Grotrian diagrams (not to scale) of the lowest levels in Fe xvii and Fe x, indicating the levels involved in the magnetically-induced transitions

Beiersdorfer et al. (2003) reported an observation of an X-ray MIT transition at 47 Å in Ne-like Ar by an EBIT with strong magnetic fields of the order of 30 kG. Li et al. (2013) reported calculations of the strength of MIT along the Ne-like sequence. The same transition in Ne-like iron (Fe xvii) originates from the metastable $2{s}^{2}2{p}^{5}\,{}^3\hbox {P}_{0}$ level (hereafter metastable $J=0$ level), which normally decays with a well-known M1 forbidden transition (at 1153.16 Å) to the lower $^1\hbox {P}_{1}$ level, see Fig. 31 (top). The decay to the $J=0$ ground state is strictly forbidden, however the MIT line at 16.804 Å was recently measured by Beiersdorfer et al. (2016) using the Livermore EBIT in the presence of a strong magnetic field, allowing them to provide an estimate of the lifetime of the metastable $J=0$ level in Fe xvii. This MIT transition is very close to one of the strong X-ray lines from this ion, at 16.776 Å, usually labelled with (3F). Figure 31 (top) also shows two other main X-ray lines (see Del Zanna and Ishikawa 2009 for a discussion on line identifications and wavelength measurements for this ion).

Another example of a MIT occurs in Cl-like iron. The Fe x spectrum was discussed in detail in Del Zanna et al. (2004a). This ion gives rise to famous red coronal line, within the $3{s}^2\,3{p}^5\,{}^2\hbox {P}_{3/2, 1/2}$ ground configuration states, and several important forbidden transitions in the EUV and visible which ultimately decay into the $3{p}^{4}\,3{d}\,{}^4\hbox {D}_{5/2, 7/2}$ levels (see Fig. 31, bottom). The $3{p}^{4}\,3{d}\,{}^4\hbox {D}_{7/2}$ level is a metastable, and can only decay to the ground state via a M2 transition. This line is the fourth strongest line in the EUV spectrum of Fe x. The $^4\hbox {D}_{5/2}$ level decays via an E1 transition to the ground state. This transition is relatively strong, but much weaker than the M2. Smitt (1977) was the first to tentatively assign an energy (of $388{,}708\,\hbox {cm}^{-1}$) to both $3{p}^{4}\,3{d}\,{}^4\hbox {D}_{5/2, 7/2}$ levels, which were then confirmed in Del Zanna et al. (2004a). The Hinode EIS measurements however showed a discrepancy of about a factor of 2 between predicted and observed intensities of these lines (Del Zanna 2012b), and it was only with the latest large-scale scattering calculation (Del Zanna et al. 2012a) that agreement has been found.

The MIT in Fe x was discussed in detail by Li et al. (2015). In the presence of a strong magnetic field, the two $3{p}^{4}\,3{d}\,{}^4\hbox {D}_{5/2, 7/2}$ states will mix, which will produce a new MIT (E1) from the $^4\hbox {D}_{7/2}$ level to the ground state $3{s}^2\,3{p}^5\,{}^2\hbox {P}_{3/2}$, see Fig. 31 (bottom). Li et al. (2015) carried out increasingly large atomic structure calculations, which were used to estimate the splitting of the $3{p}^{4}\,3{d}\,{}^{4}\hbox {D}_{5/2}$ and $3{p}^{4}\,3{d}\,{}^{4}\hbox {D}_{7/2}$ levels, which turns out to be a major uncertainty in the diagnostic. The best calculations provided a splitting of $20\,\hbox {cm}^{-1}$. The authors did not take into account the review by Del Zanna et al. (2004a) of previous experimental data, where a much smaller energy difference of $5\,\hbox {cm}^{-1}$ was suggested. The assessment was partly based on previous work, which included Skylab observations. Judge et al. (2016) carried out a similar analysis of the same Skylab observations to obtain $3.6 \pm 2.7 \hbox { cm}^{-1}$. A similar energy difference was estimated from recent EBIT measurements (Li et al. 2016), although a more accurate measurement would be useful. It remains to be seen if magnetic fields can be measured with Hinode EIS, given the various uncertainties: aside from the uncertainty in the energy splitting, and the difficulty in obtaining accurate atomic data for these levels, the transitions from the $^4\hbox {D}$ are significantly affected by variations in the electron temperature and density. They are also sensitive to non-Maxwellian electron distributions (Dudík et al. 2014b). Finally, a significant uncertainty is associated with the Hinode EIS calibration.

Finally, we point out that in principle the MIT are a general tool available for other states/sequences. For example, Grumer et al. (2013) presented calculations for the $2s\,2p\,{}^3\hbox {P}_{0} \rightarrow 2{s}^2\,{}^1\hbox {S}_{0}$ MIT transition in Be-like ions. while Grumer et al. (2014) and Johnson (2011) also discuss the effects that are present in ions/isotopes with non-zero nuclear spin, which cause hyperfine structure and hyperfine-induced E1 transitions.

### Atomic structure data

Excellent sets of A-values have been obtained with semi-empirical corrections and the superstructure and CIV3 programs. The MCHF have also been very accurate for the ground configurations, and there are several studies by C. Froese Fischer and collaborators. An on-line database of MCHF calculations is available at http://nlte.nist.gov/MCHF/. Very accurate ab initio multi-reference Møller–Plesset calculations have been carried out for a limited set of ions and configurations by Y. Ishikawa and collaborators. For example, see Ishikawa and Vilkas (2001) for the Si-like ions, and Ishikawa and Vilkas (2008) for S-like ions.

The new development of the GRASP program, GRASP2K (Jönsson et al. 2007), allows large-scale calculations to be performed even for the most complex ions. Such calculations reach spectroscopic accuracy, in the sense that theoretical wavelengths in the EUV can be within a fraction of an Å of the observed values. Previous ab-initio calculations typically had an accuracy of a few Å, which is often not sufficient to enable the line identification process (see below). A recent review of GRASP2K calculations can be found in Jönsson et al. (2017). As a measure of the accuracy, one could look at the differences between the length and velocity forms of the oscillator strengths, or at the differences in the A-values as calculated by different codes. Typical uncertainties of the order of a few percent are now achievable for the main transitions.

The literature on radiative data on each single ion is too extensive to be provided here. We refer the reader to the on-line references (http://www.chiantidatabase.org/chianti_direct_data.html) in the CHIANTI database, where details about the radiative data for each ion are provided. We also note that the NIST database at http://physics.nist.gov/PhysRefData/ASD/lines_form.html provides an extensive and very useful bibliography of all the published radiative data for each ion. NIST also provides a series of assessment reports of theoretical and experimental data.

Here, we just mention the most recent GRASP2K calculations not just because they are very accurate, but also because they cover entire isoelectronic sequences, and references to previous studies on each ion in the sequence can be found.

Jönsson et al. (2011) carried out GRASP2K calculations for states of the $2s^{2}\,2p^{2},\,2s\,2p^{3}$, and $2p^{4}$ configurations in C-like ions between F iv and Ni xxiii. These calculations have been supplemented with those from Ekman et al. (2014) for ions in the C-like sequence, from Ar xiii to Zn xxv, by including levels up to $n=4$.

Rynkun et al. (2012) carried out calculations for transitions among the lower 15 levels ($2{s}^{2}\,2{p}, 2{s}\,2{p}^2, 2{p}^3$) in ions of the B-like sequence, from N iii to Zn xxvi. Rynkun et al. (2013) calculated radiative data for transition originating from the $2{s}^{2}\,2{p}^{4}, 2{s}\,2{p}^{5}$, and $2{p}^{6}$ configurations in all O-like ions between F ii and Kr xxix. These calculations have recently been extended to include many $n=3$ levels, for ions from Cr xvii to Zn xxiii by Wang et al. (2017). Rynkun et al. (2014) calculated transition rates for states of the $2{s}^{2}\,2{p}^{3}, 2{s}\,2{p}^{4}$, and $2{p}^{5}$ configurations in all N-like ions between F III and Kr xxx. These calculations have recently been extended to include levels up to $n=4$, for ions from Ar XII to Zn XXIV, by Wang et al. (2016b). Jönsson et al. (2013a) carried out GRASP2K calculations for the $2{s}^{2}\,2{p}^{5}$ and $2{s}\,2{p}^{6}$ configurations in F-like ions, from Si vi to W lxvi. The calculations on F-like ions has recently been extended by Si et al. (2016) to include higher levels up to $n=4$ for ions from Cr to Zn. Jönsson et al. (2013b) calculated radiative data for levels up to $n=4$ for the B-like Si x and all the B-like ions between Ti XVIII and Cu XXV. Jönsson et al. (2014) carried out GRASP2K calculations for the $2{p}^{6}$ and $2{p}^{5}\,3{l}$ configurations in Ne-like ions, between Mg III and Kr XXVII. These calculations have recently been extended to include levels up to $n=6$, for all Ne-like ions between Cr xv and Kr xxvii by Wang et al. (2016a).

Wang et al. (2015) calculated radiative data for ions along the Be-like sequence.

Jönsson et al. (2016) calculated radiative data for the $3{s}^2\,3{p}^2, 3{s}\,3{p}^3$ and $3{s}^2\,3p\,3d$ configurations of all the Si-like ions from Ti ix to Ge xix, plus Sr xxv, Zr xxvii, Mo xxix. Gustafsson et al. (2017b) performed GRASP2K calculations for 3*l* 3$l'$, 3*l* 4$l'$, and $3s\,5l$ states in Mg-like ions from Ca ix to As xxii, and Kr xxv. Ekman et al. (2018) performed GRASP2K calculations for ions in the Al-like sequence, from Ti X through Kr XXIV, plus Xe XLII, and W LXII. The radiative data are for a large set of 30 configurations: $3s^2\{3l,4l,5l\}, 3p^2\{3d,4l\}, 3s\{3p^2,3d^2\}, 3s\{3p3d,3p4l,3p5s,3d4l'\}, 3p\,3d^2, 3p^3$ and $3d^3$ with $l=0,1,\ldots ,n-1$ and $l'=0,1,2$.

### Electron-ion scattering calculations

As the electron-ion collisions are the dominant populating process for ions in the low corona, significant effort has been devoted to calculate electron-ion scattering collisions over the past decades. The collision strengths usually have a slowly varying part and spikes, that are due to resonances (dielectronic capture). The theory of electron-ion collisions has received significant contributions by M. J. Seaton (University College London), P. Burke (Queens University of Belfast), and A. Burgess (University of Cambridge), among many other members of a large community of atomic physicists. Standard textbooks and reviews on electron-ion collisions are Mott and Massey (1949), Burgess et al. (1970), Seaton (1976), Henry (1981) and Burke (2011).

Using Rydberg units, the nonrelativistic Hamiltonian describing an ion with *N* electrons and a central nucleus having charge number *Z* and a free electron (i.e., the $(N+1)$-electron system) is

76 $$\begin{aligned} {H (Z,N+1)} = - \sum _{i=1}^{N+1} \left( \nabla _i^2 + {2Z \over r_i} \right) + \sum ^{N}_{i < j} {2 \over r_{ij}}. \end{aligned}$$

We search for solutions of the time-independent Schrödinger equation for the $(N+1)$-electron system

77 $$\begin{aligned} H(Z,N+1) \varPsi _c = E \varPsi _c \end{aligned}$$

where *E* is the total energy of the $(N+1)$-electron system, and $\varPsi _c$ is the wavefunction of the $(N+1)$-electron system. The $\varPsi _c$ are usually expanded in terms of products of wavefunctions of the *N*-electron target and those of the free electron.

Within the so called *close-coupling* (CC) approximation, the scattering electron sees individual target electrons, and a set of integro-differential equations need to be solved. An efficient way to solve the scattering problem is to use the *R*-matrix method, described in Burke et al. (1971), Burke and Robb (1976) and Burke (2011). Such method was further developed over many years by the Iron Project, an international collaboration of atomic physicists, which was set up to calculate electron excitation rates for all the iron ions. A significant contribution to the development of the Iron Project programs was made by Berrington, see e.g., Berrington et al. (1987).

The analysis of the scattering interaction

78 $$\begin{aligned} \langle \varPsi _i | H(Z,N+1) - E | \varPsi _{i'} \rangle \end{aligned}$$

involves the calculation of the elements of the so-called reactance matrix, $$ K_{ii'}$$. The transmission matrix $$T_{ii'}$$ is related to the reactance matrix:

79 $$\begin{aligned} T = \frac{-2\mathrm{i} K}{1-\mathrm{i} K}, \end{aligned}$$

and the resulting scattering matrix $\mathcal S$

80 $$\begin{aligned} \mathcal{S} = 1-T = {1+ \mathrm{i} K \over 1- \mathrm{i} K} \end{aligned}$$

is unitary. The dimensionless collision strength ($\varOmega _{if}$) for any transition $i-f$ from an initial *i* to a final state *f* is then obtained from the transmission matrix

81 $$\begin{aligned} \varOmega _{if} \propto |T_{if}|^2. \end{aligned}$$

The collision strengths are usually calculated over a finite range of the energy of the incoming electron. Within the Iron Project codes, the collision strengths are extended to high energies by interpolation using the appropriate high-energy limits in the Burgess and Tully (1992) scaled domain. The high-energy limits are calculated following Burgess et al. (1997) and Chidichimo et al. (2003).

#### DW UCL codes

In the *Distorted Wave* (DW) approximation, the coupling between different target states is assumed to be negligible and the system of coupled integro-differential equations is significantly reduced. A general method which includes the effects of exchange between the free and a bound electron, was developed by Eissner and Seaton (1972) at UCL. The method requires that the free-electron wavefunction $\theta _i$ are orthogonal to those of the one-electron bound orbitals with the same angular momentum. This is achieved by adopting a general expression for the total wavefunction of the $N+1$ system of the form

82 $$\begin{aligned} \varPsi =\mathcal{A} \sum _i \chi _i(\mathbf {x}_1,\ldots , \mathbf {x}_{N}) \theta _i(\mathbf {x}_{N+1}) + \sum _j \phi _j c_j, \end{aligned}$$

where $\phi _j$ is a function of bound state type for the whole system. The functions $\chi _i$ and $\phi _j$ are constructed from one-electron orbitals, and $\mathcal{A}$ is an operator that guarantees antisymmetry. The functions $\phi _j$ are called correlation functions, which are mainly introduced to obtain orthogonality. Note that the above general expression with the correlation functions was adopted by the UCL DW code, mainly developed by Eissner and Seaton (1972) and Eissner (1998). This code has been widely used for a very long time and has produced a very large amount of atomic data.

The first accurate scattering calculations for the coronal iron ions were carried out with the UCL DW codes (see, e.g., Mason 1975a). These were prompted by ground-based eclipse observations of the forbidden lines. These were also needed for the early EUV Skylab and OSO-7 observations in the 1970s. There was a series of papers from Bhatia and Mason. Such calculations were further revised and improved by several groups. These calculations were generally carried out in intermediate coupling that is transforming the LS coupling calculation using term coupling coefficients. This approximation was found adequate for most coronal ions.

**Fig. 32:**
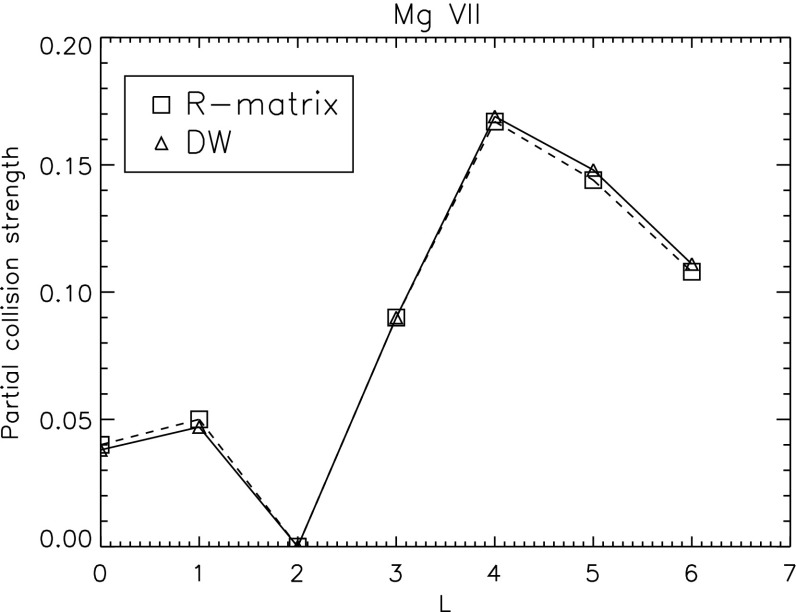
A comparison of partial collision strengths calculated with the DW and *R*-matrix codes, for the Mg vii $2{s}^2\,2{p}^2\,{}^1\hbox {S}-2{s}\,2{p}^3\,{}^1\hbox {P}$ transition at 10 Ry Reproduced with permission from Burgess et al. (1991)

Although the effects of resonance enhancement can be included in DW calculations, usually it is not. DW calculations typically agree with the background collision strength values at lower energies, lower than the maximum threshold. At energies higher than the maximum threshold, DW and *R*-matrix calculations should agree, if the same wavefunctions are used. For a comparison between DW and *R*-matrix calculations see, e.g., Burgess et al. (1991). Excellent agreement between the collision strengths calculated with the UCL DW and the *R*-matrix codes was found, to within a few percent. Figure 32 shows as an example the partial collision strengths calculated at 10 Ry for the Mg vii $2{s}^2\,2{p}^2\,{}^1\hbox {S}-2{s}~2{p}^3\,{}^1\hbox {P}$ transition.

#### Other codes: excitation by electron impact

Several other codes have been developed over the years to calculate cross-sections for excitation by electron impact, and have produced a large amount of atomic data. For example, D. Sampson and H. L. Zhang developed several codes based on the Coulomb–Born-exchange method (cf. Sampson et al. 1979). Widely used DW codes are the Flexible Atomic Code (FAC) (Gu 2003, 2008), and HULLAC (Bar-Shalom et al. 1988). Recently, the autostructure DW code (Badnell 2011) has also been implemented. FAC and autostructure are publicly available, as the UCL DW.

We note that different assumptions are made in the various DW codes. For example, the autostructure DW code does not impose the orthogonality condition and the second term in the expansion is not present, but it does calculate all the appropriate exchange overlaps. In the DW approximation, the scattering equations are uncoupled and not all the elements of the reactance matrix $$ K_{ii'}$$ need to be calculated. Another important difference is whether the DW method is unitarized. Some of the DW codes, such as the FAC and HULLAC, use the relation

83 $$\begin{aligned} T = \frac{-2\mathrm{i}K}{(1-\mathrm{i} K)} \times \frac{(1+\mathrm{i} K)}{(1+\mathrm{i} K)} = \frac{-2\mathrm{i} K + 2K^2}{1 + K^2} \approx -2\mathrm{i} K, \end{aligned}$$

in which case the DW method is called non-unitarized. As pointed out by Fernández-Menchero et al. (2015a), the non-unitarized DW method can lead to large errors (factors of 10) for a few weak transitions, where the coupling in the scattering equations becomes important. The original and default version of the autostructure DW was also non-unitarized, but a unitarized option, which provides good agreement with the fully close-coupling calculations, has recently been implemented (Badnell et al. 2016).

The *R*-matrix codes, used within the Iron Project for the scattering calculations, are described in Hummer et al. (1993), Berrington et al. (1995), Burgess (1974) and Badnell and Griffin (2001), and have been applied to the calculations of many ions. The main repository for the codes is the UK APAP web page maintained by N. R. Badnell at http://www.apap-network.org/codes.html.

Many of the calculations for the astrophysically important (not heavy) ions are carried out in *LS*-coupling, and relativistic corrections are applied later. The *R*-matrix calculations are carried out in an inner and outer region. The *R*-matrix calculations in the inner region normally include the mass and Darwin relativistic energy corrections. Many of the outer region calculations are carried out with the intermediate-coupling frame transformation method (ICFT), described by Griffin et al. (1998). This method is computationally much faster than the Breit–Pauli *R*-matrix method (BPRM).

A fully relativistic Dirac *R*-matrix code, called DARC, was developed by P. H. Norrington and I. P. Grant (see, e.g., Norrington and Grant 1981), and is also available at the UK APAP web page. Several comparisons between the results obtained by the various codes have been carried out, and are normally satisfactory. For example, Badnell and Ballance (2014) compared the *R*-matrix results of ICFT, BPRM and DARC on Fe iii. However, discrepancies for weaker transitions have also been reported (see, e.g., the discussion in Badnell et al. 2016).

A different approach, the B-spline R-matrix (BSR) method was developed by other authors (see, e.g., Zatsarinny 2006; Zatsarinny and Bartschat 2013, for details). The method uses term-dependent non-orthogonal orbital sets for the description of the target states. The wave functions for different states are then optimized independently, which result in a much better target representation. This method is computationally demanding and has mostly been used for the calculations of cross sections for neutrals and low charge states, where an accurate representation of the target states is typically difficult (see, e.g., Wang et al. 2013). In a recent work, a detailed comparison of similar-size calculations for the same ion with the BSR, ICFT, and DARC was carried out by Fernández-Menchero et al. (2017). As in the previous comparisons, good agreement was found among the lower and stronger transitions. Significant differences were however found for the weaker transitions and for transitions to the higher states. The differences were mainly due to the structure description and correlation effects, rather than due to the different treatment of the relativistic effects in the three codes.

Other approaches and codes exist. For example, the Dirac R-matrix with pseudo-states method (DRMPS), described in Badnell (2008). For H-like systems, the convergent close-coupling (CCC) method of Bray and Stelbovics (1992) and the relativistic convergent close-coupling (RCCC) method (Bostock 2011) provide extremely accurate cross sections.

### Uncertainties in electron-ion excitation rates and in the level population

In this section, we provide some examples on various effects which can reduce the accuracy of the collision strengths and the derived rates. Clearly, if the atomic structure is not accurate, this can have a direct effect on the rates. For example, the high-energy limits of the collision strengths for dipole-allowed transitions are directly related to the *gf* values (Burgess et al. 1997). Therefore, any differences in *gf* values obtained with different structure calculations are directly reflected as differences in the collision strengths. The comparisons of the *gf* values in Fig. 26 were used by Del Zanna et al. (2015a) to explain the discrepancies in the rates for Fe xiv calculated by Aggarwal and Keenan (2014).

**Fig. 33:**
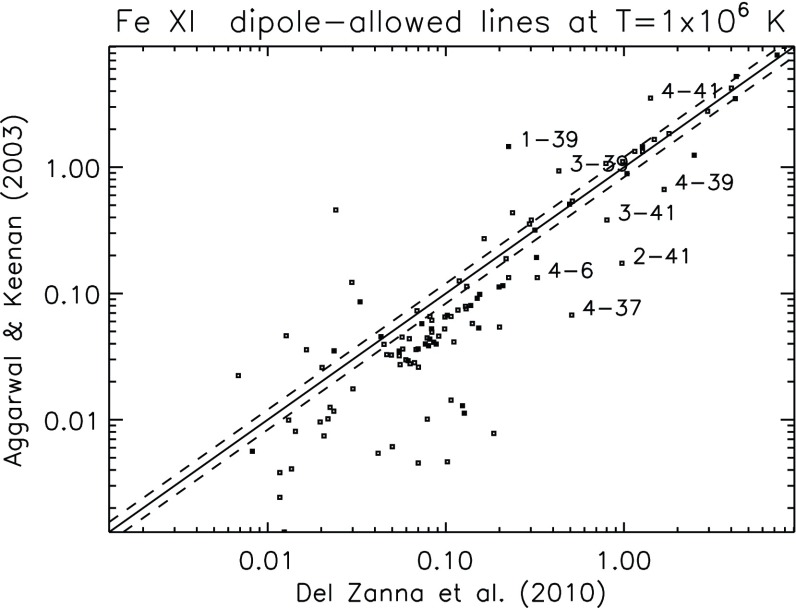
Effective collision strengths for the strongest Fe xi transitions as calculated by Aggarwal and Keenan (2003) and Del Zanna et al. (2010b). The dashed lines indicate $\pm \,20\%$. The largest differences for the strongest transitions are related to decays from $J=1$ levels (e.g., 4–37, 1–39, 3–39, 4–39, 2–41, 3–41, 4–41 indicated in the figure) Image adapted from Del Zanna et al. (2010b)

The calculations of dipole-allowed collision strengths for simpler ions obtained with different targets are normally in agreement, within 10–20%, while results for weaker transitions can differ by significant factors. However, even strong transitions in complex ions can sometimes be difficult to calculate accurately. For example, as we have previously mentioned, transitions to levels with a strong spin–orbit interaction are very sensitive to the target wavefunctions. Figure 33 shows as an example a comparison between rates for Fe xi lines, as calculated in two different ways. The largest differences are for a few of the brightest transitions, and are related to decays from three $J=1$ levels which have a strong spin–orbit interaction. Indeed, as shown in Del Zanna et al. (2010b), large differences in the *gf* values for these transitions are present.

Semi-empirical corrections are normally not applied to the scattering calculations. However, they can provide significant improvements to the rates (see, e.g., Fawcett and Mason 1989; Del Zanna and Badnell 2014).

**Fig. 34:**
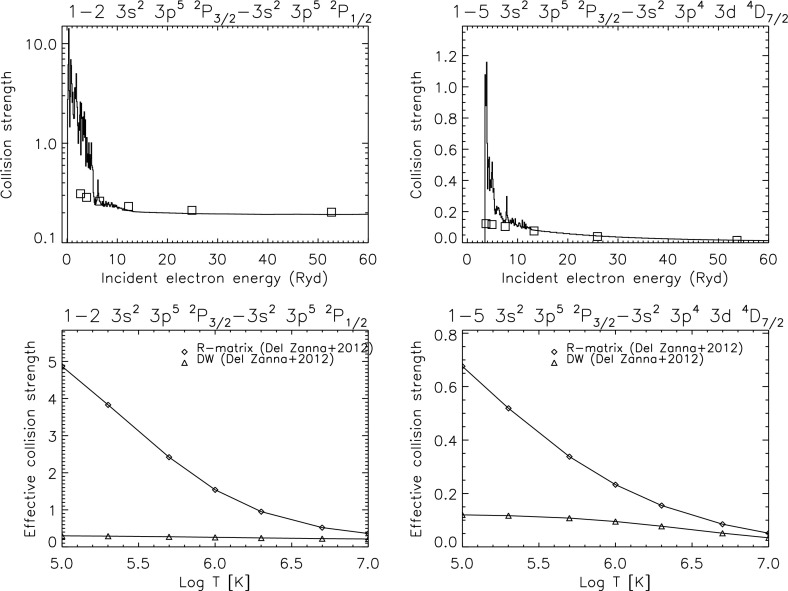
Above: collision strengths, averaged over 0.1 Ryd in the resonance region. The data points are displayed in histogram mode. Boxes indicate the DW values. Below: thermally-averaged collision strengths, with other calculations. The plots on the left are for the Fe x forbidden red coronal line within the ground configuration, those on the right for the Fe x forbidden 257.26 Å transition Adapted from Del Zanna et al. (2012a)

**Fig. 35:**
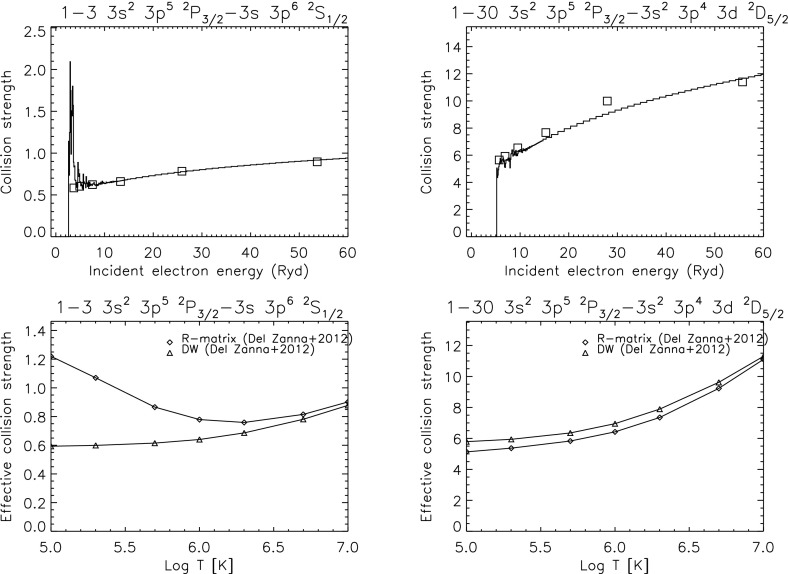
Above: collision strengths, averaged over 0.1 Ryd in the resonance region. The data points are displayed in histogram mode. Boxes indicate the DW values. Below: thermally-averaged collision strengths, with other calculations. The plots on the left are for the Fe x allowed 345.7 Å transition, those on the right for the allowed 1–30 174.5 Å transition, the strongest in the EUV Adapted from Del Zanna et al. (2012a)

Another factor in the accuracy of a calculation is the type of scattering approximation used. DW calculations are known to considerably underestimate collision strengths for some types of transitions. The resonances at lower energies typically affect the rates at lower temperatures, where the DW and *R*-matrix calculations can provide very different values for the rates. The contribution of the resonances varies with the type of transition. For dipole-allowed transitions, resonance effects can be very small. On the other hand, they are usually significant for forbidden and intersystem lines, because the background collision strength values are much lower. Figure 34 shows as an example the collision strengths and rates for forbidden transitions in Fe x, one within the ground configuration, and one not. In contrast, Fig. 35 shows collision strengths and rates for two allowed transitions in Fe x. We can see that resonances can occasionally have a significant contribution to the collision strengths even for strong dipole-allowed transitions.

**Fig. 36:**
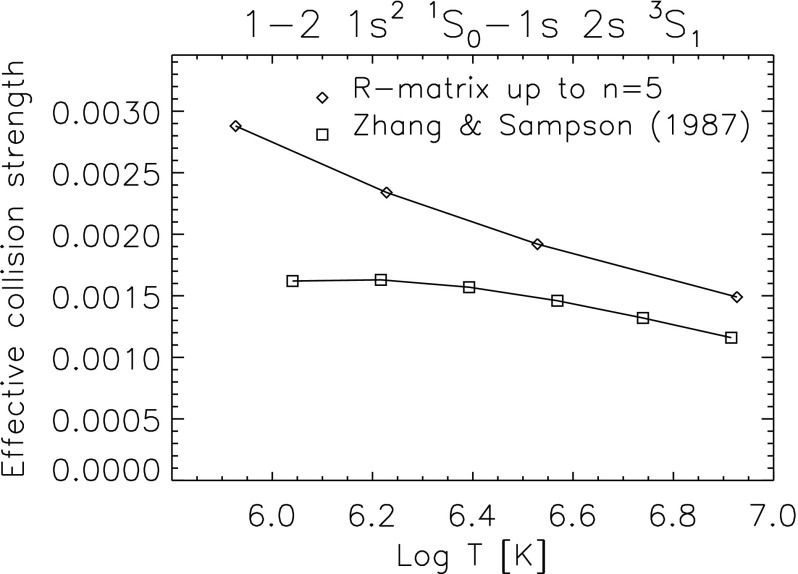
The rate for the forbidden line in Si xiii as calculated with the *R*-matrix codes by Whiteford et al. (2001) and by Zhang and Sampson (1987) with the DW method, including resonance enhancements

We note that resonances can be included within DW calculations with a perturbative treatment (DW $+$ resonances), as e.g., in H. L. Zhang and D. Sampson codes and FAC (although they are not normally included). We also note that generally the resulting collision strengths, even with the resonances included, are lower than those calculated with the close-coupling *R*-matrix calculations. An example is shown in Fig. 36, where the collision strengths for a He-like forbidden transition as calculated by Zhang and Sampson (1987) is compared to the result of an *R*-matrix calculation by Whiteford et al. (2001) (see further details in Badnell et al. 2016). This issue has been studied by Badnell et al. (1993), where significant differences between the DW $+$ resonances treatment and close-coupling *R*-matrix calculations for Mg-like ions were found. Recently, Fernández-Menchero et al. (2016b) compared two similar large-scale calculations for Fe xxi, an *R*-matrix one and an earlier DW $+$ resonances calculation of Landi and Gu (2006). Again, the comparison showed a systematic underestimate of the cross sections by the perturbative results.

**Fig. 37:**
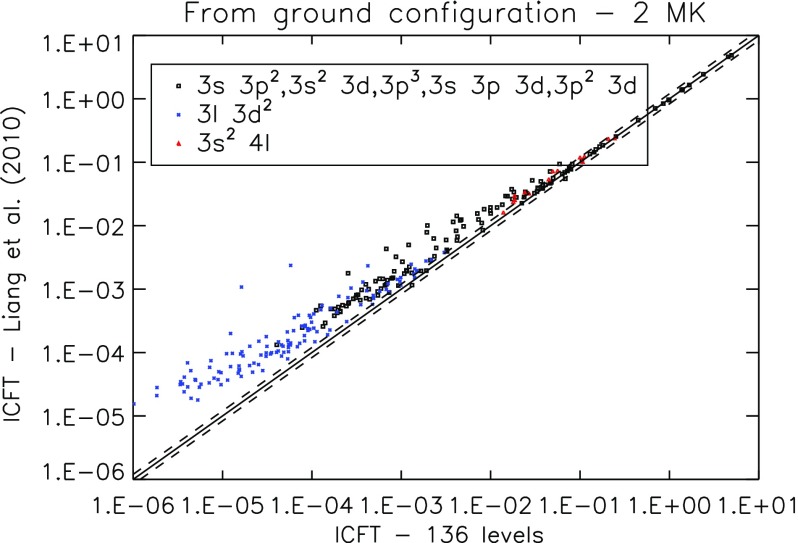
Thermally-averaged collision strengths $\varUpsilon$ for Fe xiv transitions from the ground configuration $3{s}^2\,3p$ levels at 2 MK, as calculated with a smaller target of 136-levels CI/CC, compared to those obtained by Liang et al. (2010) with a larger CC (197). The dashed lines indicate $\pm \,20\%$ Image adapted from Del Zanna et al. (2015a)

The resonances included in e.g., the *R*-matrix calculations are never complete, but depend on the number of levels included in the CC calculations. In other words, the number of configurations/levels included in the close-coupling calculations has a relevance to the accuracy of the effective collision strengths, especially towards lower temperatures. There are countless examples in the literature. Figure 37 shows the values for Fe xiv as calculated (with the same codes and approximations) with a more limited CC (136-levels) and a larger CC (197 lowest-lying levels). It is clear that there is an overall enhancement in the values obtained from the larger calculation. Some of the scatter is due to the difference in CI, related to the differences in *gf* values as we have shown in Fig. 26.

Resonance effects are more important for transitions to low-lying levels, as the number and strength of the resonances decreases with the energy of the levels. Until recently, it was thought that resonances would have a negligible effect on transitions to $n=4$ levels. However, non-negligible contributions are present, as discussed e.g., for the Fe x case in Del Zanna et al. (2012a).

Another factor in the accuracy of a calculation is the energy resolution in the resonance region. Calculations with progressively finer energy grids and more levels can be used to provide some estimates of the accuracy and convergence of the calculations. One explicit example for Fe xi was provided in Del Zanna et al. (2010b).

So far, we have discussed some of the uncertainties in the basic atomic rates. Ultimately, the best way to provide an estimate of the uncertainty of an atomic dataset is to compare calculated line intensities with observed ones, so long as the instrumental calibration is well known. However, two main questions still need to be addressed:

1. how do the uncertainties on the rates affect the line intensities?
2. how can one provide an uncertainty on the rates?

The question on how the uncertainties on the rates affect the spectral line intensities is not a trivial one to answer. One actually needs to identify the main populating processes for the levels of interest and for the densities of interest, and then assess which rates are the most important ones.

**Fig. 38:**
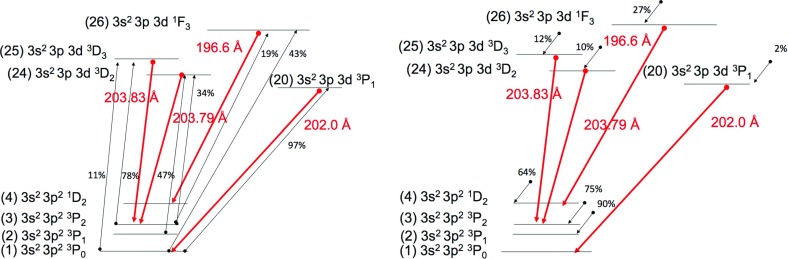
Main populating processes for Fe xiii at a relatively low density of $10^{8}\,\hbox {cm}^{-3}$. The red downward arrows indicate the main density diagnostic lines in the EUV. The upward black arrows indicate the main populating transitions; the percentage contribution to the population of the upper level is indicated. The downward arrows and percentages on the right plot indicate the contributions to the level populations due to cascading from all the higher levels

**Fig. 39:**
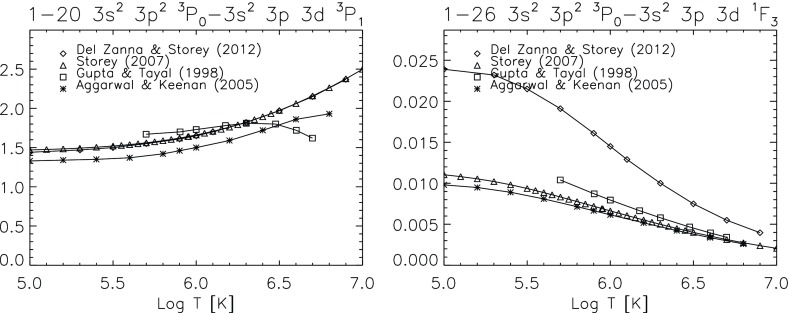
Rates for electron excitation for two important allowed transitions in Fe xiii, as obtained from different calculations

Figure 38 shows the main populating processes for Fe xiii at a relatively low density of $10^{8}\,\hbox {cm}^{-3}$, using the atomic data calculated by Del Zanna and Storey (2012), which included cascading from $n=5,6$ levels. The 202.044 Å line is mainly (97%) populated by direct excitation from the $3{s}^2\,3{p}^2\,{}^3\hbox {P}_{0}$ ground state via a strong dipole-allowed transition. This means that the uncertainty for the intensity of the 202.044 Å line is solely related to the uncertainty in the rate for direct excitation and the A value. The strength of this transition means that it is relatively easy to calculate the rate accurately. Figure 39 (left) shows the effective collision strength for this transitions, as calculated by different authors. With the exception of Gupta and Tayal’s calculation, there is excellent agreement within a few percent between all the calculations.

On the other hand, the populations of the levels which provide the density sensitivity for this ion are due to excitation from several levels, and cascading from higher levels. For example, the $3{s}^2\,3p\,3d\,^1\hbox {F}_{3}$ level, which produces a strong allowed transition at 196.6 Å, has a contribution to its population of about 43% from direct excitation from the ground state, and about 24% from cascading from higher levels (at a density of $10^{8}\,\hbox {cm}^{-3}$). The transition from the ground state is weak and forbidden, and as shown in Fig. 39 (right), different calculations have provided quite different rates. For these reasons, the intensity of the 196.6 Å line is intrinsically more uncertain than the 202.044 Å line.

Similarly, the populations of the $3{s}^2\,3p\,3d\,^3\hbox {D}_{3,2}$ levels which produce the strong allowed transitions at 203.8 Å are mainly due to excitation from the $3{s}^2\,3{p}^2\,{}^3\hbox {P}_{1,2}$ levels, although non-negligible contributions (10%) comes also from cascading from higher levels. In turn, the populations of the $3{s}^2\,3{p}^2\,{}^3\hbox {P}_{1,2}$ levels are mainly due to cascading from higher levels, as indicated in Fig. 38 (right). Therefore, many rates are actually responsible for the intensities of the lines at 203.8 Å.

The fact that cascading has a considerable effect on the populations of the lower levels means that the size of the target can also in principle indirectly affect the intensities of the lines. One recent example where the size of the target was found to have a significant effect on the populations of the levels within the ground configuration regards the important coronal iron ion Fe xii (Del Zanna et al. 2012b). The increased population of the ground configuration levels in turn affected all the density diagnostics that were associated with these levels, and the forbidden lines within the ground configuration.

Another issue is that the main populating processes change with density. Overall, obtaining the correct theoretical behaviour for most lines requires accurate scattering and radiative data for a large set of levels.

The question of how one can provide an uncertainty on the rates is also non trivial. One possibility is to compare the rates as obtained from the same set of codes and approximation but by changing some parameters, such as the CI/CC expansions in the calculations.

**Fig. 40:**
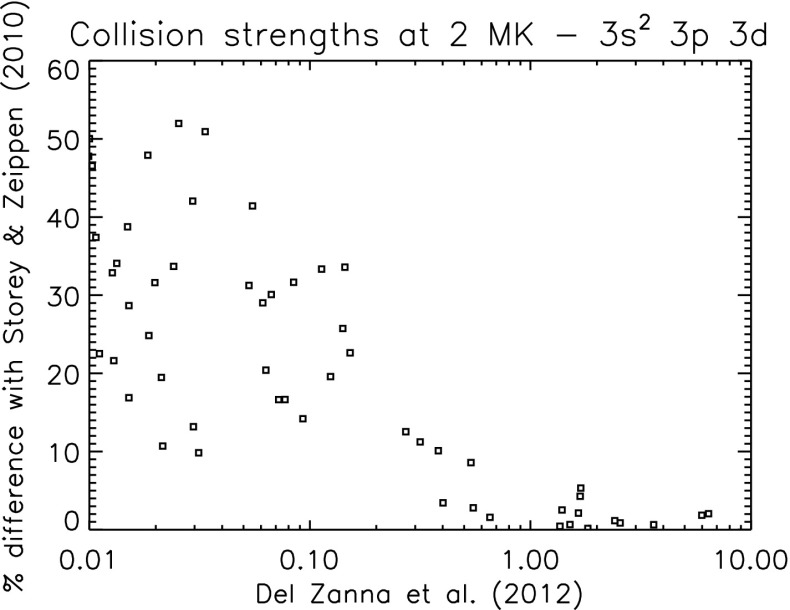
Percentage difference in the A values of Fe xiii transitions within the energetically lowest 27 levels, as obtained with two different calculations

For the excitation rates, as we have seen there are many factors that can affect the end result, so sometimes comparisons can show very large differences. A more instructive comparison is when two calculations obtained with the same codes and approximations are considered, and only the CI/CC expansions are different. Such a comparison is shown in Fig. 40, where the rates at peak Fe xiii abundance as calculated by Del Zanna and Storey (2012) which included a total of 749 levels up to $n=4$ in the CC expansion, and by Storey and Zeippen (2010), where 114 fine-structure levels within the $n=3$ complex were included. It is clear that very good agreement is present for the strongest transitions, while significant variations are present for the weaker ones.

### Electron excitation data

A review of effective collision strengths for coronal ions was published in a single volume (No. 57) of *Atomic Data and Nuclear Data Tables* in preparation for the SoHO mission. This is based on a meeting that was held in 1992 (Lang 1994). The volume contains review articles, and includes discussions of the atomic data for the main isoelectronic sequences. Since then, a significant number of new calculations have been published by various groups. Many are detailed in each of the CHIANTI database papers, where the most up-to-date calculations are assessed. As in the case of the radiative data, the references for the data included in the CHIANTI database can be found on-line at http://www.chiantidatabase.org/chianti_direct_data.html.

Atomic data for several ions have also recently been calculated at Queens University of Belfast. They are available at http://web.am.qub.ac.uk/apa/data/. A large set of excitation data have been produced by the Iron project and the earlier Opacity Project. A series of Iron Project papers have been published in Astronomy and Astrophysics. These data are available from the TIPTOPbase (http://cdsweb.u-strasbg.fr/OP.htx).

An even larger set of excitation data has been produced recently by the UK Rmax and UK APAP network (data are available at http://www.apap-network.org). using mostly the Iron Project R-matrix codes (see the review in Badnell et al. 2016) but also the new DW code within the autostructure program (Badnell 2011).

Below we summarise the latest calculations for each isoelectronic sequence, focusing on the coronal and transition-region ions of diagnostic importance. Further details on previous calculations can be found in the literature cited.

#### H-like ions

*R*-matrix calculations for several ions in this sequence (from He to Ne) were carried out by Ballance et al. (2003). *R*-matrix calculations for other ions also exist, and they are referenced in the CHIANTI database. Recently, Fernández-Menchero et al. (2016a) showed that excitation rates to levels higher than $n=5$ can be estimated quite accurately with extrapolation procedures.

#### He-like ions

Many calculations for the He-like ions exist in the literature. Earlier DW calculations such as those of Zhang and Sampson (1987) for the $n=2$ transitions have been supplemented by several *R*-matrix calculations. For the ions from C v to Zn xxix, the most accurate *R*-matrix calculations are those of Whiteford et al. (2001), mainly because radiation damping is included, unlike other calculations. In fact, radiation damping is an important effect for H- and He-like ions, as discussed e.g., in Gorczyca and Badnell (1996) and Griffin and Ballance (2009). Whiteford et al. (2001) calculated atomic data for all the transitions among the 49 levels up to 1*s* 5*l*. These *R*-matrix rates show a significant enhancement for the forbidden transition in several ions of the sequence, compared to the Zhang and Sampson (1987) data, despite the fact that resonance enhancement associated with the $1s\,3l^{\prime \prime }\,n^{\prime }\,l^{\prime \prime \prime }$ autoionizing levels of the Li-like ions was added to the DW calculations. Recently, Fernández-Menchero et al. (2016a) showed that excitation rates to levels higher than $n=5$ can be estimated quite accurately with extrapolation procedures.

#### Li-like ions

The most complete scattering calculations for electron-impact excitation of all Li-like ions from Be$^+$ to Kr$^{33+}$ is the APAP one from Liang and Badnell (2011), where the radiation- and Auger-damped ICFT *R*-matrix approach was used. The model ions included 204 close-coupling (CC) levels, with valence electrons up to $n=5$ and core-electron excitations up to $n=4$.

#### Be-like ions

The early excitation rates for many ions in this sequence were not very accurate since they were interpolated (see, e.g., Keenan et al. 1986b). This was pointed out by Del Zanna et al. (2008) who carried out an ICFT *R*-matrix calculation for Mg ix, an ion of particular importance for its temperature diagnostic applications. Significant (up to 50%) differences were found in some of the main lines and diagnostics, when using the *R*-matrix data rather than the interpolated ones.

The largest calculations for many ions in the sequence (from $\mathrm{B}^{+}$ to $\mathrm{Zn}^{26+}$) are from the APAP team (Fernández-Menchero et al. 2015b), where the ICFT *R*-matrix method was used. The CI and CC expansions included atomic states up to $nl=7\mathrm{d}$, for a total of 238 fine-structure levels. Good agreement with the previous *R*-matrix calculations for Mg ix (Del Zanna et al. 2008) and Fe xxiii (Chidichimo et al. 2005) were found.

However, these results were recently questioned by Aggarwal and Keenan (2015), because of large differences with the results of their DARC calculation. As shown by Fernández-Menchero et al. (2015b), the DARC calculation of Aggarwal and Keenan (2015) were less accurate, because of their more limited CI and CC expansions. As a follow-up, Aggarwal et al. (2016) carried out a larger DARC calculation for N iv, using the same set of configurations as that one adopted by Fernández-Menchero et al. (2015b). Large differences were still found, for transitions to highly excited levels. We should point out that good agreement (within 10–20%) is present for the strong transitions, and that transitions to high levels are typically more uncertain. This was shown by a recent calculation for N iv using a completely different approach and set of codes (the B-spline *R*-matrix) by Fernández-Menchero et al. (2017). The B-spline *R*-matrix is computationally intensive but provides much more accurate energies than the other methods. Significant differences in the rates to highly excited levels as calculated by the ICFT, DARC and B-spline *R*-matrix were found.

#### B-like ions

The most up-do-date calculation for all boron-like ions from $\hbox {C}^+$ to $\hbox {Kr}^{31+}$ is the APAP one by Liang et al. (2012), where the ICFT *R*-matrix method was used. 204 close-coupling levels were included in the target. These data are a significant improvement for many ions where only $n=2,3$ DW data were previously available.

#### C-like ions

Collisional data for 49 fine structure levels of Ne v have been calculated by Griffin and Badnell (2000) using the ICFT *R*-matrix approximation. For the important coronal ion S xi, DW calculations from Landi and Bhatia (2003) are available.

For the important Fe xxi, Badnell et al. (2001) provided ICFT *R*-matrix calculations with a large-scale model ion including 564 levels in the CI expansion and 200 levels, up to $n=4$ in the CC expansion. Recently, Fernández-Menchero et al. (2016b) recalculated the cross sections with the same codes, but including in the CC expansion all the 564 levels. A larger calculation, including some $n=5$ levels was also carried out. Significant differences between this larger calculation and the earlier one were found in the collision strengths, due to the lack of convergence of the earlier data, particularly for the $n=3$ levels.

#### N-like ions

Zhang and Sampson (1999) provided collision strengths for all transitions between the 15 levels of the $2s^2\,2p^3, 2s\,2p^4$ and $2p^5$ configurations of N-like ions, using the DW approximation. Several other DW calculations for these ions exist.

A few *R*-matrix calculations for some important ions also exist. For example, for S x Bell and Ramsbottom (2000) included the 22 levels of the $2s^2\,2p^3, 2s\,2p^4, 2p^5$ and $2s^2\,2p^2\,3s$ configurations.

Ramsbottom and Bell (1997) produced *R*-matrix calculations for Mg vi.

The most complete scattering calculations for the important Fe xx ion are the APAP ICFT *R*-matrix by Witthoeft et al. (2007), which included all the main levels up to $n=4$.

#### O-like ions

For ions of this sequence, several DW calculations exist, with some additional *R*-matrix calculations for the lowest levels.

S ix is an important coronal ion. Several DW calculations exist, the most recent is from Bhatia and Landi (2003) which included $2s^{2}$ $2p^{4}$, $2s\,2p^{5}$, $2p^{6}$ and $2s^{2}$ $2p^{3}$ $3l^{}$ ($l=s,p,d$), corresponding to 86 fine structure levels.

For Fe xix, the most up-do-date calculations are the *R*-matrix ones by Butler and Badnell (2008). The target included 342 close-coupling levels up to $n=4$.

#### F-like ions

The most important coronal ion of this sequence is Fe xviii. Large discrepancies between theory and observations in the strongest X-ray lines were finally resolved, as shown in Del Zanna (2006a), with the APAP ICFT *R*-matrix calculations of Witthoeft et al. (2006). The most complete calculations for ions along this sequence are the APAP ones by Witthoeft et al. (2007), where the ICFT *R*-matrix approach was adopted. The model ions include 195 close-coupling levels up to $n=3$.

#### Ne-like ions

The most important coronal ion of this sequence is Fe xvii. As in the Fe xviii case, large discrepancies between theory and observations in the strongest X-ray lines were present, until large-scale *R*-matrix calculations were performed.

The most up-do-date calculations for ions from $\hbox {Na}^+$ to $\hbox {Kr}^{26+}$ of this sequence are the APAP ones by Liang and Badnell (2010) data, calculated with the ICFT *R*-matrix method. The target included 209 levels, up to outer-shell promotions to $n=7$.

As shown by Del Zanna (2011b), the discrepancies in astrophysical observations were finally resolved with the Liang and Badnell (2010) data, although the Breit–Pauli *R*-matrix calculations of Loch et al. (2006) also showed good agreement with observation.

#### Na-like ions

The latest calculations for Na-like ions from $\hbox {Mg}^+$ to $\hbox {Kr}^{25+}$ are the APAP ones by Liang et al. (2009a) using the ICFT *R*-matrix approach. The close-coupling expansion included configurations up to $n=6$. Inner-shell excitation data with the ICFT *R*-matrix method with both Auger and radiation damping included were produced by Liang et al. (2009b).

#### Mg-like ions

The most complete set of scattering data for ions in this sequence have been produced by the APAP network (Fernández-Menchero et al. 2014) with ICFT *R*-matrix calculations for all the ions from $\mathrm{Al}^{+}$ to $\mathrm{Zn}^{18+}$. The target includes a total of 283 fine-structure levels in both the CI target and CC collision expansions, from the configurations $1{s}^2\,2{s}^2{p}^6\,3\{{s,p,d}\}\,nl$ with $n=4,5$, and $l=0{-}4$. Fe xv is one of the most important coronal ions in the sequence.

Si iii is also an important ion for the transition region, with lines useful for a wide range of diagnostics. The latest atomic data and diagnostics are discussed in Del Zanna et al. (2015c).

#### Al-like ions

The latest *R*-matrix scattering calculation for S iv was carried out by Del Zanna and Badnell (2016a) within the APAP network. A few problems with the previous calculations by Tayal (2000) were found, but good agreement was obtained for the intersystem lines around 1400 Å, which are the main diagnostic lines.

**Fe** **xiv**

Significant discrepancies between observed and predicted intensities of the very strong Fe xiv EUV coronal lines existed until the APAP scattering calculation of Storey et al. (2000). These calculations were further improved by the APAP ICFT *R*-matrix calculations by Liang et al. (2010). Aggarwal and Keenan (2014) carried out a DARC *R*-matrix calculation for this ion and showed large differences with the ICFT ones, suggesting that there might be a problem in the ICFT data. However, Del Zanna et al. (2015a) showed that a smaller ICFT calculation with the same smaller target used in the DARC calculations (136 levels) for the CI and CC expansions agreed quite well with the DARC one. Liang et al. (2010) calculations were further improved in Del Zanna et al. (2015a) by retaining the full set of 228 levels for the CC expansion.

#### Si-like ions

The latest data for S iii have been calculated with the *R*-matrix suite of codes by Hudson et al. (2012).

**Fe** **xiii**

This ion is important for the solar corona. For example, lines observed by Hinode EIS are used to measure electron densities. The largest calculation for this ion is the APAP ICFT *R*-matrix calculation which included a total of 749 levels up to $n=4$ (Del Zanna and Storey 2012).

**Ni** **xv**

Ni xv also produces several EUV lines observed by Hinode EIS that are useful for density and chemical abundance diagnostics of solar active regions. The largest calculation for this ion is the APAP ICFT *R*-matrix calculation which included levels up to $n=4$ (Del Zanna et al. 2014c).

#### P-like ions (Fe xii)

Fe xii lines are prominent in the EUV region of the spectrum. Several of them are routinely observed by Hinode EIS and are used to measure electron densities. The density values obtained from these lines were significantly higher than those from other ions such as Fe xiii (see, e.g., Young et al. 2009).

Fe xii is a notoriously difficult ion to carry out accurate calculations for. It was only with the APAP *R*-matrix (Storey et al. 2005) calculations that theory came close to experiment. The largest calculation for this ion is the APAP ICFT *R*-matrix one by Del Zanna et al. (2012b), with 912 levels up to $n=4$. This new data provides densities from the Hinode EIS lines about a factor of three lower than the previous model by Storey et al. (2005). This is due to the combined effect of extra cascading and increased excitation, which changed the populations of the levels of the ground configuration significantly. This affected the intensities of the forbidden lines and the density diagnostics of the EUV lines.

#### S-like ions (Fe xi)

Fe xi lines are prominent in the EUV and are observed routinely by instruments such as Hinode EIS. Fe xi is another notoriously difficult ion to deal with. Several DW and *R*-matrix calculations have been published, but large discrepancies between observation and theory were present for some of the strongest lines of this ion. The main problem was with the decays from three $n=3, J=1$ levels which have a strong spin–orbit interaction. The collision strengths to these levels are very sensitive to the target. Good agreement with observations was found with an ad-hoc target and APAP ICFT *R*-matrix calculations by Del Zanna et al. (2010b). A larger calculation, including 996 levels up to $n=4$ by Del Zanna and Storey (2013), showed similar effects as found in Fe xii, i.e., significantly increased (30–50%) intensities of strong EUV lines from lower levels due to the combined effect of extra cascading and increased excitation which affected the populations of the (energetically) lowest levels in this ion.

#### Cl-like ions

**Fe** x

Fe x is another important coronal ion. The largest scattering calculation for this ion is the APAP ICFT *R*-matrix one by Del Zanna et al. (2012a) which included levels up to $n=4$. As in the Fe xii and Fe xi cases, the larger calculation resulted in significant increases (30–50%) in the intensities of several EUV lines, in particular the strong decays from the $3{s}^2\,3{p}^4\,3d$ configuration observed by Hinode EIS (the 257.26 Å self-blend), and from the 3s $3{p}^6\,{}^2\hbox {S}_{1/2}^{\mathrm{e}}$ level. The intensities of the visible forbidden lines were also significantly affected, as shown in Del Zanna et al. (2014b).

**Ni** **xii**

The largest scattering calculation for this ion is the APAP ICFT *R*-matrix one by Del Zanna and Badnell (2016b), which included levels up to $n=4$. With the exception of the three strongest soft X-ray transitions, large differences with previous calculations are found, mainly affecting the decays from the lowest $3{s}^2\,3{p}^4\,3d$ levels and the forbidden UV lines.

#### Ar-like ions

**Fe** **ix**

Fe ix is another important ion for the solar corona, as it produces strong EUV lines from the $3{s}^2\,3{p}^4\,3{d}^2$ and $3{s}^2\,3{p}^5\,4p$ configurations, some of which are useful for measuring electron temperatures. As in the Fe xii case, significant problems in the scattering calculations for this complex ion were present, until the Storey et al. (2002) APAP calculation. The largest scattering calculation for this ion is the APAP ICFT *R*-matrix one by Del Zanna et al. (2014b), which included the main levels up to $n=5$.

**Ni** **xi**

Ni xi lines are observed in the soft X-ray and EUV by, e.g., Hinode EIS and are useful density diagnostics. The largest calculation for this ion is the APAP ICFT *R*-matrix one by Del Zanna et al. (2014a), which included the main configurations up to $n=4$. Significant differences with the smaller *R*-matrix results of Aggarwal and Keenan (2007) and the DW calculations by Bhatia and Landi (2011) were found.

#### K-like Fe viii

Fe viii is another coronal ion that has been notoriously difficult to calculate, mostly because of the difficulty in obtaining an accurate atomic structure, as described by Del Zanna (2009b), where Hinode EIS observations were used. Several calculations have been carried out (see, e.g., Griffin et al. 2000; Tayal and Zatsarinny 2011), but it was only recently that agreement with the Hinode EIS observations was achieved, with the APAP ICFT *R*-matrix calculations by Del Zanna and Badnell (2014). The agreement was achieved by adopting a new method which employs semi-empirical corrections within the scattering calculation.

#### Ca-like Fe vii

The most important ion of this sequence is Fe vii, which produces several EUV lines in the Hinode EIS spectral range. The most complete scattering calculation is the ICFT *R*-matrix one by the APAP team (Witthoeft and Badnell 2008). A benchmark of these atomic data against Hinode EIS observations has however indicated several problems (Del Zanna 2009a), some of which are caused by incorrect identifications, as also found by Young and Landi (2009). However, it is still unclear if the scattering calculations should be revised. The Fe vii atomic structure is as complex as the Fe viii one, with several strong lines arising from levels which have a strong spin–orbit mixing.

### Ionisation/recombination rates

Direct ionization (DI) via electron impact is a smoothly-varying function of energy. Earlier approximate studies were those of Lotz (1968) and Seaton (1964b) who introduced semi-empirical formulae. The former was more appropriate for non-equilibrium plasma, while Seaton’s approach provided good cross sections near threshold. Burgess (1964b) introduced the exchange classical impact parameter (ECIP) method which was used extensively. Burgess et al. (1977) later found out that the ECIP method produced good agreement with laboratory data, as good as Coulomb–Born calculations, especially for low charge states and simple ions such as those of the H- and He-like. These authors also suggested a way to include the excitation of auto-ionizing states in the calculations, an approach that was later refined in Burgess and Chidichimo (1983), where good agreement with laboratory data was found.

DI has often been provided for astrophysical applications, as the excitation–autoionization (EA), in the form of parametric fits. Examples can be found in Younger (1981) and Arnaud and Rothenflug (1985).

Various close-coupling calculations of DI of atomic ions have been carried out by Pindzola, Griffin and collaborators for a number of years (see, e.g., the review by Pindzola et al. 2014).

**Fig. 41:**
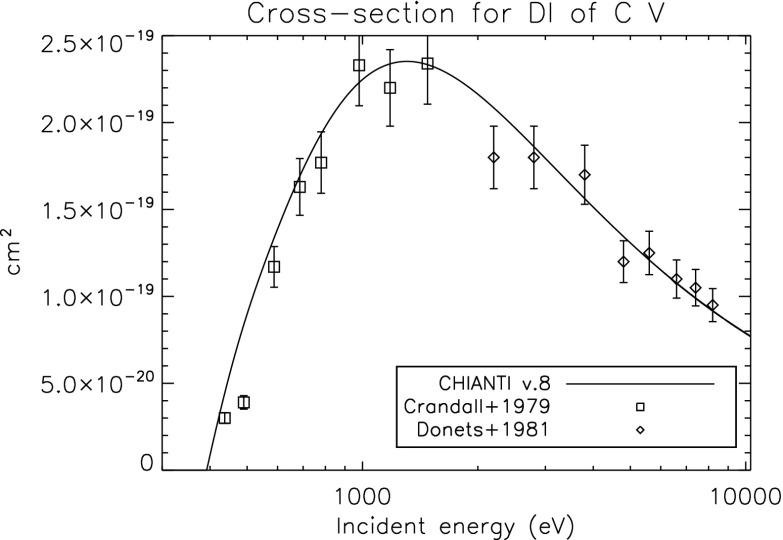
Cross-section for direct ionization of C v, as calculated and measured in the laboratory (see Dere 2007 for details)

A significant revision of the ionization rates was produced by Dere (2007), where total cross-sections for all elements and ions of isoelectronic sequences up to zinc were calculated with the DW code FAC. The calculations only considered the ground states, but both the direct an the EA processes were included. The cross-sections were fitted with splines in a scaled energy domain, also to check for the correct limit behaviour at high energies, as in the case of the cross-sections for collisional excitation. Whenever available, experimental cross-sections were compared to the calculated ones, and sometimes used to apply empirical corrections to the theoretical values. The results, in terms of cross-sections and rates, are stored in the CHIANTI database. An example is shown in Fig. 41.

Further comparisons between these cross-sections and laboratory measurements can be found in a series of papers, based on measurements of electron impact ionisation carried out using the TSR storage ring at the Max Planck Institute for Nuclear Physics in Heidelberg, Germany. Details can be found in the review by Hahn (2014) and in the cited references. A range of ions from many isoelectronic sequences (Li-like to K-like) have been studied, with the exclusion of those ions with long-lived metastable levels which make the measurements of the total cross sections difficult. Good overall agreement between theory and experiment was found. It should be said that in most cases the recent calculations by Dere (2007) are in good agreement with previous ones, although in some cases differences of the order of 10–20% have been found.

Radiative recombination (RR) and dielectronic recombination (DR) are normally calculated separately, as the two processes are quite independent (Pindzola et al. 1992). RR rates are usually calculated from the photoionization cross-sections using the principle of detailed balance. Earlier calculations only considered transitions between the ground states of the ions, plus hydrogenic cross-sections for photoionisations from excited levels of the recombined ion to the ground level of the recombining ion (see, e.g., Aldrovandi and Pequignot 1973). More complete calculations along entire isoelectronic sequences were carried out by Gu (2003) using the FAC code (Gu 2008) and Badnell (2006b) using autostructure. Badnell (2006b) calculated rates for all isoelectronic sequences up to the Na-like sequence. Further autostructure calculations for the Mg, Al and Ar sequences were published in Altun et al. (2007), Abdel-Naby et al. (2012) and Nikolić et al. (2010), respectively. Additional rates for specific iron ions were published by Badnell (2006a). The above rates plus additional ones for the minor ions, originating from a variety of sources, are available within the CHIANTI database.

**Fig. 42:**
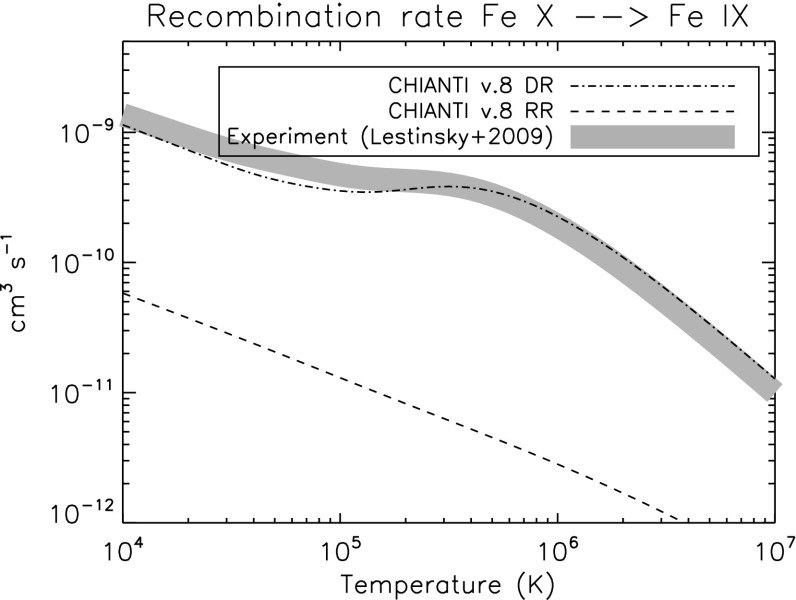
Recombination rates (radiative, RR, and dielectronic, DR) for Fe x (i.e., to recombine Fe x into Fe ix), as stored in the CHIANTI database version 8. The grey area indicates the experimental estimate for the DR rate from Lestinsky et al. (2009). The thickness of the grey area represents the approximate experimental uncertainty

Most of the DR rates currently used in atomic physics codes have been obtained within the DR project (Badnell et al. 2003) with autostructure calculations. One of the most recent papers in the series is Abdel-Naby et al. (2012) (Al-like). Comparisons between DR rates and laboratory measurements show overall good agreement, although discrepancies are present in some cases. The most significant series of studies employed the electron-ion merged-beams method applied to the observations obtained at the heavy-ion storage ring TSR of the Max Planck Institute for Nuclear Physics in Heidelberg, Germany. The DR rates have been obtained by estimating the contribution of the RR rates. A series of papers, a collaboration between N. R. Badnell and the Heidelberg group have been published. Schippers et al. (2010) reviewed such studies for the important iron ions. Figure 42 shows as an example a comparisons between the experimental DR rate for recombination of Fe x as estimated by Lestinsky et al. (2009) (grey area) and the theoretical DR rate as available from the CHIANTI database version 8 (dot-dash line). Note the good agreement between experiment and theory, and also how the RR contribution is nearly negligible for this ion.

### Proton excitation data

A significant number of cross-section for ion excitation by proton impact has been calculated following the semi-classical impact-parameter approach introduced by Seaton (1964a), which is accurate only for low energy collisions. A number of methods have been developed to improve the calculations. Details can be found in the reviews by Reid (1989) and especially Burgess and Tully (2005), where some problems in earlier calculations were highlighted.

For example, Bely and Faucher (1970) used a symmetrised first order semi-classical approximation to calculate the proton cross-sections for a large number of ions with configurations $2p, 2p^5, 3p$ and $3p^5$.

Kastner (1977) used the first order semi-classical approximation for low energies while for higher energies different approximations were adopted.

Landman, in a series of papers, computed proton rates retaining the classical treatment for the proton trajectory, but using a symmetrised, semi-classical close-coupling method to calculate transition probabilities (see, e.g., Landman 1973).

Faucher used a fully close-coupling method to compute proton cross-sections for a number of ions (see, e.g., Faucher et al. 1980).

Reid and Schwarz (1969) developed a symmetrised, semi-classical close-coupling approximation including polarization effects, which was used by Foster, Ryans and others (see, e.g., Ryans et al. 1999) to calculate proton rates for a large number of ions.

Reviews of proton excitation data can be found in Copeland et al. (1997) and in Young et al. (2003), where the data included in the CHIANTI database are listed.

### Benchmarking atomic data

Our current knowledge of the solar spectrum is not only based on a long history of observations (as we summarised in the first section) and of atomic calculations (as we briefly summarised), but also on a continued effort to establish line identifications, blends, and benchmark the reliability of the atomic calculations against experiment. In this section, we provide a few examples and references to recent studies. As the subject is vast, we mainly focus on iron lines, as they are abundant in the XUV spectra of the solar corona, and provide many diagnostic applications.

#### Line identifications

For simple ions, atomic structure calculations have been accurate enough to easily identify all the main spectral lines, hence establish all the experimental energies. For more complex ions, and until recently, ab-initio calculations of wavelengths were typically off by several Å, hence it has not been easy to establish experimental energies, and occasionally identifications have been revised several times. Surprisingly, there are many spectral ranges in the XUV where a large fraction of the observed lines are still unidentified.

Most of the identifications in the past have been based on laboratory spectra, where typically the sources have very different conditions to solar plasma (e.g., where densities are very low in comparison). The identifications were therefore biased in some sense.

A review of EUV $n=3 \rightarrow n=3$ transitions was published in a Culham report (Fawcett 1971b), while a comprehensive bibliographical review of all the identifications in the XUV ($n=2 \rightarrow n=2, n=3 \rightarrow n=3, n=2 \rightarrow n=3, n=4 \rightarrow n=4$) was published in a later Culham report by Fawcett (1990).

General compilations of line identifications and wavelengths in the XUV were produced by Kelly (1987) and Shirai et al. (2000). These compilations, with more recent updates, have been included in the National Institute of Standards and Technology (NIST) database.^3^ However, for several important ions the NIST energies are still in need of revision and are not up-to-date with the most recent literature. In what follows, we mostly mention recent literature for the important iron ions, because this is not included in the NIST compilations.

The identifications of the strongest XUV lines of the most important iron ions have been assessed in a series of benchmark papers, where a significant number of new identifications have been proposed, and where detailed references to previous original identifications can be found: Fe vii (Del Zanna 2009a), Fe viii (Del Zanna 2009b), Fe ix (Del Zanna 2009a), Fe x (Del Zanna et al. 2004a), Fe xi (Del Zanna 2010), Fe xii (Del Zanna and Mason 2005a), Fe xiii (Del Zanna 2011a), Fe xvii (Del Zanna and Ishikawa 2009; Del Zanna 2011b), Fe xviii (Del Zanna 2006a), Fe xxiii (Del Zanna et al. 2005), Fe xxiv (Del Zanna 2006b). This work was mostly focused on the Hinode EIS and SDO AIA spectral ranges, where we knew atomic data and line identification were lacking.

**Fig. 43:**
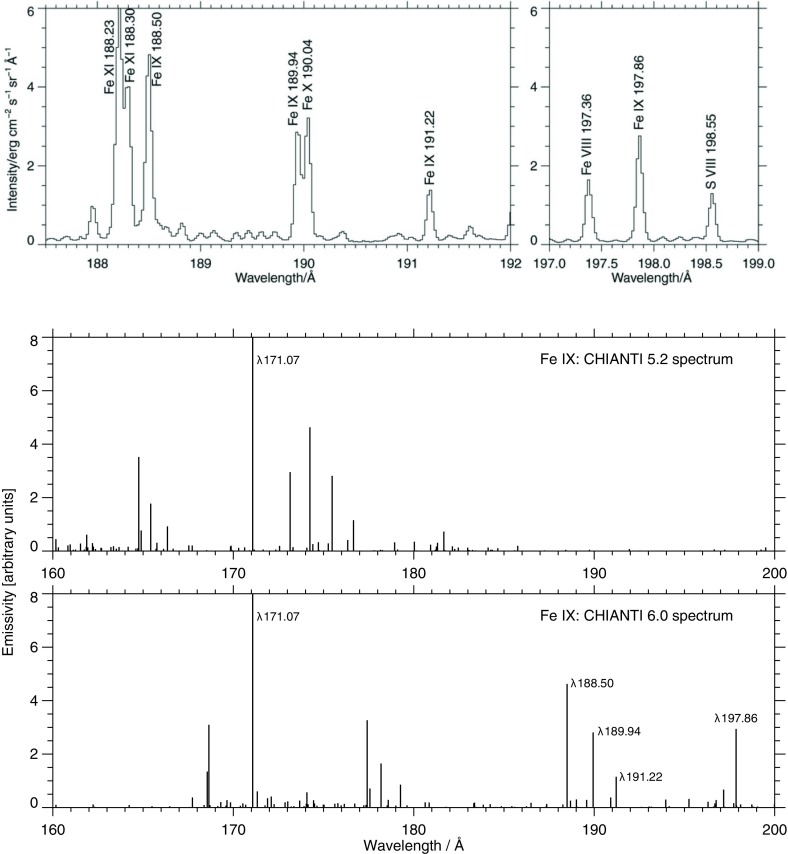
Top: Hinode EIS spectra of the newly identified Fe ix lines. Bottom: simulated spectra Images reproduced with permission from [top] Young (2009) and (bottom) from Young and Landi (2009), copyright by AAS

Similar work on Fe vii, Fe viii and Fe ix has been carried out by Young (2009), Landi and Young (2009) and Young and Landi (2009). Most of these identifications have been included in the CHIANTI version 7.1 and 8 database, and later confirmed by several analyses of solar and laboratory spectra. Many of the new identifications in the EUV have been possible thanks to the accuracy and spectral resolution of the Hinode EIS spectrometer. They have also been greatly aided by the ability to spatially resolve spectra of very different sources. By simply comparing monochromatic images of unidentified lines with those of well-known lines, it is straightforward to know the formation temperature of a line. Figure 43 shows the Fe ix lines identified by Young (2009).

We now briefly mention some of the most relevant studies, broadly dividing them by wavelengths.

**Soft X-rays**

Perhaps the most famous identifications in the XUV are those based on Edlén’s pioneering (and to date best) soft X-ray spectra in the 1930s, dominated by $n=4 \rightarrow n=3$ transitions in highly ionised ions from iron and iron-peak elements. Among these, the most famous ones are those of Fe x, where Edlén identified several transitions (Edlén 1937). These identifications established the splitting of the $^2\hbox {P}$ within the ground configuration. This allowed Edlén, in a seminal paper for solar physics (Edlén 1942), following a suggestion from Grotrian (1939), to identify the famous bright red coronal forbidden line at 6374.6 Å as the Fe x transition $^2\hbox {P}_{3/2}{-}^2\hbox {P}_{1/2}$ within the ground configuration (see Swings 1943). The forbidden line had been observed for seventy years during total solar eclipses, and it was with this identification that it was realised that the solar corona is a million degree plasma.

Edlén had also identified several lines from other ions in the soft X-rays, from iron and several other elements, so he later extended similar identifications for a large number of ions in the visible. Most of his identifications in the soft X-rays were correct, but some identifications of forbidden lines had to be revised several times, sometimes by Edlén himself. Various other researchers at Lund and other institutes continued Edlén’s work. He developed simple methods to check the identifications and suggest new ones by studying ions along isoelectronic sequences. Even today, many energies remain unknown experimentally but are relatively well known thanks to such analyses at Lund.

**Fig. 44:**
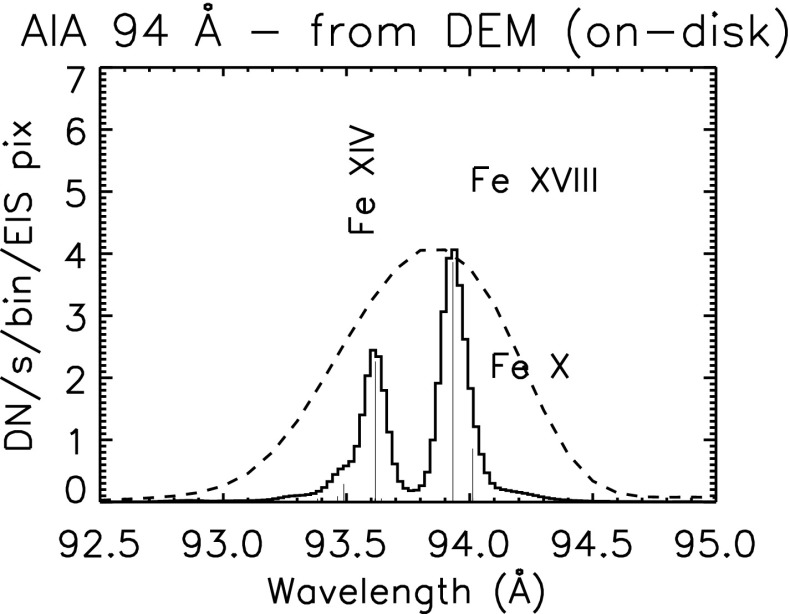
AIA 94 Å count rates for the core of an active region, as obtained with a DEM analysis using Hinode EIS observation. The dashed curve is the normalised AIA effective area Reproduced with permission from Del Zanna (2013a)

Edlén’s work in the soft X-rays was extended to the coronal iron ions ($3{s}^{2}\,3{p}^{2}\,4l, l=s,p,d,f$ levels) by the fundamental laboratory work of Fawcett et al. (1972). However, in both cases, many but not all of the observed lines with strong oscillator strengths were identified. It turns out that some of the strongest $n=4 \rightarrow n=3$ transitions in solar spectra produced by the coronal iron ions were actually not identified by Edlén or Fawcett, partly because they were not very strong in the higher density laboratory spectra. Their identification was provided by Del Zanna (2012a). This was possible thanks to large-scale scattering calculations within the APAP network and significant benchmark work using solar spectra and the original laboratory glass photographic plates from Fawcett. Some of the Fawcett et al. (1972) identifications have been revised, in particular that of the Fe xiv $3{s}^2\,3d\,^2\hbox {D}_{3/2}{-}3{s}^2\,4p\,^2\hbox {P}_{1/2}$ transition with the 93.61 Å line, which turns out to be the strongest contribution to the SDO AIA 94 Å band in the cores of active regions (whenever Fe xviii is not present), as discussed in Del Zanna (2013a) and shown in Fig. 44.

Further studies on the lower charge states of iron, from Fe vii to Fe ix were obtained with laboratory spectra from an EBIT by Lepson et al. (2002).

The soft X-ray are also rich in iron lines produced during solar flares. Such lines, from highly-charged Fe ions of the type $2{s}^2\,2{p}^k{-}2{s}\,2{p}^{k-1}$, fall around 94–135 Å and have been identified mostly using laboratory spectra of laser plasma (e.g., Fe xviii: Boiko et al. 1970; Fe xix: Feldman et al. 1973). Kastner et al. (1974a) reported the first solar-flare spectra containing the $n=2 \rightarrow 2$ L-shell iron emission, in the 66–171 Å range from OSO-V. Lawson and Peacock (1980, 1984) reviewed observations and diagnostics of $n=2 \rightarrow n=2$ transitions.

**X-rays**

Within the X-rays, the strongest lines emitted by the Sun (actually active regions) during quiet conditions are lines from Fe xvii around 15 Å. The identification of Fe xvii lines started with the excellent work of Tyrén (1938).

A significant effort in the identification of flare lines, from the X-rays to the soft X-rays was carried out by several groups, using both solar observations and high-power lasers. Most notably, G. Doschek and U. Feldman at NRL, USA, B. Fawcett at RAL, and various researchers in the USSR such as E. Kononov at the Institute for Spectroscopy, and others at the Lebedev Institute.

Transitions from highly-charged Fe ions of the type $2{p}^k{-}2{p}^{k-1}\,3l$ are as strong as the lines from H- and He-like ions, and fall around 10–20 Å.

The first observations of $n=3 \rightarrow 2$ Fe lines in solar flares were made with the OSO-III satellite in the 1.3–20 Å region, OSO-V in the 6–25 Å region and were reported by Neupert et al. (1967, 1973). Soon, similar spectra were obtained by other groups (see the review of these early observations by Doschek 1972). Several identifications were suggested by Neupert and colleagues. Further firm identifications came from the NRL group, see e.g., Doschek et al. (1972, 1973) and Feldman et al. (1973)

Laser spectra were also published in a series of papers by Boiko and colleagues (see Boiko et al. 1978a and references therein). Boiko et al. (1978a) spectral accuracy and resolution ($\simeq 0.002$ Å on average) were excellent. Very good laser spectra were also obtained at RAL (Bromage et al. 1977b, c) and were used to identify lines. The literature is too extensive to be summarised here. A good review is provided by Shirai et al. (2000), one of the NIST compilations.

**EUV**

In the early 1960s, B. C. Fawcett and collaborators established the identifications of the majority of the strongest EUV lines (from iron), with a large number of publications. The breakthrough came when laboratory spectra showed that highly-charged iron lines were the strongest lines in the EUV spectra of the Sun in the 150–300 Å spectral region (Fawcett et al. 1963; Fawcett and Gabriel 1965), which led to the first identifications (Gabriel and Fawcett 1965; Gabriel et al. 1966; Fawcett and Gabriel 1966; Fawcett et al. 1967).

Significant studies in the EUV are those of Svensson et al. (1974) on Fe ix; those of Smitt (1977) and Bromage et al. (1977a) on Fe x and Fe xi; Fawcett (1971a) on Fe xi, Fe xii, Fe xiii, Fe xiv, and Fe xv; Bromage et al. (1978) on Fe xii and Fe xiii; Churilov et al. (1985) and Cowan and Widing (1973) on Fe xv. Many Fe xvii EUV lines were identified by Jupen (1984), with further significant revisions by Feldman et al. (1985) and Del Zanna and Ishikawa (2009). Many high-temperature iron lines in the EUV have been identified using flare spectra obtained with the NRL slitless spectrometer (see, e.g., Sandlin et al. 1976; Dere 1978b).

In terms of laboratory spectra, the best measurements in the EUV have recently been carried out with electron beam ion traps (EBIT), see for example Beiersdorfer and Lepson (2012) and Beiersdorfer et al. (2014). These studies have confirmed the accuracy of the atomic data in the CHIANTI database, which are now more up-to-date than those in the NIST database. The above-mentioned identification papers also revised previous assessments in a few cases. All the new or revised identifications have so far been confirmed, with one notable exception of an Fe xii line (Beiersdorfer et al. 2014). The CHIANTI v.8 atomic data for the Fe xiii, Fe xiv, and Fe xv in terms of measurements of electron densities have recently been checked with high-density EBIT spectra around $10^{13}\,\hbox {cm}^{-3}$ by Weller et al. (2018). Very good agreement (to within about 30%) between observed and predicted line intensities was found.

In a series of studies, the Livermore EBIT was used to study the emission of the most abundant elements in the SDO AIA EUV wavelength channels, which are, with one exception, dominated by iron lines: 131 Å (Träbert et al. 2014b), 193 Å (Träbert et al. 2014a), and 304 Å (Träbert et al. 2016).

Beam-foil spectroscopy has also been fundamental to identify spectral lines (mostly in the EUV) from levels with long lifetimes, see e.g., the reviews by Träbert (1998, 2005).

**UV**

In the UV, several forbidden lines from the coronal iron ions are present. Notable identifications are those by Mason and Nussbaumer (1977) on Fe x, Sandlin et al. (1977) on Fe ix, Fe xi, Fe xii; Sandlin and Tousey (1979) on Fe x, Fe xi, and Burton et al. (1967) on Fe xii.

#### Benchmarking atomic data using line intensities

Comparisons between observed and predicted line intensities can be found throughout the literature. However, the comparisons have normally been limited to a few line ratios within an ion, or to a small wavelength range. Until recently, either the instrument calibrations or the atomic data were uncertain, so comparisons between observed and predicted line intensities were not very satisfactory, especially for the complex iron ions.

A comprehensive approach, whereby the intensities of all the strongest lines within an ion are compared to all available astrophysical and laboratory spectra has been carried out by one of us (GDZ). The work started with the coronal iron ions, in a series of benchmark papers following Fe x (Del Zanna et al. 2004a). The intensities of all the lines within an ion were compared using the emissivity ratio method, which often pointed to problems in either the radiometric calibration, the atomic data (either in the scattering calculations or in the line identification) or sometimes indicated that an observed line was blended.

Another approach is to compare predicted and observed line intensities within a spectral range. A few of such studies have been carried out. Below we mention a few examples of benchmarking studies, with particular emphasis on the iron ions, because of their diagnostic importance in the X-rays and EUV.

**X-rays**

**Fig. 45:**
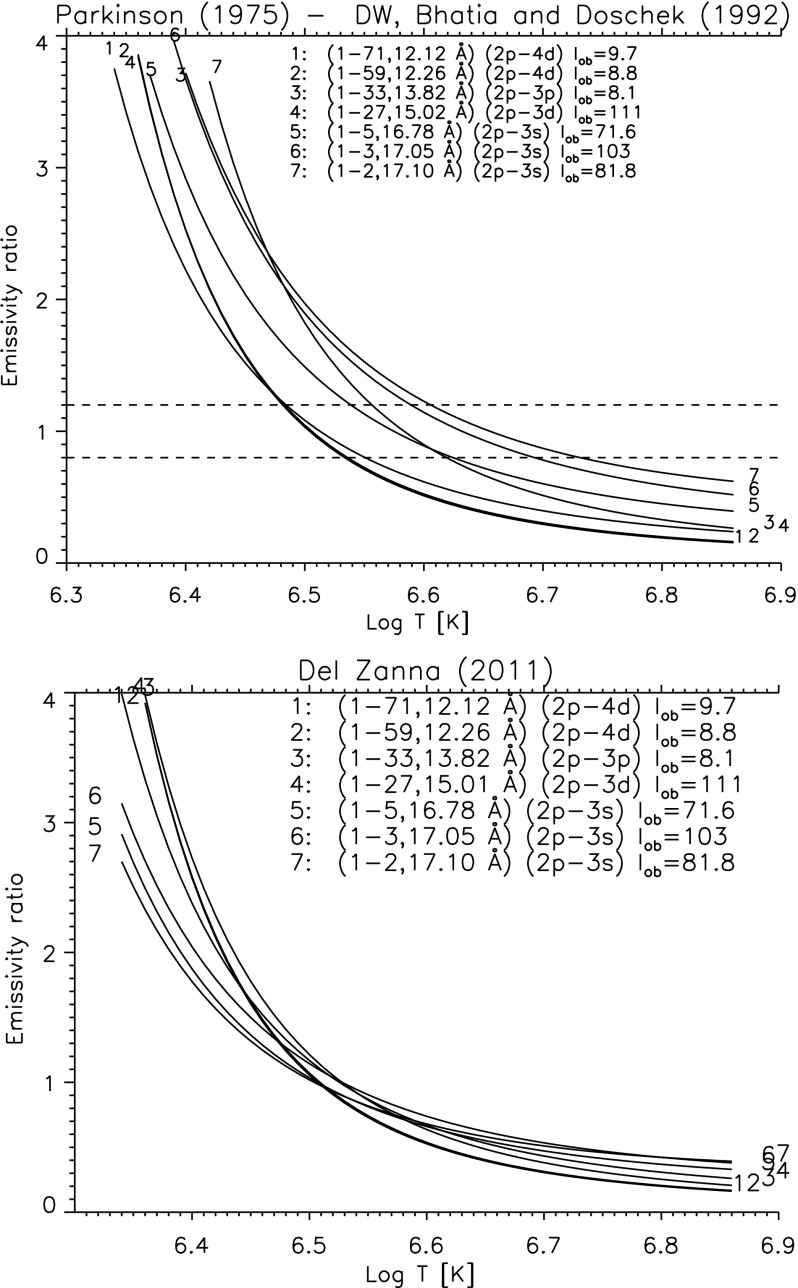
Emissivity ratio curves for the strongest Fe xvii lines, using the active region core observations of Parkinson (1975). Top: with the DW scattering calculations of Bhatia and Doschek (1992). Bottom: with the *R* matrix calculations of Liang and Badnell (2010) Figures adapted from Del Zanna (2011b)

Within the X-rays, perhaps the most important benchmark studies have been those on Fe xvii (Del Zanna 2011b), which produces the strongest X-ray lines in active regions, and of Fe xviii (Del Zanna 2006a), which produces strong lines in more active conditions. For both these complex ions, resonance effects are very important. Therefore, earlier DW scattering calculations provided poor agreement between theory and observations, with discrepancies of factors of 2–3. Such discrepancies have led to a large number of papers where possible explanations for such deviations were sought, especially for the Fe xvii case. We do not review those studies here as it turned out that the more recent *R*-matrix calculations resolved all the main discrepancies. Figure 45 shows as an example the differences for Fe xvii between one set of DW calculations and one set of *R*-matrix calculations. Similar results were obtained for Fe xviii.

Other benchmarking studies in the X-rays were carried out on Fe xxiii (Del Zanna et al. 2005) and Fe xxiv (Del Zanna 2006b). A more complete benchmark of the X-rays was carried out by Landi and Phillips (2005). They reviewed older SMM FCS observations of two solar flares, using CHIANTI atomic data. A few line identifications and blends were revised. The spectra had excellent resolution, but were limited by the fact that spectral lines at different wavelengths were not observed simultaneously.

**Soft X-rays**

**Fig. 46:**
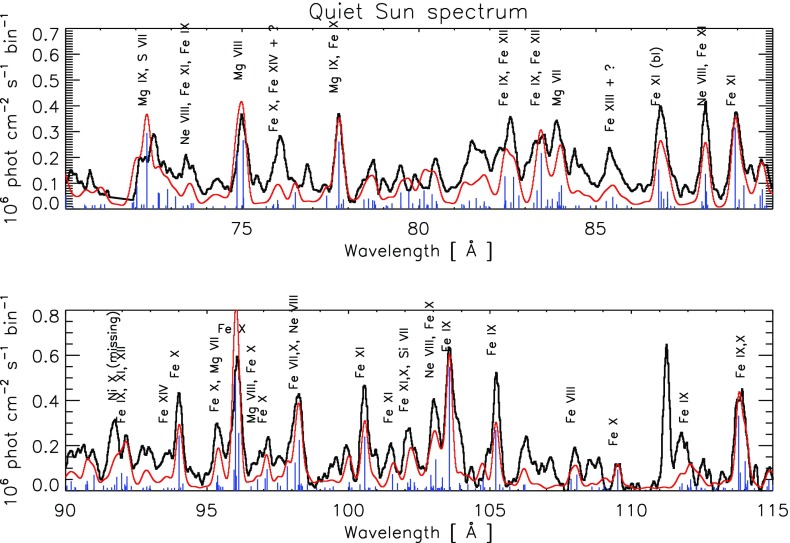
The quiet Sun soft X-ray spectrum (black) obtained from the Manson (1972) paper, with superimposed a predicted one, based on the atomic data which were subsequently made available within version 8 of the CHIANTI database Adapted from Del Zanna (2012a)

As already mentioned, little experimental and theoretical data has been available to study the soft X-rays. The high-temperature 2–2 transitions from Fe xix, Fe xx, Fe xxi, and Fe xxii were benchmarked against SDO EVE spectra of solar flares by Del Zanna and Woods (2013). Del Zanna (2012a) used the recent calculations of the coronal iron ions and benchmarked them, using the few high-resolution solar soft X-ray spectra available. Relative good agreement was found, although it is clear that a significant number of lines still needs to be identified, and atomic data are still missing, as shown in Fig. 46.

**EUV**

**Fig. 47:**
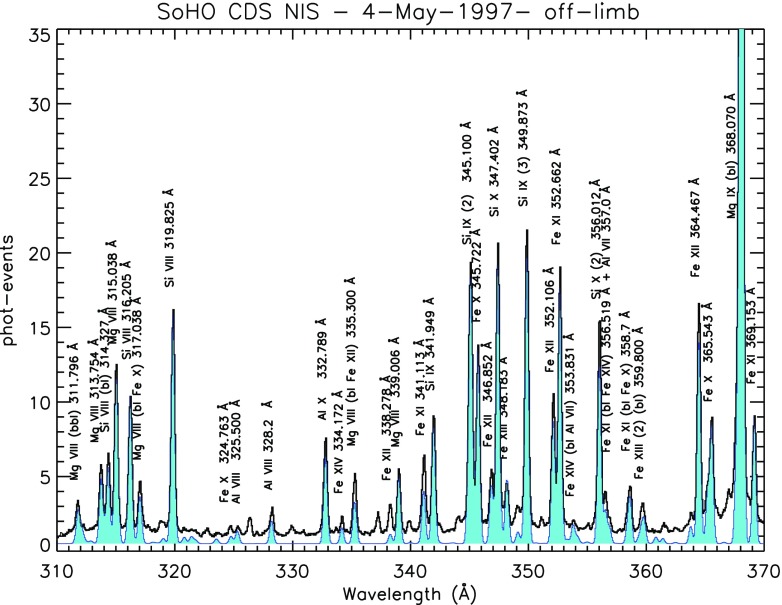
SoHO CDS NIS 1 averaged spectra of the quiet Sun, with the simulated spectra obtained with CHIANTI v.1 shown with shaded blue areas Adapted from Del Zanna (1999)

Within the EUV, a significant benchmark study based on the first SERTS rocket flight (Thomas and Neupert 1994) was provided by Young et al. (1998). Relatively good agreement between theory and observations was found. Other benchmark studies carried out using a wide range of SoHO CDS NIS and GIS observations also showed relatively good agreement (within 30–50%) between observation and theory (Del Zanna 1999), especially above 300 Å. Figure 47 shows an example of CDS NIS 1 spectra with superimposed simulated spectra obtained with CHIANTI version 1. The coronal iron lines, as well as the others from Si, Mg are quite well reproduced, although the recent large-scale calculations for the iron ions have further improved agreement.

Landi et al. (2002a) also performed a benchmark of CHIANTI data for the coronal lines using off-limb observations from SOHO CDS NIS. Some uncertainties associated with the instrument calibration were present.

With the launch of Hinode in 2006, the superior resolution and sensitivity of the EIS spectrometer indicated several problems in the 170–300 Å range, with about half of the lines not firmly identified, and discrepancies of large factors (up to 2) for several lines, including some of the strongest ones, from Fe xi. All the main lines are from the complex coronal iron ions. A long-term benchmarking programme was initiated by one of us (GDZ). It involved recalculating atomic data and reassessing line identifications using both laboratory and astrophysical spectra. It took several years to complete the work on the most complex ions. Most of the unknown levels (which were about half for the lower configurations of Fe x, Fe xi, and Fe xii) have been identified in a series of papers: Fe vii (Del Zanna 2009a), Fe viii (Del Zanna 2009b), Fe ix (Del Zanna 2009a), Fe x (Del Zanna et al. 2004a), Fe xi (Del Zanna 2010), Fe xii (Del Zanna and Mason 2005a), Fe xiii (Del Zanna 2011a), Fe xvii (Del Zanna and Ishikawa 2009). These benchmark studies identified a number of problems, some of which were in the atomic data and have been resolved with the latest calculations on these ions, where further benchmarking was also carried out.

The identifications of lines in the Hinode/EIS wavelengths proposed by Del Zanna et al. (2004a) have been confirmed in Del Zanna (2012b). Del Zanna et al. (2010b) scattering calculation, obtained with an optimal target, finally produced improved agreement and allowed the identifications of all the strongest transitions (Del Zanna 2010).

**Fig. 48:**
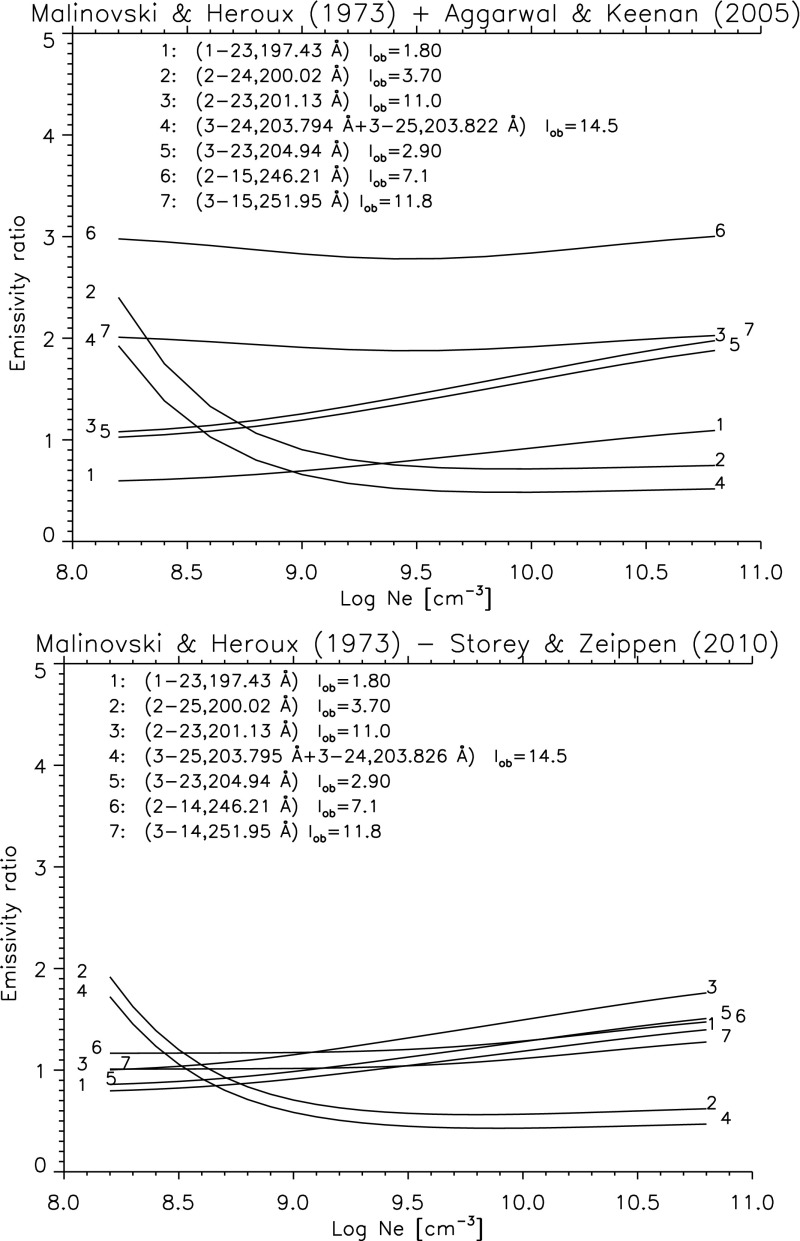
Emissivity ratio plots of the Fe xiii EUV lines reported by Malinovsky and Heroux (1973). Top: with the Aggarwal and Keenan (2005) atomic data. Bottom: with the Storey and Zeippen (2010) atomic data. Note that the curves should cross if the plasma is isothermal Image adapted from Del Zanna (2011a)

One example is Fig. 48, where the well-calibrated EUV observations of the Fe xiii reported by Malinovsky and Heroux (1973) were benchmarked against two *R*-matrix calculations. It is clear that the Storey and Zeippen (2010) calculations are in good agreement with observations, while those of Aggarwal and Keenan (2005) have several problems, with discrepancies of factors of 2–3. Similar problems have been found in other ions.

Further benchmark studies not on single ions but on full Hinode EIS spectra were those of Del Zanna (2009a) and Landi and Young (2009) where CHIANTI atomic data were benchmarked for cooler transition-region lines. Del Zanna (2012b) benchmarked the available atomic data for coronal lines observed by Hinode EIS off-limb spectra of the quiet Sun and an active region.

**EUV/UV**

At wavelengths longer than 600 Å, excellent spectral data have been obtained by SoHO SUMER. As previously mentioned, there are several line lists, but comparatively few benchmark studies of the atomic data. A comprehensive study of the coronal lines observed off-limb by SOHO SUMER was carried out by Landi et al. (2002b), using CHIANTI atomic data and the EM loci method. Relatively good agreement was generally found.

Doschek et al. (1999) carried out a systematic study of the cooler (transition-region) lines observed on-disk by SUMER. Observed line ratios were compared to those predicted with CHIANTI and with other atomic data. Significant discrepancies were found for many ions. We recall however that only lines within a 40 Å band were observed simultaneously, so despite averaging some of the discrepancies could be due to real temporal variability. Transition-region lines are in fact well-known to have a strong variability.

#### Rest wavelengths

The issue of rest wavelengths is a non-trivial one. In the X-rays (1–50 Å), the best measurements have normally been obtained from laboratory plasma (cf. Boiko et al. 1978a), with a typical accuracy around 10 Å of 0.002 Å. High-resolution crystal spectrometers (cf. SMM/FCS, Phillips et al. 1982) have achieved similar accuracies.

However, laboratory wavelengths are often not accurate enough in the EUV/UV, or are at odds with solar measurements. Databases such as NIST mostly rely on laboratory measurements and are therefore sometimes inaccurate.

The most effective way to obtain rest wavelengths is to measure them directly with high-resolution spectroscopy of the Sun. This brings in a series of problems, given that, depending on the temperature of formation of the lines and the source, significant Doppler motions are normally present.

It is now well established that lines formed around 1 MK show small Doppler motions (see, e.g., Peter and Judge 1999; Del Zanna 2008c). These lines mostly emit at soft X-ray and EUV wavelengths. The most accurate wavelengths for these lines come from full-Sun spectra obtained by two rocket flights with a spectrograph built at the Goddard Space Flight Center. The first rocket flight was on 1969 May 16. On that day, the Sun was moderately active, (the F10.7 radio flux was 159.4). The instrument observed the 60–385 Å region with high resolution (0.06 Å) (Behring et al. 1972). A second flight, on 1973 September 21, observed the 160–770 Å region (Behring et al. 1976). A few of these coronal measurements have been revised with high-resolution EUV spectra from Hinode EIS (cf. Del Zanna 2012b) and the SERTS rocket flights (cf. Thomas and Neupert 1994).

The issue of the EUV lines formed in the 1–3 MK range is more complex. Most of these lines are typically emitted by active regions. The hot core loops typically show small Doppler shifts, even at their footpoints (near sunspots and plage regions, where the ‘moss’ is, see e.g., Winebarger et al. 2013). However, there are large spatial areas where significant blue-shifts, increasing with the formation temperature of the lines, are present (Del Zanna 2008c; Doschek et al. 2008).

Lines formed at higher temperatures (above 3 MK) are normally associated with very dynamic events such as microflares and solar flares, and typically show strong Doppler motions. It is only during the gradual phase in the post-flare loops that the wavelengths of the flare lines normally become at rest.

On the other hand, it is well established that lower-temperature EUV lines are red-shifted, following various trends depending on the source (Peter and Judge 1999; Del Zanna 2008c). Accurate rest wavelengths can still be established, see e.g., Hassler et al. (1999) for a revision of the Ne viii wavelengths using SUMER, and Del Zanna (2009a) for wavelengths of cool lines observed by Hinode EIS.

In the UV, the best wavelength measurements have been obtained by Sandlin et al. (1986) with a careful analysis and averaging of Skylab limb observations (where it is assumed that lines should be at rest), as well as HRTS data. These measurements have an accuracy of about 0.005 Å, and agree, within uncertainties, with the best laboratory measurements, for the few ions we have checked.

## Non-equilibrium effects

Several non-equilibrium effects can significantly modify XUV spectra. In this section, we provide a brief overview of some of these effects. For a fuller recent review on this topic see Dudík et al. (2017a).

### Time-dependent ionisation

We recall (Eq. 43) that the charge state distribution of an element can be calculated:

$$\begin{aligned} {1 \over N_e} {{ dN}_{r}\over dt} =N_{r-1} S^e_{r-1} - N_{r} (S^e_{r} +\alpha _{r}) + N_{r+1}\alpha _{r+1} \end{aligned}$$

once the total ionisation and recombination rates are known, and a model for the plasma flow is assumed.

In the low solar corona, the timescales for $N_{r}$ to ionise ($1/(N_e S^e_{r})$) or to recombine ($1/(N_e \alpha _{r})$) are of the order of 100 s, and ionisation equilibrium is a reasonable approximation. However, in regions where strong flows and strong temperature gradients are present, significant departures from equilibrium could occur. In this section we briefly summarize a few cases, to provide the reader with an idea of where departures from ionization equilibrium are expected and what kind of effects they could have on the plasma diagnostics.

A classical example is the solar transition region, where downflows are ubiquitous, so the ions are moving into regions with much reduced temperatures (there is a steep gradient). A simple semi-empirical model for the quiet Sun transition region (e.g., Gabriel 1976) shows gradients of about 2000 K/km. Electron number densities in the transition region are of the order of $10^{10}\,\hbox {cm}^{-3}$, and the timescales for ionisation and recombination are of the order of tens of seconds, so any flow of a few km/s would have a significant effect. The main effect would be to shift the formation temperature of a line to much lower temperatures, compared to those expected if ionization equilibrium conditions. Observationally, variations of line intensities on timescales faster than ionisation/recombination times are common, so one would indeed expect that time-dependent ionisation has a major effect in the transition region.

In the literature, there are several early studies of the effects that departures from ionization equilibrium could have on the various diagnostics. For example, Raymond and Dupree (1978) showed that a 5 km/s downflow would have a significant effect on the line intensities of C iii, in particular on the electron densities estimated from line ratios of this ion. Significant effects were later confirmed with subsequent 1-D hydrodynamical modelling of loop structures by, e.g., Noci et al. (1989), Raymond (1990) and Hansteen (1993), although a complete self-consistent treatment is still lacking. An improvement in the modelling was introduced within the HYDRAD code (Bradshaw and Mason 2003), where the radiative losses are calculated self-consistently using the time-dependent ion populations. HYDRAD was used in Bradshaw et al. (2004) to model a small compact flare. An 80 Mm loop was heated by a 300 s heating pulse at the apex, raising its temperature from 1.6 MK to about 7 MK. Strong enhancements of He I, He II, and C IV line intensities were found during the impulsive phase, while the hot lines such as Fe XVIII, Fe XIX we significantly suppressed. Bradshaw et al. (2004) also noted that the enhancements could perhaps indicate a viable way to explain the anomalously high intensities of lines from these ions, which have long recombination times (see Sect. 7.3).

Several further studies of non-equilibrium ionization in transition-region lines, particularly important for the IRIS mission, have reached similar conclusions. For example, Doyle et al. (2013) applied the GCR technique to study the response of Si iv and O iv lines to short heating bursts. They found that the Si iv intensity is significantly enhanced (factor of three or more) shortly after the burst, while the O iv lines showed little variation.

Martínez-Sykora et al. (2016) ran MHD simulations using the Bifrost code (Gudiksen et al. 2011) with the inclusion of non-equilibrium ionization as described by Olluri et al. (2013b), using atomic data included in the DIPER package (Judge and Meisner 1994). They also found significant increases of Si iv relative to O iv in active regions, and although were not able to fully reproduce the IRIS observations, confirming that non-equilibrium ionization is an important effect which brings closer agreement between observations and theory.

Olluri et al. (2013a) used the same codes to study the effects that non-equilibrium ionization have on the O IV density diagnostics. They found that significant differences in the derived densities occur, because the O IV abundance is shifted to lower temperatures.

Flows in the transition region can not only affect the measurements of electron densities, but also those of relative elemental abundances. For example, Edgar and Esser (2000) showed that a significant enhancement of Mg vi emission compared to Ne vi is present, if non-equilibrium effects in a recombining downflow are considered. This would significantly affect the FIP effect measurements in the transition region (see Sect. 14). We should note, however, that Edgar and Esser (2000) considered cooling at constant pressure or at constant density. The formation mechanism producing the transition region emission is still not clear, as with the general coronal heating. However, there is some evidence that the plasma in the legs of coronal loops (where Mg vi and Ne vi are emitted) is radiatively cooling, i.e., where the heating has been switched off and the dominant cooling process is by radiation losses. The footpoints of active region 1 MK loops show ubiquitous redshifts in transition-region lines of 10–30 km/s (see, e.g., Del Zanna 2008c). The observed downflows and densities are consistent with the radiative losses sustained by an enthalpy flux while the loops radiatively cool (Bradshaw 2008). In the case of radiative cooling, $T \sim N_{\mathrm{e}}^2$ (Bradshaw and Cargill 2005), which is quite different to the cases considered by Edgar and Esser (2000).

Time-dependent ionisation can also increase the cooling times of coronal loops, as discussed e.g., in Bradshaw and Mason (2003). Also, it was found to have a significant effect in the low corona after interchange reconnection occurred in the high corona: Bradshaw et al. (2011) studied the hydrodynamic response of the plasma undergoing reconnection between a high-pressure (high temperature and density) hot loop in the core of an active region and a surrounding open field with much lower pressure. Once the reconnection has taken place, a rarefaction wave would start travelling towards the chromosphere, causing a rapid expansion and cooling of the plasma. The higher-temperature lines formed around 3 MK were found to be enhanced by a factor of about two when time-dependent ionization was considered.

Stationary plasma that is heating or cooling quickly, on timescales shorter than the local ionisation and recombination timescales, will also be out of equilibrium. In the case of heating, the plasma is under-ionised since the ions do not have enough time to ionise, despite the high temperatures. In the case of cooling, the plasma is over-ionised since the ions do not have enough time to recombine, whilst in a low-temperature environment.

Plasma is most likely to be out of ionisation equilibrium during the impulsive phase of solar flares. Indeed non-equilibrium may be a more common process in the solar corona, depending on the frequency and type of heating, and the local density. For example, Bradshaw and Cargill (2006) showed that fast heating to high temperatures (10 MK) of a low-density plasma would be considerably out of equilibrium, so there would be very little emission (if any) from the ions that are normally formed (in equilibrium) at 10 MK. The same conclusions were reached by Reale and Orlando (2008), and could explain the fact that very little emission is observed at high temperatures in active regions, despite the fact that some nanoflare modeling predicts that high temperature plasma should be present.

At the end of the cooling phase of a flare the plasma undergoes a thermal instability, which also involves strong non-equilibrium effects, as described e.g., by Reale and Orlando (2008).

Signatures of non-equilibrium in X-ray spectra during solar flares have long been sought by several authors. As we have discussed in Sect. 4, the best diagnostic to assess for departures from ionisation equilibrium is the *q* / *w* ratio of the inner-shell versus the resonance in He-like ions, which depends on the relative ion abundance between the Li-like and the He-like ion. SOLFLEX observations of this ratio for the He-like Fe and Ca are shown in Fig. 97 as an example. If one uses the temperatures obtained from the *j* / *w* and *k* / *w* ratios (see Sect. 4) to calculate the *q* / *w* ratio, one often finds that the ratio of the Li-like versus the He-like ion abundance is much higher, compared to what it should be in ionisation equilibrium. This is because of the significant intensity of the *q* line. The plasma therefore appears to be in transient ionisation. This result was obtained by Doschek et al. (1979, 1980), Doschek and Feldman (1981) and Feldman et al. (1980) using SOLFLEX observations. However, given the high densities observed during the peak phase of a flare, typical equilibration times should be of the order of a second or less, so it is hard to accept that the plasma is out of equilibrium. Indeed the authors suggest that there might be problems in the atomic data.

Later observations were obtained with other instruments, most notably with the SMM (see, e.g., Gabriel et al. 1983; Antonucci et al. 1984, 1987) and Hinotori spectrometers, but considering the various uncertainties, it was not very clear if departures from ionisation equilibrium were present or not. See the discussion by Doschek and Tanaka (1987) in their review of SMM and Hinotori X-ray observations.

In the high corona, we have the opposite situation, with the plasma flowing out into the heliosphere at progressively faster speeds, also with a significant geometrical expansion. At some point, local electron densities become so low that the ionisation/recombination timescales become much longer than the expansion timescales, so the ion populations do not change anymore (‘are frozen-in’). Typical solar wind models show that the region where this occurs is within a few solar radii from the Sun (see, e.g., Esser et al. 1998 and references therein).

### Non-Maxwellian electron distributions

A central assumption for most of the spectroscopic diagnostics applied to the solar corona is that the electrons have a Maxwellian distribution. The classical textbook plasma physics shows that the relaxation time for e–e collisions in the low corona is $\tau _{ee}\simeq 0.01~{T}^{3/2}~{N}_{e}^{-1}~\mathrm{s}$, i.e., $\simeq 0.01\,\mathrm{s}$ for an active region loop ($T=10^6\,\hbox {K}$ and $N_e=10^9\,\hbox {cm}^{-3}$), but $\simeq 300\,\hbox {s}$ for a plasma suddenly heated at $T=10^7\,\hbox {K}$ (with $N_e=10^8\,\hbox {cm}^{-3}$). After a time longer by a factor $(m_i/m_e)^{1/2} \simeq 43$, the ions also become thermal, and after the same time factor, both species are thermalised, and the electron and ion temperatures are the same: $T\equiv T_{e}=T_{i}$. Therefore, as in the non-ionization equilibrium case, there could be cases in the solar corona where the electrons have non-Maxwellian distributions.

This topic has been studied by a number of authors. Theoretical calculations based on the Fokker–Planck (sometimes also called Vlasov–Landau) equation have shown that a high-velocity tail of the electron distribution can form in some circumstances. Shoub (1983) solved the Landau Fokker–Planck equation to model the transition region, and found that significant deviations from the local Maxwellian approximation can form. This was later confirmed by Ljepojevic and Burgess (1990) where solutions to the equation in cases where large temperature and density gradients are present (as in the TR) were discussed in detail. Physically, the bulk of the electrons is found to follow the Spitzer–Harm solution, i.e., Maxwellian, while high-velocity tails of the distribution form and remain present where strong temperature gradients are present. This is because the mean free path of an electron is $\sim v^4$ so the high-velocity electrons from the hotter region stream down towards the chromosphere almost freely. Towards the chromosphere, the gradients decrease substantially, and the high-velocity tail should disappear.

Ljepojevic and MacNeice (1988) simulated a flaring coronal loop and also found for this case an enhancement of the tail population for electrons moving down the temperature gradient. Ljepojevic and MacNeice (1989) used the Landau–Fokker–Planck equation to show that the classical Spitzer–Harm approximation for the heat flux completely breaks down in the presence of non-Maxwellian electrons, with fundamental consequences for the energy balance.

Below we focus on few diagnostic applications and observational aspects.

#### Modelling the XUV spectra

The presence of a non-Maxwellian distribution affects the excitation and de-excitation rates within an ion and the ionisation and recombination rates. A complete self-consistent treatment of all the rates is still not available, although significant advances have been made in recent years.

Regarding the excitation and de-excitation collision rates, a simple way to include the effects of a non-Maxwellian distribution is to model the distribution as a superposition of Maxwellian distributions with different temperatures. The non-Maxwellian collision rates can then be calculated as a linear combination of the Maxwellian ones. Such an approach was included in the CHIANTI database version 5 (Landi et al. 2006), and proposed again by Hahn and Savin (2015) in terms of combinations that would reproduce a $\kappa$-distribution.

In order to obtain excitation rates integrated over non-Maxwellian velocities, one requires the collisional cross sections. Since these are rarely available, a way to recover the behaviour of the collision cross sections as a function of energy from the rates via a parametrisation method was developed and implemented by Dzifčáková et al. (2015) using CHIANTI version 7.1 data, and released as a separate package, KAPPA.^4^ This method typically provides cross sections sufficiently accurate, within a few percent (see, e.g., Dzifčáková and Mason 2008; Dzifčáková et al. 2015).

Of course, whenever the original cross sections are available, it is better to use them directly for the calculations of the rates. The UK APAP team is building a database of such cross-sections.

It is worth noting that, for a distribution that is not Maxwellian, the effective collision strengths for the excitation and de-excitation are not the same. In the case of $\kappa$-distributions, the $\varUpsilon _{ij}(T,\kappa )$ for the excitation and 
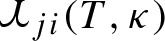
 are (see Bryans 2006; Dudík et al. 2014b):

84 $$\begin{aligned} \varUpsilon _{ij}(T,\kappa ) = A_\kappa \mathrm{exp}\left( \frac{E_{ij}}{k_{\mathrm{B}}T}\right) \int \limits _0^{+\infty } \frac{\varOmega _{ji}(E^{\prime })}{\left( 1+ \frac{E^{\prime } +E_{ij}}{(\kappa -3/2)k_{\mathrm{B}}T}\right) ^{\kappa +1}} \,\mathrm{d}\left( \frac{E^{\prime }}{k_{\mathrm{B}}T}\right) , \end{aligned}$$

85Once these rates are calculated, they can be used directly instead of the thermally-averaged cross-sections.

Regarding the ionization and recombination rates, similar issues arise. The calculations are again straightforward if the actual cross-sections are available. In the case of ionization by direct impact by electrons, parametrised versions of the ionization cross-sections have been stored in the CHIANTI database version 6 (Dere et al. 2009). They have been used by Dzifčáková and Dudík (2013) to calculate ionization rates for $\kappa$ distributions.

For the radiative recombination, Dzifčáková (1992) provided a method to estimate the rates from the usual parametrised formulas which describe the Maxwellian rates.

Regarding the DR rates, a similar approach was developed for the $\kappa$-distributions by Dzifčáková (1992) and Dzifčáková and Dudík (2013). The total DR rate for an ion is fitted with the formula:

86 $$\begin{aligned} \alpha ^{\mathrm{d}}(T) = T^{-3/2} \sum _i c_i \exp (-E_i/T) \ {\mathrm{cm}}^{3}\,\mathrm{s}^{-1} \end{aligned}$$

where the $c_i$ and $E_i$ are the fit parameters. The dielectronic recombination rate can then be calculated with

87 $$\begin{aligned} \alpha ^{\mathrm{d}}(T) = \frac{A_\kappa }{T^{3/2}} \sum _i \frac{c_i}{\left( 1 +\frac{E_i}{(\kappa -3/2)T}\right) ^{\kappa +1}} \ {\mathrm{cm}}^{3}\,\mathrm{s}^{-1} \end{aligned}$$

**Fig. 49:**
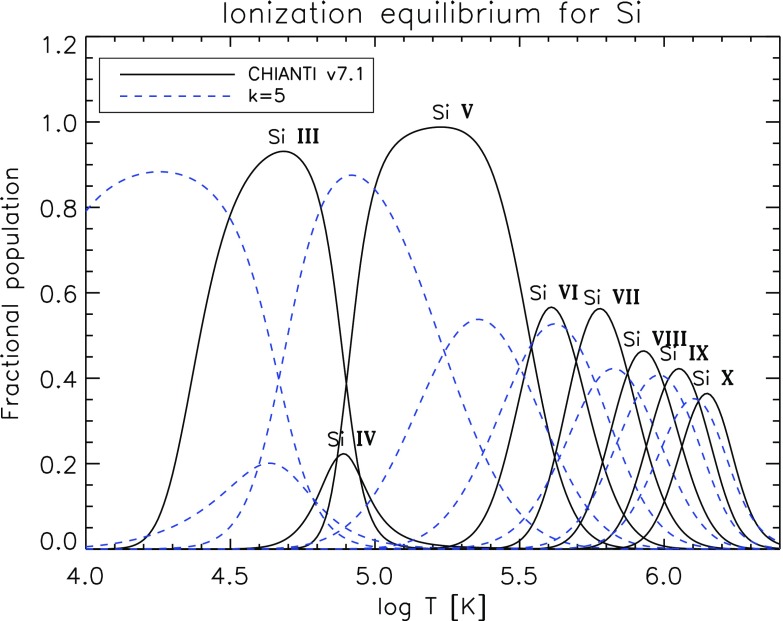
Fractional population of Si ions in the Maxwellian case (full line) and a strong non-thermal distribution with $\kappa =5$ (dashed line). The figure was obtained from the KAPPA package

Once all the rates are calculated, it is straightforward to calculate ion charge state distributions in equilibrium. A tabulation of values calculated with a $\kappa$-distribution can be found in Dzifčáková and Dudík (2013). An example is shown in Fig. 49, where the distribution of silicon ions are shown, for the Maxwellian case and for a strongly non-thermal distribution with $\kappa =5$. It is worth noting the shift of the low charge states towards lower temperatures, and the increase in the range of temperatures where each ion is formed.

#### Observational constraints

**Solar flares**

Departures from a Maxwellian distribution, especially the presence of high-energy tails, are expected to arise due to magnetic reconnection or wave-particle interactions, in particular during the impulsive phase of flares. Indeed, hard X-ray (HXR) observations above a few keV routinely show non-thermal bremsstrahlung emission which (at least partly) is caused by non-thermal electrons that have traveled from the flare heating site. Strong HXR emission is found at the footpoints of flare loops, but *Yohkoh* (see, e.g., Masuda et al. 1994) and RHESSI (see, e.g., Krucker et al. 2010) observations have also shown that much weaker emission in coronal sources is often present. This indicates that non-equilibrium processes are also occurring in the solar corona during flares.

The HXR emission is caused by bremsstrahlung of the free electrons, and has traditionally been modelled with two components, a thermal one at low energies (usually with the isothermal approximation), and a non-thermal one, usually with a power law for the electron distributions, with a cutoff at lower energies. However, it has been shown that the non-thermal part of the spectrum can also be modeled with $\kappa$-distributions (see, e.g., Kašparová and Karlický 2009; Oka et al. 2013).

One issue that hard X-ray observations at lower energies cannot easily resolve is related to the multi-thermality of flare plasma. The lower energy emission from RHESSI is often approximated with a single temperature distribution, but the emission is likely to be multi-thermal, or thermal plus non-thermal, as suggested by Oka et al. (2013).

**Fig. 50:**
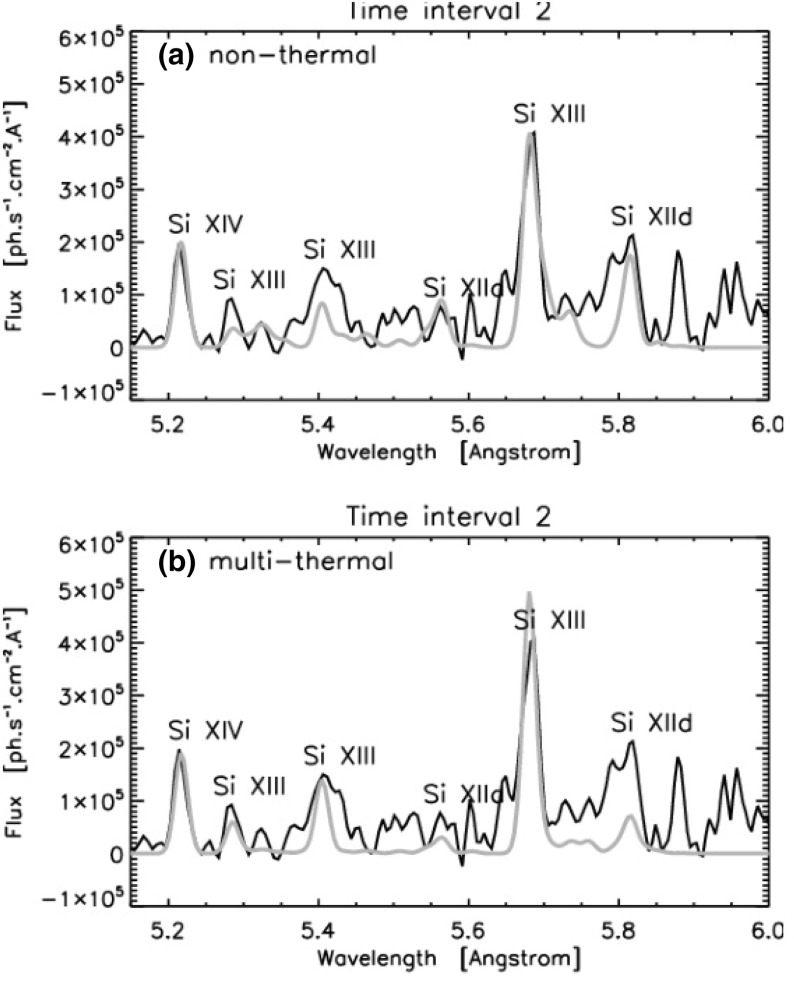
RESIK spectra of a flare, with modelled lines (grey curves) assuming a non-thermal electron distribution (top) or a multi-thermal plasma (bottom) Adapted from Dzifčáková et al. (2008)

A more direct spectroscopic method to assess if non-Maxwellian distributions exist during solar flares was suggested by Gabriel and Phillips (1979), as described previously in Sect. 4. It relies on the measurement of the dielectronic satellite lines that are observed in the wings of the Fe xxv resonance line at 1.85 Å. These satellite lines are formed by electrons dielectronically captured by Fe xxv ions, which have a very narrow energy distribution (each transition can only occur at a single energy). SOLFLEX observations of solar flares were analysed by Seely et al. (1987). The authors deblended the d13 line complex, and found some evidence, during the impulsive phase, of departures from equilibrium.

One problem with this method for solar flare diagnostics is that it requires a very high spectral resolution. In fact, the $n=3$ satellites lines are very close to the resonance line. In solar flare spectra during the impulsive phase, when one might expect departures from a Maxwellian, the satellite lines have not been fully resolved. This is because the resonance lines are always broadened.

More success has been obtained in the X-ray spectral range around 5 Å, with the satellite lines observed by the RESIK instrument during the impulsive phase of solar flares (Dzifčáková et al. 2008; Kulinová et al. 2011), although even in this case it is not trivial to assess how much a non-thermal electron distribution is present and how much the plasma is multi-thermal (see Fig. 50, Table 5).

**Table 5:** Main RESIK spectral lines

RESIK cha. no.	Ion	$\lambda$ (Å)	Transition	Log $T_{\max }$ (K)
1	Ar xvii	3.365	$1{s}^2\,{}^1\hbox {S}_{0}{-}1{s}\,3{p}\,{}^1\hbox {P}_{1}$	7.2
	Ar xvi diel.	3.428	$1{s}^{2}\,2{p}\,{}^2\hbox {P}_{3/2}{-}1{s}\,2{p}\,(^3\hbox {P})\,3{p}\,^2\hbox {D}_{5/2}$	7.1
	K xviii	3.532	$1{s}^2\,{}^1\hbox {S}_{0}{-}1{s}\,2{p}\,{}^1\hbox {P}_{1}$	7.2
	K xviii	3.571	$1{s}^2\,{}^1\hbox {S}_{0}{-}1{s}\,2{s}\,{}^3\hbox {S}_{1}$	7.2
	S xvi	3.696, 3.696	$1s\,^2\hbox {S}_{1/2}{-}5{p}\,{}^2\hbox {P}_{3/2, 1/2}$	7.2
	S xvi	3.784, 3.785	$1s\,^2\hbox {S}_{1/2}{-}4{p}\,{}^2\hbox {P}_{3/2, 1/2}$	7.2
2	Ar xvii	3.949	$1{s}^2\,{}^1\hbox {S}_{0}{-}1{s}\,2{p}\,{}^1\hbox {P}_{1}$	7.2
		3.966	$1{s}^2\,{}^1\hbox {S}_{0}{-}1{s}\,2{p}\,{}^3\hbox {P}_{2}$	7.2
		3.969	$1{s}^2\,{}^1\hbox {S}_{0}{-}1{s}\,2{p}\,{}^3\hbox {P}_{1}$	7.2
	S xv	4.089	$1{s}^2\,{}^1\hbox {S}_{0}{-}1{s}\,4{p}\,{}^1\hbox {P}_{1}$	7.2
3	S xv	4.299	$1{s}^2\,{}^1\hbox {S}_{0}{-}1{s}\,3{p}\,{}^1\hbox {P}_{1}$	7.2
	S xvi	4.727, 4.733	1s $^2\hbox {S}_{1/2}{-}2{p}\,{}^2\hbox {P}_{3/2, 1/2}$	7.2
	Si xiv	4.831, 4.831	1s $^2\hbox {S}_{1/2}{-}5{p}\,{}^2\hbox {P}_{3/2, 1/2}$	7.2
4	S xv	5.039	$1{s}^2\,{}^1\hbox {S}_{0}{-}1{s}\,2{p}\,{}^1\hbox {P}_{1}$	7.1
		5.102	$1{s}^2\,{}^1\hbox {S}_{0}{-}1{s}\,2{p}\,{}^3\hbox {S}_{1}$	7.1
	Si xiv	5.217, 5.218	1s $^2\hbox {S}_{1/2}{-}3{p}\,{}^2\hbox {P}_{3/2, 1/2}$	7.2
	Si xiii	5.286	$1{s}^2\,{}^1\hbox {S}_{0}{-}1{s}\,5{p}\,{}^1\hbox {P}_{1}$	7.1
	Si xiii	5.405	$1{s}^2\,{}^1\hbox {S}_{0}{-}1{s}\,4\hbox {p}\,{}^1\hbox {P}_{1}$	7.1
		5.408	$1{s}^2\,{}^1\hbox {S}_{0}{-}1{s}\,4{p}\,{}^3\hbox {P}_{1}$	7.1
	Si xii diel.	5.56	$1{s}^{2}\,2{p}\,{}^2\hbox {P}_{1/2, 3/2}{-}1{s}\,2{p}\,4{p}\,{}^2\hbox {D}_{3/2,5/2}$	7.0
	Si xiii	5.681	$1{s}^2\,{}^1\hbox {S}_{0}{-}1{s}\,3{p}\,{}^1\hbox {P}_{1}$	7.1
	Si xii diel.	5.816	$1{s}^{2}\,2{p}\,{}^2\hbox {P}_{1/2, 3/2}{-}1{s}\,2{p}\,(^3\hbox {P})\,3{p}\,{}^2\hbox {D}_{3/2,5/2}$	7.0

*diel.* lines formed by dielectronic recombination

Another possible diagnostic method could be high-resolution observations of the free–bound edges in the X-rays, as suggested by Dudík et al. (2012). However, observations to date have not had enough sensitivity and spectral resolution to be able to detect the presence of non-thermal electron distributions.

**Solar wind**

Another place where we know from in-situ measurements that the electron distributions are very anisotropic and non-Maxwellian is the solar wind. They can typically be modelled with $\kappa > 2.5$ distributions. For a list of references, see the recent review by Dudík et al. (2017a). Two question naturally arise: “why are there non-Maxwellian distributions?” and “do they originate in the solar corona?” If there are correlations between the particles in the system, induced by any long-range interactions in the emitting plasma such as wave-particle interactions, shocks, or particle acceleration then the distribution function may depart from the Maxwellian one. It is therefore possible that non-Maxwellian distributions originate in the corona.

We do not yet have direct in-situ measurements of the distributions functions close to the Sun to reach a conclusion. The closest observations were those based on the *Helios* spacecrafts, at 0.3 AU, although Parker Solar Probe will provide such observations. Therefore, studies of the particle distributions have so far been based on modelling. For example, Esser and Edgar (2000) allowed for the presence of non-Maxwellian electrons to try and resolve a discrepancy between the freezing-in temperatures estimated from the in-situ measurements of the fast solar wind, about 1.5 MK at 1.4 $R_\odot$, and remote-sensing measurements (in particular those from SUMER), which indicate electron temperatures below 1 MK. The freezing-in temperatures are normally obtained from in-situ measurements of the charge state distributions of several ions, and with various assumptions, such as the ion flow speed and Maxwellian electrons (see, e.g., Geiss et al. 1995). Esser and Edgar (2000) were able to reconcile the two methods with a Maxwellian distribution close to the coronal base, but that becomes rapidly non-Maxwellian as a function of height. We should note, however, that remote-sensing measurements are uncertain and have recently been revised (see Sect. 12.1), so it is not clear if such discrepancies really exists.

**Low corona**

Various studies based on remote-sensing measurements and modelling have attempted to assess for the presence of non-Maxwellian electrons in the low corona (outside of flares), but results are still inconclusive.

Feldman et al. (2007) used SOHO SUMER active region observations of He-like lines to search for signatures of a secondary Maxwellian component at high temperatures (10 MK), but nothing was found. Hannah et al. (2010) used RHESSI observations of the quiet Sun to place upper limits on the high-energy non-thermal emission. Recent direct HXR imaging (FOXI, NuSTAR) of quiescent active regions typically does not show any non-thermal emission (see, e.g., Ishikawa et al. 2014; Hannah et al. 2016).

One promising aspect for some time was associated with the anomalous Si iii line intensities. Dufton et al. (1984) and subsequent authors (e.g., Keenan et al. 1989a; Pinfield et al. 1999; Dzifčáková and Kulinová 2011) suggested that these discrepancies could have been caused by the presence of non-Maxwellian electron distributions in the low transition region. For example, Dzifčáková and Kulinová (2011) used CHIANTI atomic data, with the excitation cross sections for $\kappa$-distributions being recovered using an approximate parametric method (Dzifčáková and Mason 2008), to show that the Si iii intensities observed with SUMER by Pinfield et al. (1999) could be explained by $\kappa$-distributions. However, Del Zanna et al. (2015c) showed, using new atomic data, that the Si iii line intensities are generally consistent with Maxwellian electrons, if one takes into account the temperature sensitivity of the different Si iii lines.

**Fig. 51:**
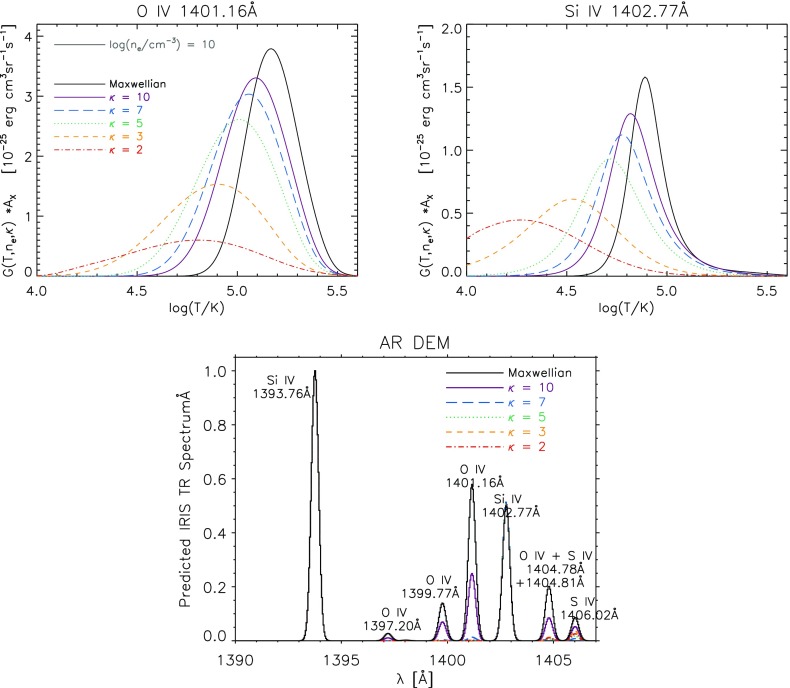
Top: contribution functions *G*(*T*) for the O iv 1401.16 Å (left) and Si iv 1402.77 Å (right) lines, calculated at fixed density and for different electron distributions. Note that the $\kappa$-distributions progressively shift the formation temperature of the lines (especially Si iv) towards lower values. Bottom: IRIS simulated O iv and Si iv line profiles in the FUV channel, normalised to the Si iv intensities. The simulations show how the O iv lines become strongly depressed when $\kappa$-distributions are considered These figures have been adapted from Dudík et al. (2014a)

One general problem with these types of diagnostics is the fact that line ratios that are good diagnostics of non-Maxwellian distributions are normally also sensitive to changes in the electron temperature. Hence, a good independent knowledge of the temperature distribution is often needed. A few recent studies have used the original atomic cross sections to search for diagnostics of non-thermal electron distributions. Dudík et al. (2014a) studied the effects of non-Maxwellian distributions on the O iv and Si iv lines observed by IRIS, showing that strong enhancements of the Si iv/O iv ratios are expected in the case of a $\kappa$-distribution (with high-energy tails), see Fig. 51. Such enhancements are indeed very common, although it is still not clear if time-dependent ionisation, non-Maxwellian distributions or other effects such as the DR suppression could be the main factors (Polito et al. 2016a).

Dudík et al. (2014b) discussed the excitation of Fe ix–Fe xiii under the assumption of a $\kappa$-distribution of electron energies, reviewing the main line ratios for these important coronal ions, which can be used to measure densities, temperatures and departures from Maxwellian distributions. Dudík et al. (2014b) pointed out that a good diagnostic is given by the ratios of the EUV allowed lines with the visible forbidden lines. However, such a comparison requires a good knowledge of the relative calibration.

**Fig. 52:**
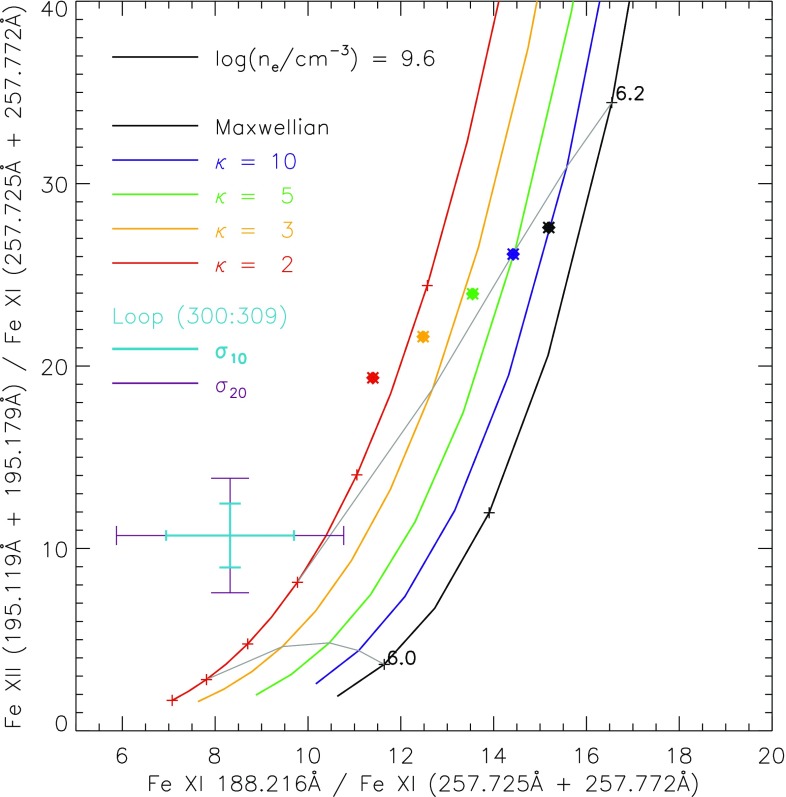
Ratio–ratio diagram of Hinode EIS line intensities for a transient loop. Lines of different colours indicate the theoretical curves for a Maxwellian and different $\kappa$-distributions. The cross, with a 10 and 20% uncertainty, indicates the measured value Adapted from Dudík et al. (2015)

Dudík et al. (2015) used Hinode EIS observations of a transient loop to study the ratios of the observed iron coronal lines that are sensitive to non-Maxwellian distributions. The observations appear to be inconsistent with a Maxwellian distribution (Fig. 52).

## Emission measure: diagnostics

If a unique relationship exists between $N_{\mathrm{e}}$ and *T*, a column differential emission measure ${ DEM}(T)$, a function of only the plasma temperature, can be defined (Withbroe 1978):

88 $$\begin{aligned} \int \limits _T { DEM} (T) dT = \int \limits _h N_e ~N_H dh, \end{aligned}$$

i.e.,

89 $$\begin{aligned} { DEM} (T) = N_e N_H {dh \over dT} \quad (\mathrm{cm}^{-5}\,\mathrm{K}^{-1}). \end{aligned}$$

For example, if we assume that the plasma pressure along the line of sight is constant, then from the perfect-gas law $N_e^2\sim ({P^2/T^2})$, and the electron density is only a function of temperature, and the ${ DEM}(T)$ can be defined as a single-value function of the temperature. The DEM gives an indication of the amount of plasma along the line of sight that is emitting the radiation observed and has a temperature between *T* and $T+dT$.

With this definition, the intensity integral becomes:

90 $$\begin{aligned} {I(\lambda _{ij})}= {Ab(Z)} {\int \limits _T ~{C(T,\lambda _{ij},N_e)} ~{ DEM} (T) ~ dT}, \end{aligned}$$

assuming that the abundance of the element *Ab*(*Z*) is constant along the line of sight.

We therefore have a system of Fredholm integrals of the first kind to be inverted, in order to deduce the DEM from a set of observed intensities. More details on various approximations and inversion techniques can be found below. This inversion procedure is notoriously difficult (see, e.g., Craig and Brown 1976; Judge et al. 1997), but has many advantages over other diagnostics based e.g., on single line ratios. For example, it has the advantage that at the same time the DEM distribution and the elemental abundances can be obtained, if a sufficient number of lines over a broad temperature range is observed. This minimizes the effects of errors in the observation or theory associated with individual lines.

Once the DEM is known, it is possible to define a column emission measure ${ EM}_T$ over some temperature interval $\varDelta T$:

91 $$\begin{aligned} { EM}_T (T_i) \equiv \int _{T_i- {\varDelta T \over 2}}^{T_i+{\varDelta T \over 2}} { DEM}(T) dT \end{aligned}$$

The total column emission measure ${ EM}$ can also be calculated, integrating the DEM over the whole temperature range:

92 $$\begin{aligned} { EM} \equiv \int _h N_e N_H dh = \int _T { DEM}(T)~ dT \quad (\mathrm{cm}^{-5}) \end{aligned}$$

Sometimes the total column emission measure is defined as $${ EM} = \int N_e^2 { dh} = \int { DEM}(T) { dT} $$, with the column differential emission measure defined as $${ DEM}(T)= N_e^2 ({{ dT}/{ dh}})^{-1} $$.

If the DEM is well defined and known, it is then useful to define an *effective temperature*. This is a weighted average of the temperature, where the weight is given by the product of the *G*(*T*, *N*) with the *DEM*:

93 $$\begin{aligned} \log T_{\mathrm{eff}} = \int G{\left( {T, N}\right) }~{ DEM}{\left( {T}\right) } \log T~dT/{\int G{\left( {T}\right) }~{ DEM}{\left( {T}\right) }~dT} \end{aligned}$$

The effective temperature $T_{\mathrm{eff}}$ gives an indication of where most of the line is formed. This is often quite different to $T_{\max }$, the temperature where the *G*(*T*) of a line has a maximum, or where the maximum ion abundance is.

**Fig. 53:**
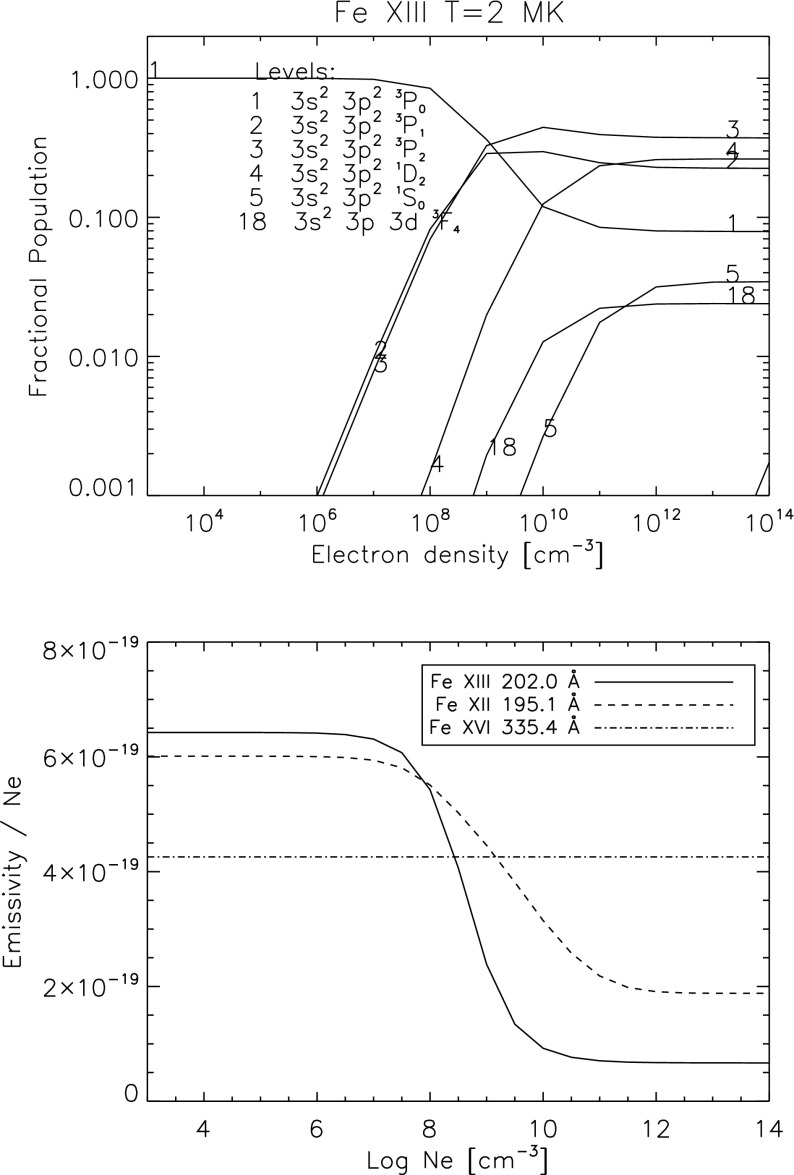
Top: the fractional population of the Fe xiii levels; note the significant decrease in the population of the ground state. Bottom: the emissivity (divided by the density) of the strongest dipole-allowed decays to the ground state of Fe xiii at 202.0 Å, Fe xii at 195.1 Å, and Fe xvi at 335.4 Å. CHIANTI v.8 was used for the emissivities

Further refinements and considerations of the integral inversion problem and extensions to an emission measure differential in both temperature and density can be found e.g., in Almleaky et al. (1989) and Hubeny and Judge (1995). However, observations are often limited by practical considerations, for example the number of spectral lines which can be observed with a given instrument. The simplest approach, used in most literature, is to assume that the ${ DEM}(T)$ is a function of only the plasma temperature, and for the inversion use only spectral lines that have contribution functions *G*(*T*) that do not vary with the density. Normally, these are dipole-allowed lines that decay to the ground state. However, even in these cases some dependence on density is present. This occurs when metastable levels reach a non-negligible population at sufficiently high densities. An example is shown in Fig. 53: the population of the Fe xiii metastable levels becomes so high that the population of the ground state decreases significantly above $N_{\mathrm{e}}=10^{8}\,\hbox {cm}^{-3}$. Therefore, lines such as the 202.0 Å that are mainly populated directly from the ground state have *G*(*T*) that are affected by density changes. Another ion strongly affected is Fe ix. Similar effects are present in many ions, e.g., Fe xii and Fe xxi. On the other hand, lines of ions such as Fe xvi which do not have metastable levels, have *G*(*T*) which are independent of density and are therefore excellent diagnostics for *DEM* analyses.

### Volume versus column EM

As we have previously discussed, most solar measurements are of radiances over a volume along the line of sight, hence the inversion methods produce a *column emission measure*, i.e., the amount of plasma along the line of sight *dh* within the observed area (*A*). Sometimes in the literature the *volume emission measure* ${ EM}_{\mathrm{V}} = \int _V N_{\mathrm{e}}^2 dV ~ (\mathrm{cm}^{-3})$ and its differential in temperature ${ DEM}_{\mathrm{V}}(T) = ~N_{\mathrm{e}}^2 {{ dV} \over { dT}}$ are used instead of the column emission measures, with ${ dV}= A \times dh$.

There are, however, measurements of the Sun as a star (i.e., irradiances), where the relation between column and volume emission measures is non-trivial. One could in principle assume that the distribution of the emitting plasma is homogeneous and has spherical symmetry, i.e., the emitting region is a layer ${ dh}$ distributed over the entire solar disk, i.e., ${ dV}=4\pi R^2_* { dh}$ ($R_*$ is the solar radius). Then the *volume* *DEM* can be scaled with the factor $4\pi R^2_* \over d^2$ to obtain a *column* *DEM*. However, the layers over which lines emitted at different temperatures would be different, so a simple relation is not straightforward.

The same issue occurs when relating the radiances observed over the solar disk to the irradiances. Again if the Sun were homogeneous and only emitting from its disk, the measured irradiance at the Earth in a line $F(\lambda _{ji})$ ($\mathrm{erg}\,\mathrm{cm}^{-2}\,\mathrm{s}^{-1}$) would be related to the radiance on the solar disk $I(\lambda _{ji})$ ($\mathrm{erg}\,\mathrm{cm}^{-2}\,\mathrm{s}^{-1}\,\mathrm{sr}^{-1}$) by

94 $$\begin{aligned} F(\lambda _{ji}) = \pi {R^2_* \over d^2} ~ I(\lambda _{ji}). \end{aligned}$$

However, a significant fraction of the emission in most spectral lines comes from the limb brightening and from the off-limb corona. A correction factor for the limb-brightening for chromospheric and transition-region lines would need to be introduced, to relate the on-disk Sun-centre radiance in a line to its irradiance. A recent review of limb-brightening estimates for EUV/UV lines is given by Andretta and Del Zanna (2014). A further off-limb contribution would also need to be estimated (see, e.g., Del Zanna and Andretta 2010).

### EM, DEM approximations

Over the years, various approximations and methods have been introduced in order to approximate the DEM and to deduce elemental abundances. We describe one method below, while the other ones are described later when we discuss abundance measurements, since those methods were mainly used for this purpose.

#### The emission measure loci approach

One direct approach to assess how the plasma is distributed in temperature is to plot the ratio of the observed intensity $I_{\mathrm{ob}}$ of a line with its contribution function, $I_{\mathrm{ob}}/G(T)$, as a function of temperature. The loci of these curves are an upper limit to the emission measure distribution. In fact, for each line and temperature $T_i$ the value $I_{\mathrm{ob}}/G(T_i)$ represents an upper limit to the value of the *line emission measure* ${ EM}_L$ (Eq. 126, below) at that temperature, assuming that all the observed emission $I_{\mathrm{ob}}$ is produced by plasma at temperature $T_i$.

The method was first introduced by Strong (1979) and later applied by Veck et al. (1984) and following authors to the analysis of solar X-ray spectra, mainly to obtain relative abundances. It was also used to study stellar emission measures (Jordan et al. 1987).

**Fig. 54:**
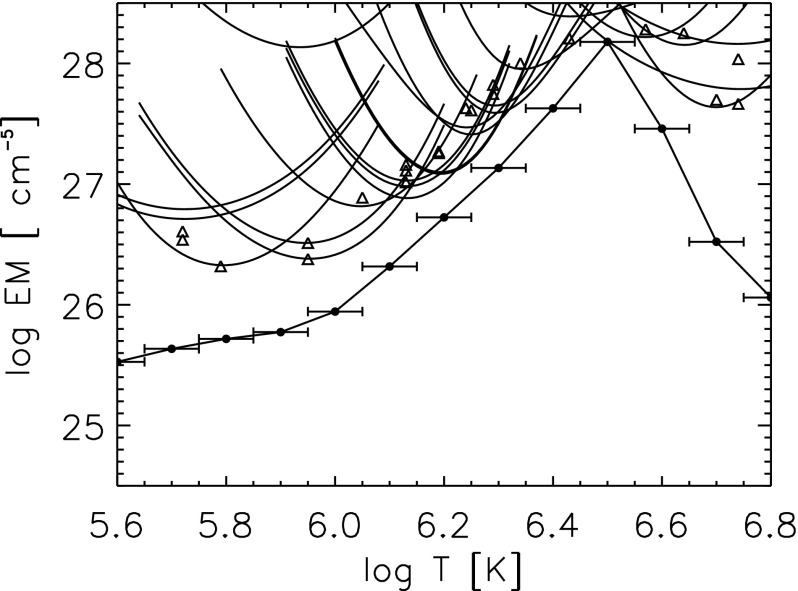
The EM loci ($I_{\mathrm{ob}}/G(T)$) curves for a selection of lines observed by Hinode EIS in an active region core (Del Zanna and Mason 2014). The filled circles are the ${ EM}_T$ values calculated from the DEM with $\varDelta$ log $T=0.1$ K. The triangles, plotted at the temperature $T_{\max }$, are the Jordan and Wilson (1971) values

The $I_{\mathrm{ob}}/G(T)$ curves for a selection of lines observed by Hinode EIS in an active region core (Del Zanna and Mason 2014) are displayed as an example in Fig. 54. The filled circles are the ${ EM}_T$ values calculated from the DEM with $\varDelta$ log $T=0.1$ K. The triangles, plotted at the temperature $T_{\max }$, are the Jordan and Wilson (1971) values (see below in Sect. 14).

### The anomalous EM problem

**Fig. 55:**
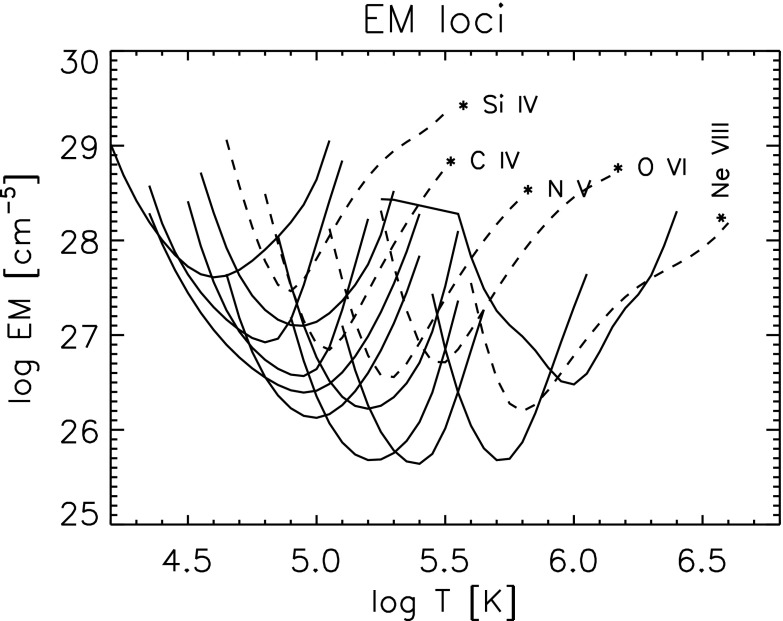
EM loci curves from the solar irradiance measurements presented in Judge et al. (1995) using CHIANTI v.8. The EM loci curves of the spectral lines of the Li- and Na-like sequences are displayed as dashed lines. The other curves are from C ii, O iii, Si iii, N iii, O iv, O v, Ne vii, Mg ix

The Li and Na-like ions give rise to some of the strongest lines in the UV. However, the emission measures for lines emitted by ions of the Li- and Na-like isoelectronic sequences are at odds with those of the other ions. The problem is clearly present even in the early Pottasch (1963) irradiance measurements, although it was not noted by that author (see Fig. 105). The issue was mentioned by Burton et al. (1971) and Dupree (1972), and later discussed by several authors, e.g., Judge et al. (1995). For a detailed discussion see Del Zanna et al. (2002) and references therein. This anomaly is illustrated in Fig. 55, where the solar irradiance measurements presented in Judge et al. (1995) are combined with the atomic data in CHIANTI v.8. Note that the irradiances were approximately converted to radiances by Judge et al. (1995). It can be seen that the lines from the Li- and Na-like isoelectronic sequences are clearly at odds with the other isoelectronic sequences.

Del Zanna et al. (2002) showed that the same problem occurs when stellar emission measures are considered. This was shown by combining XUV observations from different instruments (FUSE, HST/STIS, EUVE). All previous literature considered stellar observations at wavelengths where by far the brightest lines are those from Li- and Na-like ions (e.g., IUE and HST/STIS), so all the published results (in terms of DEM, abundances, densities) were incorrect. In fact, using ions from the Li- and Na-like sequences together with those from other sequences leads to incorrect estimates of elemental abundances, densities, and temperatures.

There are clearly some processes by which the lines of the anomalous ions are enhanced. The enhancements vary depending on the region observed, but are typically about a factor of 5. They affect lines formed in the lower and upper transition region (0.2–0.9 MK), but occasionally also coronal lines (1–3 MK). One possible explanation that has recurrently been suggested is the possible effect of high densities in the charge state distribution. However, this effect is not very large (see, e.g., Bradshaw 2004; Doyle et al. 2005), and would require high densities ($N_{\mathrm{e}}=10^{11}\,\hbox {cm}^{-3}$ or more), so it would presumably only affect lines formed in the low transition region.

The contribution functions of the Li-like and Na-like ions have a significant high-temperature tail, i.e., are more sensitive to high-energy electrons. Strong departures from Maxwellian distributions could in principle explain the discrepancies.

It is interesting to note that even larger enhancements are observed in all the EUV helium lines in the quiet Sun. There is an extensive literature on this subject, where many possible explanations have been considered. One issue that is not clear, for the helium lines, is their formation process. Some authors believe (see, e.g., Zirin 1975) that the dominant process is photoionisation followed by recombination to the upper level of the line (either directly or in a cascade through intermediate levels). If collisional excitation is the dominant process (see e.g., Athay 1988), then the large excitation energy compared to their ionisation equilibrium temperature would make those lines very sensitive to non-thermal effects. Also, the large non-thermal velocities observed imply that many of the ions would be subject to higher-temperature thermal electrons in the regions where the temperature gradient is large, as in the solar transition region (Jordan 1975, 1980). Ambipolar diffusion is also an important process (see, e.g., Fontenla et al. 1993, 2002). Non-equilibrium ionization effects can also increase helium and TR line intensities (Raymond 1990; Bradshaw et al. 2004). Further details on the helium EUV lines can be found in Andretta et al. (2003), Pietarila and Judge (2004), Jordan et al. (2005) and Andretta et al. (2008) and references therein.

### Methods to estimate the differential emission measure

There are many methods and codes available in the literature to obtain estimates of the DEM, using either spectral lines or broad-band imaging. Each method has advantages and limitations and results can often be misleading. Only a brief summary of some of the most widely used methods is given here.

The inversion problem itself is not simple and requires some assumptions about the nature of the solution. A series of workshops was sponsored in 1990/1991 to study differential emission measure techniques (Harrison and Thompson 1992). It was found that most codes eventually gave consistent results, but that the DEM derived depends rather critically on the methods used to constrain the solution and the errors in the observed intensities and atomic data.

Perhaps one of the earliest methods developed to estimate the DEM was the iterative approach of Withbroe (1975), later implemented by several other authors, mostly for the analysis of solar X-ray observations. A statistical method based on the Bayesian formalism and using the Markov-Chain Monte Carlo (MCMC) approach was developed by Kashyap and Drake (1998). This MCMC method is part of the PINTofALE^5^ modelling package and is widely used. The advantages of this method are that it provides positive solutions, a measure of the associated uncertainty, and does not require a prescribed functional form, although the program forces the solution to be smooth. The program starts with a guess solution and applies random adjustments in a Markov-Chain, producing a family of DEM solutions that are a representation of the probability distribution function of the actual DEM. The program is robust but very slow, compared to other methods.

Most codes adopt a simple $\chi ^2$ approach. However, since the solution is normally undetermined (has more degrees of freedom than the input values), the DEM solutions have to be ‘regularised’ in some way, to obtain a smooth result. One way to enforce a smooth and positive solution is to impose a functional form to the DEM. One of simplest functional forms is a single Gaussian, and such an approach was developed by e.g., Aschwanden and Boerner (2011) and Guennou et al. (2012). Another common example is to assume that the DEM can be modelled with a spline function. Such an approach, combined with a simple $\chi ^2$ minimization (using MPFIT), is adopted by the XRT_DEM iterative method (available within SolarSoft), coded by M. Weber, and widely used. The minimization provides DEM values at the spline nodes which best describe the observations. It is possible to evaluate uncertainties by running the code many times, by varying the input intensities to within their uncertainties (a sort of Monte Carlo forward modelling).

Additional constraints are often added using Lagrange multipliers, as with the ‘maximum entropy method’, as discussed by A. Burgess in one of the DEM workshops (Harrison and Thompson 1992). A common implementation of this method has the additional assumption that the DEM is a cubic spline. This procedure has the advantage of giving only positive *DEM* values and converging quickly. This method was adopted by several authors, e.g., Monsignori Fossi and Landini (1991), Landi and Landini (1997) and Del Zanna (1999). Note that the smoothness of the DEM distribution depends on the number and position of the selected mesh points, which should be chosen at those temperatures where there are constraints from the observations.

Another approach is the so-called *zero-order regularization*, which was implemented by e.g., Hannah and Kontar (2012) and Plowman et al. (2013). The DATA2DEM_REG^6^ routine implemented by Hannah and Kontar (2012) recovers the DEM using a Tikhonov regularization and a generalized singular value decomposition (GSVD) approach. The solution is not always positive which is a major disadvantage, but the code is faster than most other methods.

**Fig. 56:**
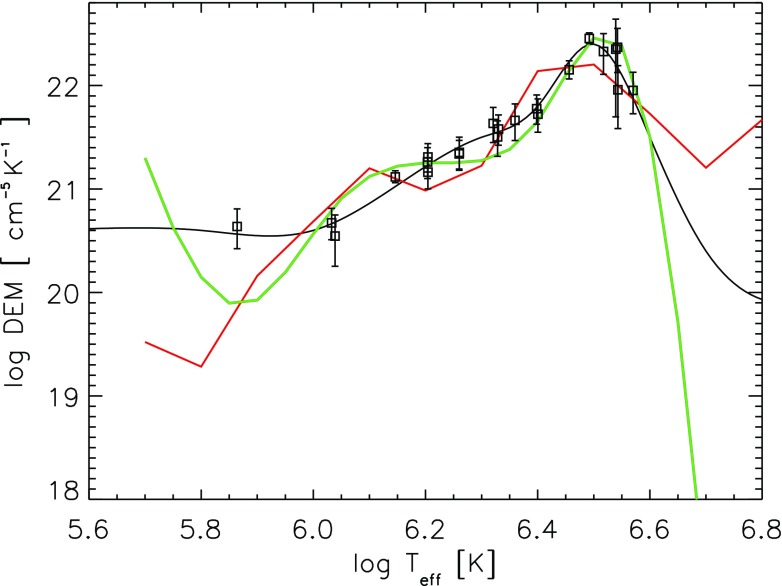
DEM of an AR core observed with Hinode EIS, obtained with the spline (MEM_DEM) method (black smooth curve), with the MCMC_DEM program (red curve) and XRT_DEM method (green curve). The points are plotted at their effective temperature and the corresponding DEM value, multiplied by the ratio of the observed versus predicted intensity Adapted from Del Zanna and Mason (2014)

In the temperature regions where the DEM is well constrained, the results of the various methods normally agree quite well. The best constraints are given by a range of individual emission lines formed over a wide range of temperatures. Figure 56 shows a comparison of different DEM methods using Hinode EIS observations in an active region core (Del Zanna and Mason 2014).

One major limitation which can occurs is when the plasma is nearly isothermal. In this case, most codes generally fail, as they enforce a continuous DEM solution. A possible way to recover both a nearly isothermal and a continuous solution was presented by Del Zanna (2013a), where the DEM was modelled by a superposition of Gaussian distributions. A simple $\chi ^2$ minimization iterative method was used to obtain DEM solutions from SDO AIA data. The solution could be nearly isothermal, with a suitable choice of the width of the Gaussians, or continuous across the entire temperature range. A similar approach (in terms of basis functions) was introduced by Cheung et al. (2015) to obtain DEM solutions from SDO AIA data, although the actual inversion method is different and its implementation is fast.

Another significant limitation of all the available codes is when the kernels (i.e., the line contribution function or a temperature response for a broad-band imaging instrument) have a double peak feature. For example, several of the SDO AIA channels have this problem. Naturally, the codes tend to find continuous DEM curves which cover both peaks, but these are not necessarily the correct solutions. A way to improve the solution is to have different kernels (and input) for different cases, as suggested for SDO AIA by Del Zanna (2013a).

It has been shown by Dere (1978a) that if the DEM is approximated with a non-negative smooth function, then the solutions have errors that are not significantly greater than the errors in the spectral data, in the temperature ranges where there are observational constraints. In the regions with limited observational constraints, the DEM values can have large errors, as shown by e.g., Jakimiec et al. (1984).

## Emission measure: observations

The above methods have been applied in a wide range of cases. As we have mentioned, the main purpose is to define the temperature distribution in the plasma, which is a fundamental parameter for any coronal heating study.

Once the DEM/EM is known, estimates of densities can be obtained (as described below), as well as the optically thin radiative losses, which are needed to study the energy budget in any coronal structure. Earlier studies used the DEM slope below 1 MK to infer the temperature gradient in the transition-region and build semi-empirical hydrostatic models (see e.g., Mariska 1992). Later studies, briefly mentioned below, focused more on the forward-modelling, i.e., built theoretical models and then predicted the DEM distribution.

A second and important purpose of the DEM/EM methods is to obtain the relative elemental abundance, as described below in Sect. 14.

The literature on EM/DEM measurements is too extensive to even be summarised here. Measurements across the XUV spectrum on all solar features, using both spectral lines and broad-band instruments have been carried out for over 50 years. We focus in this review on more direct measurements of temperatures. A selection of results on some of the main solar features is however presented here, to provide an overview to the unfamiliar reader.

### Quiet Sun and coronal holes

**Fig. 57:**
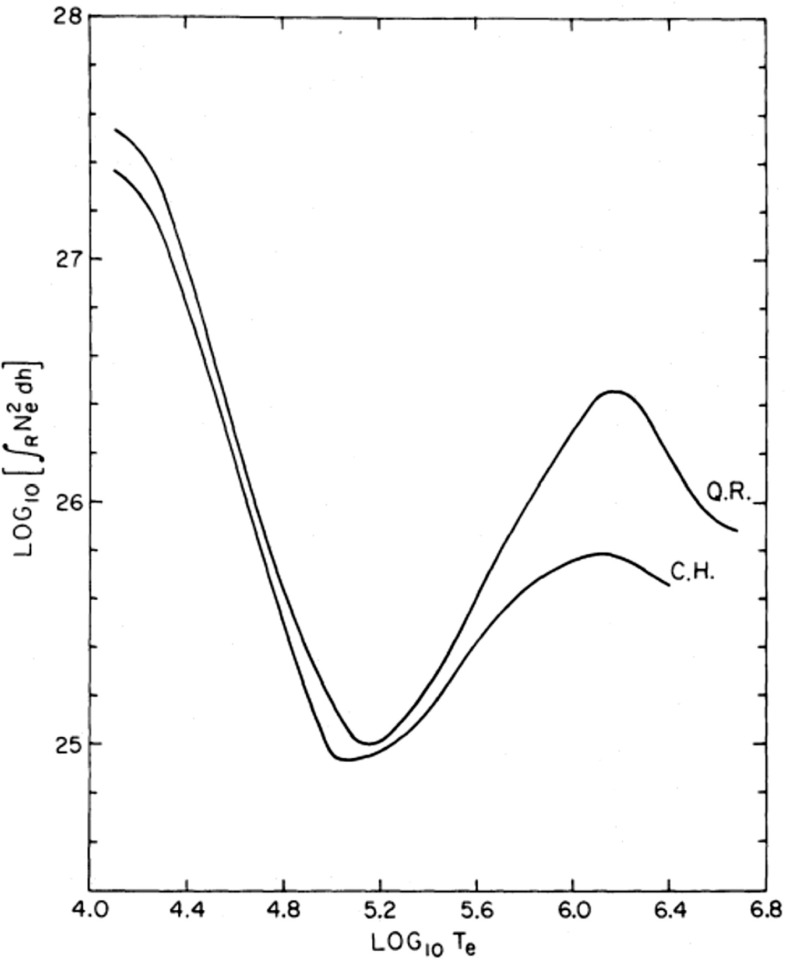
The DEM distributions of the average quiet Sun and coronal hole obtained by Raymond and Doyle (1981)

Following Pottasch (1963), all subsequent measurements based on solar irradiances clearly showed that the quiet Sun has a peak EM/DEM around 1 MK, a minimum around log $T\,(\hbox {K})=5.2$, and a steep increase towards the chromosphere. Radiance measurements of the quiet Sun on-disk produced the same picture. Notable examples are the results obtained by Withbroe (1975), based on OSO-IV and OSO-VI, and those based on Skylab observations (see, e.g., Raymond and Doyle 1981 and Fig. 57). The slope above 2 MK is difficult to establish, as there is very little emission from the quiet Sun. All the observations from later instruments gave similar results.

In off-limb observations of the quiet Sun, the EM distribution tends to be very narrow around 1 MK, as e.g., clearly shown with the EM loci method applied to SOHO SUMER observations (see, e.g., Feldman et al. 1997; Landi and Feldman 2003) or Hinode EIS (see, e.g., Warren and Brooks 2009; Del Zanna 2012b). The peak temperature of the EM distribution typically increases with the height above the limb.

Radiance measurements of coronal holes typically produce EM distributions that are similar to the quiet Sun ones, up to 1 MK, where a sharp drop occurs (i.e., the coronal distribution is nearly isothermal). In fact, transition region lines have almost the same intensity distribution inside and outside coronal holes, with the main difference that the brightest supergranular cell boundaries (network) are depressed in coronal holes (by about 50%), and the height of the TR layer is much higher. Notable results are those based on OSO-IV and Skylab observations, such as those of Munro and Withbroe (1972) and Raymond and Doyle (1981). All later results based on SOHO CDS (see, e.g., Mason et al. 1997; Del Zanna and Bromage 1999b; Young et al. 1999), SOHO SUMER (see, e.g., Feldman et al. 1998b; Landi 2008), Hinode EIS (see, e.g., Hahn et al. 2011), and other missions were similar.

Within coronal holes, plumes are long-lasting features (see the recent review from Poletto 2015). These were spatially resolved with SOHO CDS, and EM/DEM analyses clearly showed that near their bases, the cross-sections of plumes are almost isothermal (Del Zanna and Bromage 1999a; Del Zanna et al. 2003). This confirmed an earlier suggestion of Ahmad and Withbroe (1977) based on off-limb Skylab HCO observations of Mg x and O vi. The isothermality of the plumes has important implications in terms of the derived elemental abundances, as discussed below in Sect. 14.

### Active region cores

A popular theory for the coronal heating assumes that nanoflare storms occurring in the corona heat the plasma to $T_{\mathrm{e}} >3$ MK, with subsequent cooling to form the $\simeq 3\,\hbox {MK}$ loops typical of AR quiescent cores (see e.g., in particular Parker 1988; Klimchuk 2006, 2010; Patsourakos and Klimchuk 2009; Bradshaw and Klimchuk 2011; Cargill 2014 and in general the Living Review by Reale 2014). Other theories, such as the dissipation of Alfven waves (van Ballegooijen et al. 2011) also predict high-temperature plasma heated by short-lived and frequent heating events. Measuring the slopes *a* [$\hbox {EM}(\hbox {T})~\propto \,\hbox {T}^{-a}$] at temperatures above 3 MK is therefore fundamental for constraining the heating of the core loops. The frequency of the heating events also leaves an imprint in the plasma emission. In the high-frequency heating scenario, the duration between successive heating events is shorter than the cooling time, which produces a narrow EM distribution with a steep slope *b* [$\hbox {EM}(\hbox {T})~\propto \,\hbox {T}^b$] in the 1–3 MK range. However, in the low-frequency heating scenario, the time duration is longer than the cooling time, and the plasma has sufficient time to cool down before being heated again. Hence, there is a substantial amount of cooler material, producing shallower EM slopes (see e.g., Mulu-Moore et al. 2011; Tripathi et al. 2011; Bradshaw et al. 2012; Cargill 2014 and references therein). Generally, the models predict that low-frequency nanoflares can only account for slopes *b* that are below 3.

There is a vast literature with contradictory results in terms of the slopes *a*, *b*. Our view has always been that the AR cores are nearly isothermal around 3 MK because it was clear from CDS images that they were only visible in Fe xvi (see, e.g., Del Zanna and Mason 2003). This view is not new. For example, Rosner et al. (1978) found that the excellent Skylab X-ray imaging was consistent with nearly isothermal plasma at 3 MK. Evans and Pounds (1968) found similar results using X-ray spectroscopy. However, the limitation of X-ray spectroscopy is that only lines formed above 2–3 MK are observed. Many analyses of X-ray spectra such as those of SMM FCS indeed assumed that the plasma was isothermal (see, e.g., Schmelz et al. 1996). Results from other instruments have also confirmed that the emission in the cores is always around 3 MK. For example, this was obtained from Yohkoh BCS (see, e.g., Sterling et al. 1997).

EUV spectroscopy with SOHO CDS (see, e.g., Mason et al. 1999; Del Zanna and Mason 2003) and Hinode EIS (see, e.g., O’Dwyer et al. 2011; Tripathi et al. 2011; Winebarger et al. 2011; Warren et al. 2012; Schmelz and Pathak 2012) have shown a relatively steep plasma distribution in the 1–3 MK range, but with a wide range of values of *b*, between 2 and 5. The revised Hinode EIS calibration of Del Zanna (2013b) substantially increased the slopes *b* to values around 5, which increase even further if foreground/background emission is taken into account (Del Zanna 2013a; Del Zanna et al. 2015d). These results suggest that low-frequency nanoflare modeling should be ruled out. However, evidence of significant lower-temperature emission in AR cores, interpreted as signatures of cooling, has also been presented (see, e.g., Viall and Klimchuk 2012).

Clearly, it is not easy to disentangle line of sight issues without high spatial resolution stereoscopy. Our view (see, e.g., Del Zanna and Mason 2003; O’Dwyer et al. 2011; Del Zanna 2013a), based on the spatial distribution of structures seen with SOHO CDS and Hinode EIS in lines formed over different temperatures, is that AR cores present a near isothermal distribution near 3 MK in the core, a less isothermal (around 2 MK) ‘unresolved’ and more extended emission, plus a near isothermal emission around 1 MK in warm loops. SOHO SUMER observations of an active region above the limb also indicate the presence of three near-isothermal structures (Landi and Feldman 2008).

Regarding the high-temperature slopes *a*, a much larger scatter of values is found in the literature. One main problem is the limitation of EUV spectroscopy to observe lines above 3 MK. For example, Hinode EIS lines from e.g., Fe xvii, Fe xxiii, Fe xxiv, Ca xvi, Ca xvii are all blended (see, e.g., Young et al. 2007a; Del Zanna 2008b; Ko et al. 2009; Del Zanna et al. 2011b). Several studies complemented these EUV observations with imaging data from the X-rays (e.g., Hinode XRT) or soft-Xrays (e.g., SDO AIA), however the results are always uncertain, given the multi-thermal nature of imaging data. For example, the SDO AIA 94 Å band was used by Warren et al. (2012) to find slopes *a* ranging between 6 and 10. This band was thought to be dominated by Fe xviii. However, Del Zanna (2013a) showed that a significant fraction of the AIA 94 Å counts could be due to a newly identified Fe xiv line, and that most of the Fe xviii weak emission is formed at 3 MK and not at higher temperatures.

A few other studies measured the hot emission at different wavelengths. For example, Teriaca et al. (2012) measured Fe xviii 974 Å emission in SOHO SUMER, while Brosius et al. (2014) measured the Fe xix 592.2 Å emission with the EUNIS-13 rocket flight.

Another problem is the fact that AR cores often exhibit microflaring activity, so single measurements are not necessarily representative of the quiescent emission.

The above limitations were overcome by Del Zanna and Mason (2014) with the use of SMM observations. Only observations of quiescent cores of several active regions were chosen, based on the lightcurves of the BCS instrument. The FCS X-ray spectra were then used to find steep slopes ($a \simeq 14$ in one case) from a peak around 3 MK. An upper limit to the EM at 7 MK was obtained from the SMM spectra. It was about three orders of magnitude lower than the value of the EM at 3 MK. It was also found that the Fe xvii and Fe xviii lines were mainly formed around 3 MK, and not at higher $T_{\mathrm{e}}$ as one would expect. Similar results have recently been obtained by combining EIS and SUMER observations of an active region observed off-limb (Parenti et al. 2017), with the difference that in several places faint Fe xix emission was observed, providing the need for the presence of an EM three orders of magnitude below the peak in those regions.

Steep slopes (*a* about 10) have recently also been found with direct focusing X-ray optics by FOXSI (Ishikawa et al. 2014; Glesener et al. 2016). Several results from the hard X-ray astrophysical mission Nuclear Spectroscopic Telescope ARray (NuSTAR) have recently been presented, confirming steep slopes (see, e.g., Grefenstette et al. 2016; Hannah et al. 2016).

Finally, we recall that high temperatures could actually exist, if the plasma was out of ionization equilibrium (see Sect. 6).

### Moss

The term ‘moss’ was introduced (cf. Berger et al. 1999) because of the textured appearance of this low-lying emission, best seen in the TRACE EUV 173 Å passband. However, the first description of this emission is due to Peres et al. (1994), that reported high-resolution Normal Incidence X-ray Telescope (NIXT) observations. The NIXT passband had a peak response at $T \sim 1$ MK, similar to the TRACE 173 Å one. Berger et al. (1999) presented TRACE and Yohkoh data, together with ground-based observations. They found that moss was mostly associated with magnetic plage as seen in the Ca II K line. Yohkoh/SXT images showed emission overlying the regions where the moss was located, and therefore led the authors to conclude that moss regions occur only above the magnetic plage that underlie soft X-ray coronal loops. Martens et al. (2000) proposed that moss is the transition-region emission of the 3 MK core loops seen with Yohkoh/SXT, a view that is now commonly accepted.

Fletcher and De Pontieu (1999) analysed SoHO CDS observations of the same active region discussed by Berger et al. (1999). They found, with a *DEM* analysis, that a patch of moss emission mostly emits at 1–3 MK temperatures. Hinode EIS observations have confirmed that moss emission peaks around 2 MK (see, e.g., Tripathi et al. 2010).

### Active region unresolved emission

The cores of active regions are normally surrounded by a ‘halo’ of diffuse, unresolved emission between 1 and 3 MK. This emission is more extended than the core 3 MK emission and is typically multi-thermal, as shown e.g., by Del Zanna and Mason (2003) with SoHO CDS and e.g., O’Dwyer et al. (2011), Del Zanna (2012b) and Subramanian et al. (2014) with Hinode EIS.

### Coronal warm (1 MK) and fan loops

Active regions also contain the so-called warm loops, i.e., structures which reach about 1 MK at their top and appear to be nearly resolved. These loops have been observed in their full glory in the TRACE 173 Å band, and more recently with the SDO AIA 171 Å images.

Del Zanna (2003) and Cirtain et al. (2007) used the EM loci method applied to SOHO CDS observations (at about $4{^{\prime \prime }}$ resolution) of legs of ‘warm’ (1 MK) loops, which have strong emission in upper TR lines. They found that these loops appear to be nearly isothermal in their cross-section. In fact, if the $I_{\mathrm{ob}}/G(T)$ curves all cross at one temperature, this means that the observations are consistent with the plasma being isothermal (although this is not necessarily always the case). Cooler and hotter loops are intermingled and fill the entire AR volume (Del Zanna et al. 2006b).

Hinode EIS, with its effective $3{^{\prime \prime }}$ resolution, allowed measurements of some parts of the coronal warm loops, which overall also show near isothermal distributions (see, e.g., Warren et al. 2008b; Tripathi et al. 2009).

**Fig. 58:**
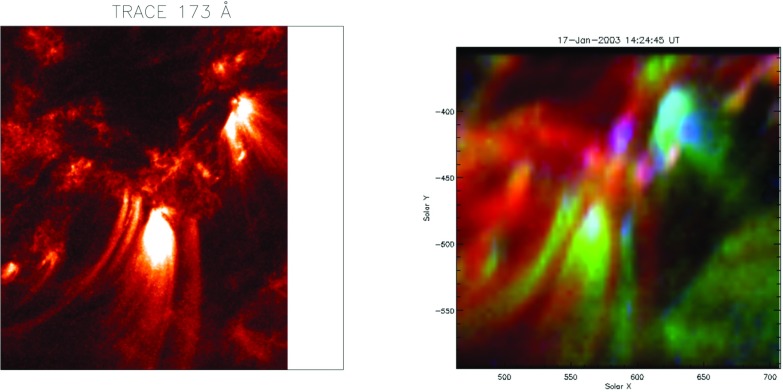
Left: TRACE 173 Å (1 MK) images of the legs of warm 1 MK loops (bottom) and fan loops (on the right). Right: false-colour image obtained from monochromatic CDS images (Ne vii, 0.7 MK, in blue; Ca x, 1 MK, in green; and Si xii, 3 MK, in red). The warm loops (green) are clearly seen in TRACE, however hotter loops (red) are not visible in this band. CDS also shows that fan loops are cooler Adapted from Del Zanna et al. (2006b)

The so-called fan loops are typically anchored in sunspots and their penumbra, are cooler than the warm loops, and are condensed in the form of a fan. This was obvious from SoHO CDS observations (see, e.g., Cirtain et al. 2007 and Fig. 58), and later with Hinode EIS (see, e.g., Del Zanna 2009a; Brooks et al. 2011).

## Diagnostics of electron densities

### $N_{\mathrm{e}}$ from line ratios

The main principle for the spectroscopic diagnostic of the electron density involves a ratio of two lines, where at least one line is connected to a metastable level. We recall that metastable levels have very small radiative decay rates, so that collisional de-excitation competes with radiative decay as a depopulating process.

The ratio of two spectral lines, where one is populated from the ground state and one is either a decay from a metastable level (a forbidden or intersystem transition) or is an allowed transition that is populated from a metastable level is therefore strongly dependent on the electron density. A ratio of two forbidden or intersystem lines, decays from two different metastable levels, is also a density diagnostic. This is the basic principle of most of the electron density diagnostics (see Gabriel and Mason 1982).

For illustration purposes we consider a simplified model ion. For forbidden transitions the radiative decay rate is very small ($A_{m,g} \simeq 10^0{-}10^2\,\mathrm{s}^{-1}$), so the collisional de-excitation term ($N_{\mathrm{e}}~ C^e_{m,g}$) becomes an important depopulating mechanism at sufficiently high densities. We recall that

95 $$\begin{aligned} N_m={N_{\mathrm{g}} N_{\mathrm{e}} C^e_{g,m} \over {N_{\mathrm{e}} C^e_{m,g} + A_{m,g}}} \end{aligned}$$

For small electron densities, $N_{\mathrm{e}} \rightarrow 0, A_{m,g} \gg N_{\mathrm{e}} C^e_{m,g}$, then the intensity has the same dependence on the density as an allowed line ($I^A$):

96 $$\begin{aligned} I_{m,g}~ \simeq N_{\mathrm{e}}^2 \end{aligned}$$

For very large values of electron density, $N_{\mathrm{e}}\rightarrow \infty$, the collisional de-population dominates, $N_{\mathrm{e}} C^e_{m,g} \gg A_{m,g}$; the metastable level is in Boltzmann equilibrium with the ground level:

97 $$\begin{aligned} {N_{\mathrm{m}} \over N_{\mathrm{g}}}={C^e_{\mathrm{g,m}} \over C^e_{\mathrm{m,g}}} = {\omega _m \over \omega _{\mathrm{g}}}~exp\left( {-\varDelta E_{\mathrm{g,m}} \over kT}\right) \end{aligned}$$

The line intensity has the form:

98 $$\begin{aligned} I_{\mathrm{m,g}} \simeq N_{\mathrm{e}} \end{aligned}$$

For intermediate values of electron density ($A_{m,g} \simeq N_{\mathrm{e}} C^e_{m,g}$), the population of the metastable level is significant and the intensity varies as:

99 $$\begin{aligned} I_{m,g} \simeq N_{\mathrm{e}}^\beta \quad 1< \beta <2 \end{aligned}$$

The intensity ratio of a forbidden to an allowed transition ($I^F/I^A$) for different spectral lines from the same ion can be used to determine an average electron density for the emitting volume. This value is independent of the elemental abundance, ionisation ratio and any assumptions about the size of that volume.

If the population of metastable level (*m*) is comparable with the ground level (*g*), then other excited levels (*k*) can be populated from this metastable level as well as from the ground level and the dependence of the intensity on electron density becomes:

100 $$\begin{aligned} I_{k,m} \simeq N_{\mathrm{e}}^\beta \quad 2< \beta <3 \end{aligned}$$

The classical simple example is the Fe xiv case. The ground configuration $3{s}^23{p}$ has two levels—$^2\hbox {P}_{1/2}$ and $^2\hbox {P}_{3/2}$—the transition between these two levels gives rise to the coronal green line at 5303 Å, with a probability to the ground level of only $60\,\hbox {s}^{-1}$. For low electron densities (less than $10^8\,\mathrm{cm}^{-3}$), almost all the population for Fe XIV is in the ground level, but as the electron density increases (greater than $10^{10}\,\mathrm{cm}^{-3}$), the upper level becomes more populated.

**Fig. 59:**
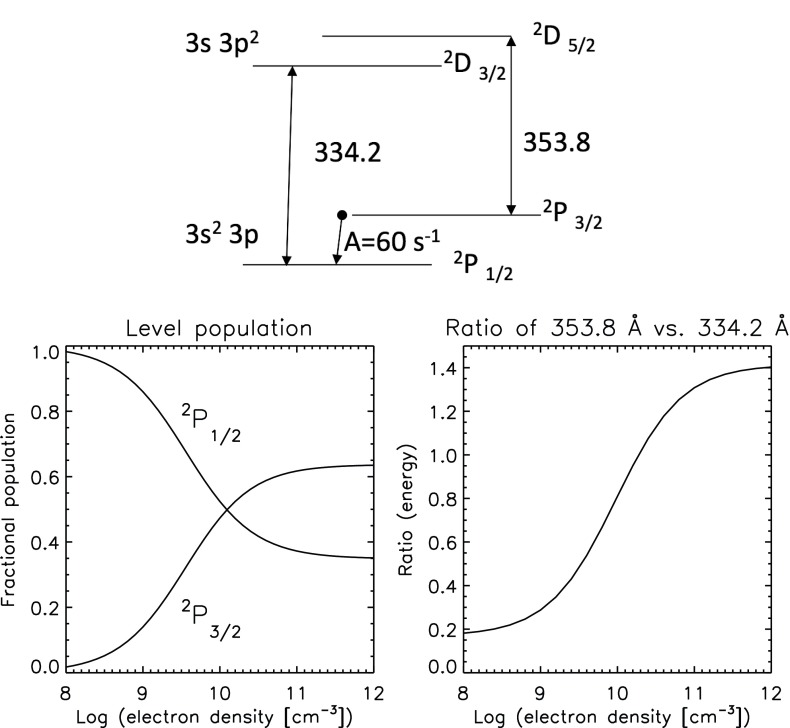
Electron density diagnostics from Fe xiv

The upper level of the spectral line at 334.2 Å is mainly populated by direct excitation from the ground level ($3{s}^{2}\,3{p}~{}^2\hbox {P}_{1/2}$), whereas the upper level of the 353.8 Å line is mainly excited from the upper level of the ground configuration ($3{s}^{2}\,3{p}~{}^2\hbox {P}_{3/2}$). The intensity ratio of these two lines therefore varies with the electron density, following the relative change in the populations of the two levels in the ground configuration, as shown in Fig. 59. This ratio was observed, e.g., by SOHO CDS (see Mason et al. 1999).

To avoid additional uncertainties, lines from the same ion should be used to measure the density. Also, lines that have a similar temperature dependence should be used. The temperature dependence comes in when the lines have very different excitation energies, which means that lines far away in wavelength should in general not be used.

Each ion and line ratio has a range of density sensitivity. The number of diagnostics depends on the number of metastable levels, the most important ones being those in the ground configuration (the most populated). As the density increases, it reaches a point where the levels reach Boltzmann equilibrium, so the level population does not change with density. For more details, see Gabriel and Mason (1982).

In the visible range of the spectrum, density-sensitive line ratios have been studied for a long time (Aller et al. 1949). We note that lines in the visible wavelength range are strongly affected by photoexcitation, a process that needs to be included in the modeling. Ratios of a forbidden or intersystem to an allowed line are very common diagnostics, but they can also be sensitive to the temperature. Examples are the Be-like C iii 1909/977 and O v 1218/629 Å (see below). Ratios of two intersystem lines such as the O iv 1407.3/1404.8 Å are not sensitive to the temperature, hence are better in this respect (but see below).

Ratios of two allowed lines are very common, for example, the Fe xii 186.8/195.1 Å (see below). Both lines are usually populated from the ground configuration so have a similar temperature dependence. However, if one of the lines is populated by a metastable level that is in an excited configuration, then there will be some temperature dependence. Examples are the strong TR lines from the Be-like ions C iii 1176/977 Å and O v 760/629 Å (see below), which are ratios of multiplets of lines with the resonance lines.

In any case, the density that can be obtained is always going to be an averaged value along the line of sight over the formation temperature for that ion, weighted by the (unknown) density distribution. If the plasma distribution is homogeneous, then the measured value is the real value. However, if the plasma is inhomogeneous, different line ratios can produce different density values, as, e.g., Doschek (1984) pointed out. The dominant contribution obviously comes from the regions with the highest densities. In principle, if several line ratios are observed, one could try to estimate the density distribution. However, to date uncertainties on the radiometric calibration and on the atomic data have been significant and made such measurements difficult. We note that the situation in recent years has dramatically improved with regard to the accuracy of the atomic data, but not significantly improved with regard to the reliability of the instrument calibration.

### $N_{\mathrm{e}}$ from multiple lines within the same ion

Plots of theoretical ratios of lines as a function of the electron density are common in the literature. Quite often, some line ratios show discrepancies between theory and observations, and it is not obvious which line is most affected. When several lines from the ion are observed, it is much more instructive to plot all the information about the lines at once. This was originally suggested by Brunella Monsignori Fossi, and has been implemented in two slightly different ways, the *L*-function method (Landi and Landini 1997) and the emissivity ratio method (Del Zanna et al. 2004a).

**Fig. 60:**
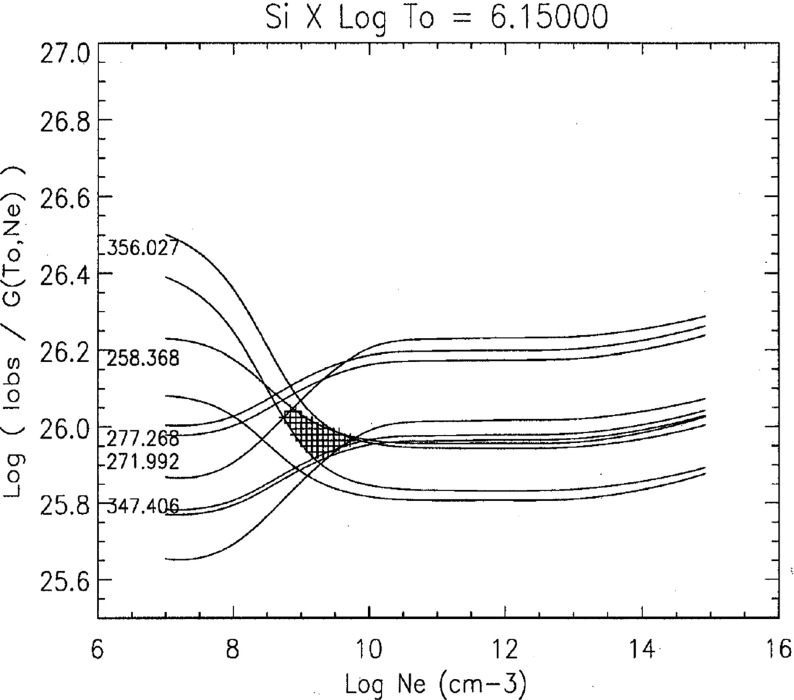
The *L*-function curves of a few among the strongest EUV lines of Si x Image reproduced with permission from Landi and Landini (1997), copyright by ESO

The *L*-function method consists in basically plotting the ratio of the observed intensity (or flux) in a line with its contribution function *G*(*T*, *N*), calculated at an effective temperature $T_{\mathrm{eff}}$:

101 $$\begin{aligned} L = \frac{I_{\mathrm{ob}}}{G(N_{\mathrm{e}}, T_{\mathrm{eff}})}. \end{aligned}$$

An example is shown in Fig. 60. We recall that the effective temperature gives an indication of where most of the line is formed. This is often quite different to $T_{\max }$, the temperature where the *G*(*T*) of a line has a maximum, or where the maximum ion abundance is. If the plasma distribution along the line of sight has constant density, all the *L*-function curves should be either overlapping (if the lines have the same density sensitivity) or intersect. One non-trivial issue is obtaining the effective temperature of the lines as it requires the calculation of the DEM, with all its associated uncertainties. However, in principle the method can also be used when the plasma is isothermal.

**Fig. 61:**
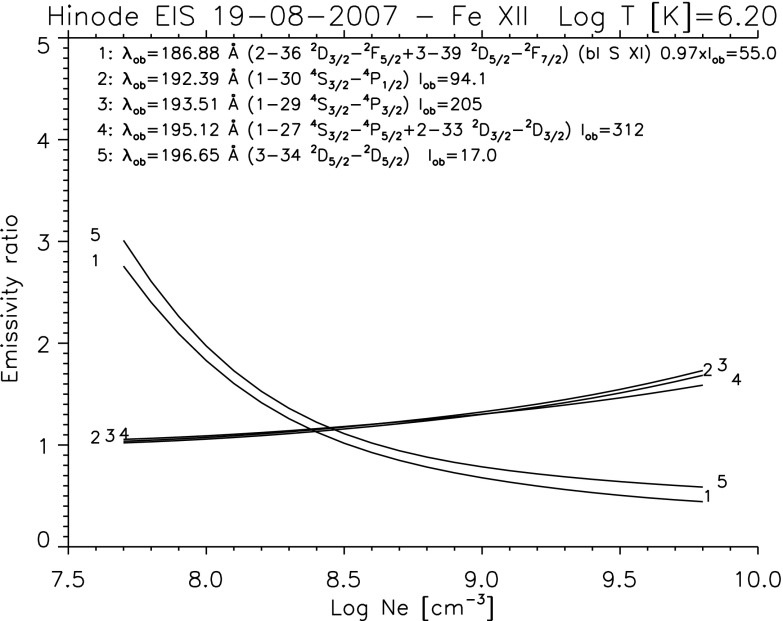
The emissivity ratio curves of a few among the strongest lines of Fe xii, as observed with Hinode EIS and using the new atomic data Adapted from Del Zanna et al. (2012b)

The other similar approach is the emissivity ratio method, where the ratios of the observed ($I_{\mathrm{ob}}$, energy units) and the calculated line emissivities:

102 $$\begin{aligned} R_{ji}= {I_{\mathrm{ob}} N_{\mathrm{e}} \lambda _{ji} \over N_j(N_{\mathrm{e}}, T_{\mathrm{e}})\,A_{ji}}\,{\mathrm{Const}} \end{aligned}$$

are plotted as a function of the electron density $N_{\mathrm{e}}$. Here, $N_j (N_{\mathrm{e}}, T_{\mathrm{e}})$ is the population of the upper level *j* relative to the total number density of the ion, calculated at a fixed temperature $T_{\mathrm{e}}$ (the same for all the lines), $\lambda _{ji}$ is the wavelength of the transition, $A_{ji}$ is the spontaneous radiative transition probability, and ${\mathrm{Const}}$ is a scaling constant that is the same for all the lines within one observation. The value of ${\mathrm{Const}}$ is chosen so that the emissivity ratios $R_{ji}$ are near unity where they intersect, so the scatter of the curves around unity provides a direct measure on the relative agreement between predicted and observed intensities. One example is shown in Fig. 61. There is agreement within about 10% for all the lines at $\log N_{\mathrm{e}}\,(\mathrm{cm}^{-3}) = 10^{8.5}$.

The advantage of the emissivity ratio method is that it does not depend on the ion population, and does not require knowledge of the temperature distribution. The disadvantage is that it cannot be used if the lines have a very different temperature dependence.

### $N_{\mathrm{e}}$ from other methods

The previous approaches can also be followed whenever lines from different elements are observed, as long as the relative chemical abundances are known, and the temperature of formation of the lines is also known. For example, an extension of the L-function method for an isothermal plasma at temperature $T_{0}$ is to plot the emissivity ratios *ER* of a series of lines, to measure the density:

103 $$\begin{aligned} { ER}_{ji}= {I_{\mathrm{ob}} \lambda _{ji} \over G(N_{\mathrm{e}}, T_{0})}\,{\mathrm{Const}}. \end{aligned}$$

These curves are effectively EM loci curves, scaled by a constant (see Fig. 68 below for an example).

If the volume of the emitting plasma can be estimated, then an average value of the electron density $\langle N_{\mathrm{e}} \rangle$ can be deduced from the total emission measure. In the case of radiance observations:

104 $$\begin{aligned} \langle N_e^2 \rangle = { EM}/dh, \end{aligned}$$

where ${ dh}$ is the path length of the emitting plasma and EM is the column emission measure. Alternatively, the path length ${ dh}$ can be estimated from the column EM once the average electron density $\langle N_{\mathrm{e}} \rangle$ is measured from a line ratio. In most cases, when the values of $\langle N_{\mathrm{e}}^2 \rangle$ are compared to the $\langle N_{\mathrm{e}} \rangle ^2$ values which are calculated from direct measurements of the average (along the line of sight) density $\langle N_{\mathrm{e}} \rangle$ in the transition region, large discrepancies are often found.

Dere et al. (1987) used HRTS observations to obtain EM estimates from C iv lines and densities from O iv (both formed at around $10^5\,\hbox {K}$). The resulting path lengths were in the range 0.1–10 km, which is much smaller than the observed sizes of the spicular structures at the HRTS resolution (2400 km). These measurements suggest that the transition region has a filamentary structure, with most of the plasma occupying only a small fraction (0.01–0.00001—the filling factor) of the observed volume. However, alternative interpretations have also been proposed (see, e.g., Judge 2000).

In principle, the EM could also be obtained from the free–free and the free–bound continuum. From these EM values, $N_{\mathrm{e}}$ can then be estimated. Added uncertainties are that the continua depend on the ion and elemental abundances, which are normally unknown.

The broadening of the neutral hydrogen lines depends on thermal motions, but also on the Stark effect, and on the collisions with free electrons. It therefore depends, among other things, on $N_{\mathrm{e}}$, so in principle the broadening can be used to estimate $N_{\mathrm{e}}$ (see, e.g., Kurochka and Maslennikova 1970).

The method has been used with Skylab spectra to get densities via Stark broadening in Rosenberg et al. (1977) (quiet Sun and polar hole) and by Feldman and Doschek (1977c) (active regions).

### $N_{\mathrm{e}}$ from specific ions

There are a large number of papers where density diagnostics are discussed, from both a theoretical and/or observational point of view. In what follows we review the main diagnostics in the EUV/UV, providing lists of the strongest unblended line ratios that have a strong density sensitivity. We flag a line when there is a known blend.

The lists are not comprehensive, i.e., there are always other options/combinations of lines. The choice mostly depends on the spectral range covered by an instrument, and its resolving power. Indeed most of the lines turn out to be blended in medium (and some even in high) resolution spectra.

The improvement in atomic calculations has gone hand in hand with better observations. For many ions, electron densities obtained with the earlier atomic data were inaccurate. The atomic data for the simpler ions reached sufficient accuracy in the 1980s. However, the atomic data for the most complex isoelectronic sequences and the iron coronal ions (which are in many ways the most important ones) have only recently been available to a high accuracy.

Earlier reviews on spectroscopic diagnostics are given in Feldman and Doschek (1977b), Feldman et al. (1978a, 1992), Dere et al. (1979), Dere and Mason (1981), Feldman (1981), Doschek (1985) and Mason and Monsignori Fossi (1994). They cover many spectral ranges of earlier instruments.

A comprehensive review of the density diagnostics available across the wide SERTS-89 spectral range (235–450 Å) is given by Young et al. (1998). A review of the density diagnostics available to SoHO CDS is given in Del Zanna (1999). The density diagnostics of coronal lines available to SoHO SUMER have been discussed in several papers, see, e.g., Laming et al. (1997) and Mohan et al. (2003). The density diagnostics for the coronal lines observed by Hinode EIS were reviewed by Del Zanna (2012b).

Keenan (1996) provided an extended bibliographical list of previous work. There are also several papers and reviews that discuss atomic data and diagnostics within an isoelectronic sequence. Some of these are cited below.

#### $N_{\mathrm{e}}$ from He-like ions

**Fig. 62:**
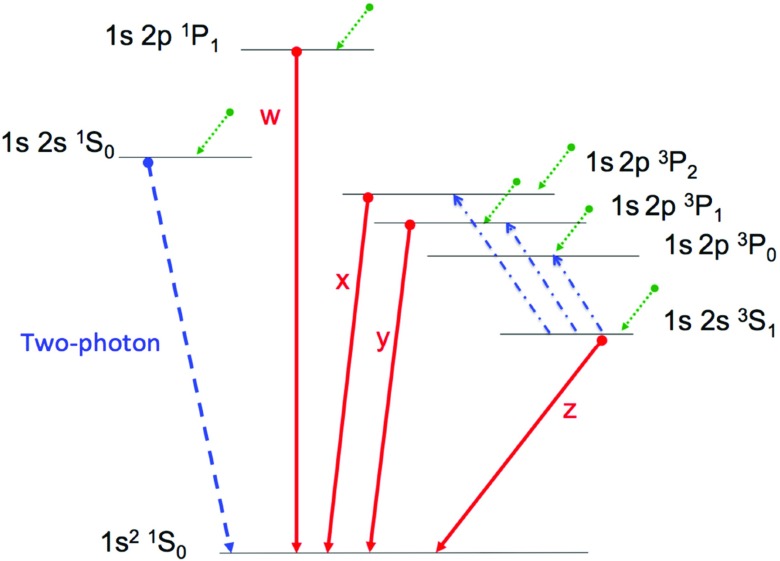
A diagram (not to scale) of the main transitions in He-like ions. The red downward arrows indicate the main spectral lines, while the blue dashed line indicates the two-photon decay. The downward green dotted lines indicate contributions from cascading/recombination. The blue dot-dash lines show where photoexcitation is an important excitation mechanism

Gabriel and Jordan (1969) showed that the ratios *R* of the forbidden *z* with the intercombination lines $x+y$ (see Fig. 62) in the He-like C, O, Ne, Mg are mostly density-sensitive, with little temperature sensitivity. Lines from these ions are observed in the X-rays, as shown in Table 6. The *x* line is a magnetic quadrupole (M2) transition, while the *y* is an electric dipole one (E1). The cross-section for excitation to their upper levels is relatively easy to calculate accurately. Photoexcitation and cascading from higher levels (and recombination) need to be taken into account as they can significantly affect the upper level populations. The excitation rate to the forbidden *z* magnetic dipole (M1) transition is more difficult to calculate accurately, as it is very sensitive the effects of the resonances. We note that in previous literature, resonance effects were sometimes added to the DW calculations. However, when results have been compared to more recent full CC *R* matrix calculations, significant differences were found (see Badnell et al. 2016).

**Table 6:** He-like main lines

Ion	*w*	*x*	*y*	*z*	Log *T*
	$1{s}^2\,{}^1\hbox {S}_{0}{-}1{s}\,2{p}\,{}^1\hbox {P}_{1}$	$1{s}^2\,{}^1\hbox {S}_{0}{-}1{s}\,2{p}\,{}^3\hbox {P}_{2}$	$1{s}^2\,{}^1\hbox {S}_{0}{-}1{s}\,2{p}\,{}^3\hbox {P}_{1}$	$1{s}^2\,{}^1\hbox {S}_{0}{-}1{s}\,2{s}\,{}^3\hbox {S}_{1}$	
C v	40.268	40.728	40.731	41.472	5.5
N vi	28.787	29.082	29.084	29.534	5.75
O vii	21.602	21.804	21.807	22.101	6.0
Ne ix	13.447 (bl Fe xix)	13.550	13.553 (bl Fe xix)	13.699	6.2
Mg xi	9.169	9.228 (bl Fe xxiii)	9.231	9.314	6.45
Si xiii	6.648	6.685	6.688	6.740	6.65
S xv	5.039	5.063	5.066	5.101	6.65
Ca xix	3.177	3.189	3.193	3.211	7.15
Fe xxv	1.8504	1.8554	1.8595	1.8682	7.5

The log *T* (K) values indicate the temperatures of peak ion abundance in equilibrium, although we note that the temperature of formation can be quite different. Wavelengths are in Å and are from CHIANTI v.8

**Fig. 63:**
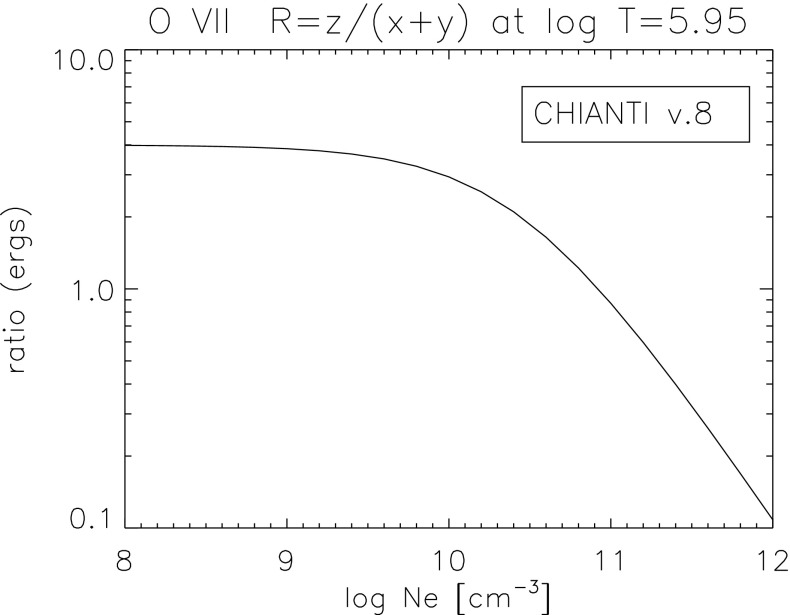
*R* ratio for the He-like O vii

One problem is that the range of densities for which the line ratios are sensitive is higher than usual solar flare densities for all ions, with the exception of O vii. Figure 63 shows the *R* ratio for this ion. Another non-trival issue is the fact that satellite lines can contribute to the observed intensities of the lines.

Another problem is that the lines need to be observed with high-resolution spectrometers. One example is the flare observation obtained by a sounding rocket, used to obtain densities by Brown et al. (1986). Another one is the SOLEX observation reported by McKenzie et al. (1980a) and Doschek et al. (1981b). For more details on the He-like ions, see the review by Porquet et al. (2010).

#### $N_{\mathrm{e}}$ from Be-like ions

Table 7 lists the lines most useful for density diagnostics in Be-like ions. Lines from the Be-like C iii can be used to measure densities (see e.g., Munro et al. 1971; Jordan 1974; Dupree et al. 1976; Dufton et al. 1978).

**Table 7:** Be-like density diagnostics

Transition	C iii	O v	Ne vii	Mg ix	Al x	Si xi
	(4.8)	(5.4)	(5.7)	(6.0)	(6.1)	(6.2)
$2s\,2p\,^3P_{2}$–$2s\,3d^3D_{3}$		192.904				
$2s\,2p\,^3P_{2}$–$2s\,3d\,^3D_{2}$		192.911				
$2s\,2p\,^3P_{1}$–$2s\,3s\,^3S_{1}$		215.103				
$2s\,2p\,^3P_{2}$–$2s\,3s\,^3S_{1}$		215.245				
$2s\,2p\,^1P_{1}$–$2s\,3d\,^1D_{2}$		220.353				
$2s\,2p\,^1P_{1}$–$2s\,3s\,^1S_{0}$		248.460				
$2s^2\,{}^1S_{0}$–$2s\,2p\,^1P_{1}$ (a)	977.020	629.732				
$2s\,2p\,^3P_{1}$–$2p^2\,{}^3P_{2}$ (b)	1174.933	758.677		439.176		
$2s\,2p\,^3P_{0}$–$2p^2\,{}^3P_{1}$ (b)	1175.263	759.442		441.199		
$2s\,2p\,^3P_{1}$–$2p^2\,{}^3P_{1}$ (b)	1175.590	760.227		443.404		
$2s\,2p\,^3P_{2}$–$2p^2\,{}^3P_{2}$ (b)	1175.711	760.446		443.973		
$2s\,2p\,^3P_{1}$–$2p^2\,{}^3P_{0}$ (c)	1175.987	761.128		445.981		
$2s\,2p\,^3P_{2}$–$2p^2\,{}^3P_{1}$ (b)	1176.370	762.004		448.294		
$2s^2\,{}^1S_{0}$–$2s\,2p\,^3P_{1}$	1908.734	1218.344	895.18	706.06	637.76	580.91
$2s\,2p\,^1P_{1}$–$2p^2\,{}^1D_{2}$	2296.870	1371.296				
$2s^2\,{}^1S_{0}$–$2s\,2p\,^3P_{2}$			887.28	693.98	623.32	564.02

The log $T_{\mathrm{e}}$ (K) values (in parenthesis) indicate the temperatures of peak ion abundance in equilibrium. Wavelengths are in Å. The density can be measured from, e.g., the ratios of the resonance (a) line with any of the multiplet (b, c), or from within the multiplet between line (c) and any of the (b) lines

**Fig. 64:**
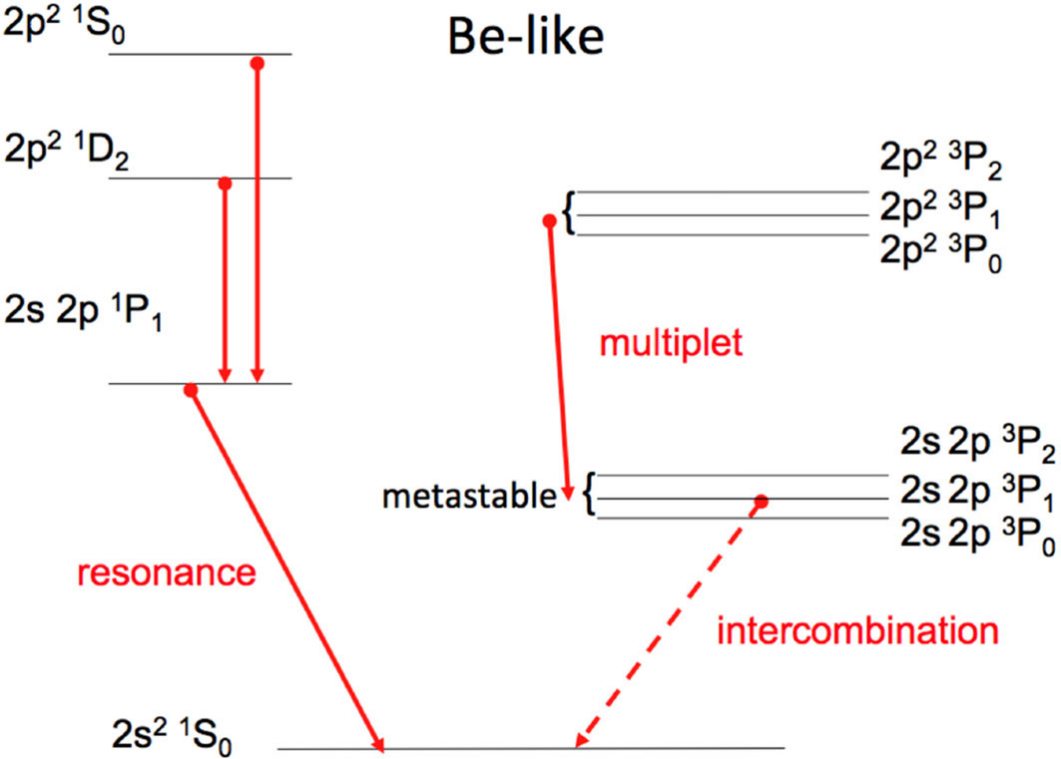
A diagram (not to scale) of the main levels in Be-like ions. The red downward arrows indicate the main spectral lines

The first excited configuration for the Be-like ions is $2s\,2p$, which has $^3\hbox {P}$ and $^1\hbox {P}_{1}$ levels (see Fig. 64). The $^3\hbox {P}$ levels are metastables, so ratios of lines populated from these levels such as the $2s\,2p ^3\hbox {P}{-}2{p}^2\,{}^3\hbox {P}$ multiplet at 1175 Å with the resonance $2{s}^2\,{}^1\hbox {S}_{0}{-}2{s}\,2{p}\,{}^1\hbox {P}_{1}$ line at 977.022 Å are density-sensitive. However, this diagnostic is only sensitive up to $N_{\mathrm{e}} = 10^{10}\,\mathrm{cm}^{-3}$, does not have a large variation with density, and is also temperature sensitive. Moreover, opacity effects can affect the resonance line. Another possibility is to use the ratio of the 1175.987 Å line with any other line of the multiplet, if high-resolution spectra are available. In this case, the ratios do not depend on the temperature.

The same ratio for O v involves the resonance 629.73 Å line and the multiplet at 760 Å. This is a good density diagnostic in the range $N_{\mathrm{e}} = 10^{10}{-}10^{12}\,\mathrm{cm}^{-3}$, although it is not ideal because of the strong temperature dependence. This ratio was studied by several authors, see e.g., Munro et al. (1971), Jordan (1974) and Dufton et al. (1978) for observations pre-SOHO. SOHO CDS and SUMER observed the O v $2s\,2p\,{}^3\hbox {P}{- }2{p}^2\,{}^3\hbox {P}$ transitions around 760 Å (760.444, 758.675, 762.002, 759.439, 760.225, 761.126 Å) and the 629.73 Å resonance line. The 761.126 Å line is a good density-diagnostic in conjunction with any of the other lines of the multiplet, because there is no dependence on temperature. SUMER was able to clearly separate the lines in the multiplet, allowing this ratio to be used to derive densities (see, e.g., Doschek et al. 1998a). The O v ratio of the 1218.390 and 1371.292 Å lines is an excellent diagnostic (because it has a small temperature dependence) at high densities, above $10^{11}\,\hbox {cm}^{-3}$, although the lines are weak. Other lines from O v suffer from various problems (continuum, blends) as summarised in Mariska (1992). The same transitions in N iv are in principle useful, but the lines are significantly blended (see, e.g., Dufton et al. 1979).

The other possibility is to use the forbidden lines which fall in the UV, as suggested by Munro et al. (1971). However, even there the diagnostics are not straightforward, and line ratios have to be carefully selected. For example, Doschek and Feldman (1977a) pointed out that the ratio of the C iii multiplet at 1176 Å with the intercombination line at 1909 Å cannot be used without a knowledge of the temperature structure of the atmosphere.

At densities well above $10^{10}\,\hbox {cm}^{-3}, n=3\rightarrow n=2$ transitions in O v in the 190–250 Å range are useful, as suggested by Widing et al. (1982). They have been observed with the Skylab NRL spectrograph, and some of them more recently with Hinode EIS.

The 192.750, 192.797, 192.801 Å lines of O v are self-blended and also blended with other transitions from Fe xi and Ca xvii, so are not listed in Table 7. The 192.904 and 192.911 are self-blended, and can be blended with Ca xvii during flares, when high temperatures are present. However, the ratio of the 192.9 Å self-blend with the 248.460 Å line is in principle a good density diagnostic for Hinode EIS, although the 248.460 Å line is very weak, especially after the instrument degradation, and there is some temperature dependence on this ratio. Other ratios of the lines listed in Table 7 are possible, but were only available to Skylab.

Another density ratio is the one between the intercombination $2{s}^2\,{}^1\hbox {S}_{0}{-}2{s}\,2{p}\,{}^3\hbox {P}_{1}$ line and the $2{s}^2\,{}^1\hbox {S}_{0}{-}2{s}\,2{p}\,{}^3\hbox {P}_{2}$. Such ratios have been observed by SoHO SUMER, see e.g., Laming et al. (1997). One of the most used ratios is the Mg ix 694 versus 706.06 Å. One problem with such a ratio is that both lines are relatively weak. The Mg ix lines were often observable by SUMER (off-limb), but the Al x and Si xi lines were only visible in more active spectra. Another problem is that the ratio is significantly temperature-sensitive.

For flare densities, two other line ratios are potentially useful. The first is the S xiii 308.95 versus 256.68 Å, sensitive up to $10^{11}\,\hbox {cm}^{-3}$, although the first line is normally very weak (at most 2.5% of the stronger line in ergs). The second is the Ar xv 266.23 versus 221.13 Å, sensitive in the $10^{10}{-}10^{12}\,\hbox {cm}^{-3}$ range, although again the weaker line is at most 2% of the stronger line (ratio value in ergs). The above S xiii and Ar xv lines have been observed in Skylab flare spectra.

**Fig. 65:**
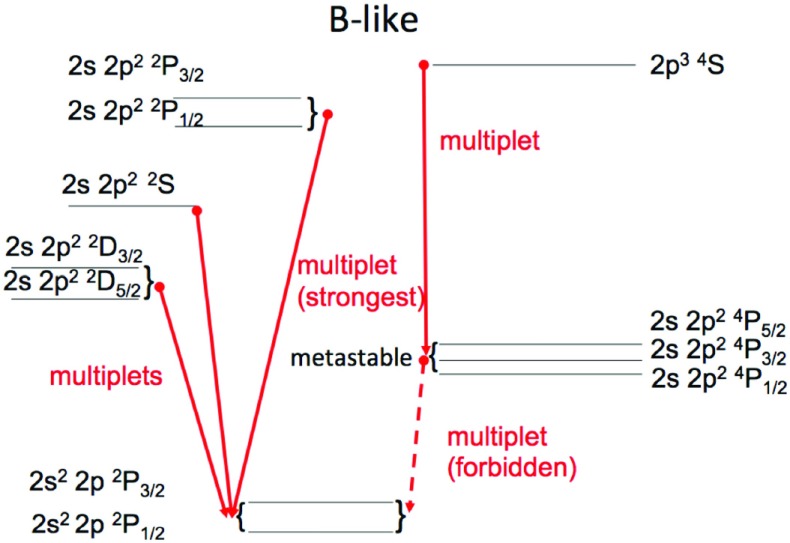
A diagram (not to scale) of the main levels in B-like ions. The red downward arrows indicate the main spectral lines

#### $N_{\mathrm{e}}$ from B-like ions

The boron sequence contains some particularly useful electron density sensitive line ratios. Na vii, Si x, S xii were studied by Flower and Nussbaumer (1975a). N iii, Ne vi, Mg viii and Si x were studied by Vernazza and Mason (1978). Nussbaumer and Storey (1979) studied N iii. The ground levels are $2{s}^2\,2p\,^2\hbox {P}_{1/2,3/2}$ and the first excited levels are $2s\,2{p}^2\,{}^4\hbox {P}_{1/2,3/2,5/2}$ which are metastable (see Fig. 65). The second excited term is the $2s\,2{p}^2\,{}^2\hbox {D}$ which produces allowed decays to the ground term. As in the previous cases, the density sensitivity involves levels that are collisionally excited from the metastable levels, for example the decays from $2{p}^3\,{}^4\hbox {S}$. Ratios of this type are listed in the first line of Table 8. The N iii 772/989 Å is a good diagnostic. The O iv 625.9 Å line is also an excellent diagnostic (Doyle et al. 1985a), e.g., in combination with the 790.1 Å line, as the ratio varies by a factor of 35. However, this line was blended in Skylab spectra with the Mg x 624.9 Å line, which is normally much stronger. This O iv 625.9 Å line, at the SoHO CDS NIS resolution, becomes measurable (even though it is in the wing of the Mg x 624.9 Å line) especially when the Mg x intensity is reduced, as in coronal holes (Del Zanna 1999) or in transition region brightenings (Young and Mason 1997). The ratio of the 625.9 Å line with any other O iv line in the SOHO CDS wavelength range (553.329, 554.513, 555.263, 608.4 Å) is also density-sensitive. The analogous Ne vi 454/562 Å ratio is not very useful as the 454 Å line is very weak.

**Table 8:** B-like density diagnostics

Transitions				
Ion	$\lambda _1$ (Å)	$\lambda _2$ (Å)	$\mathrm{Log}\,T_{\mathrm{e}}$	$\mathrm{Log}\,N_{\mathrm{e}}$
$$2{s}\,2{p}^{2}\,{}^4\hbox {P}_{3/2,5/2}{-}2{p}^{3}\,{}^4\hbox {S}_{3/2} \quad 2{s}^{2}\,2{p}\,{}^2\hbox {P}_{3/2}{-}2{s}\,2{p}^{2}\,{}^2\hbox {D}_{5/2,3/2}$$				
N iii	772	991.5	4.9	9–11
O iv	625	790.1	5.1	9–12
$$2{s}^{2}\,2{p}\,{}^2\hbox {P}_{3/2}{-}2{s}\,2{p}^{2}\,{}^2\hbox {D}_{5/2,3/2} \quad 2{s}^{2}\,2{p}\,{}^2\hbox {P}_{1/2}{-}2{s}\,2{p}^{2}\,{}^2\hbox {D}_{3/2}$$				
Mg viii	436.73	430.45	5.9	7–9
Si x	356.03	347.40	6.0	8–10
S xii	299.54	288.42	6.4	8–11
Ar xiv	257.44 (bl)	243.8 (bl)		9–12
Ca xvi	224.54	208.57 (bl Ca xv)	6.7	10–12
Fe xxii	156.0	135.79	7.2	11.5–15
$$2{s}^{2}\,2{p}\,{}^2\hbox {P}_{3/2}{-}2{s}\,2{p}^{2}\,{}^2\hbox {P}_{3/2} \quad 2{s}^{2}\,2{p}\,{}^2\hbox {P}_{1/2}{-}2{s}\,2{p}^{2}\,{}^2\hbox {S}_{1/2}$$				
Mg viii	315.01	335.23 (bl)	5.9	7–8.5
Si x	258.37	271.99	6.0	8–10
S xii	218.20	227.50	6.4	9–11
Ar xiv 187.97 (bl)	194.41 (w)	9–12		
Ca xvi	164.17	168.85	6.7	10–13
Fe xxii	114.41	117.15 (bl Fe xxi)	7.2	11.5–15
$$2{s}^{2}\,2{p}\,{}^2\hbox {P}_{1/2}{-}2{s}^{2}\,4\hbox {d}\,{}^2\hbox {D}_{3/2} \quad 2{s}^{2}\,2{p}\,{}^2\hbox {P}_{3/2}{-}2{s}^{2}\,4\hbox {d}\,{}^2\hbox {D}_{5/2,3/2}$$				
Fe xxii	8.976	9.075	7.1	12.5–15
$$2{s}^{2}\,2{p}\,{}^2\hbox {P}_{3/2}{-}2{s}\,2{p}^{2}\,{}^4\hbox {P}_{5/2} \quad 2{s}^{2}\,2{p}\,{}^2\hbox {P}_{3/2}{-}2{s}\,2{p}^{2}\,{}^4\hbox {P}_{3/2}$$				
Na vii	872.12	880.33	5.8	6–8
Mg viii	772.28	782.36	5.9	6.5–8.5
Al ix	691.54	703.66	5.9	7–9
Si x	624.70	638.94	6.0	8–10
$$2{s}^{2}\,2{p}\,{}^2\hbox {P}_{3/2}{-}2{s}\,2{p}^{2}\,{}^4\hbox {P}_{5/2} \quad 2{s}^{2}\,2{p}\,{}^2\hbox {P}_{3/2}{-}2{s}\,2{p}^{2}\,{}^4\hbox {P}_{1/2}$$				
Na vii	872.12	885.98	5.8	6–8
Mg viii	772.28	789.44	5.9	6.5–8.5
Al ix	691.54	712.23	5.9	7–9
Si x	624.70	649.19	6.0	8–10

$T_{\mathrm{e}}$ (K) indicates the temperature of peak ion abundance in equilibrium. The $N_{\mathrm{e}}$ values ($\hbox {cm}^{-3}$) indicate the approximate range of densities where the ratios are usable

**Fig. 66:**
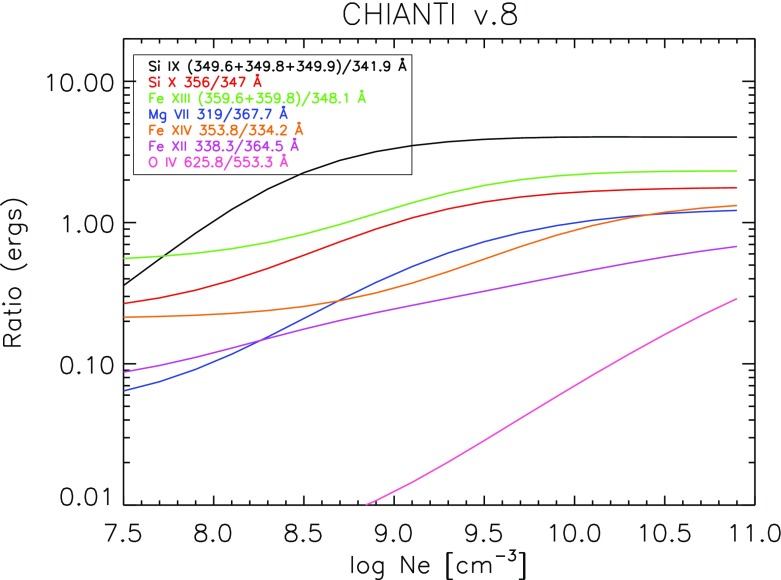
The main diagnostic ratios available to the SoHO CDS NIS. The atomic data are from CHIANTI version 8

Other types of diagnostic ratios are available. For example, ratios of allowed lines, where one decays to the ground state and one to the metastable level $2{s}^2\,2p\,^2\hbox {P}_{3/2}$. Table 8 shows two examples. The first one is excellent, since the lines are close in wavelength. Very good diagnostics are offered by Mg viii, Si x, and S xii. The Mg viii 436.7 (436.735 $+$ 436.672) Å/430.465 Å and the Si x 356.0 (self-blend)/347.40 ratios were among the best diagnostic ratios available for the SOHO GIS and NIS. They are shown, together with a few others, in Fig. 66.

One of the Ca xvi lines (208.57 Å) is blended with a Ca xv 208.70 Å even at the Hinode EIS spectral resolution. At flare temperatures, Fe xxii soft X-ray lines within the $2{s}^2\,2{p}{-}2{s}\,2{p}^2$ transition array are useful density diagnostics (Doschek et al. 1973; Mason and Storey 1980; Mason et al. 1984; Del Zanna and Woods 2013), but at rather high densities (above $10^{11.5}\,\hbox {cm}^{-3}$), i.e., higher than those normally found in solar flares. Other ions produce either weak or blended lines, as is the case for the Ar xiv lines.

Laming et al. (1997) suggested the use of two other diagnostic ratios, one is the ratio of the decays from the $2s\,2{p}^2\,{}^4\hbox {P}_{3/2,5/2}$ levels to the $2{s}^2\,2p\,{}^2\hbox {P}_{3/2}$, while the other is the ratio of the decays from the $2s\,2{p}^2\,{}^4\hbox {P}_{1/2,5/2}$ levels to the $2{s}^2\,2p\,^2\hbox {P}_{3/2}$. The lines are relatively close in wavelength but are quite weak, although lines from a few ions have been observed with SoHO SUMER.

A few diagnostics in the X-rays (around 10 Å) are available, from Fe xxii $2{s}^2\,2{p}{-}2{s}^2$ 4d transitions (cf. Table 8 and Fawcett et al. 1987; Phillips et al. 1996). However, these lines are weak and only sensitive to high densities.

Ratios of forbidden transitions between the metastable $2s\,2{p}^2\,{}^4\hbox {P}$ and the ground configuration $2{s}^2\,2p\,^2\hbox {P}$ in N iii are also density-sensitive in the $10^{9}{-}10^{11}\,\hbox {cm}^{-3}$ range (Nussbaumer and Storey 1979). It should be noted that these ratios are virtually independent of temperature. However, the variations are not large (a factor of 2.5) and some lines are affected by blending (see e.g., Mariska 1992). The same ratios for O iv fall around 1400 Å and have been used extensively. They are discussed below.

**O** **iv** **1400 Å multiplet**

The O iv multiplet around 1400 Å has been studied by Flower and Nussbaumer (1975b) and many other authors. Feldman and Doschek (1979) also discussed the potential of N III and O iv intersystem multiplets as density diagnostics.

Several solar instruments have observed the O iv lines: Skylab S082B, HRTS, SMM UVSP (see, e.g., Hayes and Shine 1987), SOHO SUMER (see, e.g., Teriaca et al. 2001), and more recently IRIS (see, e.g., Dudík et al. 2014a).

Table 9 lists the main diagnostic lines and the densities that can be measured. We note that some discrepancies in the wavelengths of these lines can be found in the literature. This table lists the wavelength values as reviewed in Polito et al. (2016a), which are mainly based on Skylab observations at the limb, as reported by Sandlin et al. (1986), with an accuracy of about 0.005 Å, and laboratory measurements from Bromander (1969).

The densities obtained from O iv have been particularly important because they have been used to obtain a very small filling factor (< 1%) for the transition region emission, corresponding to a path length of around 10 km (see, e.g., Dere et al. 1987, using HRTS observations).

**Table 9:** O iv transitions commonly used to measure densities in the $\log \,N_{\mathrm{e}}\,(\hbox {cm}^{-3}) = 9.5{-}11.5$ range (wavelengths from Polito et al. 2016a)

Transitions	$\lambda$ (Å)
$2{s}^2\,2{p}\,{}^2\hbox {P}_{1/2}{-}2{s}\,2{p}^{2}\,{}^4\hbox {P}_{3/2}$	1397.226
$2{s}^2\,2{p}\,{}^2\hbox {P}_{1/2}{-}2{s}\,2{p}^{2}\,{}^4\hbox {P}_{1/2}$	1399.776
$2{s}^2\,2{p}\,{}^2\hbox {P}_{3/2}{-}2{s}\,2{p}^{2}\,{}^4\hbox {P}_{5/2}$	1401.163
$2{s}^2\,2{p}\,{}^2\hbox {P}_{3/2}{-}2{s}\,2{p}^{2}\,{}^4\hbox {P}_{3/2}$	1404.806 (bl S iv)
$2{s}^2\,2{p}\,{}^2\hbox {P}_{3/2}{-}2{s}\,2{p}^{2}\,{}^4\hbox {P}_{1/2}$	1407.384

**Fig. 67:**
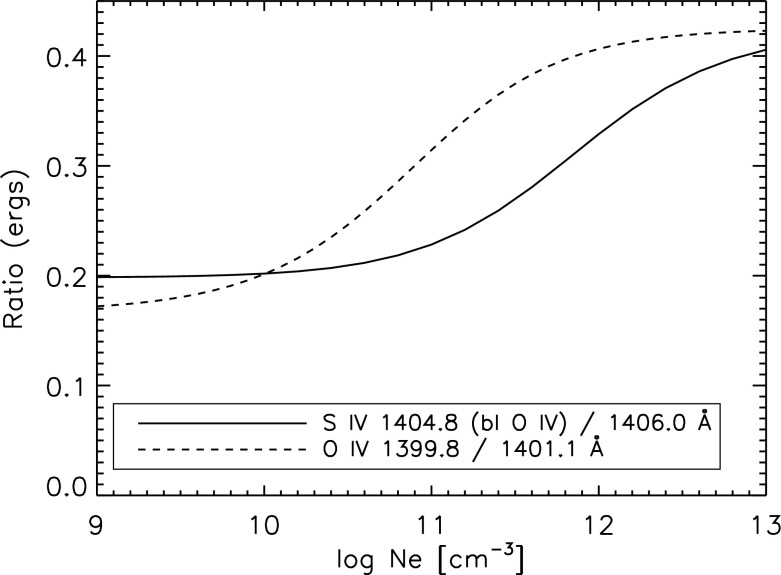
Electron density sensitivity of the O iv, S iv lines, observed with IRIS

One problem is that the O iv lines are often weak and blended. The 1397.22 and 1399.78 Å lines are particularly weak. The ratios that include the 1401.16 Å line have a small density sensitivity, as shown in Fig. 67 and already noted by Feldman and Doschek (1979). Ratios involving the 1404.78 Å line are better; however, the 1404.78 Å line is a well-known blend with S iv (1404.8 Å). It is common practice to use the S iv theoretical ratio of the 1404.8 Å and 1406.01 Å lines to infer the contribution of the 1404.81 Å line to the observed blend with O iv, however the S iv ratio is density sensitive as well, so deblending the line is not straightforward. Another issue is that the O iv lines within the multiplet have a density sensitivity only up to about $10^{11}\,\hbox {cm}^{-3}$, as Fig. 67 shows. On the other hand, the S iv lines are much better since flare densities up to about $10^{13}\,\hbox {cm}^{-3}$ can be measured.

The O iv and S iv density sensitive ratios have been analysed by several authors in both solar and stellar spectra (cf. Keenan et al. 1994c; Cook et al. 1995; Del Zanna et al. 2002; Polito et al. 2016a). Inconsistencies between densities of O iv and S iv have been noted (Cook et al. 1995). Some of the discrepancies were subsequently resolved by Keenan et al. (2002a) by taking into account line blending and the then new atomic data. Nevertheless, some inconsistencies still remain, in particular regarding the 1404.8 Å blend, which is about 30% stronger than predicted, as discussed in Del Zanna et al. (2002).

Recently, several studies have been carried out with IRIS. Peter et al. (2014) used the O iv lines in combination with the Si iv lines to find very high densities (about $10^{13}\,\hbox {cm}^{-3}$ or higher) in the so called IRIS plasma ‘bombs’. Several problems with the use of such diagnostic were however pointed out by Judge (2015). In fact, the O iv and Si iv ratios are sensitive to changes in the temperature and the relative abundances of O and Si. In addition, high density effects such as the suppression of the dielectronic recombination rate can shift the formation temperature of these ions (Polito et al. 2016a). Judge (2015) also mentioned the fact that the intensity of the Si iv lines is known to be anomalous.

The important piece of work carried out by Hayes and Shine (1987) using SMM/UVSP, where the densities obtained from the two methods (O iv vs. Si iv and O iv line ratios) were compared is often overlooked in the literature. Indeed, they found a large discrepancy of about an order of magnitude, with a large scatter. Large discrepancies with the use of these two methods (O iv line ratios vs. the intensities of the O iv vs. Si iv lines) were also found using IRIS data by Polito et al. (2016a). The differences vary substantially depending on the source region (e.g., AR plage or solar flare). The direct O iv line ratio method clearly provides more accurate measurements, although the variations in the densities can also be roughly estimated with the other method (using the ratio of lines from O iv versus Si iv, see Doschek et al. 2016).

**Fig. 68:**
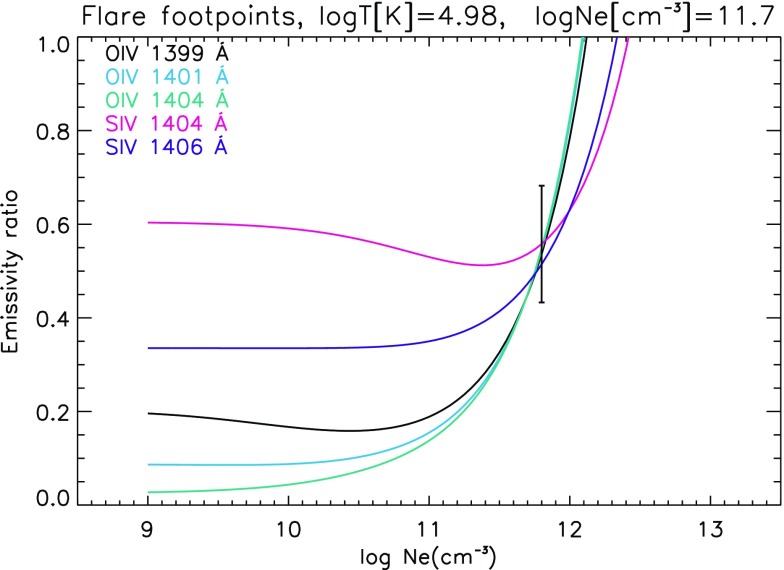
Electron densities obtained from the emissivity ratio (*ER*) curves of S iv and O iv IRIS lines observed in an active region (adapted from Polito et al. 2016a). The curves are obtained assuming that both O iv and S iv are formed at the same isothermal temperature of log $T\,(\mathrm{K})=4.98$

Regarding the discrepancies between densities found using the O iv and S iv lines, Polito et al. (2016a) has shown that agreement is found if the plasma is assumed to be isothermal. Figure 68 shows the emissivity ratio (*ER*) curves obtained assuming that both O iv and S iv are formed at the same isothermal temperature.

Ideally one would need to have an independent way to measure the plasma temperature, i.e., the ion charge state distribution. It is well known that various effects can shift the temperature of formation of the O iv lines (as well as other TR lines), as discussed in Polito et al. (2016a). For example, taking into account high density effects on the ion balance shifts the ion formation towards lower temperatures (cf. Fig. 17). A similar effect is obtained with non-thermal electron distributions (Dudík et al. 2014a). Non-equilibrium ionisation can also significantly affect these ratios, as discussed in Sect. 6.

#### $N_{\mathrm{e}}$ from C-like ions

**Fig. 69:**
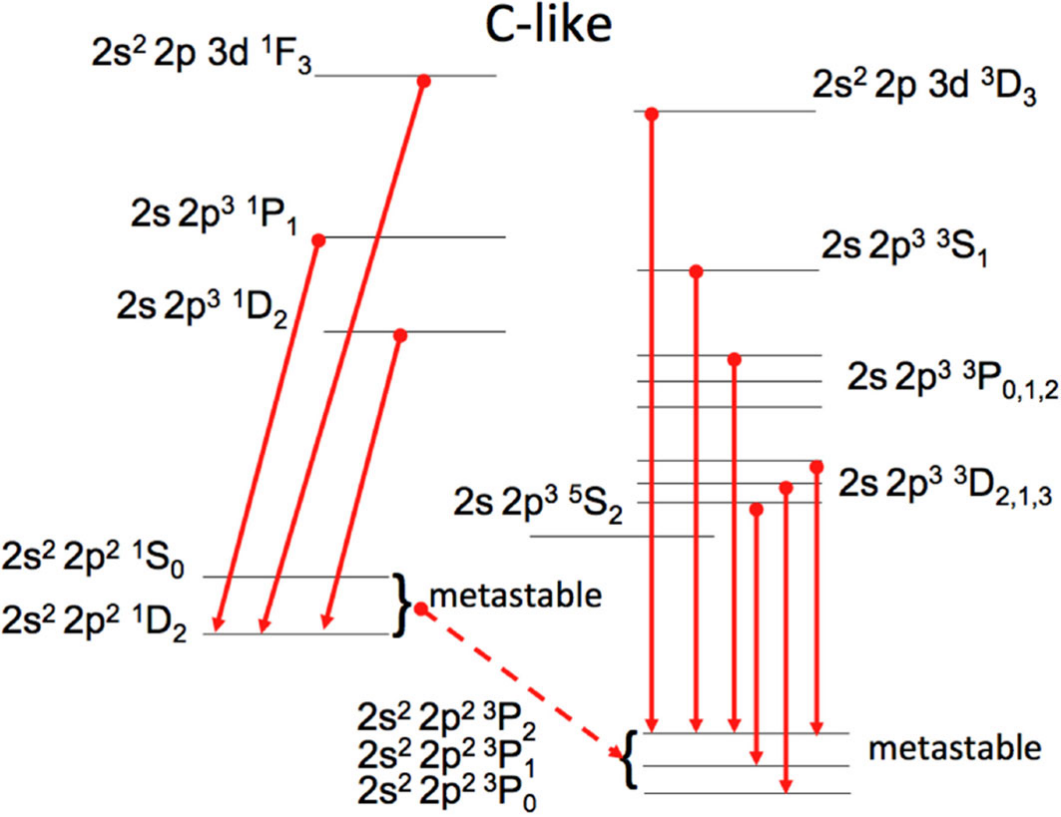
A diagram (not to scale) of the main levels in C-like ions. The red downward arrows indicate the main spectral lines

Transitions within the $2{s}^2\,2{p}^2{-}2{s}\,2{p}^3$ configuration in the C-like ions fall in the EUV and are useful density diagnostics. The density sensitivity arises from the fact that the four excited levels within the ground configuration are metastable (see Fig. 69).

Mason and Bhatia (1978) discussed in detail the diagnostic ratios for Mg vii, Si ix, and S xi. A notable comprehensive study was based on Skylab observations with the NRL slitless spectrograph (Dere et al. 1979). Several other authors later improved the atomic data and presented comparisons with observations. Table 10 lists the main lines.

Transitions within the $2{s}^2\,2{p}^2{-}2{s}^2\,2p\,3d$ configurations fall in the X-rays or soft X-rays (Brown et al. 1986) and have not been observed/used as much as the EUV lines. Only lines from Ca xv are shown in Table 10, although similar line ratios from other ions are also potentially useful.

A few diagnostics in the X-rays (around 10 Å) are available, from Fe xxi $2{s}^2\,2{p}^{2}{-}2{s}^2\,2p\,4d$ transitions (cf. Table 10 and Fawcett et al. 1987; Phillips et al. 1996).

The Mg vii 319.0/367.7 Å ratio was observed by SOHO CDS, however the 319.0 Å line is often blended with Ni xv and the 367.7 Å line is in the blue-wing of the Be-like Mg resonance line, so careful deblending is needed. There are other lines in the CDS spectral range, which could be used instead of the 367.7 Å line, but they are weaker and also blended. The ratio of the Mg vii 280.74 Å line with several other lines (e.g., 434.70 Å) observed by SOHO/GIS is also a good diagnostic. The Mg vii 280.74/278.41 Å ratio was observed by Hinode EIS, but there are various problems in deblending one of the lines, as discussed in Del Zanna (2012b). S xi has some excellent diagnostic ratios observed by Hinode EIS (Del Zanna 2012b).

**Table 10:** C-like density diagnostics (layout as in Table 8)

Transitions	Ion	$\lambda _1$ (Å)	$\lambda _2$ (Å)	$\mathrm{Log}\,T_{\mathrm{e}}$	$\mathrm{Log}\,N_{\mathrm{e}}$
$2{s}^{2}\,2{p}^{2}\,{}^3\hbox {P}_{2}{-}2{s}\,2{p}^{3}\,{}^3\hbox {D}_{3}/2{s}^{2}\,2{p}^{2}\,{}^3\hbox {P}_{0}{-}2{s}\,2{p}^{3}\,{}^3\hbox {D}_{1}$	Si ix	349.86	341.95	6.0	7–9
	S xi	291.58	281.41	6.3	8–12
	Ar xiii	248.70	236.28	6.45	9–11
	Ca xv	215.38	200.98	6.65	10–12
	Fe xxi	145.73	128.75	7.1	11–15
$2{s}^{2}\,2{p}^{2}\,{}^3\hbox {P}_{1}{-}2{s}\,2{p}^{3}\,{}^3\hbox {D}_{2}/2{s}^{2}\,2{p}^{2}\,{}^3\hbox {P}_{0}{-}2{s}\,2{p}^{3}\,{}^3\hbox {D}_{1}$	Si ix	345.12	341.95	6.0	7–9
	S xi	285.83 (bl O iv)	281.41	6.3	8–12
	Ar xiii	242.24	236.28	6.45	9–11
	Fe xxi	142.14 (bl)	128.73	7.1	11–15
$2{s}^{2}\,2{p}^{2}\,{}^3\hbox {P}_{2}{-}2{s}\,2{p}^{3}\,{}^3\hbox {P}_{2}/2{s}^{2}\,2{p}^{2}\,{}^3\hbox {P}_{0}{-}2{s}\,2{p}^{3}\,{}^3\hbox {D}_{1}$	Si ix	296.11 (sbl)	341.95	6.0	7–9
	S xi	246.9	281.41	6.3	8–12
	Ar xiii	210.47	236.28	6.45	9–11
	Ca xv	181.90	200.98	6.65	10–12
	Fe xxi	121.21 (bl)	128.73	7.1	11–15
$2{s}^{2}\,2{p}^{2}\,{}^1\hbox {D}_{2}{-}2{s}\,2{p}^{3}\,{}^1\hbox {D}_{2} 2{s}^{2}\,2{p}^{2}\,{}^3\hbox {P}_{2}{-}2{s}\,2{p}^{3}\,{}^3\hbox {P}_{2}$	Mg vii	319 (bl Ni xv)	367.7 (bl)	5.8	8–10
	Si ix	258.08 (bl Ar xiv)	296.11 (sbl)	6.0	9–12
	S xi	215.97 (bl Ni xv)	246.89	6.3	8–12
$2{s}^{2}\,2{p}^{2}\,{}^1\hbox {D}_{2}{-}2{s}\,2{p}^{3}\,{}^1\hbox {P}_{1}/2{s}^{2}\,2{p}^{2}\,{}^3\hbox {P}_{2}{-}2{s}\,2{p}^{3}\,{}^3\hbox {S}_{1}$	Mg vii	280.74	278.41 (bl Si vii)	5.8	8–10
	Si ix	227.36 (bl)	227.0	6.0	9–12
	S xi	190.35 (bl Fe xi)	191.26 (bl Fe ix)	6.3	8–12
$2{s}^{2}\,2{p}^{2}\,{}^1\hbox {D}_{2}{-}2{s}^{2}\,2{p}\,3{d}\,{}^1\hbox {F}_{3}/2{s}^{2}\,2{p}^{2}\,{}^3\hbox {P}_{2}{-}2{s}^{2}\,2{p}\,3{d}\,{}^3\hbox {D}_{3}$	Si ix	56.03	55.40	6.1	7–11
$2{s}^{2}\,2{p}^{2}\,{}^3\hbox {P}_{2}{-}2{s}^{2}\,2{p}\,3{d}\,{}^3\hbox {D}_{3}/2{s}^{2}\,2{p}^{2}\,{}^3\hbox {P}_{0}{-}2{s}^{2}\,2{p}\,3{d}\,{}^3\hbox {D}_{1}$	Ca xv	22.78	22.73	6.6	10–12
	Fe xxi	12.32	12.282	7.1	11–15
$2{s}^{2}\,2{p}^{2}\,{}^3\hbox {P}_{1}{-}2{s}^{2}\,2{p}\,4\hbox {d}\,{}^3\hbox {P}_{2}/2{s}^{2}\,2{p}^{2}\,{}^3\hbox {P}_{1}{-}2{s}^{2}\,2{p}\,4\hbox {d}\,{}^3\hbox {D}_{1}$	Fe xxi	9.548	9.542	7.1	11–15

The Si ix lines at 341.95, 345.10, 349.9 Å (a self-blend of three transitions) were observed by SOHO/NIS and have been used extensively to measure densities. The 227.36 Å line is blended with several transitions, as discussed by Dere et al. (1979).

At high densities, typical of flares, Ca xv lines are a good density diagnostic. Transitions within the $2{s}^2\,2{p}^{2}{-}2{s}\,2{p}^3$ fall in the EUV and are very good diagnostics, as shown by Dere et al. (1979). The ratios of the 215.38 or 181.90 Å lines versus the resonance EUV line at 200.97 Å are excellent density diagnostics around 4 MK at high densities typical of flares. Skylab observations showed consistent density values (Keenan et al. 1988a). The Ca xv 181.90/200.98 Å ratio is also useful for Hinode EIS observations, as shown by Warren et al. (2008a), who analysed the first EIS full-spectrum of a small flare, also first discussed in Del Zanna (2008b). In principle, there is another useful transition, at 208.72 Å. However, this line is blended with Fe xiii. Transitions within the $2{s}^2\,2{p}^2{-}2{s}^2\,2p\,3d$ configurations fall in the X-rays and are also useful density diagnostics, as shown by Brown et al. (1986). The lines are very close in wavelength, so high-resolution spectrometers are needed to resolve them.

A few Ar xiii lines are also potentially useful, although it is not clear if the problems reported by Dere et al. (1979) are due to blending or the atomic data.

Fe xxi soft X-ray lines offer excellent diagnostics for solar flares (Mason et al. 1979, 1984; Del Zanna and Woods 2013), although several of them are blended. The 121.21 or the 145.73 Å lines versus the resonance line at 128.75 Å are the best ratios.

**Table 11:** N-like density diagnostics (layout as in Table 8; w: weak, bl: blended)

Transitions	Ion	$\lambda _1$ (Å)	$\lambda _2$ (Å)	$\mathrm{Log}\,T$	$\mathrm{Log}\,N_{\mathrm{e}}$
$2{s}^{2}\,2{p}^{3}\,{}^4\hbox {S}_{3/2}{-} 2{s}^{2}\,2{p}^{3}\,{}^2\hbox {D}_{5/2}/2{s}^{2}\,2{p}^{3}\,{}^4\hbox {S}_{3/2}$–$2{s}^{2}\,2{p}^{3}\,{}^2\hbox {D}_{3/2}$	Si viii	1440.51	1445.73	5.9	6–8.5
	P ix	1307.57 w	1317.65 w	6.0	
	S x	1196.21	1212.93	6.2	7–9.5
	Ar xii	1018.89 (bl)	1054.57	6.4	
	K xiii	945.83 w	994.52 w	6.4	
	Ca xiv	880.35	943.70	6.5	
$2{s}^{2}\,2{p}^{3}\,{}^2\hbox {D}_{5/2}{-}2{s}\,2{p}^{4}\,{}^2\hbox {P}_{3/2}/2{s}^{2}\,2{p}^{3}\,{}^4\hbox {S}_{3/2}{-}2{s}\,2{p}^{4}\,{}^4\hbox {P}_{5/2}$	Si viii	216.92 (bl Fe ix)	319.84	5.9	7–12.5
	S x	180.73	264.23	6.1	7–12.5
	Ar xii	154.42 (?)	224.25	6.4	8.5–13
	Ca xiv	134.27 (?)	193.87	6.55	9.5–14
$2{s}^{2}\,2{p}^{3}\,{}^2\hbox {D}_{5/2}{-}2{s}\,2{p}^{4}\,{}^2\hbox {D}_{5/2}/2{s}^{2}\,2{p}^{3}\,{}^4\hbox {S}_{3/2}{-}2{s}\,2{p}^{4}\,{}^4\hbox {P}_{5/2}$	Si viii	277.06 (bl)	319.84		7–12.5
	S x	228.69	264.23	6.1	7–12.5
	Ar xii	193.70 (bl Fe x)	224.25	6.4	8.5–13
	Ca xiv	166.96 (?)	193.87	6.55	9.5–14
$2{s}^{2}\,2{p}^{3}\,{}^2\hbox {D}_{5/2}{-}2{s}^{2}\,2{p}^{2}\,3{d}\,{}^2\hbox {F}_{7/2}/2{s}^{2}\,2{p}^{3}\,{}^4\hbox {S}_{3/2}{-}2{s}^{2}\,2{p}^{2}\,3{d}\,{}^4\hbox {P}_{3/2}$	Fe xx	12.86	12.83	7.0	11.5–15

#### $N_{\mathrm{e}}$ from N-like ions

**Fig. 70:**
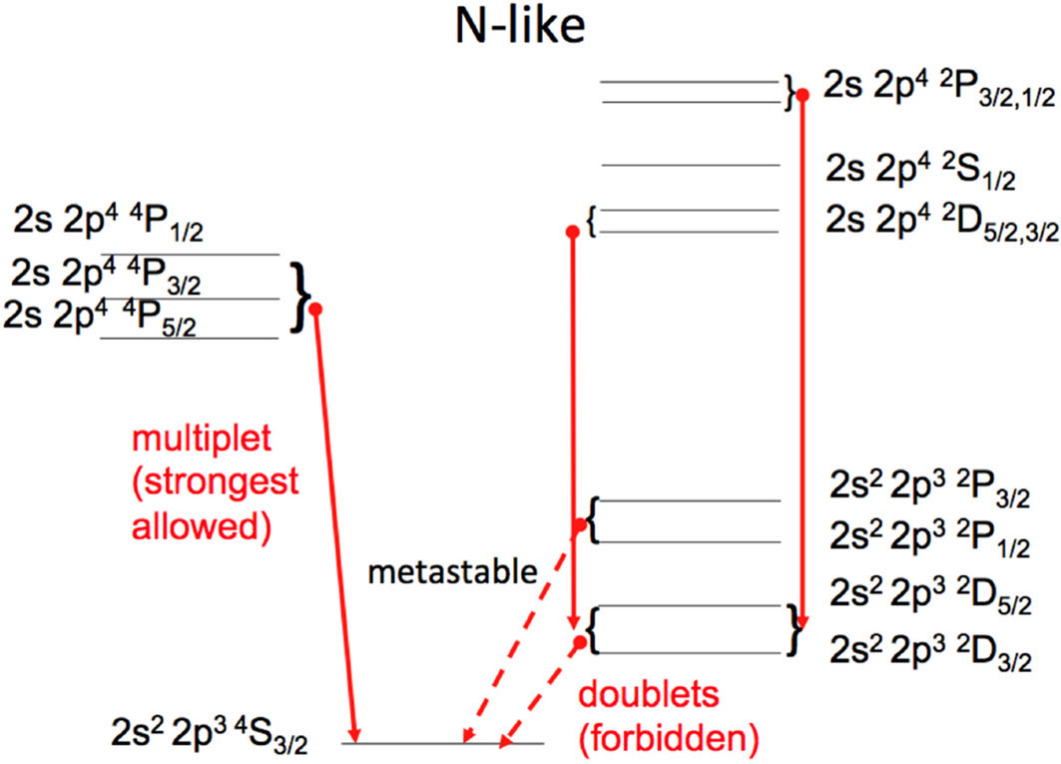
A diagram (not to scale) of the main levels in N-like ions. The red downward arrows indicate the main spectral lines

N-like ions offer several diagnostic ratios, as shown in Table 11. The density sensitivity arises because the four excited levels within the ground configuration $2{s}^2\,2{p}^3$ are metastable (see Fig. 70). Ratios of lines within the ground configuration are found in the UV. The advantage is that lines are close in wavelength and ratios are insensitive to temperature. They have been observed mainly with Skylab and SOHO SUMER. The disadvantage is that these forbidden lines are intrinsically very weak and in on-disk observations are virtually unobservable because they are blended with cool emission. The strongest lines providing density diagnostics are in the EUV for most ions in this sequence.

Feldman et al. (1978b) considered two line ratios within the ground configuration for Mg vi, Si VIII, S x, and Ar xii. These lines are intrinsically very weak and Skylab observations only allowed measurements of the S x 1213/1196 Å ratio. The main ratio is the $2{s}^2\,2{p}^3\,{}^4\hbox {S}_{3/2}{-}2{s}^2\,2{p}^3\,{}^2\hbox {D}_{5/2}/2{s}^2\,2{p}^3\,{}^4\hbox {S}_{3/2}{-}2{s}^2\,2{p}^3\,{}^2\hbox {D}_{3/2}$. The Si viii 1445/1440 Å ratio is also a good diagnostic for quiet Sun densities.

Indeed, the S x and Si viii ratios were used to obtain densities off-limb with SOHO SUMER (Doschek et al. 1997b). Mohan et al. (2003) extended the discussion of these forbidden lines within the ground configuration to more ions observed by SOHO SUMER. These other line ratios have rarely been observed because lines are weak and formed at higher temperatures. One example is the off-limb flare observations of SOHO SUMER reported by Landi et al. (2003), where lines from P, Ar, K, and Ca were observed. For active regions, probably the best ratio is the Ca xiv one, since the lines are relatively bright. The main ratios are shown in Table 11. Mohan et al. (2003) also listed a few weaker line ratios ($2{s}^2\,2{p}^3\,{}^4\hbox {S}_{3/2}{-}2{s}^2\,2{p}^3\,{}^2\hbox {P}_{3/2}/2{s}^2\,2{p}^3\,{}^4\hbox {S}_{3/2}{-}2{s}^2\,2{p}^3\,{}^2\hbox {P}_{1/2}$) from Al, Si, P, S, and Ar, but resulting densities were inconsistent. We point out that some inconsistencies for ions of this sequence are to be expected because accurate atomic data are still not available.

Another diagnostic possibility is to use EUV lines, which are intrinsically much stronger. The EUV lines of the N-like ions Mg vi, Si viii, S x, Ar xii, Ca xiv were discussed by Bhatia and Mason (1980) and later by several authors, including Dwivedi and Raju (1988). The main ratios are shown in Table 11. Dwivedi and Mohan (1995) used them to analyse SERTS observations.

The Mg vi lines are weak and blended. The Si viii lines would be useful but are normally blended in medium or low-resolution spectra. The S x 180.73/264.23 Å is an excellent diagnostic ratio observed by Hinode EIS (Del Zanna 2012b). The Ar xii lines are normally weak and blended. The Ca xiv 134.27 and 166.96 Å lines have not been observed in solar spectra, but in principle could be used simultaneously with the resonance line 193.87 Å to measure densities.

At flare temperatures, Fe xx lines offer the possibility of measuring electron densities above $10^{11}\,\hbox {cm}^{-3}$, with the 110.63/121.85 or 113.35/121.85 Å ratios (Mason et al. 1984; Del Zanna and Woods 2013). In fact, the strongest transition, $2{s}^2\,2{p}^3\,{}^4\hbox {S}_{3/2} {-}2{s}\,2{p}^4\,{}^4\hbox {P}_{5/2}$ falls at 132.85 Å, where this line is blended with the Fe xxiii resonance line.

#### $N_{\mathrm{e}}$ from O-like ions

Raju and Dwivedi (1978) discussed the diagnostics for Mg v, Si vii, S ix, Ar xi, however these ions produce weak lines in solar spectra.

The N iv 923 versus 765 Å ratio would be a good density diagnostic for the transition region, but these lines are blended, with an H i Lyman line and N iii, respectively.

#### $N_{\mathrm{e}}$ from Mg-like ions

**Fig. 71:**
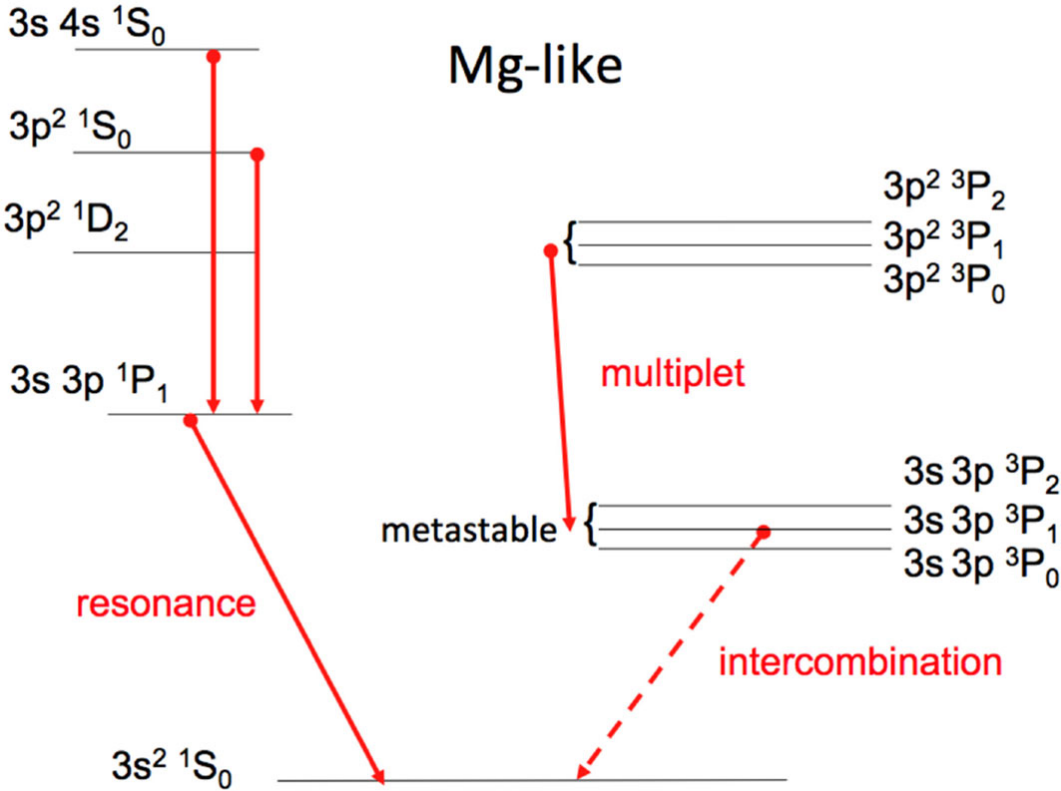
A diagram (not to scale) of the main levels in Mg-like ions. The red downward arrows indicate the main spectral lines

Ions in this sequence have a $3{s}^2\,{}^1\hbox {S}_{0}$ ground state, i.e., have a similar atomic structure to the Be-like (where the ground state is $2{s}^2\,{}^1\hbox {S}_{0}$), hence have similar density diagnostics (see Fig. 71). As in the Be-like case, the ratio of any lines within the $3s\,3p\,^3\hbox {P} {-}3{p}^2\,{}^3\hbox {P}$ multiplet with the resonance $3{s}^2\,{}^1\hbox {S}_{0}{-}3{s}\,3{p}\,{}^1\hbox {P}_{1}$ line is density-sensitive, although there is also a strong temperature dependence. The most famous ion is Si iii (see, e.g., Nicolas et al. 1977; Kjeldseth Moe and Nicolas 1977; Dufton et al. 1983a), where there is the additional problem, as in C iii, that the resonance line at 1206.499 Å is often affected by opacity. The ratio of the 1301.15 Å with any of the other lines within the multiplet is a good density diagnostic in the $N_{\mathrm{e}} = 10^{9}{-}10^{12}\,\mathrm{cm}^{-3}$ range as it varies by a factor of 5 and is not temperature dependent. Indeed Doschek (1997) used the Si iii 1296.73/1301.15 Å ratio to get electron densities from Skylab spectra. A recent review of the Si iii diagnostics can be found in Del Zanna et al. (2015c).

The equivalent ratios for S v are observed in the 850–860 Å range, but they are only sensitive to very high densities, above $10^{11}\,\mathrm{cm}^{-3}$. Dufton et al. (1986) suggested another density diagnostic, the ratio of the 1501.766 versus the 1199.136 Å lines for densities above $10^{12}\,\mathrm{cm}^{-3}$, although we note that the lines are weak and the ratio is strongly temperature sensitive.

Other ions in the sequence such as Ar vii and Ca ix produce weak lines, except Fe xv and Ni xvii which are discussed below (Table 12).

**Table 12:** Mg-like density diagnostics

Transition	Si iii	S v	Fe xv	Ni xvii
	4.7	5.1	6.3	6.5
$3{s}\,3{p}\,{}^3\hbox {P}_{2}{-}3{s}\,3{d}\,{}^3\hbox {D}_{3}$	1113.232 (sbl)		233.866	207.520
$3{s}\,3{p}\,{}^1\hbox {P}_{1}{-}3{s}\,3{d}\,{}^1\hbox {D}_{2}$	1206.557 (sbl)		243.794	215.910 (bl S xi)
$3{s}^{2}\,{}^1\hbox {S}_{0}{-}3{s}\,3{p}\,{}^1\hbox {P}_{1}$	1206.502 (sbl)		284.15	249.185
$3{s}\,3{p}\,{}^3\hbox {P}_{1}{-}3{p}^{2}\,{}^3\hbox {P}_{2}$	1294.548	849.240	292.275	251.952
$3{s}\,3{p}\,{}^3\hbox {P}_{0}{-}3{p}^{2}\,{}^3\hbox {P}_{1}$	1296.728	852.178	302.334	263.591
$3{s}\,3{p}\,{}^3\hbox {P}_{2}{-}3{p}^{2}\,{}^3\hbox {P}_{2}$	1298.948 (sbl)	854.770 (sbl)	304.894 (bl Fe xvii)	266.064
$3{s}\,3{p}\,{}^3\hbox {P}_{1}{-}3{p}^{2}\,{}^3\hbox {P}_{1}$	1298.894 (sbl)	854.870 (sbl)	307.747	269.416 (bl Fe xvii)
$3{s}\,3{p}\,{}^3\hbox {P}_{1}{-}3{p}^{2}\,{}^3\hbox {P}_{0}$	1301.151	857.828	317.597	281.469
$3{s}\,3{p}\,{}^3\hbox {P}_{2}{-}3{p}^{2}\,{}^3\hbox {P}_{1}$	1303.325	860.473	321.769	285.616
$3{s}\,3{p}\,{}^3\hbox {P}_{2}{-}3{p}^{2}\,{}^1\hbox {D}_{2}$	1447.191		327.033	289.743
$3{s}^{2}\,{}^1\hbox {S}_{0}{-}3{s}\,3{p}\,{}^3\hbox {P}_{1}$		1199.136		
$3{s}\,3{p}\,{}^1\hbox {P}_{1}{-}3{p}^{2}\,{}^1\hbox {D}_{2}$		1501.766		

The log $T_{\mathrm{e}}$ (K) values indicate the temperatures of peak ion abundance in equilibrium. Wavelengths are in Å

**Fe** **xv**

There are several diagnostics in the EUV within Fe xv (formed around 3 MK). Two ratios that would in principle be excellent are, among others, the 233.87/243.79 Å (see, e.g., Cowan and Widing 1973) and 321.78/327.03 Å ratios. The 233.87 Å line is collisionally populated from the $3s\,3p\,^3\hbox {P}$ which is metastable, while the 243.79 Å is not populated from a metastable, hence the density sensitivity of the ratio, for densities between $10^{9}$ and $10^{11}\,\mathrm{cm}^{-3}$. The lines are close in wavelength. The 321.78/327.03 Å ratio is particularly useful because it changes by a factor of 3 in the same density interval, and the lines are close.

However, significant problems in these ratios have been noted by several authors (see, e.g., Dere et al. 1979; Dufton et al. 1990; Bhatia and Mason 1997). It was not clear if the problem was due to blending of the lines or with the atomic data. Keenan et al. (2006a) suggested that the main problem is blending of virtually all the lines. The 321.78/327.03 Å ratio was however found to be reliable in Fernández-Menchero et al. (2015a), using the most accurate atomic data for this ion (UK APAP data, see Fernández-Menchero et al. 2014).

The analogous of the Si iii 1296.73/1301.15 Å ratio is the 317.597 versus 302.334 Å. It is not very sensitive as it varies by only a factor of 5 within $10^{8}$ and $10^{10}\,\mathrm{cm}^{-3}$. The ratio of the 304.98 Å line with e.g., the resonance line is also a good density diagnostic, but this line is blended with He ii. There are other possible diagnostics in the soft X-ray region (see, e.g., Bhatia et al. 1997), but the lines are relatively weak.

**Ni** **xvii**

The 207.520 versus 215.910 Å ratio is in principle a good density diagnostic in the $10^{9}{-}10^{12}\,\mathrm{cm}^{-3}$ range, but the 215.91 Å line is blended with S xi. Instead of the 215.91 Å line, the 289.743 Å could be used, although this line can become blended with a Fe xix 289.76 Å if high temperatures are present. Another option is to use the ratio of the 266.064 Å with the 289.743 Å line.

#### $N_{\mathrm{e}}$ from Al-like ions

**Fig. 72:**
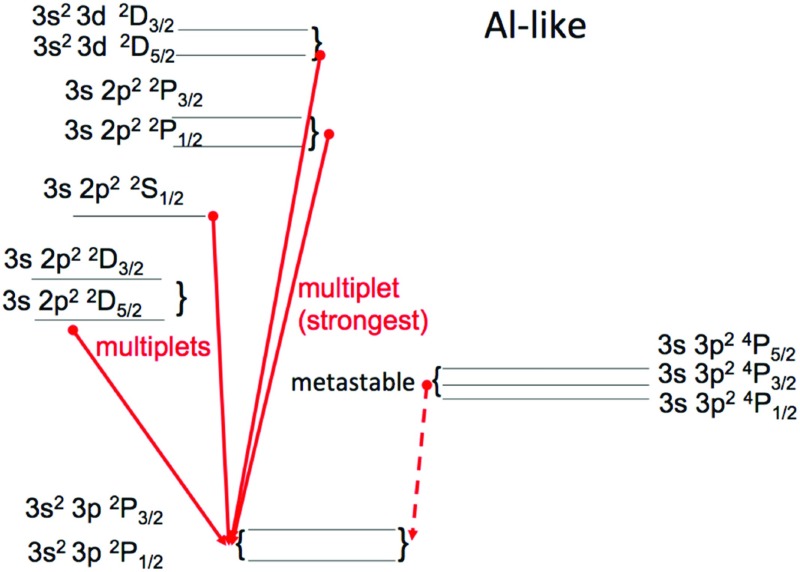
A diagram (not to scale) of the main levels in Al-like ions. The red downward arrows indicate the main spectral lines

The most widely used ion in this sequence is Fe xiv. The main density sensitivity is associated with decays of levels that are populated from the metastable $^2\hbox {P}_{3/2}$ within the ground configuration $3{s}^2\,3p$ (see Fig. 72, Table 13).

**Table 13:** Some of the common density diagnostics for Fe xiv

Transition	$\lambda$ (Å)	Observed	Log N$_{\mathrm{e}}$
$3{s}^{2}\,3{p}\,{}^2\hbox {P}_{3/2}{-}3{s}^{2}\,4\hbox {d}\,{}^{2}\hbox {D}_{5/2}$	59.58		
$3{s}^{2}\,3{p}\,{}^2\hbox {P}_{1/2}{-} 3{s}^{2}\,4\hbox {d}\,{}^2\hbox {D}_{3/2}$	58.96		9–11
$3{s}^{2}\,3{p}\,{}^2\hbox {P}_{3/2}{-}3{s}^{2}\,3{d}\,{}^2\hbox {D}_{5/2}$	219.12	Skylab	
$3{s}^{2}\,3{p}\,{}^2\hbox {P}_{3/2}{-}3{s}^{2}\,3{d}\,{}^2\hbox {D}_{3/2}$	220.08	Skylab	9–11
$3{s}^{2}\,3{p}\,{}^2\hbox {P}_{3/2}{-}3{s}\,3{p}^{2}\,{}^2\hbox {P}_{3/2}$	264.79 (bl)	EIS	9–11
$3{s}^{2}\,3{p}\,{}^2\hbox {P}_{1/2}{-}3{s}\,3{p}^{2}\,{}^2\hbox {P}_{3/2}$	252.20	EIS	9–11
$3{s}^{2}\,3{p}\,{}^2\hbox {P}_{1/2}{-}3{s}^{2}\,3{d}\,{}^2\hbox {D}_{3/2}$	211.32		
$3{s}^{2}\,3{p}\,{}^2\hbox {P}_{3/2}{-}3{s}\,3{p}^{2}\,{}^2\hbox {D}_{5/2}$	353.84 (bl)		9–11
$3{s}^{2}\,3{p}\,{}^2\hbox {P}_{3/2}{-}3{s}^{2}\,3{d}\,{}^2\hbox {D}_{5/2}$	334.18	CDS	

(bl) indicates a blend, while (sbl) indicates a self-blend of more than one transition from the same ion

Fe xiv (2 MK) offers many excellent density diagnostics involving strong lines that have been observed by several instruments. We note that reliable densities have been obtained only with recent atomic calculations (Storey et al. 2000).

The best Fe xiv diagnostic is the ratio of two strong lines close in wavelength, at 219.12/211.3 Å. Another good ratio is the 219.12/220.08 Å. There are several other good ratios observed by e.g., Hinode EIS (see Del Zanna 2012b for details), for example the 211.32 or 274.2 Å lines (which is blended with Si vii) versus the 264.79 Å (or 252.20 Å).

Other good ratios are found at longer wavelengths observed by e.g., SOHO/CDS (see, e.g., Mason et al. 1997) or SERTS (see, e.g., Young et al. 1998). The best one is the 353.84/334.18 Å, although it should be used with caution, since the Fe xiv 353.83 Å becomes blended with an Ar xvi 353.920 Å line in active region spectra. There are other possible diagnostics in the soft X-ray region, but lines are relatively weak.

The accuracy of the atomic data in CHIANTI v.8 to measure densities in EBIT spectra of Fe xiv has recently been confirmed by Weller et al. (2018).

After Fe xiv, the most widely used ion in the sequence is S iv. As discussed by, e.g., Dufton et al. (1982), the 1416.93/1406.06 and 1423.88/1416.93 Å ratios are in principle good density diagnostics. The S iv 1398.08, 1416.93 Å and 1423.88 Å lines are weak and are often not detected. Table 14 lists the main S iv transitions.

**Table 14:** The S iv transitions commonly used to measure densities (wavelengths from Del Zanna and Badnell 2016a)

Ion	Terms	Wavelength (Å)
S iv	$^2\hbox {P}_{1/2}{-}{}^4\hbox {P}_{3/2}$	1398.08
S iv	$^2\hbox {P}_{1/2}{-}{}^4\hbox {P}_{1/2}$	1404.85 (bl O iv)
S iv	$^2\hbox {P}_{3/2}{-}{}^4\hbox {P}_{5/2}$	1406.059
S iv	$^2\hbox {P}_{3/2}{-}{}^4\hbox {P}_{3/2}$	1416.928
S iv	$^2\hbox {P}_{3/2}{-}{}^4\hbox {P}_{1/2}$	1423.885

We note that, as in the O iv case, there has been some confusion in the literature regarding the wavelengths of the S iv lines. Table 14 lists the wavelengths as recommended by Del Zanna and Badnell (2016a), which are based on a reassessment of all the experimental data for this ion. Of particular importance is the wavelength of the line at 1404.85 Å, estimated to be very close to the wavelength of the O iv, 1404.82 Å. Indeed there is no indication of a wider separation of the two lines from the IRIS spectra.

As we have mentioned when discussing O iv, several inconsistencies in the S iv densities were reported (see, e.g., Cook et al. 1995). They were mostly due to the inaccuracy of the excitation data. The more recent calculations by Tayal (2000) resolved the main discrepancies. However, given the inconsistencies in the 1404.8 Å blend (Del Zanna et al. 2002), a new *R*-matrix scattering calculation was carried out by Del Zanna and Badnell (2016a). The new data corrected a few problems with the previous calculation, but still did not fix the problem with the 1404.8 Å blend, which could however be resolved by assuming that the plasma is isothermal (see details in Polito et al. 2016a).

The S iv lines are particularly important because they allow measurements of densities up to about $10^{13}\,\mathrm{cm}^{-3}$, i.e., higher than those measurable with the O iv intercombination lines. Polito et al. (2016a) presented IRIS measurements in kernels of chromospheric evaporation, which showed high densities, reaching the $10^{13}\,\mathrm{cm}^{-3}$ limit, during the impulsive phase of a flare.

#### $N_{\mathrm{e}}$ from other sequences: iron coronal ions, Fe ix–Fe xiii

The coronal iron ions offer a very large number of diagnostics. Iron lines dominate the XUV spectra, with a large number of strong lines. Table 15 presents a list of EUV line ratios useful to measure densities. Only the main ratios are listed, involving pairs of strong lines close in wavelength. There is a very large number of possible other combinations. There is an extensive literature on the possible diagnostics of iron ions. A fairly complete early review of the iron EUV lines is given in Dere et al. (1987).

The density ranges where the ratios are sensitive are also shown in Table 15. However, this does not mean that all the ratios listed in the table are usable in all cases. Ions like Fe xiii that emit above a million degrees, for example, are simply not seen in coronal holes or in very quiet regions of the Sun. Many lines are blended to some degree, and blending changes depending on the source region.

**Fig. 73:**
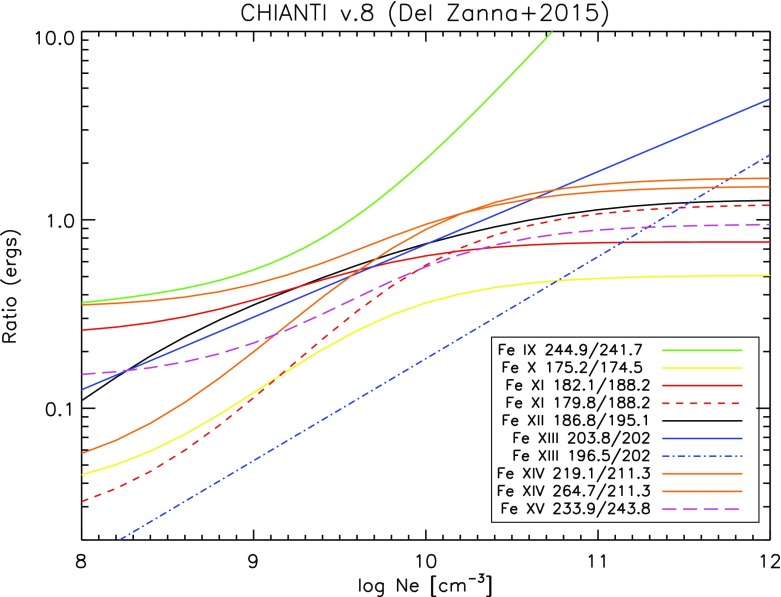
The main diagnostic ratios in the EUV for the iron coronal ions. Many of them are available to the Hinode EIS. The atomic data are from CHIANTI v.8 Reproduced with permission from Del Zanna et al. (2015b)

We emphasize Fe xii and Fe xiii below because the density diagnostics involve strong lines, many of which are routinely observed by Hinode EIS. Some of the main diagnostic ratios (in the EUV), many of which are available to the Hinode EIS, are shown in Fig. 73.

**Table 15:** Some of the common density diagnostics of coronal iron ions

Ion	Transition	$\lambda$ (Å)	R	Observed	Log $T_{\mathrm{e}}$	$\hbox {Log N}_{\mathrm{e}}$
Fe ix	$3{s}^{2}\,3{p}^{6}\,{}^1\hbox {S}_{0}{-}3{s}^{2}\,3{p}^{5}\,3{d}\,{}^3\hbox {P}_{2}$	241.739	a	Skylab		
	$3{s}^{2}\,3{p}^{6}\,{}^1\hbox {S}_{0}{-}3{s}^{2}\,3{p}^{5}\,3{d}\,{}^3\hbox {P}_{1}$	244.909	b	Skylab	6.0	9–12
Fe x	$3{s}^{2}\,3{p}^5\,{}^2\hbox {P}_{1/2}{-}3{s}^{2}\,3{p}^{4}\,3{d}\,{}^2\hbox {D}_{3/2}$	175.27	a	CDS, EIS		9.0–11.0
	$3{s}^{2}\,3{p}^5\,{}^2\hbox {P}_{3/2}{-}3{s}^{2}\,3{p}^4\,3{d}\,{}^2\hbox {D}_{5/2}$	174.534	b	CDS, EIS	6.05	
Fe xi	$3{s}^{2}\,3{p}^4\,{}^1\hbox {D}_{2}{-}3{s}^{2}\,3{p}^3\,3{d}\,{}^1\hbox {F}_{3}$	179.764	a	CDS, EIS		9.0–11.0
	$3{s}^{2}\,3{p}^4\,{}^3\hbox {P}_{1}{-}3{s}^{2}\,3{p}^3\,3{d}\,{}^3\hbox {D}_{2}$	182.17	a	CDS, EIS		9.0–11.0
	$3{s}^{2}\,3{p}^4\,{}^3\hbox {P}_{0}{-}3{s}^{2}\,3{p}^{3}\,3{d}\,{}^3\hbox {D}_{1}$	181.13	a	CDS, EIS		9.0–11.0
	$3{s}^{2}\,3{p}^4\,{}^1\hbox {D}_{2}{-}3{s}^{2}\,3{p}^3\,3{d}\,{}^1\hbox {D}_{2}$	184.79	a	CDS, EIS		9.0–11.0
	$3{s}^{2}\,3{p}^4\,{}^3\hbox {P}_{2}{-}3{s}^{2}\,3{p}^3\,3{d}\,{}^3\hbox {P}_{2}$	188.216	b	CDS, EIS	6.1	
Fe xii	$3{s}^{2}\,3{p}^3\,{}^2\hbox {D}_{5/2}{-}3{s}^{2}\,3{p}^2\,3{d}\,{}^2\hbox {F}_{7/2,5/2}$	186.8 (sbl)	a	CDS, EIS		8.5–12
	$3{s}^{2}\,3{p}^3\,{}^2\hbox {D}_{5/2}{-}3{s}^{2}\,3{p}^2\,3{d}\,{}^2\hbox {D}_{5/2}$	196.65 (bl)	a	CDS, EIS		8.5–12
	$3{s}^{2}\,3{p}^3\,{}^4\hbox {S}_{3/2}{-}3{s}^{2}\,3{p}^2\,3{d}\,{}^4\hbox {P}_{5/2}$	195.12 (sbl)	b	CDS, EIS	6.16	
	$3{s}^{2}\,3{p}^3\,{}^2\hbox {D}_{5/2}{-}3{s}\,3{p}^4\,{}^2\hbox {D}_{5/2}$	338.26	a	CDS		7.0–12.0
	$3{s}^{2}\,3{p}^3\,{}^4\hbox {S}_{3/2}{-}3{s}\,3{p}^4\,{}^4\hbox {P}_{5/2}$	364.47	b	CDS	6.16	
Fe xiii	$3{s}^{2}\,3{p}^2\,{}^1\hbox {D}_{2}{-}3{s}^2\,3p\,3d\,{}^1F_{3}$	196.52	a	CDS, EIS		9.0–11.0
	$3{s}^{2}\,3{p}^2\,{}^3\hbox {P}_{1}{-}3{s}^{2}\,3{p}\,3{d}\,{}^3\hbox {D}_{2}$	200.02	a	CDS, EIS		9.0–11.0
	$3{s}^{2}\,3{p}^2\,{}^3\hbox {P}_{2}{-}3{s}^{2}\,3{p}\,3{d}\,{}^3\hbox {D}_{3,2}$	203.8 (sbl)	a	CDS, EIS		9.0–11.0
	$3{s}^{2}\,3{p}^2\,{}^3\hbox {P}_{0}{-}3{s}^{2}\,3{p}\,3{d}\ {}^3\hbox {P}_{1}$	202.04	b	CDS, EIS	6.2	
	$3{s}^{2}\,3{p}^2\,{}^3\hbox {P}_{1}{-}3{s}\,3{p}^{3}\,{}^3\hbox {D}_{2}$	359.64 (sbl)	a			8.5–10.5
	$3{s}^{2}\,3{p}^2\,{}^3\hbox {P}_{0}{-}3{s}\,3{p}^{3}\,{}^3\hbox {D}_{1}$	348.18	b	CDS	6.2	

(bl) indicates a blend, while (sbl) indicates a self-blend of more than one transition from the same ion. The column R indicates which combination of lines is a good density diagnostic: a/b or b/a

**Fe** **xiii**

Fe xiii (1.5 MK) also has many excellent density diagnostics in the EUV, as recently reviewed, e.g., by Young et al. (2009) and Del Zanna (2011a, 2012b). At the Hinode EIS wavelengths, any ratio involving the main decay to the ground state (at 202.044 Å) and any permitted line decaying to an excited state is density-dependent. Depending on which excited state is involved, the ratio is sensitive to a different density range.

One of the strongest lines is the 203.8 Å, however this line is actually a self-blend of two strong Fe xiii lines, plus other weaker ones tentatively identified by Del Zanna (2011a), and a relatively strong Fe xii line. Other good options are the 196.52 or the 200.02 Å lines. We recall that Fig. 38 shows the main populating processes for these transitions.

**Fig. 74:**
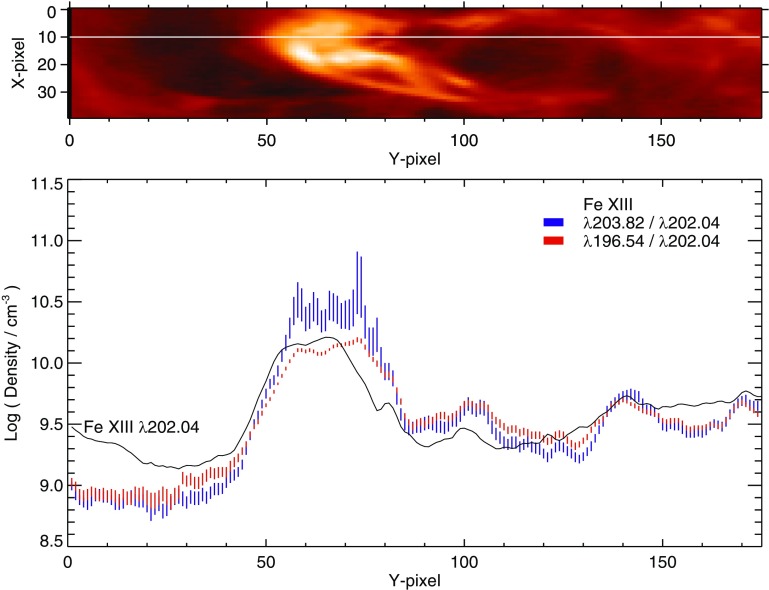
The upper panel shows an image of the intensity of the Fe xiii 202.02 Å line across some active region loops. In the lower panel the red and blue lines show densities derived from two Fe xiii ratios. The black line shows the variation in intensity of the Fe xiii 202.02 Å line (the plotted quantity is log ($10^5 \times \hbox {I}$)) Image reproduced with permission from Young et al. (2009), copyright by the authors

An example of the high accuracy achieved on both the observational (Hinode EIS) and atomic physics (CHIANTI) sides is given in Fig. 74. Excellent agreement in the densities obtained from two different line ratios from Fe xiii is obtained with a cut across some active region loops.

At longer wavelengths, observed by e.g., SOHO/CDS (see, e.g., Mason et al. 1997) and the SERTS rocket flights (see, e.g., Young et al. 1998; Landi 2002), other good density diagnostics are the ratios involving the decay to the ground state at 348.18 Å and any decay to an excited level (e.g., the 359.64 Å and possibly the 311.55, 312.87, 320.8 Å lines, although the last three can be blended).

The accuracy of the atomic data in CHIANTI v.8 to measure densities in EBIT spectra of Fe xiii has recently been confirmed by Weller et al. (2018).

**Fe** **xii**

**Fig. 75:**
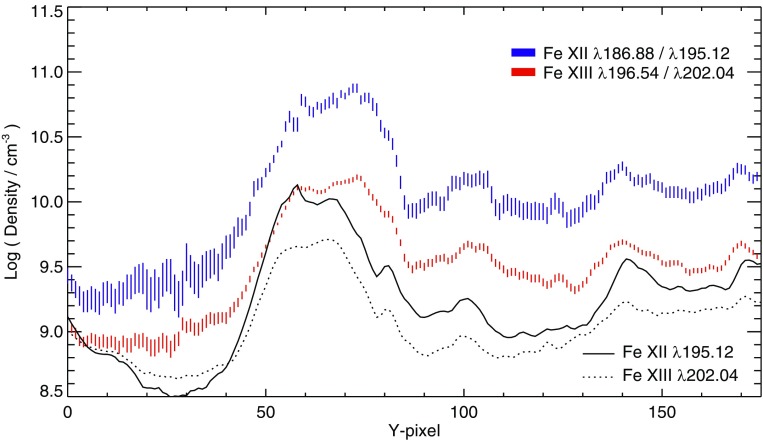
Same as Fig. 74, but with the densities obtained from Fe xiii and Fe xii lines

Fe xii (1.5 MK) also has many excellent density diagnostics in the EUV and UV (see, e.g., Del Zanna and Mason 2005a; Del Zanna 2012b), although the complexity of this ion is such that only recent large-scale scattering calculations (Del Zanna et al. 2012b) seem to have resolved previous long-standing discrepancies. As discussed by e.g., Young et al. (2009), some line ratios were providing too high densities, by about a factor of three, compared to those obtained from Fe xiii lines, as shown in Fig. 75 (using CHIANTI v. 5.2).

**Fig. 76:**
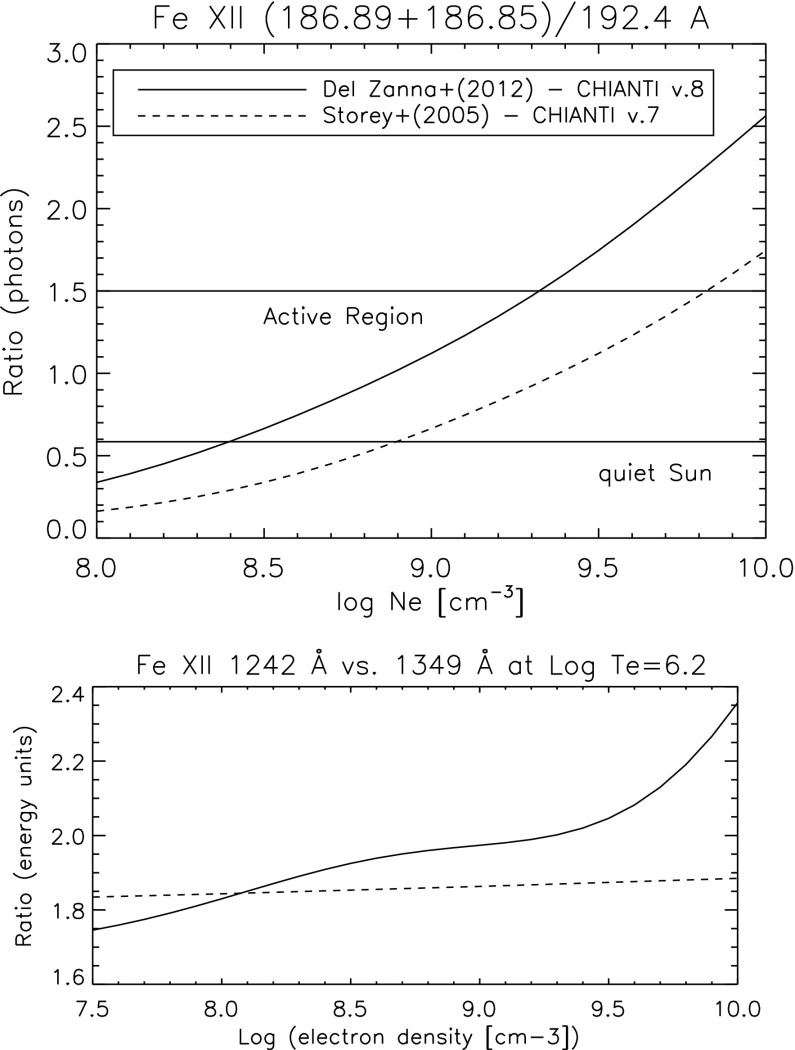
Top: a comparison of different calculations for Fe xii—one of the main density diagnostic ratio in the EUV. Bottom: the ratio of the UV forbidden lines is density-sensitive with the latest atomic data

A large-scale calculation for Fe xii (Del Zanna et al. 2012b) resulted in significantly lower densities obtained from this ion (about 0.5 dex), apparently resolving the discrepancy with the Fe xiii results. This occurred because the population of the lower levels is mainly driven by cascading, so even small changes in the excitation rates to higher levels have a large cumulative effect. Del Zanna et al. (2012b) found increased populations of the ground configuration of about 30%. As a result, intensities of the forbidden lines are also affected, as shown in Fig. 76.

It should be noted that even larger discrepancies between observed and predicted intensities were present until the scattering calculations of Storey et al. (2005). Del Zanna and Mason (2005a) used these data to identify several new lines and point out the best diagnostics for this ion, in particular the ratio of the two self-blends at 186.88–195.12 Å, identified as such by Binello et al. (2001), and later confirmed using laboratory plates (where the lines were actually resolved) by Del Zanna and Mason (2005a). The 186.88 and 195.12 Å lines are routinely observed by Hinode EIS, as discussed, e.g., in Young et al. (2009).

At high densities, the 196.65 Å is to be preferred to the 186.88 Å line, although it appears to be slightly blended in on-disk observations. Several other ratios are useful, although many lines are blended. At longer wavelengths, the ratio of the 338.3 Å with e.g., the 364.5 Å line is also an excellent diagnostic and was observed by the SOHO CDS and SERTS, as discussed e.g., in Young et al. (1998) and Del Zanna (1999). In the UV, a few forbidden lines can also be used to obtain densities. Also, the ratio of the 1242 and 1349 Å lines is seen to be density-sensitive with the latest atomic data.

**Fe** **xi**

This ion has been the most difficult of all coronal ions to provide reliable atomic data for, with a history of several discrepancies and incorrect line identifications for some of the strongest EUV lines. Now, with reliable atomic data and identifications, it is possible to use several line ratios from this ion, as discussed in Del Zanna (2010, 2012b). The best ratios, with the strongest lines, fall at the lower Hinode EIS wavelengths. They are obtained from one of the decays to the ground state at 180.4 (blended with Fe x), 188.2,188.3 Å and from one of several decays to an excited level (179.76, 184.79, 181.13, 182.17 Å). Most of the other lines are blended or weaker.

**Fe** **x**

A review of the density diagnostics for this ion was provided by Del Zanna et al. (2004a). Within the EUV, only the ratio of the weak 175.27 Å line with any of the strong decays to the ground state (e.g., 174.53 Å) is density-sensitive. Several forbidden lines, not listed here, are also useful density diagnostics in other wavelength regions. The latest scattering calculations (Del Zanna et al. 2012a) for this ion changed the population of the lowest levels significantly, removing previous discrepancies between observed and predicted line intensities for the forbidden lines (Del Zanna et al. 2014b).

**Fe** **ix**

One of the best EUV density diagnostic for the 1 MK corona is the Fe ix 241.739/244.909 Å ratio, as discussed in Storey et al. (2002). Feldman et al. (1978c) used Skylab observations and older atomic data to point out that in principle the Fe ix 241/244 Å should be an excellent diagnostic. However, they obtained values that were largely at odds with those obtained from other ions. This led Feldman (1992b) to suggest that non-equilibrium effects were at play. However, it was shown by Storey et al. (2002) that the main problem was in the earlier scattering calculations, as has often been the case for many of these complex coronal ions. A weak density sensitivity is also present in other EUV lines observed by Hinode EIS, as discussed by Young (2009).

## Measurements of electron densities

In what follows, we provide several examples of measurements of electron densities for different solar regions, to show which diagnostics have been applied. Measurements of densities in the off-limb (outer) corona are fundamental for our understanding of coronal heating processes and as a boundary for solar wind modelling. Measurements of densities during flares are also extremely important as they provide information on the timescales of ionisation/recombination processes.

### Densities in coronal holes

Following the first XUV observations, coronal holes were defined as those areas with much lower (by factors up to 10) intensities in coronal lines. They should not be confused, however, with *filament channels*, where the decreased in the intensity is mostly due to absorption by neutral hydrogen and helium. Also, they should not be confused with *dark halos* around active regions, which have been described by Andretta and Del Zanna (2014). Studies based on Skylab and HRTS data have shown that transition region lines have almost the same intensity distribution inside and outside coronal holes, and also show the same pattern of supergranular network.

Various features occur in coronal holes: bright points, macrospicules, jets, etc. However, during solar minimum, (especially the 1995–1996 one) the main feature was that of coronal hole plumes. They appear as ray-like, extended (up to few $R_\odot$) structures in white-light eclipse coronagraph images of the polar regions. Their bases are visible in 1 MK and lower temperature lines in the EUV. It is important to study plumes because they are the only stable observable structures which trace the open field lines within coronal holes, thought to be the source regions of the fast solar wind.

At large distances from the limb, electron densities can be measured from the white-light images, following the van de Hulst (1950) method (see, e.g., Guhathakurta et al. 1993).

Direct measurements from ratios of coronal lines were sparse until SOHO. There were some measurements at lower temperatures, but these were somewhat unreliable, mostly because of the low spectral resolution or low signal of early instruments (see, e.g., Munro et al. 1971; Doschek et al. 1978; Vernazza and Mason 1978). Off-limb densities from S x) were obtained from Skylab (Feldman et al. 1978b) but did not have any spatial resolution along the slit.

#### SOHO

There is quite an extended literature on the topic of coronal hole densities from SOHO, so we just mention here some of the earlier studies, and point out that Wilhelm et al. (2011) has recently provided a review of coronal hole plumes and lane measurements obtained from SOHO.

**Fig. 77:**
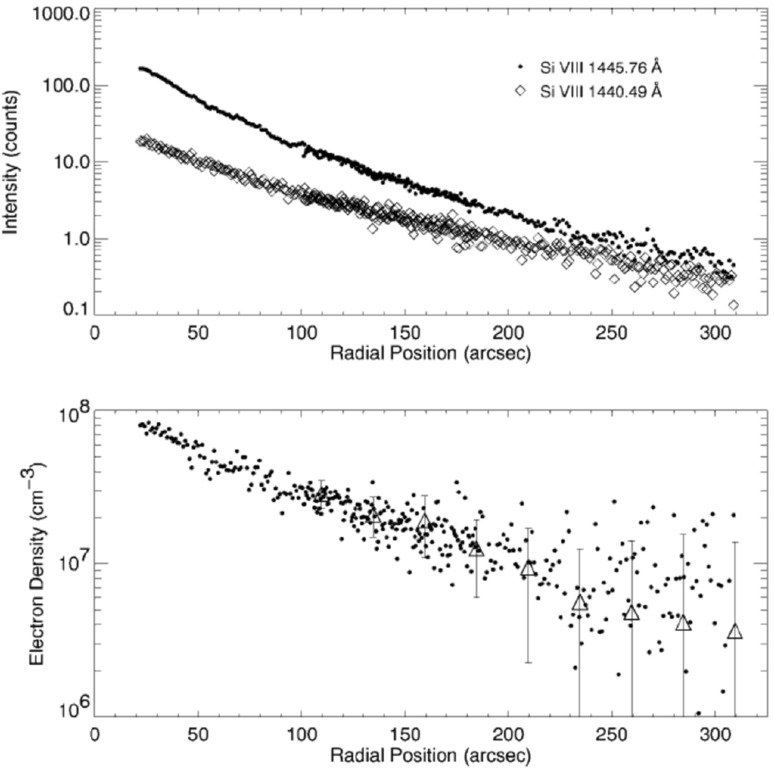
Top: intensities in two Si viii) lines observed off the limb with SoHO SUMER. Bottom: electron densities obtained from the ratio of the two lines Image reproduced with permission from Doschek et al. (1997b), copyright by AAS

Doschek et al. (1997b) applied a ratio technique to coronal lines (Si viii and S x) in SUMER off-limb observations (see Fig. 77). These are important observations as they are the first to provide spatially-resolved densities off-limb at high resolution. Doschek et al. (1997b) found the coronal hole densities to be systematically lower than those in the quiet Sun, by a factor of about 2. At the base of the corona, densities are about $10^{8}\,\hbox {cm}^{-3}$. Similar results were obtained by others also using SUMER, see e.g., Laming et al. (1997), Banerjee et al. (1998) and Warren (1999). Densities from plumes and lanes were obtained from Wilhelm et al. (1998), Wilhelm (2006) among others. Densities decreased from $10^{8}$ to $10^{7}\,\hbox {cm}^{-3}$ around $300{^{\prime \prime }}$ above the limb. Densities in lanes were normally found to be lower than those in plumes. Doschek et al. (1998a) used the O v 759.441 and 761.128 Å lines observed at the limb by SUMER to derive densities inside coronal holes of $N_{\mathrm{e}} = 2{-}3 \times 10^{9}\,\hbox {cm}^{-3}$, a factor of two lower than in the quiet Sun.

SoHO CDS also provided on-disk and off-limb measurements of densities for coronal holes. CDS was able to resolve the weak O iv 625 Å from the nearby strong Mg x line, and many density measurements have been obtained using this line, as well as other lines formed at higher temperatures. Several coronal ions were observed by CDS which allowed on-disk and off-limb measurements of densities. A complete dataset of coronal hole observations was presented in Del Zanna (1999). The Whole Sun Month (WSM) international campaign was set up to obtain coordinated observations from all the SOHO instruments during August 1996. Two workshops followed. As part of the Whole Sun Month measurements of densities from the Si ix 349/341 Å ratio in the polar regions were obtained with CDS. Results are discussed in Del Zanna (1999) and Fludra et al. (1999). Further polar observations in 1997 and 1998 were analysed (Del Zanna 1999), and similar values to those of August 1996 were found (see also Fludra et al. 2002). Such observations were important as they showed that solar cycle variations did not significantly affect the density of the 1 MK coronal plasma. The CDS coronal hole densities were found to be about a factor of two lower than in the quiet Sun regions, as in the case of the SUMER measurements. Similar results have been obtained by others, see e.g., Gallagher et al. (1999).

During the WSM, a large equatorial coronal hole crossed the meridian. It was named the Elephant’s Trunk (Del Zanna and Bromage 1999b). Supergranular network cell centres and boundaries were selected, and averaged densities were obtained from Si ix and O iv line ratios. The O iv ratio showed a tendency to have slightly higher densities in the cell centres ($N_{\mathrm{e}} \simeq 0.6{-}1.1 \times 10^{10}\,\hbox {cm}^{-3}$) than in the boundaries ($N_{\mathrm{e}} \simeq 0.5{-}0.7 \times 10^{10}\,\hbox {cm}^{-3}$). The Si ix) ratio produced $N_{\mathrm{e}} \simeq 1{-}2 \times 10^{8}\,\hbox {cm}^{-3}$. The fact that coronal holes are virtually indistinguishable from QS areas in TR lines suggests that it is the spectroscopic filling factor that changes, assuming the density measurements are correct.

A plume was identified in the Elephant’s Trunk (Del Zanna and Bromage 1999b). The Si ix ratio produced $N_{\mathrm{e}} \simeq 2 \times 10^{8}\,\hbox {cm}^{-3}$ at its base, i.e., a density comparable to those of the QS. Further CDS observations of polar coronal holes showed similar values (Del Zanna 1999; Del Zanna et al. 2003). Higher densities were found in a bright plume observed with CDS (Young et al. 1999). Antonucci et al. (2004) obtained densities and outflowing velocities in a polar coronal hole using the O vi 1032, 1037 Å doublet observed by SOHO UVCS.

### Densities in the quiet Sun

Direct measurements from line ratios in the quiet Sun were also sparse until SOHO, and large discrepancies between results obtained from different lines or instruments were common. Dupree et al. (1976) analysed Skylab HCO data and found significant variations in the C iii 1176/977 Å ratio, for different solar regions, with an average value for the quiet Sun of 0.29, yielding a density $N_{\mathrm{e}} = 4.6 \times 10^9\,\hbox {cm}^{-3}$. A trend for higher densities in the cell centres was found, although in the quiet Sun regions the uncertainty was large. Vernazza and Reeves (1978) derived densities from the same C iii 1176/977 Å lines observed by the Skylab HCO instrument, but found $N_{\mathrm{e}} \simeq 10^{10}\,\hbox {cm}^{-3}$ from the averaged spectra. Moreover, differences were found between the cell centres and network regions, but this time with the network having larger (by about a factor of two) densities than the cell centres, in contradiction to the previous results by Dupree et al. (1976). As we mentioned previously, this ratio is not ideal, because it is also temperature-dependent and the strong resonance line at 977 Å can have optical depth effects.

Various authors used the Skylab NRL S082-B to measure densities. However, only observations close to the limb could be used. Doschek et al. (1978) and Doschek (1997) presented densities for coronal holes and the quiet Sun close to the limb, using C iii, O iii, Si iii, and Si iv lines, finding an averaged value $N_{\mathrm{e}} \simeq 1 \times 10^{10}\,\hbox {cm}^{-3}$. Similar results were obtained by Cook and Nicolas (1979) using C iii, Si iii and Si iv lines.

The atomic data and Skylab, HRTS, and SOHO SUMER observations of the Si iii lines have recently been reviewed in Del Zanna et al. (2015c). Reliable measurements involve only the 1301 Å and nearby lines. Typical quiet Sun on disk or at the limb values are 1–$3\times 10^{10}\,\hbox {cm}^{-3}$.

Doschek (1997) obtained densities of about $1{-}2 \times 10^{9}\,\hbox {cm}^{-3}$ from the N iii 1749/1753 ratio using QS and CH Skylab S083B spectra 4$''$ above the limb. They adopted the atomic data used by Brage et al. (1995). For on-disk spectra, the lines are blended with chromospheric lines and are not usable.

There are several observations of the O iv lines around 1400 Å, however as we mentioned the variations of the ratios are small, and accurate observations and atomic data are required for reliable measurements. Pre-SOHO measurements were reviewed by Brage et al. (1996). A significant scatter in the results was present, however most QS observations yielded values around $10^{10}\,\hbox {cm}^{-3}$.

Keenan et al. (1995a) obtained densities of log $N_{\mathrm{e}}\,(\hbox {cm}^{-3})=10.5$ from Skylab S082B observations of the O v 1371.29/1218.35 Å near the QS limb, using new calculations for O v. However, as Doschek (1997) pointed out, this ratio has little density sensitivity for CH and QS densities, and is actually rather temperature-sensitive. The uncertain Skylab calibration makes the results very uncertain. Keenan et al. (1995a) also presented HRTS observations near disk centre, indicating similar values. Keenan et al. (1995a) found no differences between coronal holes and the quiet Sun.

Keenan et al. (1994c) presented densities obtained from the N iv 1718.6 and 1486.5 Å line ratio measured near the limb from the NRL S082B spectrograph on board Skylab. They obtained large variations across the limb, suggesting that the 1718.6 Å line is blended with a chromospheric line.

Vernazza and Mason (1978) obtained a QS density of $2 \times 10^{8}\,\hbox {cm}^{-3}$ from the Si x 347/356 Å ratio. Feldman et al. (1978b) measured rather high densities from the N-like S x forbidden lines at 1196.3 and 1213 Å above the solar limb with the NRL Skylab spectrograph, about $10^{9}\,\hbox {cm}^{-3}$. The problem with these lines is that they are intrinsically weak, and blended with cool lines in on-disk spectra.

#### SOHO

SUMER provided many observations of the O iv multiplet at 1400 Å. The lines are intrinsically weak and vary little with density, hence results are somewhat unreliable. Moreover, the 1407.4 Å is blended in SUMER spectra with the strong O iii multiplet at 703.8 Å in second order, and the 1408.8 Å line is blended with S iv. Most of the results have been obtained from the 1399.8/1401.2 Å ratio.

Griffiths et al. (1999) and Landi et al. (2000) obtained QS O iv densities of about $10^{10}\,\hbox {cm}^{-3}$, with a large uncertainty and scatter of values. No significant variation with the supergranular network was found. In contrast, Doschek and Mariska (2001) found some evidence that the average densities increase from 6 to $19 \times 10^{9}\,\hbox {cm}^{-3}$ between quiet and brighter network regions (while the 1399.8/1401.2 Å ratio only changes on average from 0.19 to 0.22).

SUMER was able to resolve the O v multiplet at 760 Å. Doschek et al. (1998a) obtained QS densities near disk centre in the range $5{-}8 \times 10^{9}\,\hbox {cm}^{-3}$. SUMER also observed the weak O v forbidden line at 1213.9 Å. Pinfield et al. (1998) used the ratio with the intercombination line at 1218.35 Å to find a density for the QS of $10^{8.5}\,\hbox {cm}^{-3}$, a value at odds with most other measurements.

At the SUMER wavelengths, many diagnostic lines are blended with low-T lines on-disk, but several off-limb observations have been run, up to about 1.3 solar radii. Results from the Si viii 1445.7/1440.5 Å forbidden ratio have been published by several authors, e.g., Laming et al. (1997), Doschek et al. (1997b) and Feldman et al. (1999). Typical QS densities near the limb are $2 \times 10^{8}\,\hbox {cm}^{-3}$, and decrease by about one order of magnitude at 1.3 $R_\odot$. Doschek et al. (1997b) also considered the ratio of the S x forbidden lines at 1213.0 and 1196.3 Å, but concluded that typical QS densities are too close to the low-density limit for reliable measurements to be obtained. Laming et al. (1997) measured densities near the limb from several other line ratios, generally finding good agreement with the Si viii results.

With CDS it was possible to resolve the weak O iv 625 Å from the nearby strong Mg x line and density measurements have been obtained (Del Zanna and Bromage 1999c). On-disk observations of the O iv ratio showed a tendency towards higher densities in the cell centres ($N_{\mathrm{e}} \simeq 1.2 \times 10^{10}\,\hbox {cm}^{-3}$) than in the boundaries ($N_{\mathrm{e}} \simeq 0.5 \times 10^{10}\,\hbox {cm}^{-3}$). The Si ix ratios produced $N_{\mathrm{e}} \simeq 4 \times 10^{8}\,\hbox {cm}^{-3}$. Landi and Landini (1998) found slightly higher values from the Si ix ratios in two QS areas, in the range $5{-}8 \times 10^{8}\,\hbox {cm}^{-3}$. Warren (2005) obtained somewhat lower values around $3 \times 10^{8}\,\hbox {cm}^{-3}$ from the Si ix and Si x ratios. Young (2005a) also measured QS densities using the Si ix 349.9/345.1 Å, finding a remarkable constant value around $2.6 \times 10^{8}\,\hbox {cm}^{-3}$ during the 1996–1998 period. A similar value was also obtained by Brooks and Warren (2006).

**Fig. 78:**
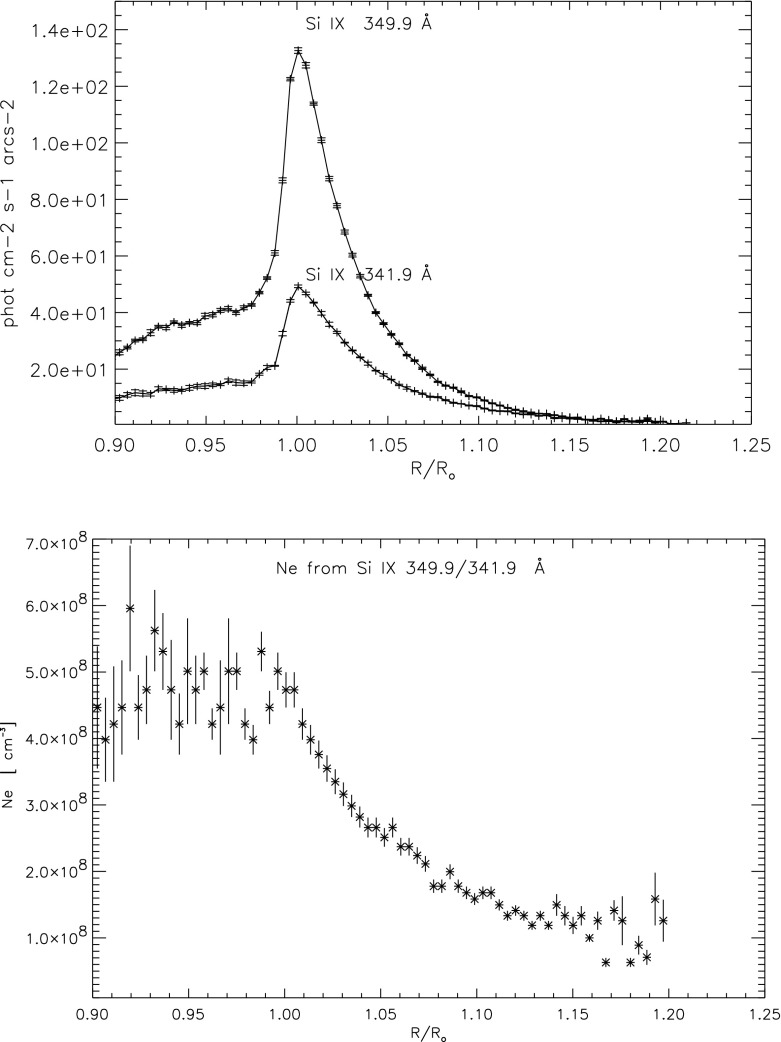
Radiances in a quiet Sun off-limb area and relative electron densities obtained from a SoHO CDS NIS Si ix ratio and CHIANTI v.2 Image reproduced from Del Zanna (1999)

As part of Whole Sun Month campaign CDS obtained measurements of densities from the Si ix 349/341 Å ratio in off-limb observations. Results are described in Del Zanna (1999) and Fludra et al. (1999). The density was found to decrease from $5 \times 10^8$ at the limb, to $1 \times 10^8\,\hbox {cm}^{-3}$ at 1.2 $R_\odot$, see Fig. 78. Gibson et al. (1999) showed that the densities determined from CDS and those determined from white-light observations and the van de Hulst (1950) inversion technique were very similar, an important result. Off-limb observations were regularly carried out by CDS, but no significant differences to the earlier results were found (Del Zanna 1999; Fludra et al. 2002).

#### Densities from Hinode/EIS

Hinode EIS has several density diagnostics for coronal temperatures. Measurements of the off-limb QS corona have produced results in broad agreement with the SOHO CDS ones (see, e.g., Warren and Brooks 2009; Del Zanna 2012b). On-disk measurements are also in good agreement with previous ones (see, e.g., Brooks et al. 2009 using Fe xii).

One advantage of the Hinode EIS measurements is the fact that observations have been carried out continuously since 2006, while e.g., SoHO CDS measurements of the coronal diagnostic lines were significantly affected by degradation (in 1998) of the NIS. This has allowed us to study the variation of the coronal density over a solar cycle. For example, Kamio and Mariska (2012) used Hinode EIS synoptic observations of the quiet Sun and measured densities using the Si x 258.4/261.0 Å line ratio. Values in the range $3{-}5 \times 10^{8}\,\hbox {cm}^{-3}$ were found.

**Fig. 79:**
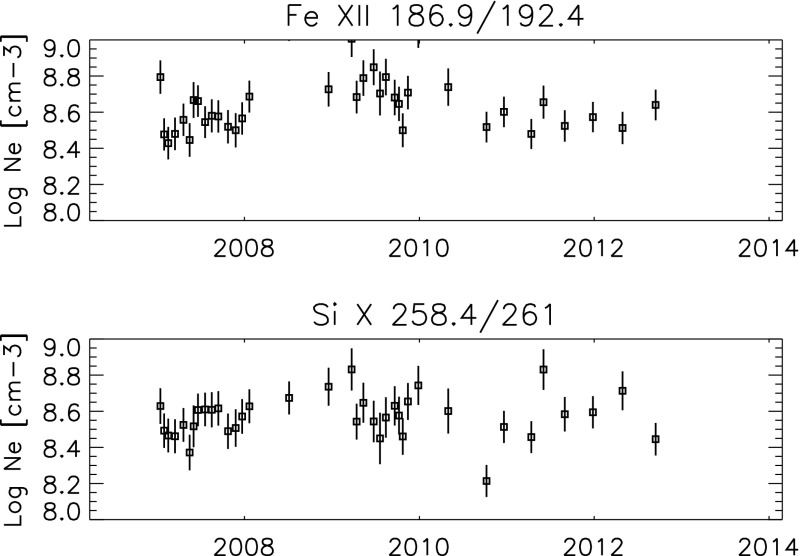
Electron densities in quiet Sun areas obtained from Hinode EIS Si x and Fe xii lines over an 8-year period Adapted from Del Zanna (2013b)

We note, however, that for some line ratios the latest atomic data provide different values, especially for Fe xii. The above studies also used the EIS ground calibration which needed revision. Del Zanna (2013b) presented a new EIS calibration and used Si x and the new atomic data for Fe xii (Del Zanna et al. 2012b) to obtain quiet Sun densities from carefully selected regions between 2006 and 2013. The densities were reasonably constant around a value of $10^{8.5}\,\hbox {cm}^{-3}$ (see Fig. 79), i.e., around $3 \times 10^{8}\,\hbox {cm}^{-3}$, in close agreement with the Si ix results for the 1996–1998 period by Young (2005a).

It is important to keep in mind, however, that the so-called quiet Sun areas as observed in lines formed above 1 MK become increasingly affected by the presence of the active regions during increased solar activity, as shown in Del Zanna and Andretta (2011) and Andretta and Del Zanna (2014).

### Active regions and bright points

There is an extended literature on measurements of densities in active regions, although we would like to point out that the spatial resolution of previous and current spectrometers has not allowed a complete and detailed description of how densities vary along fine structures (e.g., warm loops) that are observed with the highest-resolution imagers. A good review of the diagnostic possibilities in the EUV offered by the Skylab NRL S082A slitless instrument for active regions is given by Dere and Mason (1981). For general reviews on active regions see, e.g., Reale (2014) and Mason and Tripathi (2008).

Kastner et al. (1974b) used OSO-7 off-limb observations of coronal lines with the Goddard spectroheliogram to measure densities and temperatures. They used the Fe xiv lines and the atomic data available at the time to find densities ranging from $1\times 10^{9}$ to $1\times 10^{8}\,\hbox {cm}^{-3}$ above an active region.

Several results have been obtained from the Skylab instruments. For example, Feldman and Doschek (1978) used Skylab observations at the limb with the NRL slit spectrometer (1175–1940 Å range) to obtain densities in an active region from Si iii, C iii, O iv, and O v that ranged from $2\times 10^{10}$ to $3\times 10^{11}\,\hbox {cm}^{-3}$.

**Fig. 80:**
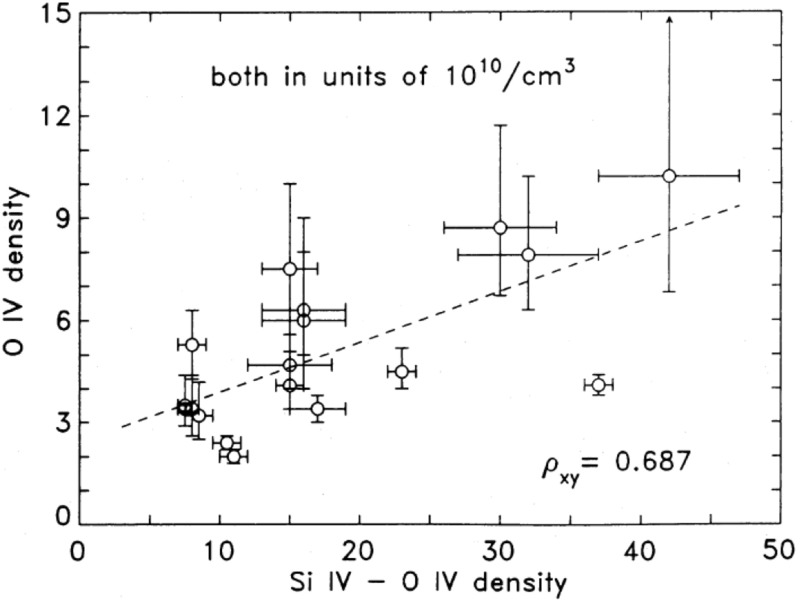
Electron densities obtained from the SMM UVSP observations of the O iv intercombination lines versus those obtained from the ratio of the O iv to the Si iv lines Reproduced with permission from Hayes and Shine (1987)

There are several results from UV lines, measured from e.g., the HRTS and SMM UVSP instruments. For example, Hayes and Shine (1987) used the SMM UVSP observations of the O iv intercombination lines to obtain densities in the brightenings that occur frequently in active regions. Values around $5\times 10^{10}\,\hbox {cm}^{-3}$ were obtained, as shown in Fig. 80. There is in principle another way to estimate densities, that is from the ratio of the O iv intercombination lines with the Si iv allowed lines. It is interesting to note, however, that the densities obtained with this method are found to be quite different than those obtained from the O iv ratios, as shown in Fig. 80. This issue is still hotly debated in the literature regarding the interpretation of similar active region brightenings recently observed by IRIS (Peter et al. 2014; Judge 2015).

HRTS measurements of plage regions from the Si iii lines provided values around $10^{11}{-}10^{12}\,\hbox {cm}^{-3}$ (see, e.g., Nicolas et al. 1979; Del Zanna et al. 2015c).

**Fig. 81:**
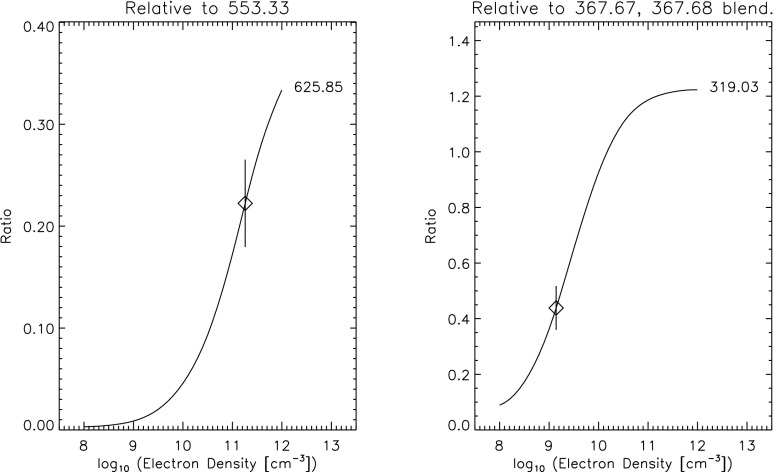
Densities measured by Young and Mason (1997) in TR brightenings occurring in the cores of an active region using SOHO NIS. The left plot is using O iv lines, while the right plot using Mg vii lines Image reproduced with permission from Young and Mason (1997), copyright by Kluwer

The SOHO/SUMER observations of active regions were somewhat limited (to preserve the life of the detectors), so there are not many studies of active regions. SOHO/CDS with its NIS instrument offered several density diagnostics, from the transition region [O iv, Mg vii] to the 1 MK [Si ix, Si x] and higher temperatures [Fe xiii, Fe xiv], however the degraded NIS spectral resolution after the SOHO loss in 1998 limited the results. Young and Mason (1997) measured densities of about $10^{11}\,\hbox {cm}^{-3}$ from the O iv and Mg vii lines in TR brightenings in the cores of an active region using SOHO NIS, as shown in Fig. 81. Density variations were found to be associated to flux emergence.

Averaged densities in the cores of active regions were found to be $2{-}3 \times 10^9\,\hbox {cm}^{-3}$, with higher densities at lower heights (see, e.g., Mason et al. 1999). These results pertain to the 3 MK emission of the hot loops, as is clearly visible off-limb. The legs of the ‘warm’ 1 MK loops could be easily resolvable with NIS, and densities of about $2 \times 10^9\,\hbox {cm}^{-3}$ were obtained at their bases (see, e.g., Del Zanna 2003). Several density measurements have been obtained from EUV lines measured by the various SERTS rocket flights, with results in broad agreement with those from SOHO CDS (Young et al. 1994; Brosius et al. 1996, 2000; Young et al. 1998).

Hinode EIS has offered several excellent density diagnostics for active regions, from Mg vii, Fe xi, Fe xii, Fe xiii, Fe xiv, Si x. Several studies have been carried out, see for example Doschek et al. (2007b), Watanabe et al. (2007, 2009), Young et al. (2009, 2012) and O’Dwyer et al. (2011). The densities in active region cores were found to vary between $10^9$ and $10^{10}\,\hbox {cm}^{-3}$. A summary of the densities obtained from the various ions observed by Hinode EIS is provided in Del Zanna (2012b).

**Fig. 82:**
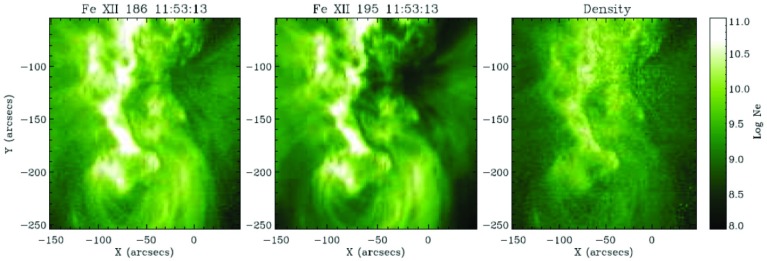
A 2-D map of the average electron density in active region moss as obtained from the ratio of two Fe xii lines observed by Hinode EIS Adapted from Tripathi et al. (2008)

Off-limb observations of ARs with Hinode/EIS indicated similar densities as found from SOHO/CDS (see, e.g., O’Dwyer et al. 2011). On-disk observations of the hot cores of ARs produced averaged densities in agreement with earlier results (see, e.g., Tripathi et al. 2008, 2011; Del Zanna 2013a). An example is shown in Fig. 82. The highest densities are in the ‘moss’ regions, and correspond to the strongest magnetic fields.

**Fig. 83:**
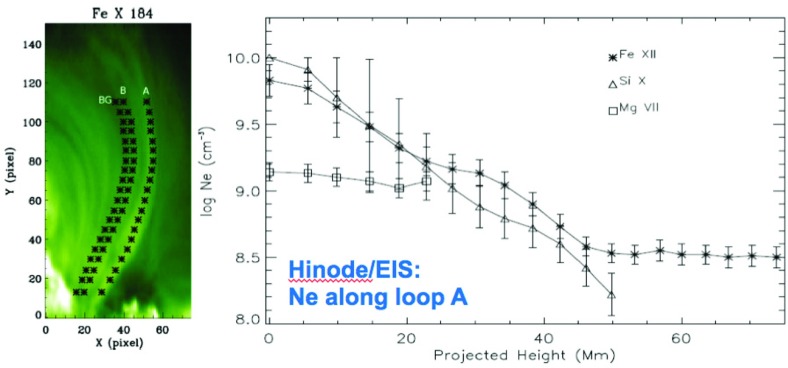
Electron densities along the leg of a warm loop as measured from line ratios observed with Hinode EIS Adapted from Tripathi et al. (2009)

At lower temperatures, Hinode/EIS has a Mg vii diagnostic ratio (Young et al. 2007a, b), although some problems with this ratio have been noted (Del Zanna 2009a). Hinode EIS measurements in the Mg vii lines at the legs of AR warm loops have generally provided densities of about $3 \times 10^9\,\hbox {cm}^{-3}$ and filling factors between 0.2 and 1 (see, e.g., Tripathi et al. 2009; Young et al. 2012), in agreement with the earlier SOHO CDS results.

Hinode EIS measurements of the densities of coronal loops are limited by the effective spatial resolution of the instrument ($3{-}4{^{\prime \prime }}$). Results have been published by various authors, see e.g., Warren et al. (2008b), Tripathi et al. (2009) and Gupta et al. (2015). An example is shown in Fig. 83. Tripathi et al. (2009) used Hinode EIS measurements of densities from Mg vii, Si x and Fe xii to estimate the spectroscopic filling factor of a warm loop. Values around unity were found, with smaller values from Si x and Fe xii (0.02) near the base of the loop. The values based on Fe xii need to be revised in light of the revised atomic data for this ion (Del Zanna et al. 2012b).

Brooks et al. (2012) used a combination of Hinode EIS Fe xiii density diagnostics, Fe xii intensities and SDO AIA images to infer that many of the warm loops that are visible at the EIS spatial scales are probably composed of just a few sub-resolution structures.

#### Active region jets and bright points

**Fig. 84:**
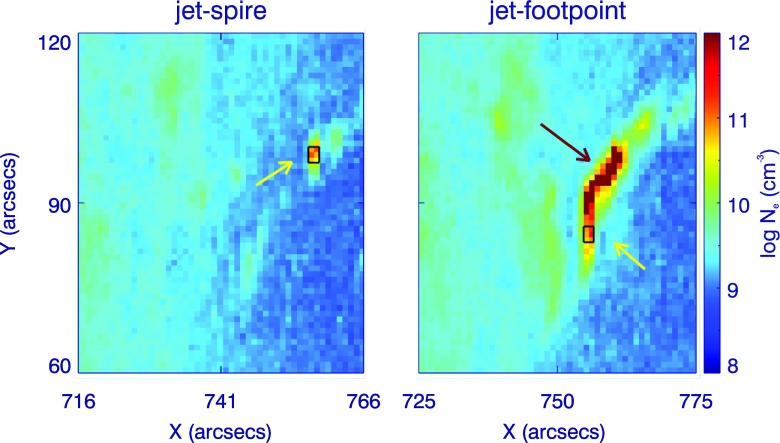
Density maps obtained from the Fe xii lines observed by Hinode EIS for a jet spire and footpoint region Adapted from Mulay et al. (2017)

An interesting measurement of the density of an active region jet was obtained by Chifor et al. (2008) with Hinode EIS. The density-sensitive Fe xii 186.8 Å self-blend showed strong blue-shifted components, so different densities for the various components were estimated. Densities reached values of about $10^{11}\,\hbox {cm}^{-3}$ in the blue-shifted component. Densities from Hinode EIS Fe xii observations of a jet footpoint and spire were also obtained by Mulay et al. (2017). Also in this case, values of about $10^{11}\,\hbox {cm}^{-3}$ or more were obtained as shown in Fig. 84.

Hinode EIS measurements of densities of bright points have also been carried out, see for example Dere (2008) and Alexander et al. (2011). Densities of about $5 \times 10^9\,\hbox {cm}^{-3}$ and small filling factors were found. We note, however, that the densities obtained from Fe xii should be revisited.

### Flares

**Fig. 85:**
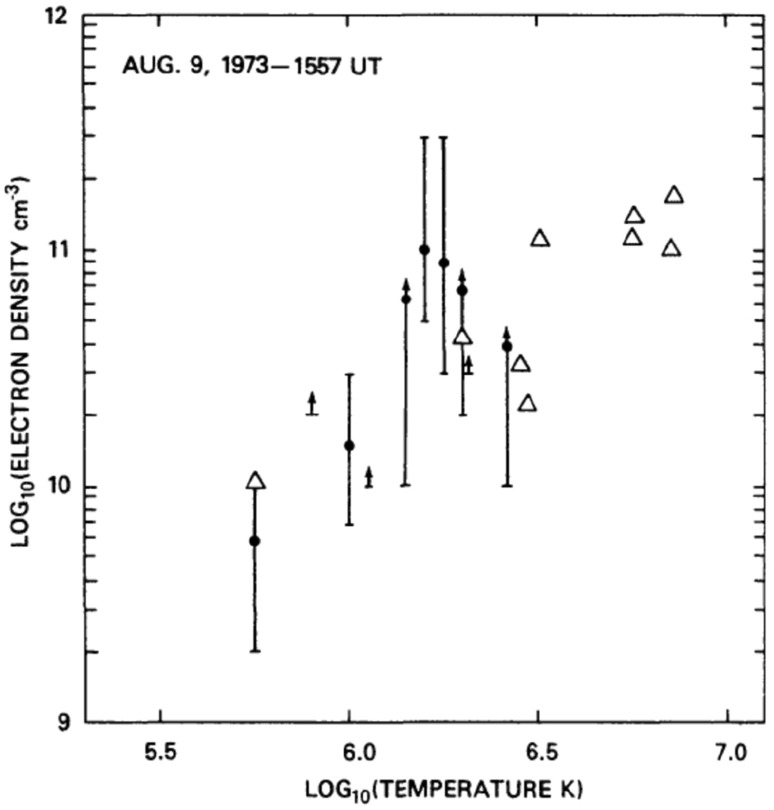
Electron densities obtained from Skylab during a small compact flare. The points were obtained from line ratios, while the triangles from the emission measures and the flare size Image reproduced with permission from Dere et al. (1979), copyright by AAS

A general review of solar flares and diagnostics is given by Doschek (1990). One of the most comprehensive reviews of electron densities measured during solar flares in the EUV is given by Dere et al. (1979), where observations with the Skylab NRL slitless S082A instrument were used. One example is shown in Fig. 85. That instrument was ideal for compact, bright, isolated features.

The observations were excellent in that several line ratios of many ions could be used at once. Such a comprehensive diagnostic has not been available since. Electron densities ranged between 0.1 and $1 \times 10^{11}\,\hbox {cm}^{-3}$, from ions formed in the 1–6 MK range. The densities increase with the temperature of formation of the line. Most of the diagnostics covered the 1–3 MK range. Only one diagnostic at higher temperatures was available, from Ca xvii. It provided a density of about $10^{11}\,\hbox {cm}^{-3}$. A similar result ($5 \times 10^{11}\,\hbox {cm}^{-3}$) was obtained by Doschek et al. (1977b) also from Ca xvii for the 1973 August 9 flare.

A good review of the diagnostic potential in the UV offered by the Skylab NRL S082B normal incidence slit spectrometer is provided by Widing and Cook (1987), who analysed the 1973 December 17 flare.

In what follows we give a short overview of the main results, organised by the formation temperature of the lines.

#### Flare lines (about 10 MK)

During flares, a significant amount of plasma is heated to 10 MK or more. It is therefore very important to measure the density of this high-temperature plasma. However, results so far have been limited. There are several diagnostics available in the X-rays, but they are mainly useful for densities much higher than those typical of most solar flares, $10^{11}\,\hbox {cm}^{-3}$ (see, e.g., McKenzie et al. 1985). For example, the Si xiii and S xv lines become sensitive to densities well above $10^{13}\,\hbox {cm}^{-3}$, so are not useful for solar flares.

A few diagnostics in the X-rays (around 10 Å) are available, mostly from Fe xxi and Fe xxii 2*p*–4*d* transitions, which were identified by Fawcett et al. (1987) from SMM FCS observations and discussed by Phillips et al. (1996). The SMM FCS observations suggested that very high densities, about $\log N_{\mathrm{e}}\,(\hbox {cm}^{-3}) = 13$ could be present. However, the lines were very weak, so accurate measurements were not possible. SMM BCS observations of satellite lines indicated lower densities, which was interpreted by Phillips et al. (1996) as due to the much larger spatial area observed by this instrument, compared to FCS.

**Fig. 86:**
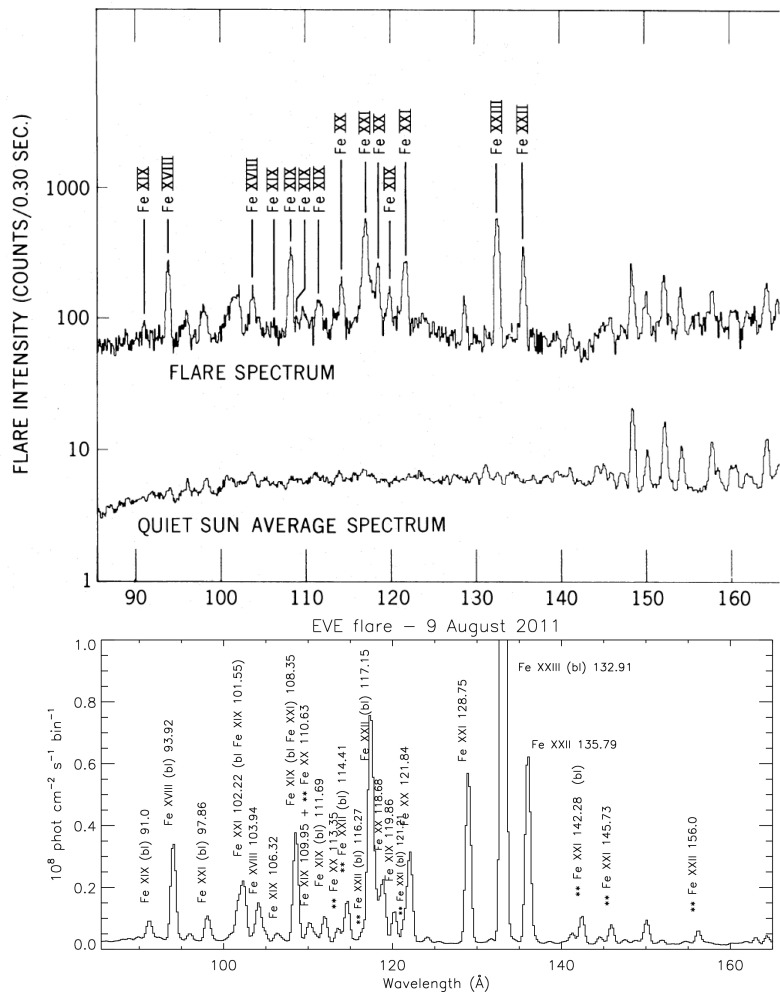
Top: flare ‘E’ spectrum from OSO-5. Note that the quiet Sun spectrum is displayed on a different scale. Image reproduced with permission from Kastner et al. (1974a), copyright by AAS. Bottom: SDO EVE spectrum of the X-class flare on 2011 August 9, with a pre-flare spectrum subtracted (Del Zanna and Woods 2013) and in the same wavelength range as the OSO-5 spectrum. Lines that are density-sensitive (decays to excited levels) are highlighted with double asterisks

The strongest lines emitted by flare iron ions fall in the soft X-rays. Table 16 provides a list of the main lines. OSO-5 obtained the first solar flare spectra of these lines (Kastner et al. 1974a). An example spectrum (flare E) is shown in Fig. 86, together with a more recent flare spectrum from SDO EVE (Del Zanna and Woods 2013).

**Table 16:** List of the main soft X-rays Fe flare lines

Ion	$\lambda$	Transition	$T_{\max }$	Notes
Fe xix	91.013	$2{s}^2\,2{p}^4\,{}^1\hbox {D}_{2}{-}2{s}\,2{p}^5\,{}^1\hbox {P}_{1}$	7.0	
Fe xxi	91.27	$2{s}^2\,2{p}^2\,{}^3\hbox {P}_{0}{-}2{s}\,2{p}^3\,{}^3\hbox {S}_{1}$	7.1	
Fe xx	93.781	$2{s}^2\,2{p}^3\,{}^2\hbox {D}_{5/2}{-}2{s}\,2{p}^4\,{}^2\hbox {P}_{3/2}$	7.0	
Fe xviii	93.923	$2{s}^2\,2{p}^5\,{}^2\hbox {P}_{3/2}{-}2{s}\,2{p}^6\,{}^2\hbox {S}_{1/2}$	6.9	
Fe xxi	97.864	$2{s}^2\,2{p}^2\,{}^3\hbox {P}_{1}{-}2{s}\,2{p}^3\,{}^3\hbox {S}_{1}$	7.1	
Fe xvii	98.25	$2{s}^{2}\,2{p}^{5}\,3{s}\,{}^3\hbox {P}_{1}{-}2{p}^{6}\,3{s}\,{}^1\hbox {S}_{0}$	6.9	
Fe xxii	100.775	$2{s}^2\,2{p}\,^2\hbox {P}_{1/2}{-}2{s}\,2{p}^2\,{}^2\hbox {P}_{3/2}$	7.1	
Fe xix	101.55	$2{s}^2\,2{p}^4\,{}^3\hbox {P}_{2}{-}2{s}\,2{p}^5\,{}^3\hbox {P}_{1}$	7.0	
Fe xxi**	102.217	$2{s}^2\,2{p}^2\,{}^3\hbox {P}_{2}{-}2{s}\,2{p}^3\,{}^3\hbox {S}_{1}$	7.1	
Fe xviii	103.948	$2{s}^2\,2{p}^5\,{}^2\hbox {P}_{1/2}{-}2{s}\,2{p}^6\,{}^2\hbox {S}_{1/2}$	6.9	
Fe xix	106.317	$2{s}^2\,2{p}^4\,{}^3\hbox {P}_{1}{-}2{s}\,2{p}^5\,{}^3\hbox {P}_{0}$	7.0	Weak
Fe xxi	108.118	$2{s}^2\,2{p}^2\,{}^3\hbox {P}_{0}{-}2{s}\,2{p}^3\,{}^3\hbox {P}_{1}$	7.1	Weak
Fe xix	108.355	$2{s}^2\,2{p}^4\,{}^3\hbox {P}_{2}{-}2{s}\,2{p}^5\,{}^3\hbox {P}_{2}$	7.0	Strong
Fe xix	109.952	$2{s}^2\,2{p}^4\,{}^3\hbox {P}_{0}{-}2{s}\,2{p}^5\,{}^3\hbox {P}_{1}$	7.0	Weak
Fe xx	110.627	$2{s}^2\,2{p}^3\,{}^2\hbox {D}_{3/2}{-}2{s}\,2{p}^4\,{}^2\hbox {D}_{3/2}$	7.0	
Fe xix	111.695	$2{s}^2\,2{p}^4\,{}^3\hbox {P}_{1}{-}2{s}\,2{p}^5\,{}^3\hbox {P}_{1}$	7.0	Weak
Fe xx	113.349	$2{s}^2\,2{p}^3\,{}^2\hbox {D}_{5/2}\,2{s}\,2{p}^{4}\,{}^2\hbox {D}_{5/2}$	7.0	
Fe xxii**	114.410	$2{s}^2\,2{p}\,^2\hbox {P}_{3/2}{-}2{s}\,2{p}^2\,{}^2\hbox {P}_{3/2}$	7.1	
Fe xxii	116.268	$2{s}^2\,2{p}\,^2\hbox {P}_{3/2}{-}2{s}\,2{p}^2\,{}^2\hbox {S}_{1/2}$	7.1	
Fe xxii	117.154	$2{s}^2\,2{p}\,^2\hbox {P}_{1/2}{-}2{s}\,2{p}^2\,{}^2\hbox {P}_{1/2}$	7.1	Strong
Fe xxi**	117.50	$2{s}^2\,2{p}^2\,{}^3\hbox {P}_{1}{-}2{s}\,2{p}^3\,{}^3\hbox {P}_{1}$	7.1	
Fe xx	118.680	$2{s}^2\,2{p}^3\,{}^4\hbox {S}_{3/2}{-}2{s}\,2{p}^4\,{}^4\hbox {P}_{1/2}$	7.0	Strong
Fe xix**	119.983	$2{s}^2\,2{p}^4\,{}^3\hbox {P}_{1}{-}2{s}\,2{p}^5\,{}^3\hbox {P}_{2}$	7.0	
Fe xxi**	121.213	$2{s}^2\,2{p}^2\,{}^3\hbox {P}_{2}{-}2{s}\,2{p}^3\,{}^3\hbox {P}_{2}$	7.1	Weak
Fe xx	121.845	$2{s}^2\,2{p}^3\,{}^4\hbox {S}_{3/2}{-}2{s}\,2{p}^4\,{}^4\hbox {P}_{3/2}$	7.0	Strong
Fe xxi**	123.831	$2{s}^2\,2{p}^2\,{}^3\hbox {P}_{2}{-}2{s}\,2{p}^3\,{}^3\hbox {P}_{1}$	7.1	Weak
Fe xxi	128.753	$2{s}^2\,2{p}^2\,{}^3\hbox {P}_{0}{-}2{s}\,2{p}^3\,{}^3\hbox {D}_{1}$	7.1	Strong
Fe xx	132.840	$2{s}^2\,2{p}^3\,{}^4\hbox {S}_{3/2}{-}2{s}\,2{p}^4\,{}^4\hbox {P}_{5/2}$	7.0	Strong
Fe xxiii	132.906	$2{s}^2\,{}^1\hbox {S}_{0}{-}2{s}\,2{p}\,{}^1\hbox {P}_{1}$	7.2	Strong
Fe xxii	135.791	$2{s}^2\,2{p}\,^2\hbox {P}_{1/2}{-}2{s}\,2{p}^2\,{}^2\hbox {D}_{3/2}$	7.1	Strong
Fe xxi**	142.144	$2{s}^2\,2{p}^2\,{}^3\hbox {P}_{1}{-}2{s}\,2{p}^3\,{}^3\hbox {D}_{2}$	7.1	Weak
Fe xxi**	142.281	$2{s}^2\,2{p}^2\,{}^3\hbox {P}_{1}{-}2{s}\,2{p}^3\,{}^3\hbox {D}_{1}$	7.1	Weak
Fe xxi**	145.732	$2{s}^2\,2{p}^2\,{}^3\hbox {P}_{2}{-}2{s}\,2{p}^3\,{}^3\hbox {D}_{3}$	7.1	Weak
Fe xxii**	156.019	$2{s}^2\,2{p}\,^2\hbox {P}_{3/2}{-}2{s}\,2{p}^2\,{}^2\hbox {D}_{5/2}$	7.1	bl

The lines useful as density-diagnostics are highlighted with **. $\lambda$ (Å) is the experimental wavelength, $T_{\max }$ (in logarithm) the approximate temperature of formation of the ion in equilibrium; bl means the line is blended, although we note that all the lines are blended to some degree with lower-temperature transitions

**Fig. 87:**
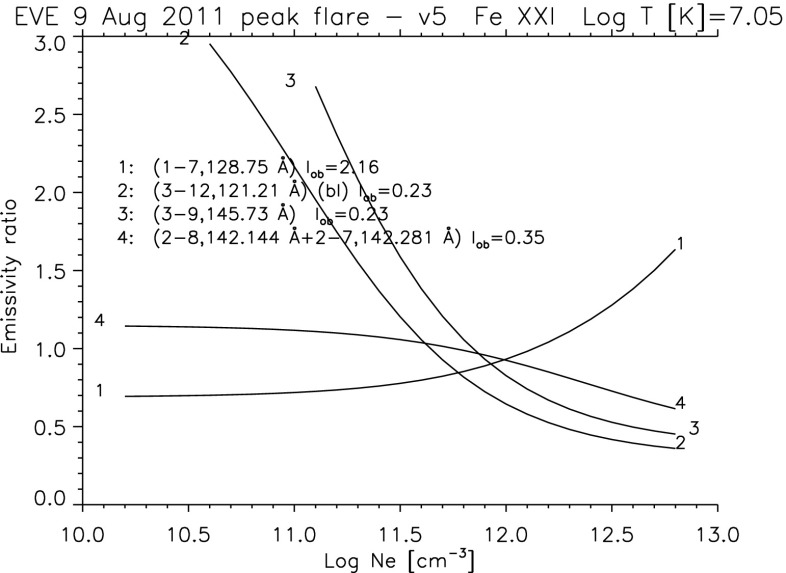
Emissivity ratio plots for Fe xxi lines observed by SDO EVE during the X-class flare on 2011 August 9 Revised from Del Zanna and Woods (2013)

Several density-sensitive line ratios are available in this wavelength region, from Fe xx, Fe xxi, Fe xxii, as discussed in Mason et al. (1984). An analysis of these spectra is also given in Mason et al. (1984), where a density of $4 \times 10^{11}\,\hbox {cm}^{-3}$ was found from the Fe xxi lines.

A recent review of the diagnostics available from flare iron lines in the soft X-rays, with a discussion of all the blends and recent atomic data is given by Del Zanna and Woods (2013), using SDO/EVE version 3 flare spectra. The low spectral resolution of the EVE instrument is a limiting factor, as many of the density-sensitive lines are blended with stronger lines. Del Zanna and Woods (2013) found densities between $10^{11}$ and $10^{12}\,\hbox {cm}^{-3}$ from one of the flares, in agreement with the previous OSO-5 results. A density of about $10^{11}\,\hbox {cm}^{-3}$ was also found by Warren et al. (2013) using SDO EVE spectra. On the other hand, Milligan et al. (2012) obtained from the EVE spectra slightly higher densities, reaching $10^{12}\,\hbox {cm}^{-3}$. We have re-analysed one of the large X-class flares discussed in Del Zanna and Woods (2013), where the density-sensitive lines are more visible. We used the more recent version 5 EVE data, and also found densities of the order of $10^{12}\,\hbox {cm}^{-3}$, as shown in Fig. 87. In any case, it is important to keep in mind that such values are averaged values over the whole flare, so it is perfectly reasonable to assume that higher densities could be present in localised places.

Densities obtained from the emission measures and estimates of the sizes of the post-flare loops in smaller flares are often lower, of the order of $5 \times 10^{10}\,\hbox {cm}^{-3}$ (cf. Del Zanna et al. 2011b; Polito et al. 2015), if the plasma is assumed to be homogeneously distributed within the flare loops (i.e., a spectroscopic filling factor of unity is assumed). In other words, such measurements from the emission measures are lower estimates of what the densities actually are. The important issue of the actual densities of high-temperature flare plasma remains to be resolved with a next-generation spectrometer that could spatially resolve flares. Measuring densities is important for flare modelling, as cooling times for very high densities ($10^{13}\,\hbox {cm}^{-3}$) and temperatures (12 MK) can reach very short timescales, of the order of seconds.

#### Coronal lines (1–6 MK)

**Fig. 88:**
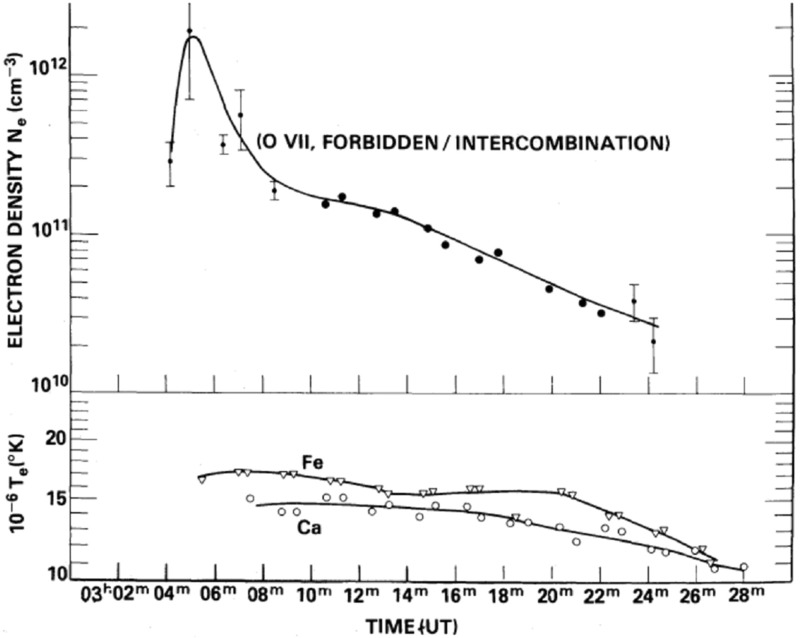
Top: Electron densities obtained from O vii SOLEX observations during a flare. Bottom: temperatures obtained from the same observation Image reproduced with permission from Doschek et al. (1981b), copyright by AAS

As already mentioned, He-like ions provide some diagnostics. O vii, formed around 2 MK, has been used quite extensively in the past. One example comes from the P78-1 SOLEX observations of the O vii lines during a major solar flare, analysed by McKenzie et al. (1980a). The density during the flare raised from about 0.5–$2\times 10^{11}\,\hbox {cm}^{-3}$. A similar study of SOLEX O vii observations of two flares was published by Doschek et al. (1981b). Similar densities were obtained, with a (single) peak value for both flares of about $10^{12}\,\hbox {cm}^{-3}$. Figure 88 shows one example.

Among the other He-like ions, the Ne ix lines would be useful but they are blended in flares with Fe xix lines, and accurate deblending is needed. Mg xi lines are useful only for densities higher than $10^{12}\,\hbox {cm}^{-3}$, and require very high resolution spectra.

Aside from the He-like ions, excellent diagnostics of 1–6 MK plasma are found in the soft X-rays and in the EUV. While there are plenty of measurements in the EUV, mostly from Skylab and Hinode/EIS, only one observation in the soft X-rays is available. This was obtained from a huge (5 m) grazing-incidence spectrograph on board a sounding rocket on 1982 July 13, about 2 min after the peak of a C9-class flare. The instrument and the observations are described in Acton et al. (1985), while the diagnostics are discussed in Brown et al. (1986). These latter authors obtained densities from Fe xiv (59 Å) and Si ix (56 Å) line ratios of about $2{-}3 \times 10^{10}\,\hbox {cm}^{-3}$, and about $5 \times 10^{10}\,\hbox {cm}^{-3}$ from Ca xv lines at 22.7 Å. Densities from the He-like C, N, O lines were similar, about $1{-}3\times 10^{10}\,\hbox {cm}^{-3}$, while those from Ne ix were significantly higher, perhaps because of the known blending issues.

Widing and Cook (1987) discussed Skylab NRL S082A spectroheliograph observations in the EUV of the 1973 December 17 flare, where lines from Ca xv and Ca xvi (3–5 MK) showed densities slightly above log $N_{\mathrm{e}}\,(\hbox {cm}^{-3})=11$, while lines from Fe xiv and Fe xv (2 MK) indicated much smaller values.

Keenan et al. (1993a) used interpolated collision strengths for Ar xiii to measure densities from EUV flare observations with the Skylab NRL S082A instrument. They consistently obtained values around $10^{11}\,\hbox {cm}^{-3}$, in very good agreement with those obtained from Ca xv (Keenan et al. 1988a).

Keenan et al. (1989c) used lines from the Be-like Ar xv and EUV observations of a flare with the Skylab NRL S082A slitless spectrograph to attempt a measurement of the density from the 221.12/266.23 Å ratio. However, the ratio was below the theoretical density limit, which was interpreted as being caused by blending of the 266.23 Å line with an unidentified line.

Lines from the B-like S xii were used by Keenan et al. (2002b) to obtain densities for active regions and flares (log $N_{\mathrm{e}}\,(\hbox {cm}^{-3}) \simeq 9.5$, 10, respectively) from Skylab NRL S082A and SERTS observations. The results are in agreement with those obtained from Fe xiv and Fe xv lines, emitted at similar temperatures (2 MK).

With Hinode EIS, it has been possible to spatially resolve the various flare emission during flares and measure densities from coronal lines. Watanabe et al. (2009) showed that the Fe xiii 203.8/202 Å ratio seemed to reach a high-density limit (of about 4.34) in a kernel of a flare, indicating densities of about $10^{11}\,\hbox {cm}^{-3}$ or higher. Discrepancies with previous atomic data were noted by these authors. However, we note that this observationally-based high-density limit is in very good agreement with the theoretical value of 4.4 obtained from the latest calculations (Del Zanna and Storey 2012).

Del Zanna et al. (2011b) measured densities from Fe xiv line ratios observed by Hinode EIS during a small B-class flare. The Fe xiv line profiles in the ribbons, kernels of chromospheric evaporation during the impulsive phase, were a superposition of two components, a rest and a blue-shifted component. The rest component, which was due to the overlying foreground plasma, had a density of about $6 \times 10^{9}\,\hbox {cm}^{-3}$. The blue-shifted component had densities in some places close to the high-density limit, i.e., indicating densities of about $10^{11}\,\hbox {cm}^{-3}$ or higher. From the emission measures, it was estimated that the depth of the chromospherically-evaporated material was only about 10 km. Young et al. (2013) used the same Fe xiv diagnostics but obtain a lower density of $3 \times 10^{10}\,\hbox {cm}^{-3}$ in the blue-shifted component, in a kernel of a large solar flare.

#### Cooler TR lines (below 1 MK)

Widing and Cook (1987) obtained from the Skylab NRL S082B normal incidence slit spectrometer observations of the 1973 December 17 flare densities from low-temperature lines [O iv, O v, and S iv] and found values in the range $\log N_{\mathrm{e}}\,(\hbox {cm}^{-3})\,=\,11{-}12$ (S v) showed higher values).

Feldman et al. (1977) obtained densities from the intersystem O iv 1401/1407 Å ratio using a flare observation with the Skylab NRL S082B slit spectrometer. The spectral profiles showed a stationary component but a significant non-thermal width. Densities were not very high, a few times $10^{11}\,\hbox {cm}^{-3}$. Conflicting results concerning the O v 1371/1218 Å ratio were found. The authors noted that the 1371 line becomes blended with a line at 1371.37 Å. Keenan et al. (1995a) obtained higher densities, log $N_{\mathrm{e}}\,(\hbox {cm}^{-3}) \simeq 12$, from the same instrument and ratio, during the 1973 August 9 flare.

Dufton et al. (1982) used Skylab NRL S082B AR and flare observations of the Al-like S iv lines, obtaining values in the range log $N_{\mathrm{e}}\,(\hbox {cm}^{-3}) \simeq 11{-}12$.

Keenan et al. (1991) re-analysed Skylab NRL S082A observations of two flares with updated atomic data for the 2–3 transitions in O v. From the lines at 192.80, 192.90, 215.10, 215.25, 220.35, and 248.46 Å, they obtained densities of approximately $10^{12}\,\hbox {cm}^{-3}$, significantly higher than those measured by Widing et al. (1982) using previous atomic data. The 192.80, 192.90 (as self-blend) and 248.46 Å lines are observed by Hinode EIS, as discussed in detail by Young et al. (2007b) and Del Zanna (2009a). However, one problem in flare observations is that the lines become broad and the 192.9 Å normally becomes blended with the strong resonance line from Ca xvii. The 248.46 Å line might also be blended. Other problems are the temperature sensitivity of the ratio, and the degradation of the EIS instrument at the 248.46 Å wavelength.

**Fig. 89:**
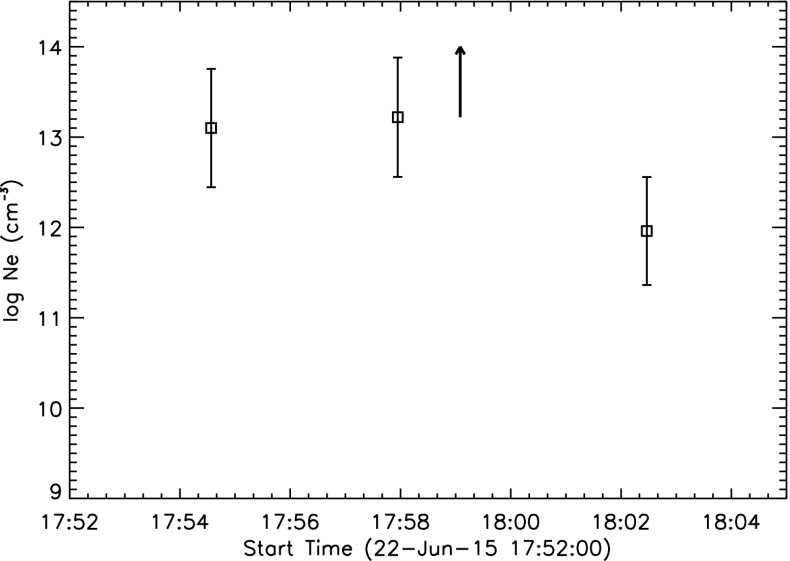
Electron densities obtained from S iv IRIS observations during a flare Adapted from Polito et al. (2016b)

With the launch of IRIS, it has become possible to measure densities using the S iv and O iv lines around 1400 Å during flares. An up-to-date discussion of the atomic data and measurements during the impulsive phase of flare ribbons can be found in Polito et al. (2016a). As already mentioned in the diagnostic section, the O iv are not sensitive to densities higher than $10^{12}\,\hbox {cm}^{-3}$, but S iv lines are. Figure 89 shows the evolution over time of S iv densities, indicating very high values during the initial phase of the flare. Indeed the high-density limit of $10^{13}\,\hbox {cm}^{-3}$ was reached in one instance.

## Diagnostics of electron temperatures

There is ample literature where temperatures have been obtained from spectral lines emitted using different ions. These results are strongly dependent on the knowledge of the relative ion populations, which even in equilibrium are sometimes quite uncertain. There is also ample literature where temperatures have been obtained from broad-band imaging, where additional uncertainties such as off-band contributions and elemental abundance variations are present. In this section, we mainly focus on results based on lines emitted from the same ion, which in principle are more reliable.

### Temperatures from line ratios within the same ion

A direct way to measure the electron temperature is to consider the intensity ratio of two allowed lines that have different excitation energies. If, e.g., the two lines are excited from the ground level *g*:

105 $$\begin{aligned} \frac{I_{g,j}}{I_{g,k}} = \frac{\varDelta E_{g,j} \varUpsilon _{g,j}}{\varDelta E_{g,k} \varUpsilon _{g,k}} \exp \left[ \frac{\varDelta E_{g,k} - \varDelta E_{g,j}}{k_B T} \right] , \end{aligned}$$

where *j* and *k* denote the excited levels, $\varUpsilon$ is the thermally averaged collision strength, and $k_B$ is the Boltzmann constant. The ratio is temperature-dependent if the thermal energy of the electrons is much smaller than the difference between the excitation energies

106 $$\begin{aligned} \frac{\varDelta E_{g,k} - \varDelta E_{g,j}}{k_B T} \gg 1. \end{aligned}$$

This method of determining $T_{\mathrm{e}}$ was first used in solar physics by Heroux et al. (1972) using Li-like ions and Flower and Nussbaumer (1975c) using Na-like ions. One problem with this method is that normally such spectral lines are far apart in wavelength, which often means that they need to be observed by different instruments.

Alternatively, one of the lines which is observed might be collisionally excited from the ground level, but radiatively decay to an excited level. The temperature sensitivity will remain the same, but the two spectral lines might be closer in wavelength.

In general, such line ratio methods have been applied mostly to lines formed in the transition region, where non-equilibrium ionisation effects are likely to be important. Another major problem is that the temperature variation in the transition region is very steep, so different lines from the same ion are naturally emitted in spatially different regions, as pointed out for the Si iii case in Del Zanna et al. (2015c). Therefore, it is not surprising to see that different line ratios imply different temperatures, as is often found in the literature.

As with the density case, a useful way to measure temperatures when there are observations of more than two lines is to plot the emissivity ratios as a function of temperature, although they obviously also have the same limitations as the line ratios.

We now briefly review a few diagnostics that have been explored in the literature. Compared to the density case, there are relatively few results on this topic.

#### $T_{\mathrm{e}}$ from He-like ions

**Fig. 90:**
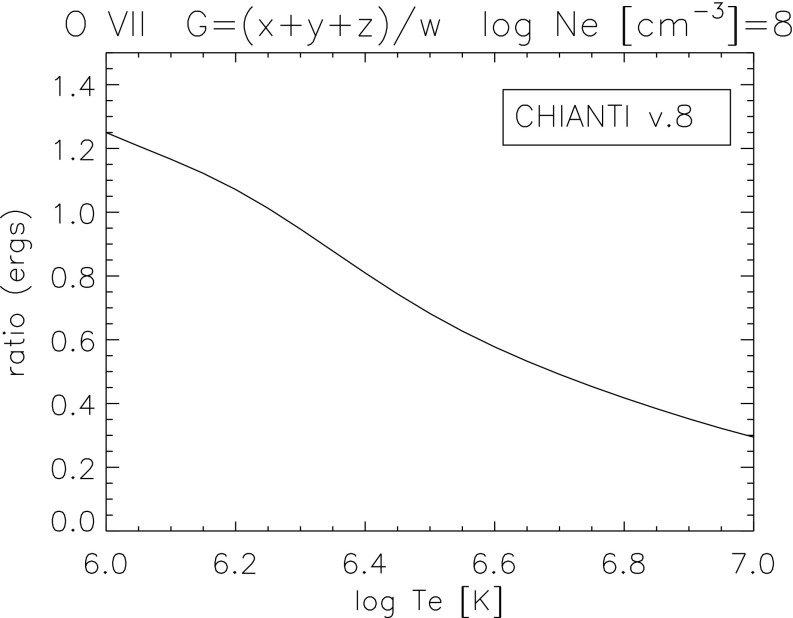
G-ratio for the He-like O vii

Gabriel and Jordan (1969) discussed the use of the so called G ratio $(x+y+z)/w$ (recall Fig. 62 and Table 6) of the intercombination and forbidden lines versus the resonance line *w* as a good temperature diagnostic for the helium-like ions, as it does not have a strong dependence on electron density. There is an extended literature on the use of the G ratios; see for example (Keenan et al. 1990) for Si xiii, (Keenan et al. 1992b) for Mg xi. Figure 90 shows O vii as an example.

**Fig. 91:**
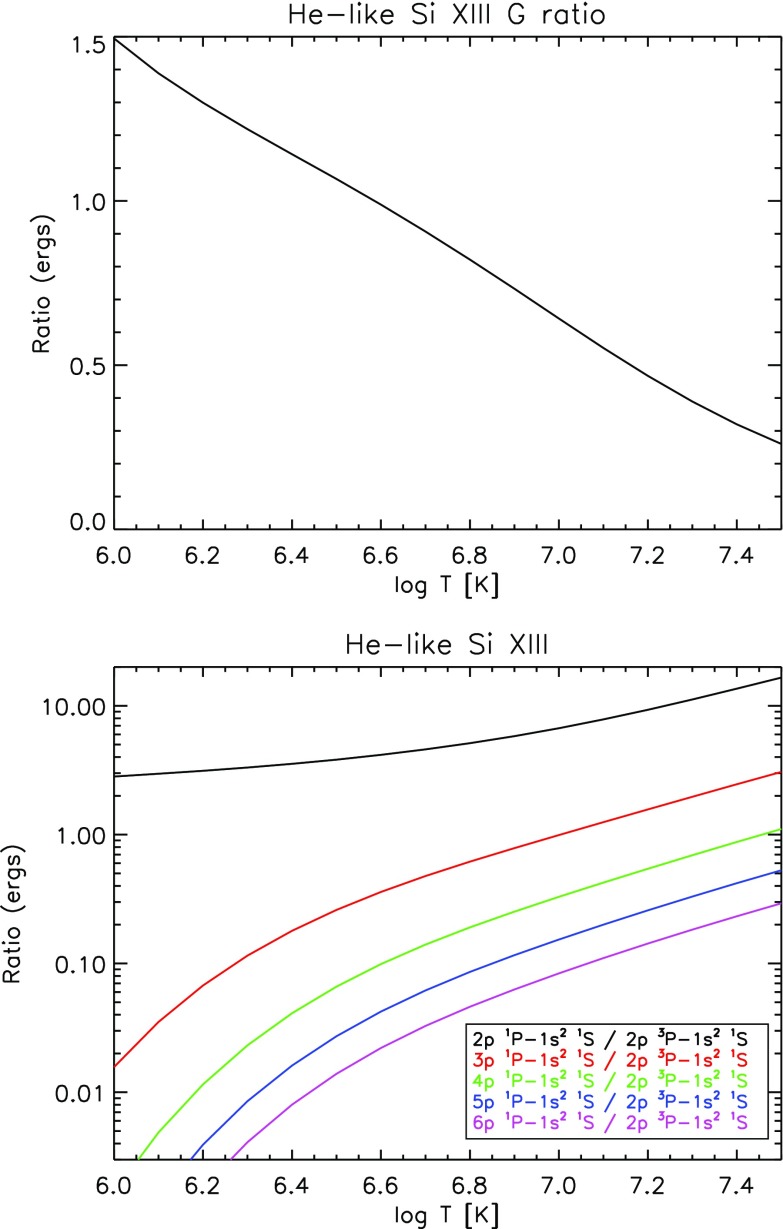
Top: G-ratio for Si xiii. Bottom: other temperature-sensitive ratios from highly excited levels

It has been known that ratios of the decays from the 3*p*, 4*p*, 5*p*,  etc. to the decay from the 2*p* to the ground state are also temperature sensitive (see, e.g., Keenan et al. 1990, for a discussion on Si xiii). Figure 91 (right) shows these ratios for Si xiii, as obtained from the atomic data discussed in Fernández-Menchero et al. (2016a). They are in principle better than the G-ratio (shown on the left of the figure) for two main reasons. First, the ratios vary more than the G-ratio, as is clear from the figure. Second, the resonance and forbidden lines are more difficult to calculate accurately. For example, the contribution of the satellite lines (not included in the plot), which is particularly important for the resonance line, needs to be included in the modelling. One drawback of the transitions from highly excited levels is that these lines are weaker.

Kepa et al. (2006) however found significant discrepancies between observed and predicted ratios in various He-like ions during the impulsive phase of solar flares observed with RESIK. Previously, discrepancies were also noted (see, e.g., Keenan et al. 1990). Therefore, the results obtained from these ratios should be treated with caution until these discrepancies are understood. The atomic data for these highly excited states have been reviewed by Fernández-Menchero et al. (2016a), where the possibility that non-Maxwellian electron distributions would increase the ratios was investigated. The non-Maxwellian distributions only increased the ratios at lower temperatures, so other processes are likely increasing these ratios.

#### $T_{\mathrm{e}}$ from Li-like ions

Heroux et al. (1972) and other authors have proposed the use of 2*s*–2*p*/2*s*–3*l* (${l}=s,p,d$) ratios of Li-like ions to obtain information about the temperature. However, the temperature sensitivity of these transitions is so different that the lines could easily be formed over different temperatures. The *G*(*T*) of these lines are so extended that it is possible that the lines are not formed within the same volume, hence a temperature obtained from the ratio does not have much physical meaning. Indeed Heroux et al. (1972) did not obtain a temperature from the observed ratios (obtained with an excellent rocket spectrograph that observed the Sun as a star), but rather predicted the intensities of the lines using a *DEM* obtained from other lines. With the ionization equilibrium tables available at the time, relative good agreement was found. Heroux et al. (1972) discuss the various lines and possible blending issues. Examples are the O vi 1032, 173 Å lines, later observed by SOHO SUMER and CDS/GIS (David et al. 1998), as discussed below. Off-limb measurements of the quiet Sun are clearly an opportunity to obtain direct measurements without depending too much on the *DEM*, since plasma is normally nearly isothermal. Del Zanna (2012a) showed that the Mg x soft X-ray lines have some temperature sensitivity.

#### $T_{\mathrm{e}}$ from Be-like ions

**Fig. 92:**
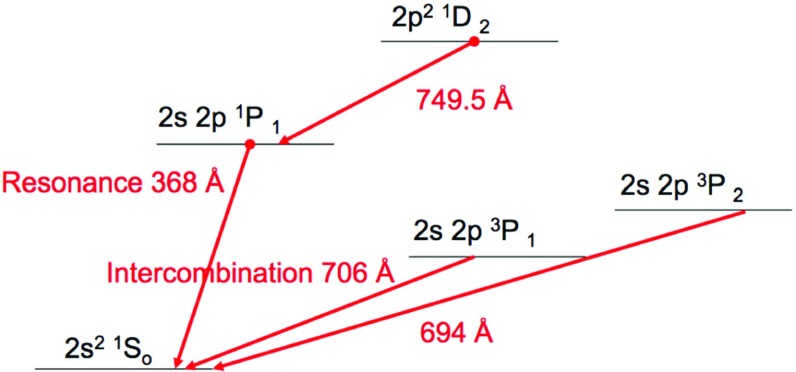
Diagram (not to scale) of the main diagnostic levels for Mg ix

The Be-like ions offer excellent temperature diagnostics, in particular with the ratios of the strong resonance and intercombination lines, which have very little density sensitivity and are very strong. The atomic structure of Be-like ions is such that the intercombination line has a wavelength about twice the resonance line, so both lines appear close in wavelength, if the instrument is sensitive to second order diffraction. A list of the main ions is provided in Table 17, while Fig. 92 shows a sketch of an energy level diagram with the main diagnostic levels listed in the table, for Mg IX.

Of all the Be-like ions, Mg ix is the one formed closest to the average coronal temperature (1 MK), so it is the best diagnostic for the average solar corona. The lines at 368.07, 706.06Å have been observed with e.g., SOHO CDS GIS (see, e.g., Del Zanna 1999; Del Zanna et al. 2008). The resonance line is somewhat blended though with several transitions, depending on the instrument resolution. Excellent agreement between observations and theory has been found only recently with new atomic data, as described below.

A good temperature diagnostic for the TR is the ratio of the O v 1218 Å with the resonance line at 629 Å (see, e.g., Keenan et al. 1994c). Dufton et al. (1978) used N iv and Ne vii Skylab observations of temperature-sensitive ratios to find values close to the peak abundance in ionization equilibrium. Keenan (1991) used Skylab observations of various features and obtained from three temperature-sensitive Ne vii ratios values close to the peak abundance in ionization equilibrium ($\log T=5.7$). A similar study on N iv was carried out by Keenan (1990).

**Table 17:** Be-like temperature diagnostics

Ion	$2{s}^{2}\,{}^1\hbox {S}_{0}{-}2{s}\,3{p}\,{}^1\hbox {P}_{1}$	$2{s}^{2}\,{}^1\hbox {S}_{0}{-}2{s}\,2{p}\,{}^1\hbox {P}_{1}$	$2{s}^{2}\,{}^1\hbox {S}_{0}{-}2{s}\,2{p}\,{}^3\hbox {P}_{1}$	$2{s}\,2{p}\,{}^1\hbox {P}_{1}{-}2{p}^{2}\,{}^1\hbox {D}_{2}$	$\mathrm{Log}\,T$
	$\lambda _1$	$\lambda _2$	$\lambda _3$	$\lambda _4$	
C iii	386.20 w	977.02	1908.73	2297.58 w	4–4.8
N iv	247.20	765.15	1486.50	1718.55 w	4–5.2
O v	172.17	629.73	1218.34	1371.30	4–6
Ne vii	97.49 w	465.22	895.17	973.33 (bl?)	5–6
Mg ix	62.75	368.07 (bl)	706.06	749.55	5–7
Al x	72.99 w	332.79	637.76	670.05	5–7
Si xi	43.75	303.32 (bl He ii)	580.92	604.15	5–7
S xiii	32.24	256.68	491.46	500.33 w	6–7
Ar xv	221.13	423.97	*u*	*u*	6–7
Ca xvii	19.56 w	192.85 (bl)	371.05	357.79 w	6–8
Fe xxiii	11.02	132.91 (bl)	263.76	221.34 w	6–8
Ni xxv	9.35	117.94	238.86	188.15 w	6–8

The ratios of the resonance ($\lambda _2$) and intercombination ($\lambda _3$) lines are temperature-sensitive. The ratios of the lines in the fifth column ($\lambda _4$) with the intercombination ($\lambda _3$) lines are also temperature-sensitive. Finally, another possibility is to use the ratios of the lines in the first column ($\lambda _1$) with the resonance lines. The $\log T$ values (K) in the last column indicate the approximate range where the ions can be used. Lines denoted with ‘bl’ are blended, those with ‘w’ are so weak that we are not aware of any observed intensities. Wavelengths are in Å

*u* unknown

The ratio of the resonance (192.8 Å) with the intercombination line (371.1 Å) in Ca xvii can in principle be used to measure the temperature (see, e.g., Dufton et al. 1983b). The resonance line is however blended with strong Fe xi and O v transitions (cf. Hinode EIS spectra, Del Zanna et al. 2011b) so accurate deblending needs to be applied to the observations. The resonance/intercombination ratio for Ar xv is discussed in Keenan et al. (1989c). Good agreement between theory and Skylab observations was found. A similar study on S xiii, with similar conclusions, was presented in Keenan et al. (1988b). The ratio of the intercombination with the resonance line in Fe xxiii is a very good temperature diagnostic, although the resonance line is blended with a relatively strong Fe xx line. Examples are provided, e.g., in Keenan et al. (1993b) and Del Zanna et al. (2005).

Another diagnostic possibility is the ratio of the $2s\,2p \,^1\hbox {P}_{1}-{}2{p}^2\,{}^1\hbox {D}_{2}$ transition with the intercombination. For a recent review of this ratio see Landi et al. (2001). Table 17 lists the main ions. The advantage of this ratio is that the lines are close in wavelength, and as in the previous case the ratio is insensitive to density. However, the $^1\hbox {P}_{1}{-}^1\hbox {D}_{2}$ transitions are extremely weak and only those for a few ions have been observed. The Mg ix ratio has been applied to SOHO SUMER measurements, but will also be available to the Solar Orbiter SPICE spectrometer, in off-limb observations, since on-disk the weak 749.55 Å line is blended with cool emission.

A third diagnostic possibility is the ratio of the $2{s}^2\,{}^1\hbox {S}_{0}{-}2{s}\,3{p}\,{}^1\hbox {P}_{1}$ with the resonance line. This is similar to the Li-like one. The downside is that lines are very far in wavelength, so only some simultaneous observations exist. Malinkovsky et al. (1973) used the O v [629/172 Å] ratio to find an average temperature $\log T=5.4$ from the full-Sun grazing-incidence spectrum. Finally, Keenan et al. (2006b) and Del Zanna (2012a) showed that the Mg ix soft X-ray lines have some temperature and density sensitivity, but it is only with recent atomic data that good agreement with observations has been found.

#### $T_{\mathrm{e}}$ from B-like ions

Dwivedi and Gupta (1994) suggested using the ratio of the O iv 790/554 Å multiplets to measure TR temperatures. O’Shea et al. (1996) used Skylab S-055 observations of the ratio of the O iv 790/554 Å multiplets to find averaged temperatures close to those of peak abundance in ionization equilibrium. Peng and Pradhan (1995) suggested the use of similar ratios in other ions along the sequence, but it turns out that some are sensitive to density and the variation with temperature is very small. Doron et al. (2003) suggested the use of the N iii lines around 990 Å to infer temperatures (see below). Keenan et al. (1995b) investigated the use of Ne vi line ratios using Skylab observations, but found significant discrepancies between theory and observations, mostly due to blending and temporal variability. Foster et al. (1997) studied the diagnostics available within Mg viii and found several useful ratios in the EUV. However, all ratios depended on both density and temperature. The approach suggested is to use ratio-ratio plots to infer both temperatures and densities at the same time.

**Table 18:** Temperature diagnostics for C-like, Mg-like, and Al-like ions

Transition 1	$\lambda _1$ (Å)	Transition 2	$\lambda _2$ (Å)	Ion	$\mathrm{Log}\,T_{\mathrm{e}}$
$2{s}^2\,2{p}^2\,{}^1\hbox {D}_{2}{-}2{s}\,2{p}^3\,{}^1\hbox {D}_{2}$	599.59	$2{s}^2\,2{p}^2\,{}^3\hbox {P}_{1}{-}2{s}\,2{p}^3\,{}^3\hbox {D}_{1,2}$	833.7 (sbl)	O III	4–5.5
$2{s}^2\,2{p}^2\,{}^1\hbox {D}_{2}{-}2{s}\,2{p}^3\,{}^1\hbox {D}_{2}$	599.59	$2{s}^2\,2{p}^2\,{}^3\hbox {P}_{2}{-}2{s}\,2{p}^3\,{}^3\hbox {D}_{1,2,3}$	835 (sbl)	O III	4–5.5
$2{s}^2\,2{p}^2\,{}^1\hbox {D}_{2}{-}2{s}\,2{p}^3\,{}^1\hbox {D}_{2}$	416.21	$2{s}^2\,2{p}^2\,{}^3\hbox {P}_{2}{-}2{s}\,2{p}^3\,{}^3\hbox {D}_{3}$	572.34	Ne V	4.5–6.
$2{s}^2\,2{p}^2\,{}^3\hbox {P}_{2}{-}2{s}\,2{p}^3\,{}^3\hbox {S}_{1}$	278.40 (bl)	$2{s}^2\,2{p}^2\,{}^3\hbox {P}_{2}{-}2{s}\,2{p}^3\,{}^3\hbox {D}_{3}$	434.92	Mg VII	5–6.5
$3{s}^2\,{}^1\hbox {S}_{0}{-}3{s}\,3{p}\,{}^1\hbox {P}_{1}$	1206.50	$3{s}^2\,{}^1\hbox {S}_{0}{-}3{s}\,3{p}\,{}^3\hbox {P}_{1}$	1892.03	Si iii	4.3–5.5
$3{s}^2\,{}^1\hbox {S}_{0}{-}3{s}\,3{p}\,{}^1\hbox {P}_{1}$	786.47	$3{s}^2\,{}^1\hbox {S}_{0}{-}3{s}\,3{p}\,{}^3\hbox {P}_{1}$	1199.13	S v	4.5–5.7
$3{s}\,3{p}\,{}^1\hbox {P}_{1}{-}3{s}\,3{d}\,{}^1\hbox {D}_{2}$	696.62	$3{s}^2\,{}^1\hbox {S}_{0}{-}3{s}\,3{p}\,{}^1\hbox {P}_{1}$	786.47	S v	4.5–5.7
$3{s}^2\,3{p}\,{}^2\hbox {P}_{1/2}{-}3{s}^{2}\,3{d}\,{}^2\hbox {D}_{3/2}$	657.319	$3{s}^2\,3{p}\,{}^2\hbox {P}_{1/2}{-}3{s}\,3{p}^{2}\,{}^2\hbox {D}_{3/2}$	1062.664	S iv	4.5–6.

The $\log T_{\mathrm{e}}$ values (K) indicate the range of sensitivity. Wavelengths are in Å. bl indicates that the transition is blended. sbl indicates that the line is a self-blend of transitions from the same ion

#### $T_{\mathrm{e}}$ from C-like ions

Keenan and Aggarwal (1989) studied the Te-sensitive 703/599.6 and 834/599.6 Å line ratios from the C-like O iii. They considered the quiet Sun Skylab observations of Vernazza and Reeves (1978) and obtained isothermal temperatures in the range $\log T\,(\mathrm{K})=4.7{-}5.2$, i.e., close to the maximum ion abundance value in ionisation equilibrium ($\log T\,(\mathrm{K})=5.0$). However, the temperature sensitivity of the 703/599.6 Å ratio is very small around those temperatures, so the ratios 834/599.6 Å or even better the 835/599.6 Å are to be preferred. This and similar ratios along the isoelectronic sequence are shown in Table 18. Keenan et al. (1989b) suggested the use of several O iii lines in the 500–600 Å range to measure temperatures, and obtained, using observations from the Skylab slitless spectrograph, temperatures close to $10^5$ K, i.e., around the peak abundance in equilibrium. However, at such temperatures, recent atomic data show that the variations of the suggested ratios are very small, hence these ratios are not very useful temperature diagnostics. Keenan et al. (1986a) suggested the use of either the Ne v 569.8 or 572.3 Å versus the 416.2 Å line to measure temperatures, since these ratios have a small density sensitivity at the typical densities where this ion is formed. These authors used Skylab observations of a flare and a prominence obtained with the NRL slitless spectrometer and found values close to the peak temperature in equilibrium ($\log T\,(\mathrm{K})=5.5$). Between the two lines, the 572.3 Å is a better choice because the 569.8 Å line is weaker and blended. The 572.3 Å versus the 416.2 Å ratio is indeed a very good one for the TR. Keenan et al. (1992a) suggested the use of further line ratios from this ion, but their temperature sensitivity is not very good. We found that another good temperature diagnostic is the Mg vii ratio of the 278.404 versus the 434.923 Å lines, although the 278.404 Å line is blended with a Si vii line even at high resolution.

#### $T_{\mathrm{e}}$ from Na-like ions

Flower and Nussbaumer (1975c) suggested the use of ratios of lines from the 3*s*–3*p* and 3*p*–3*d* in Na-like ions, because these lines fall relatively close in wavelength. They applied the technique to Si iv (3*p*–3*d*)/(3*s*–3*p*) [1128.3/1393.8] and Si vi lines, but did not obtain agreement between theory and observations, because it turns out that lines are blended in low-resolution spectra.

Keenan et al. (1986c) and Doschek and Feldman (1987a) studied Al iii, obtaining some results (see below). Keenan et al. (1986c) also studied Si iv, but concluded that higher-resolution spectra for the Si iv lines (1128.3, 1393.8, 815.0 Å) were needed. Keenan and Doyle (1988) later actually obtained good agreement between theory (using updated calculations) and observations of the Si iv multiplets at 1120 and 815 Å for a solar active region at the limb obtained with the Harvard S-055 spectrometer on board Skylab, but noted that the 1122.5 Å must be severely blended with a cool emission line. Keenan et al. (1994b) investigated the use of Ca x line ratios using Skylab observations of flares with the NRL slitless spectrometer, but found several discrepancies, which they ascribed to blending problems. Indeed the 419.74 Å line is blended with a strong C iv, and the 574.0 Å line with O iii.

Keenan et al. (1994a) suggested the use of Fe xvi line ratios involving the 251, 263, and 335.4 Å lines, and compared predicted with observed values obtained with the Skylab NRL slitless spectrometer. Some discrepancies were found, and ascribed to a problem in the calibration of the strong 335.4 Å line (which is also blended). However, aside from these discrepancies, the main limitation of such ratios is that they have very little temperature sensitivity when this ion is formed in ionization equilibrium. Keenan (1988) studied the S vi 712.8/944.5 Å ratio, but concluded that both lines are blended at the resolution of the Skylab Harvard S055 spectrometer.

#### $T_{\mathrm{e}}$ from Mg-like ions

The ratio between the intercombination and resonance line in Si iii is in principle a good temperature diagnostic. However, the ratio is also sensitive to densities above $10^{10}\,\hbox {cm}^{-3}$, so the density has to be measured independently (see Del Zanna et al. 2015c, for a recent review on this ion).

Doyle et al. (1985b) and Keenan and Doyle (1990) studied temperature-sensitive S v line ratios, finding relatively good agreement with theory. The ratios involved the resonance line at 786 Å and the 663.2, 852.2, and 854.8 Å lines. However, these ratios have a very small temperature sensitivity around $10^5$ K where this ion is formed. We suggest that a much better ratio is when the 696.62 Å is considered. Alternatively, the ratio with the UV line at 1199.13 Å is also a good temperature diagnostic, unless very high densities above $10^{12}\,\hbox {cm}^{-3}$ are reached, in which case the ratio also becomes density-sensitive. The main ratios we recommend are listed in Table 18.

#### $T_{\mathrm{e}}$ from Al-like ions

Doyle et al. (1985a) pointed out that ratios of S iv multiplets at 657, 750, 810 Å with the 1070 Å multiplet are temperature-sensitive. Several combinations are available, also involving the strongest line, at 1406 Å. One of the best ratios is the 657.319 versus 1062.664 Å, given that this has no density sensitivity below $10^{12}\,\hbox {cm}^{-3}$. The 657.319 Å line is the resonance (strongest) line for this ion. The analogous ratio for Fe xiv (211.32 vs. 334.18 Å) does not vary much with temperature.

#### $T_{\mathrm{e}}$ from S-like ions

Doschek and Feldman (1987a) suggested that ratios of lines from S iii $3{s}^2$ 3*p* 4*p* levels (e.g., 1328.12, 1328.52 Å) with those from the 3*s* $3{p}^3$ levels (i.e., the strong line at 1200.96 Å) would be a good temperature diagnostic, because these lines are close in wavelength.

#### $T_{\mathrm{e}}$ from iron ions

Given that iron lines are very abundant in XUV spectra, it would clearly be very useful to use diagnostics based on them. There are many possible diagnostics, but most of them involve lines at very different wavelengths, so results have been scarce. Table 19 lists the main diagnostics which are available for Fe x, Fe xi, Fe xii, and Fe xiii. In principle, very good diagnostics are offered by the ratio of one of the forbidden lines within the ground configuration ($\lambda _4$ in Table 19) and one of the strongest dipole-allowed EUV lines ($\lambda _1$ in Table 19). Such measurements are however not easy, as it is not simple to obtain well-calibrated simultaneous observations at very different wavelengths. Also, the accuracy of atomic data plays an important role: the recent large-scale calculations for these iron ions (discussed in the atomic data Sect. 5) have modified the predicted intensities of the forbidden lines of the iron coronal ions by large factors (2–3, relative to the dipole-allowed lines).

**Table 19:** Temperature diagnostics from coronal Fe ions

Ion	$\lambda _1$ (Å)	$\lambda _2$ (Å)	$\lambda _3$ (Å)	$\lambda _4$ (Å)	$\mathrm{Log}\,T$
Fe viii	186.6, 185.21	130.94	253.96, 255.11		5.7
			255.35, 255.68		
Fe ix	171.07	188.49, 197.85			5.8
Fe x	174.53, 184.53	257.26 (sbl)	345.74	6376	6.0
Fe xi	180.40, 188.22	257.55 (sbl)	352.67	2649.50	6.1
Fe xii	193.51, 192.39		364.47	1242.0, 1349.4	6.2
				2169.76, 2406.41	
Fe xiii	202.04			10,749	6.3

Three main wavelength ranges (values in Å) are shown. Any ratio among them is a good temperature diagnostic. Only the strongest lines are shown. The first group of lines ($\lambda _1, \lambda _2, \lambda _3$) are the strongest lines in the EUV, while the UV/visible lines ($\lambda _4$) are the forbidden lines within the ground configuration. The $\log T$ values (K) indicate the approximate formation temperature of the ions

On the other hand, there are several other ratios of iron lines which are closer in wavelength and which are temperature sensitive. Some ratios for Fe x, Fe xi, Fe xii (see Table 19) involve lines around 180 and 350 Å, listed in the $\lambda _1, \lambda _3$ columns in Table 19. These diagnostics have been known for some time, but lines have usually been observed by different instruments. Note that the recent large-scale calculations for these iron ions have modified the predicted intensities of the $\lambda _3$ lines by typically 30%.

Several other ratios offer temperature diagnostics, including ratios where both lines were observed by the same instrument, Hinode EIS. These have recently been identified: Fe viii (Del Zanna 2009b); Fe ix (Young 2009; Del Zanna 2009a); Fe xi (Del Zanna 2010). Some of them are listed in the $\lambda _1, \lambda _2$ columns of Table 19. Also in this case, we point out that it was only recently, with large-scale scattering calculations, that significant discrepancies between observed and predicted line intensities were resolved for Fe viii (Del Zanna and Badnell 2014), Fe ix (Del Zanna et al. 2014b), Fe xi (Del Zanna et al. 2010b). This improvement was due to the increases in intensities, by 30–50%, for the lines listed in the $\lambda _2$ column. Temperature measurements from these ions therefore now appear to be feasible, although results also depend on an accurate radiometric calibration of Hinode EIS. This turns out to be particularly important as the sensitivity of these ratios is not as large as that available when the forbidden lines are used. Line ratios useful for measuring the electron temperatures from Fe xvi, Fe xvii, Fe xviii, Fe xxiii are briefly discussed below in Sect. 12.3.

### *T* from ratios of two lines from different ions

Another way to estimate temperatures is to use the observed ratio of two line intensities produced by two ionisation stages of the same element. If ionisation equilibrium is assumed, the observed ratio can then be directly compared to the theoretical ratio, and a temperature obtained. This method has been widely used in the past, although its accuracy relies on the reliability of the ionisation/recombination rates and, most of all, on the ionisation equilibrium assumption. This last assumption is usually reasonable in the solar corona, but in the transition region it is clear that dynamical timescales are often shorter than recombination times, hence departures from ionisation are to be expected.

### *T* from several lines

Whenever more than two lines from different ionisation stages of the same element are observed, one could obtain an estimate of the temperature from the line ratios assuming ionisation equilibrium, and compare the results. If all the temperatures agree, the plasma is likely to be isothermal. A more elegant way to analyse the data is to use the EM loci method, which we discussed in Sect. 7.2.1. If the EM loci curves cross at one temperature, it means that the data are consistent with the plasma being isothermal (although that is not necessarily the only solution). If no crossing is obtained, it means that the emission is multi-thermal, and a *DEM* analysis should be carried out.

### *T* from dielectronic satellite lines

We have seen in Sect. 4 that the ratio of a satellite line formed by dielectronic capture with the resonance line is an excellent temperature diagnostic, because it is independent of the population of the ions and the electron density. The main problems with such measurements are that very high resolution spectra are needed, and the contribution of the satellite lines to the resonance line needs to be taken into account.

### *T* from the X-ray continuum

The spectral distribution of the X-ray continuum depends quite strongly on the electron temperature, so in principle this continuum could be used to measure the electron temperature. For example, it is very common to obtain an isothermal temperature from the ratio of the GOES X-ray fluxes during a solar flare. The GOES fluxes are dominated by the continuum, which is mostly free–free emission for the largest flares. However, some contribution from the free–bound emission is normally also present (recall Sect. 3.7). While the free–free is mostly due to hydrogen, hence is directly proportional to the hydrogen (proton) density, the free–bound depends on the chemical abundances, so in principle an independent estimate of the composition of the plasma (see Sect. 14) should be carried out when measuring the temperature.

Direct measurements of the spectral distribution of the X-ray continuum are difficult, and only a few observations are available. Parkinson et al. (1978) was able to obtain with excellent OSO-8 X-ray spectra both measurements of continuum and line intensities of a small flare and an active region after the flare. An isothermal model was able to reproduce both the continuum and the ratio of the H-like to He-like Si (i.e., Si xiv vs. Si xiii). The RESIK spectrometer observed the spectral range where a significant temperature sensitivity of the continuum exist.

**Fig. 93:**
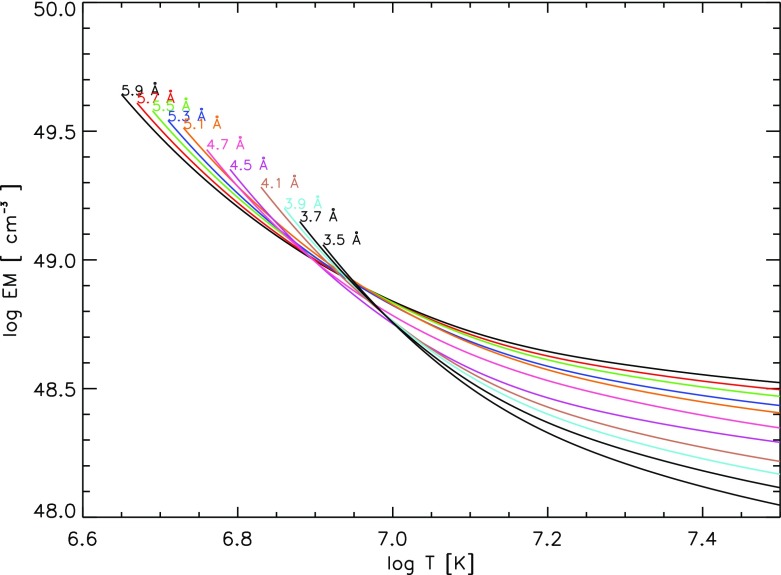
EM loci curves for the X-ray continuum during the peak phase of a flare observed with the RESIK spectrometer (adapted from Chifor et al. 2007). The crossing indicates an isothermal temperature of almost 10 MK

A diagnostic similar to the EM loci curves, but applied to the X-ray continuum, was devised by one of us (GDZ) and applied to the X-ray continuum measured during a flare by the RESIK spectrometer. The emissivity of the continuum was calculated as a function of temperature for the observed RESIK wavelengths using the CHIANTI database, and compared to the observed continuum, by plotting the ratio of the observed intensities with the emissivity. Figure 93 shows the results for the peak phase of a flare. As we discussed in the instrument’s Sect. (2), the RESIK instrumental fluorescence created a complex background emission which needed to be subtracted to reliably use the continuum for diagnostic purposes. This added some extra uncertainty on the continuum measurements. However, as described in Chifor et al. (2007), the continuum EM loci curves were consistent with a nearly isothermal plasma, in good agreement with the results of the line EM loci curves. Furthermore, the isothermal temperature was decreasing during the flare.

## Measurements of electron temperatures

In what follows, we provide several examples of measurements of electron temperatures for different solar regions, with emphasis on more recent results, to show which diagnostics have been applied. We stress that the types of measurements described in this section are fundamental to constrain the wide-range of coronal heating and solar wind theories (see, e.g., Cranmer 2009).

### *T* in coronal holes

Measurements of electron (and ion) temperatures in coronal holes are very important, especially when defining the lower boundary of models which attempt to explain how the fast solar wind becomes accelerated inside coronal holes (see, e.g., the Living Reviews by Cranmer 2009; Poletto 2015).

Estimates of the coronal temperatures have historically been obtained from the ratios of lines emitted by different ions or DEM analyses. These were effectively ‘ionisation’ temperature, and not strictly electron temperatures. A good review of the range of temperatures that have been obtained from remote measurements of coronal holes before SoHO was given by Habbal et al. (1993). Coronal temperatures were found to be about 1 MK, but with a large scatter and significant uncertainties. One of the issues was, and still is, how much of the emission observed off-limb in coronal holes originates from the foreground/background quiet Sun, and whether the plasma emitted by different ions is co-spatial.

Esser et al. (1995) analysed daily intensity measurements at 1.15$R_\odot$ of the green Fe xiv 5303 Å and red Fe x 6374 Å lines. Large temperature variations (more than 0.8 MK) were found over the time interval of the observations which covered about four solar rotations. They also discussed how the regions surrounding the coronal holes might have influenced the temperature measurements.

Guhathakurta et al. (1992) derived temperatures from the green Fe xiv 5303 Å and red Fe x 6374 Å forbidden lines, together with a broad-band image centred at 173 Å and recorded by a sounding rocket. They found an average coronal temperature of 1.34 MK for a south polar coronal hole and 1.27 MK for a north polar coronal hole, at 1.15$R_\odot$, during the 1988 March 17–18 solar eclipse.

#### *T* from SOHO observations

SOHO enabled more direct measurements of electron temperatures. Also, coronal hole plumes could be differentiated from the inter-plume regions (lanes). There were several studies on coronal hole and plume temperatures obtained from various methods. The most common ones were based on emission measure analyses, see e.g., the ‘Elephant’s trunk’ equatorial coronal hole measurements (Del Zanna and Bromage 1999b). The plume bases were shown to be nearly isothermal, at temperatures below 1 MK (cf. Del Zanna et al. 2003). Off-limb SUMER observations near the limb indicated nearly isothermal plasma at a temperature just below 1 MK (Feldman et al. 1998b). For a recent review on coronal hole measurements, see Wilhelm et al. (2011).

Measurements from line ratios, which are in principle more accurate, were also obtained. The Mg ix lines around 700 Å are potentially an excellent temperature diagnostic, available for SOHO SUMER and CDS observations. The Mg ix resonance and intercombination lines are also an excellent temperature diagnostic, as shown in Del Zanna et al. (2008) using SOHO/GIS observations, where the resonance 368.07 Å line is observed in second order near the intercombination line at 706.06 Å. Wilhelm et al. (1998) presented SOHO SUMER off-limb (in the range 1.03–1.6 $R_\odot$) observations of plume and inter-plume regions, and measured temperatures using the Mg ix 749/706 Å line ratio. Plume temperatures were found to be approximately constant around $T=780{,}000$ K and then decrease at higher radial distances, while inter-plume temperatures were found to increase over the same height range. These results were limited by the fact that plumes and inter-plume regions were compared using data over a time span of 6 months, and at different spatial locations. Another limiting factor was the use of atomic data that were interpolated, not calculated. The first *R*-matrix scattering calculation for Mg ix was carried out by Del Zanna et al. (2008). This new atomic data produced significantly different temperatures. For example, a coronal hole inter-plume temperature of 850,000 K found by Wilhelm et al. (1998) was revised to 1,160,000 K.

David et al. (1998) used the O vi 173/1032 Å ratio to measure temperatures from off-limb spectra in coronal holes and quiet Sun areas. Close to the limb, coronal holes were found to be about 0.8 MK, rising to about 1 MK around 1.15 $R_\odot$, and then decreasing down to about 0.4 MK at 1.3 $R_\odot$. However, these results should be treated with caution for several reasons. First, SOHO was rotated and the slit were therefore tangential to the limb of the Sun, i.e., plume and lanes could not easily be distinguished. Second, the observations were obtained by two different instruments (CDS/GIS and SUMER), with an uncertain relative calibration. This was not in principle a major problem since the measurements were made relative to the quiet Sun. However, the GIS observations used the long slit and not one of the usual pinhole slits. The spectra obtained with the pinhole slit have been calibrated in-flight (Del Zanna et al. 2001a; Kuin and Del Zanna 2007), but the long slit observations are impossible to calibrate, since the detectors were not fully illuminated in this case.

Finally, it is worth mentioning that SUMER (e.g., Hassler et al. 1997) and UVCS (e.g., Noci et al. 1997) off-limb observations have shown that the spectral line widths in the inter-plume lanes are larger than in the plumes, indicating lower ion temperatures in plumes as a possible explanation. Very large non-thermal widths in the O vi lines were also discovered by UVCS. They are signatures of strong anisotropies, much larger than those of the protons, already measured pre-SOHO. These observations have prompted new theories on the heating and acceleration of the fast solar wind. For more details, see the reviews by Kohl et al. (2006) and Cranmer (2009).

### *T* in the quiet Sun

There is a vast literature on ratios of lines emitted by different ions and emission measure analyses of quiet Sun areas, most of which indicate that the quiet Sun has a near isothermal DEM distribution around 1 MK (see, e.g., Feldman et al. 1999; Landi et al. 2002b; Young 2005a; Brooks et al. 2009). These results are not reviewed here since we focus on diagnostics from individual ions.

Keenan et al. (1986c) used Skylab NRL S082B spectra of the QS and obtained a range of values, $\log T\,(\mathrm{K})=4.4{-}4.7$, from the Al iii 3*p*–3*d* 1611.9 Å, 3*p*–4*s* 1379.7 Å, and 3*s*–3*p* 1862.8/1854.7 Å lines (in equilibrium the Al iii abundance peaks at $\log T\,(\mathrm{K})= 4.6$). Similar values were obtained by Doschek and Feldman (1987b), i.e., temperatures slightly below those of the ion peak abundance in ionisation equilibrium. However, slightly lower temperatures would be expected, given the steep gradient in the emission measure distribution.

Lines from Si iii have been used in the literature to measure temperatures, however as suggested by Doschek (1997) and discussed in detail in Del Zanna et al. (2015c) the line ratios lose meaning because of the steep temperature gradients where the Si iii lines are formed. Lines sensitive to different temperatures are likely to be formed in different layers of the atmosphere, so the line ratio method cannot really be applied.

Doron et al. (2003) obtained SUMER measurements of the B-like N iii lines around 990 Å and obtained, assuming isothermal emission, temperatures in the range $5.7\times 10^4{-}6.7\times 10^4$ K, considerably lower than the calculated temperature of maximum abundance of N iii, which is about $7.6\times 10^4$ K. The Na-like 3*p*–3*d* and 3*s*–3*p* transitions in Si iv required a UV instrument like SOHO SUMER. Doschek et al. (1997a) used SUMER measurements of the 1128.32, 1128.34, 1402.77 Å Si iv lines in the quiet Sun near disk centre and found temperatures systematically higher than the temperature of maximum ion abundance in equilibrium. Ahmed et al. (1999) on the other hand measured Si iv ratios involving the 1394, 1403 and 818 Å lines for the quiet Sun at disk centre with SOHO SUMER finding $\log T\,(\mathrm{K})=4.8$, which is close to the temperature of maximum ion abundance. Doschek (1997) pointed out that the uncertainty in the Skylab S082B calibration and the density sensitivity of the Be-like O v 1371.29A/1218.35 Å ratio makes the temperature results from this ion very uncertain. The uncertainty is even larger for the Be-like C iii 1247/1908 Å ratio.

Muglach et al. (2010) recently attempted a measurement of the QS temperature from ratios of O iv and O vi lines observed by Hinode EIS and SOHO SUMER in 2007; however, large discrepancies with theory were found, with measured ratios being factors between 3 and 5 less than those expected in ionisation equilibrium. The authors checked the relative radiometric calibration and increased the SUMER radiances by a factor of 1.3. However, the relative calibration of the instruments is probably a source of error. Indeed the Hinode EIS ground calibration was found to be incorrect even in early observations (Del Zanna 2013b).

In contrast, Giunta et al. (2012) analysed similar Hinode EIS and SOHO SUMER observations in 2009 of the same O iv 787.72/279.93 Å ratio and found reasonable temperatures in the range log $T=5.2{-}5.4$, close to those expected from ionisation equilibrium. The main difference was the adoption of a larger atomic model, and an increase of the SUMER radiances by a factor of 1.5. However, the Giunta et al. (2012) results are even more affected by the degradation of the EIS instrument and should be revisited.

**Fig. 94:**
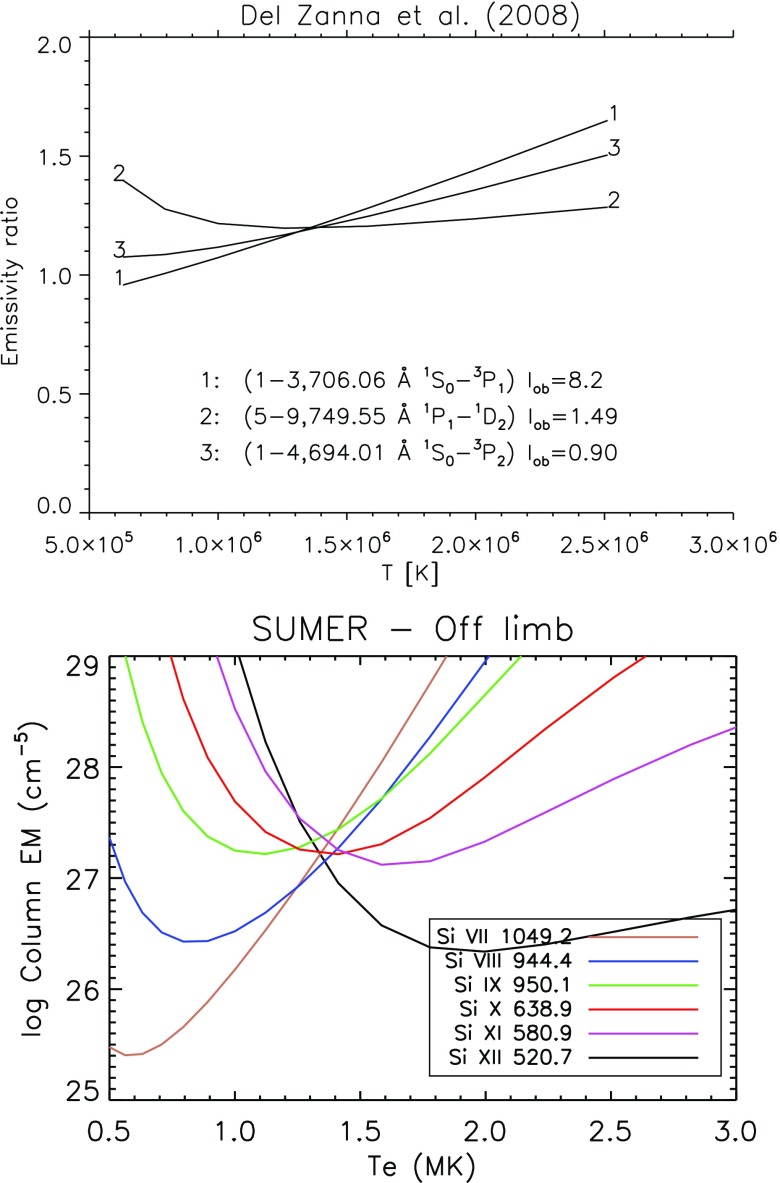
Top: emissivity ratio curves of Mg ix lines observed by SoHO SUMER in an off-limb quiet Sun area (image adapted from Del Zanna et al. 2008). Bottom: EM loci curves of silicon ions obtained from the same observation and CHIANTI v.8 data

Del Zanna et al. (2008) presented new *R*-matrix calculations for Mg ix and used them to obtain an isothermal temperature of 1.35 MK in an off-limb quiet Sun area measured with SoHO SUMER and discussed by Feldman et al. (1999) and Landi et al. (2002b). The emissivity ratio curves are shown in Fig. 94 (top). This temperature is in excellent agreement with the one obtained from the EM loci curves, which indicates that the corona is approximately isothermal, as already noted by Feldman et al. (1999). The EM loci curves shown in Fig. 94 (bottom) are obtained with CHIANTI v.8 data. We note that previous atomic data for Mg ix were interpolated and provided a lower temperature of 1 MK. We also note that the EM loci plot in Fig. 94 (bottom) is slightly different than the one presented by Feldman et al. (1999), because of the use of more recent atomic data.

### *T* in active regions and flares

For general reviews on studies of active regions and the importance of measuring temperatures see, e.g., Reale (2014) and Mason and Tripathi (2008).

Keenan and Doyle (1988) used Skylab Harvard S-055 spectra of an active region and a sunspot to derive $\log T\,(\mathrm{K})=4.6$ from the Si iv ratio of the 1128.3 and 818.1 Å lines. This temperature is only slightly lower than the equilibrium value of $\log T\,(\mathrm{K})=4.8$. Keenan and Doyle (1990) measured the temperature-sensitive ratios R1 $=$ 854.8/786.9 Å, R2 $=$ 852.2/786.9 Å, R3 $=$ 849.2/786.9 Å, and R4 $=$ 1199.1/786.9 Å using sunspot data obtained with the Harvard S-055 spectrometer on board Skylab. They found overall agreement between theory and observations, except in the case of R1, probably due to blending of the 854.8 Å line.

**Fig. 95:**
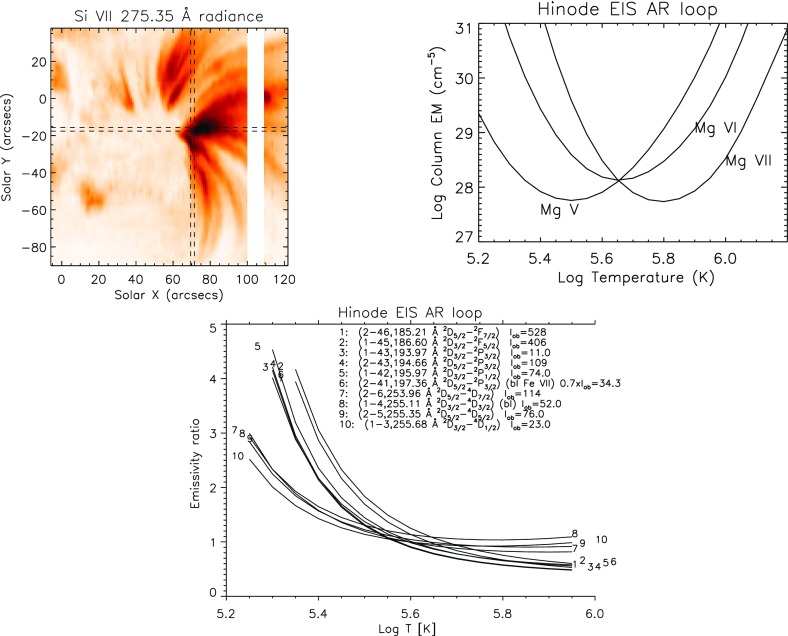
Top left: a negative monochromatic image in Si vii observed by Hinode EIS showing a fan of AR loops rooted in a sunspot; top right: the EM loci curves for Mg lines relative to a region near the base (indicated by a cross in the image). Bottom: emissivity ratio curves for the Fe viii lines, using the (Del Zanna and Badnell 2014) atomic data and the (Del Zanna 2013b) EIS calibration Images adapted from Del Zanna (2009b), Del Zanna and Badnell (2014)

Temperatures at the legs of AR loops obtained from Fe vii (Del Zanna 2009a) and Fe viii (Del Zanna 2009b; Del Zanna and Badnell 2014) line ratios observed by Hinode EIS are in reasonable agreement with those obtained from the EM loci curves, suggesting that the plasma is nearly isothermal and close to ionization equilibrium. One example is shown in Fig. 95. However, the temperature-sensitive lines are relatively weak and results depend quite critically on the EIS radiometric calibration.

Keenan et al. (1989c) used the 221.12 and 423.98 Å lines from the Be-like Ar xv and EUV observations of two flares with the Skylab NRL S082A slitless spectrograph, finding $\log T\,(\mathrm{K})=6.46, 6.32$, i.e., close to the temperature of peak abundance in equilibrium, $\log T\,(\mathrm{K})= 6.5$.

Keenan et al. (1994a) looked at the ratios of the Na-like Fe xvi 335.4 versus the 251, 263, and 265 Å lines observed in active regions and flares by the Skylab NRL S082A instrument. Some of the observations were consistent with isothermal temperatures of $\log T\,(\mathrm{K})=6.4$, i.e., close to the peak ion temperature in equilibrium. Keenan et al. (2007) compared Fe XVI observations with more recent atomic data, finding overall agreement. They used EUV SERTS active region observations and the soft X-ray XSST observation of a flare, but the temperature sensitivity was not discussed in detail.

SUMER measurements in the UV of flare temperatures were discussed in Feldman et al. (2000).

**Fig. 96:**
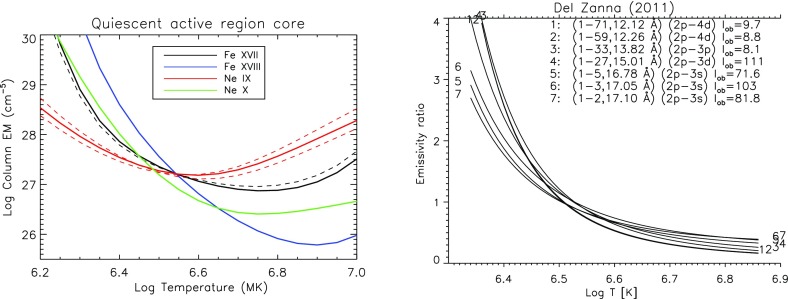
Left: EM loci curves of an active region core observed by Parkinson (1975). The EM loci curves have been recalculated with CHIANTI v.8. Right: emissivity ratio curves for the Fe xvii lines, using the Liang and Badnell (2010) atomic data Images adapted from Del Zanna (2011b)

The soft X-ray lines have some temperature sensitivity, but results only improve with accurate scattering calculations, as shown in Del Zanna (2012a), where the same XSST observation was analysed with different atomic data. Within the X-rays (5–50 Å), the strongest lines are those from the Ne-like Fe xvii around 15–17 Å. It has been known for a long time that these strong X-ray lines from Fe xvii could be used to measure temperatures. However, large discrepancies between theory and observations have also been present for a long time. There is an extended literature on this important diagnostic, where various attempts have (largely unsuccessfully) been made to explain the discrepancies. It was only with accurate *R*-matrix scattering calculations (Loch et al. 2006; Liang and Badnell 2010) that the discrepancies with the astrophysical observations have been resolved, as discussed in Del Zanna (2011b). Solar active region observations from SMM/FCS and an excellent spectrum obtained from a Skylark sounding rocket (Parkinson 1975) were used by Del Zanna (2011b) to obtain near-isothermal temperatures close to 3 MK, the typical formation temperature of the hot core loops. An example is shown in Fig. 96.

We recall (see Sect. 8) that the fact that quiescent active region cores have near-isothermal distributions around 3 MK was already indicated by Skylab X-ray imaging (Rosner et al. 1978), and then later confirmed with X-ray (e.g., SMM FCS, Yohkoh BCS) and EUV/UV (e.g., SoHO CDS, SUMER) spectroscopy.

Significant discrepancies between theory and observations for the Fe xviii lines also existed for a long time. Del Zanna (2006a) showed that the X-ray lines from Fe xviii can reliably be used, in conjunction with accurate *R*-matrix scattering calculations (Witthoeft et al. 2006), to measure temperatures in solar flares. Values close to the peak ion temperature in equilibrium were found.

Keenan et al. (1993b) noted that the OSO-5 flare observation (Mason et al. 1984) of the ratio of the Fe xxiii resonance (132.8 Å) and intercombination (263.8 Å) lines was consistent with a temperature close to the peak ion temperature in equilibrium, $\log T\,(\mathrm{K}) = 7.1$. Del Zanna et al. (2005) used updated atomic data for Fe xxiii (Chidichimo et al. 2005) to measure a temperature of about 10 MK from emissivity ratios involving 2–4 transitions and X-ray SMM BCS observations of a flare. Higher temperatures were obtained by Del Zanna et al. (2005) from the SOLEX observations.

As we have presented in Sect. 4, the ratio of a satellite lines formed by dielectronic capture with the resonance line is an excellent temperature diagnostic, independent of the population of the ions and the electron density.

**Fig. 97:**
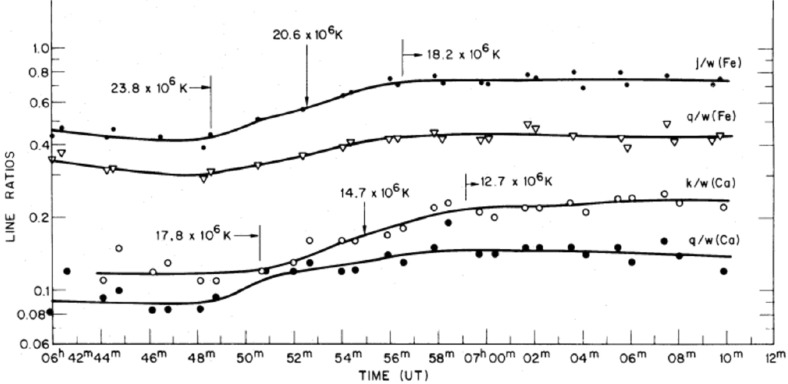
A variation of ratios of lines within the He-like Fe and Ca complexes as observed during a large flare with SOLFLEX Image reproduced with permission from Doschek et al. (1980)

As an example we show the results obtained for one flare observed with SOLFLEX (Doschek et al. 1980) in Fig. 97. The electron temperature as obtained from the ratio of the *j* / *w* lines in the He-like Fe shows a marked increase from about 15 MK to at most 24 MK at the peak emission, then it shows a slow decay. The temperatures obtained from the *k* / *w* ratio in the He-like Ca show a similar behaviour, but are consistently lower by about 5 MK. This has been interpreted as an indication of the presence of multi-thermal plasma during the peak phase.

Similar results have been obtained by the Hinotori, Yohkoh and SMM satellites (see, e.g., Doschek 1990), although the Hinotori satellite also performed observations of the H-like Fe, discovering that a super-hot component was also present in some of the largest flares.

In general, smaller flares reach lower peak temperatures. Indeed, there is a good correlation between the class of a flare, as estimated e.g., by the total X-ray flux from GOES, and the isothermal temperatures at peak estimated from the He-like spectra, as shown by Feldman et al. (1996).

## Line widths and ion temperatures

A significant amount of information on the solar plasma can be obtained from the shape of a spectral line profile. There are many effects that can change this. Only a brief summary is given below, without discussing detailed effects caused by any external electromagnetic fields (e.g., Zeeman effect, Stark effect) or by opacity.

### Natural, collisional, and Doppler broadening

The main effects which shape the profile of a spectral line are natural broadening, collisional broadening and Doppler broadening. The natural broadening is related to the uncertainty principle in quantum mechanics: if we consider a transition with a probability of spontaneous decay $A_{ji}$, the average lifetime of the upper level is 1/$A_{ji}$, so the uncertainty principle is

107 $$\begin{aligned} \varDelta E {1 \over A_{ji}} \simeq h, \end{aligned}$$

where $\varDelta E$ is the broadening in energy.

Collisional (or pressure) broadening is caused by the interaction between the ion and other particles. It can be shown that the line profile $\phi$, normalised in frequency, is a Lorentzian function:

108 $$\begin{aligned} \phi (\nu - \nu _0) = {1 \over \pi }\,{\varGamma \over (\nu - \nu _0)^2 + \varGamma ^2}, \end{aligned}$$

where the damping constant $\varGamma$ is

109 $$\begin{aligned} \varGamma = {A_{ji} \over 4 \pi } + {f \over 2 \pi }, \end{aligned}$$

and *f* is the number of collisions per unit time.

The Doppler broadening is due to the thermal motion of the ions. The shift caused by the Doppler effect in the frequency of the photons emitted is, for non-relativistic velocities, proportional to *v* / *c*:

110 $$\begin{aligned} {v \over c}= {\nu - \nu _0 \over \nu _0}, \end{aligned}$$

where $\nu _0$ is the frequency at rest.

If one assumes a thermal (Maxwellian) distribution for the velocities of the ions, the probability $f(v)\,\mathrm{d}v$ that the ion has a velocity (in the 3-D space) between *v* and $v+\mathrm{d}v$ is:

111 $$\begin{aligned} f(v) = 4\,\pi \,v^2 \left( {M \over 2\,\pi \,kT_{\mathrm{i}}} \right) ^{3/2}\,{\mathrm{e}}^{-{M v^2/2\,kT_{\mathrm{i}}}}, \end{aligned}$$

where *k* indicates Boltzmann’s constant, *M* is the mass of the ion (often written as the product of the atomic mass times the proton mass), and $T_{\mathrm{i}}$ the ion temperature. The normalised profile function is obtained by expressing the Maxwellian function in terms of frequency, and considering $\mathrm{d}v = (c/\nu _0) \mathrm{d}\nu$:

112 $$\begin{aligned} \phi = {1 \over \pi ^{1/2}\,\varDelta \nu _D} {\mathrm{e}}^{-{({\nu - \nu _0 \over \varDelta \nu _D})^2}}, \end{aligned}$$

where

113 $$\begin{aligned} \varDelta \nu _D = {\nu _0 \over c} \left( 2\,k\,T_{\mathrm{i}} \over M \right) ^{1/2} \end{aligned}$$

is the Doppler width of the line in frequency. Note that the constant is such that the profile function is normalised to one: $\int \phi (\nu ) \mathrm{d}\nu =1$.

The sum of the three broadening effects results in a Voigt profile. However, for the solar TR and corona both natural and collisional broadening are negligible, the Voigt profile can be approximated with a Gaussian, and the intensity of a spectral line can be written in terms of wavelength $\lambda$ as

114 $$\begin{aligned} I_\lambda ={I_{\mathrm{tot}} \over \sqrt{2 \pi } \sigma } e^{\left[ - {(\lambda - \lambda _0)^2 \over 2 \sigma ^2}\right] }, \end{aligned}$$

where $I_{\mathrm{tot}}=\int I_\lambda d\lambda$ is the integrated intensity and $\sigma$ is the Gaussian width given by:

115 $$\begin{aligned} \sigma ^2 = {\lambda _0^2 \over 2 c^2} \left( {2 k T_{\mathrm{i}} \over M} \right) . \end{aligned}$$

Normally, the spectral resolution of XUV instruments is such that the instrumental width (assumed as a Gaussian with width $\sigma _I$) is an important additional broadening. In addition, local turbulent motions often provide further broadenings. For an optically thin line the profile is usually assumed Gaussian, but with a Gaussian width given by:

116 $$\begin{aligned} \sigma ^2 = {\lambda _0^2 \over 2 c^2} \left( {2 k T_{\mathrm{i}} \over M} + \xi ^2 \right) + \sigma _I^2\, \end{aligned}$$

where $\xi$ is the non-thermal velocity, i.e., the most probable velocity of the random bulk plasma motions, assuming they are Maxwellian. For example, the thermal broadening (Gaussian width $\sqrt{2 k T_{\mathrm{i}}/M}$) of Si iv is $6.88\,\hbox {km\,s}^{-1}$, assuming $T_{\mathrm{i}} = 80{,}000$ K, being $k= 1.38 \times 10^{-16}$ and $M=28.086 \times 1.66 \times 10^{-24}$ (cgs units). For comparison, the instrumental width of the IRIS instrument is significantly lower, $3.9\,\hbox {km s}^{-1}$.

Note that with the above definition of the Gaussian width $\sigma$, the full-width-half-maximum $\hbox {FWHM}\,=\,2\,\sqrt{(2\,ln 2)} \sigma \simeq 2.35 \sigma$. Also note that in the literature the non-thermal velocity $\xi$ is sometimes obtained from the measured FWHM:

117 $$\begin{aligned} { FWHM}^2 = w_{\mathrm{I}}^2\,+ w_{\mathrm{O}}^2 = w_{\mathrm{I}}^2\,+\,4\,\ln 2\,\left( {\lambda _0 \over c} \right) ^2\,\left( {2 k T_{\mathrm{i}} \over M} + \xi ^2 \right) , \end{aligned}$$

where $w_{\mathrm{I}}$ is the instrumental FWHM and $w_{\mathrm{O}}$ is the observed FWHM once the instrumental width is removed.

The above expression provides an important diagnostic for the solar corona, i.e., the possibility of measuring the ion temperatures, if some additional constraints are adopted. The approach suggested by Tu et al. (1998) is to consider that the ion temperature $T_{\mathrm{i}}$ of a line cannot be larger than the value $T_{\mathrm{max,i}}$ obtained when $\xi =0$:

118 $$\begin{aligned} T_{\max ,i} = {{M c^2}\over {8 k \ln 2}} {\left( {{{w_{\mathrm{O}}} \over {\lambda _0}}}\right) }^2. \end{aligned}$$

Similarly, the excess width cannot be larger than the value obtained when $T_{\mathrm{i}}=0, \xi _{\mathrm{max}}$:

119 $$\begin{aligned} \xi _{\max } = \sqrt{{{c^2}\over {4\ln 2}}{{\left( {{{w_{\mathrm{O}}}\over {\lambda _0}}}\right) }^2}} = 1.80\times 10^{10} {\left( {{{w_{\mathrm{O}}}\over {\lambda _0}}}\right) }. \end{aligned}$$

If we define with $\xi _{\mathrm{m}}$ as the smallest among a set of $\xi _{\mathrm{max}}$ values obtained from several lines, the ion temperature of each ion must be larger than the value obtained when $\xi =\xi _{\mathrm{m}}$. If we indicate with *m* the line for which $xi=\xi _{\mathrm{m}}$, the ion temperatures should be higher than the values

120 $$\begin{aligned} T_{\min ,i} = {{M c^2}\over {8 k \ln 2}} {\left[ {{\left( {{{w_{\mathrm{O}}}\over {\lambda _0}}}\right) }_i^2-{\left( {{{w_{\mathrm{O}}}\over {\lambda _0}}}\right) }_m^2}\right] }, \end{aligned}$$

i.e., we obtain a lower limit for $T_{\mathrm{i}}$ (except for the line *m* where the lower limit is zero).

In addition, broadening due to opacity effects can also be present, especially in low-temperature strong lines in the upper chromosphere/lower transition region. If we assume that the source function $S_\lambda$ is constant along the direction of the observer, the emergent intensity *I* is given by

$$\begin{aligned} I(\lambda ) = \int \limits _{0}^{\tau (\lambda )} S_\lambda \exp {(-t_\lambda )} \mathrm{d}t_\lambda = S_\lambda \left[ 1 - \exp (-\tau (\lambda ))\right] . \end{aligned}$$

For a Gaussian profile

121 $$\begin{aligned} \phi (\lambda )\,=\,{I(\lambda ) \over I_0}\,=\,\mathrm{exp}\left( -\frac{(\lambda -\lambda _0)^2}{2 \sigma ^2} \right) , \end{aligned}$$

where the total intensity is

122 $$\begin{aligned} I_{\mathrm{tot}} = \int \limits _{-\infty }^{+\infty } I(\lambda ) \mathrm{d}\lambda = \sigma I_0 (2\pi )^{1/2}, \end{aligned}$$

we have

123 $$\begin{aligned} {\mathrm{FWHM}(\tau _0)}^2 = 8 \sigma ^2 \left[ \ln (\tau _0) - \ln \left( \ln (2) - \ln (1+ \mathrm{e}^{-\tau _0})\right) \right] , \end{aligned}$$

from which one can estimate how much the line width increases for values of the optical depth at line centre much greater than 1.

### An overview of line broadening measurements

The main observational results are that profiles of transition-region (TR) lines always exhibit non-thermal (also called excess) broadenings of the order of $10{-}40\,\hbox {km s}^{-1}$. The profiles of coronal lines also often exhibit non-thermal broadenings of the order $18\,\hbox {km s}^{-1}$ or larger during flares. The interpretation of these non-thermal broadenings is complex and has not yet been fully resolved.

This excess broadening has particular scientific relevance as it is most probably related to the physical processes that heat or accelerate the plasma. For example, it has long been thought that the excess broadening is a signature of waves that are propagating in the solar atmosphere, which could contribute to the coronal heating and acceleration of the solar wind. The observed non-thermal broadenings in such a case provide severe constraints on the types of waves (see, e.g., Hollweg 1978, 1984; Parker 1988; van Ballegooijen et al. 2011). For example, a decrease in the excess broadening with distance from the Sun has often been reported (see below) and interpreted as caused by the damping of Alfvén waves in the corona. It is in fact thought that convective motions at the footpoints of magnetic flux tubes generate wave-like fluctuations that propagate up into the extended corona (see, e.g., Cranmer and Ballegooijen 2005). The wave turbulence is therefore intimately related to the heating of the solar corona and the acceleration of the solar wind (see, e.g., Ofman 2010; Arregui 2015).

Nanoflare heating could also cause spectral line broadening in the coronal lines (see, e.g., Cargill 1996; Patsourakos and Klimchuk 2006) through turbulent processes.

On the other hand, there is always the possibility that the excess broadenings are due to a superposition of velocity fields with scales smaller than the instrument resolution. In this case, one would expect to observe a decrease in the excess broadening with increased resolution, at least in the transition region (in fact, in the corona the long path lengths and the superposition of structures could limit such a decrease).

In principle, the possibility that the excess broadening in the TR lines is due to random mass flows is not unreasonable. In fact, if the spectroscopic filling factor in these lines is assumed to be a real indication of sub-resolution structures, then HRTS results indicate the presence of scales down to a few km (Dere et al. 1987), i.e., much below the resolution of any previous and current instruments. Recently, IRIS observations at $0.33{^{\prime \prime }}$ (slit width) resolution (about 250 km) have been obtained. De Pontieu et al. (2015) suggests that the non-thermal broadening in Si iv in the quiet Sun is still of the same order (about $20\,\hbox {km}\,\hbox {s}^{-1}$) as that previously observed with earlier instruments, which had a resolution of about $1{^{\prime \prime }}$. Therefore, any velocity fields which could be responsible for the broadenings must have scales smaller than the IRIS resolution, unless they are consistent along the line of sight. However, as we show below, where we present the IRIS results in the context of earlier ones, our analysis shows that IRIS did actually observe lower excess widths.

Finally, other processes could influence the excess widths in the TR lines. For example, De Pontieu et al. (2015) found indications that non-equilibrium ionisation could account for a significant fraction of the observed non-thermal broadening. That is that ions are not formed at the temperature corresponding to peak abundance in the ionisation balance in equilibrium.

In the present section we provide a brief overview of the main observational constraints, organised in different sub-sections, depending on the solar feature observed. Before going into detail, however, it is worth pointing out a few key general issues which can affect the observations and their interpretation. In fact, contradictory results have often been reported in the literature. Variations in the excess widths have often been attributed to a variety of reasons such as the choice of ion temperatures, non-equilibrium effects, curvature of the magnetic field, instrumental effects and solar cycle variations.

On the observational side, we point out that for most instruments, measurements of line widths are subject to large uncertainties, some being systematic. For several instruments, the instrumental line width is a significant fraction of the observed width. Characterising the instrumental line width and its variations is notoriously difficult. Instrumental line widths are normally assumed to be Gaussians but in reality they are not. The instrumental PSF often shows unexplained characteristics as well. Furthermore, several instruments (e.g., SoHO/CDS, Hinode/EIS) were not specifically designed to study line profiles, so the instrumental line width is sampled with only a few pixels across it. Hence, accurate measurements of line profiles are very difficult.

On the issue of interpreting the observed widths, when we say that the profiles exhibit non-thermal broadening we mean that the line width is larger than the one expected from thermal broadening in equilibrium conditions. For the equilibrium temperature of an ion it is often assumed that the ion and electron temperatures are the same, although in reality what one measures is an ion temperature from the line width. This assumption is justified by the fact that the equilibration time for the ion and electron to thermalise is very short. For example, in the transition region, the equilibration time is about 0.04 s at a typical TR density of $10^{10}\,\hbox {cm}^{-3}$. The electron temperatures in turn are normally estimated from the intensity ratios of lines from different ions, assuming that the ion charge state distributions are in equilibrium, or occasionally by more direct measurements of line ratios from the same ion (cf. Sect. 11). The ion temperatures as obtained from the line widths are typically a factor 4–5 higher than the ionisation temperatures, corresponding to peak ion abundance, in the TR.

It would however be difficult to find a mechanism that keeps ion and electron temperatures so different. Bruner and McWhirter (1979) estimate that maintaining such a difference would also require a significant amount of energy. So, a likely explanation would be that the ionisation temperatures are not representing the real electron temperatures, which could actually be much higher. This could be caused by several factors. We have seen in Sect. 3.5 that various effects can change the ion charge state distribution even assuming equilibrium. Non-equilibrium ionization is likely to occur in the TR if one considers the observed dynamics and flows, temperature gradients and temporal variability in the line intensities. On the other hand, we have seen in Sect. 12.2 that the few measurements of direct electron temperatures in the TR do not suggest significant differences with the ionisation temperatures calculated assuming equilibrium, in most cases. However, there are a few cases where differences were reported. The situation is therefore unclear. More accurate observations and modelling of plasma processes, taking into account of non-equilibrium and time dependent processes, are needed to resolve these issues.

Another approach often adopted in the literature is to assume that the excess widths of lines formed at similar temperatures should be the same, and the ion kinetic temperature is independent of the mass and charge of the ions. The first assumption is reasonable, as one would expect that lines formed at similar temperatures should experience, within the same volume, the same processes. The second assumption is based on the idea that the ions are in thermal equilibrium between themselves. For example, Seely et al. (1997) made these two assumptions and determined the turbulent velocity from the observed widths by assuming that the ion temperature was equal to the electron temperature.

More generally, if ions of different masses are observed, one could relax the second assumption and obtain limits on the ion temperatures from the measured excess widths (see, e.g., Tu et al. 1998, and below). Tu et al. (1998) applied this method to polar coronal hole observations by SUMER, finding ion temperatures significantly higher than electron temperatures. Other authors have applied the same method to observations of different regions. For example, Landi (2007) used SUMER observations and this method to find that the ion temperatures were close to the electron temperatures, during solar minimum conditions. On the other hand, Landi and Cranmer (2009) found in coronal holes ion temperatures significantly higher than electron temperatures.

### TR lines in the quiet Sun

The profiles of transition region lines are typically much broader than their thermal width in the quiet Sun. Observations have been obtained with e.g., the Skylark rocket (Boland et al. 1973, 1975), the Skylab NRL slit spectrometer (see, e.g., Doschek et al. 1976; Kjeldseth Moe and Nicolas 1977; Mariska et al. 1978, 1979), OSO 8 (see, e.g., Shine et al. 1976), the HRTS rocket (see, e.g., Dere et al. 1987; Dere and Mason 1993), SoHO SUMER (see, e.g., Chae et al. 1998; Teriaca et al. 1999; Peter 2001) at about $1{-}2{^{\prime \prime }}$ resolution, and more recently with the IRIS satellite at $0.3{^{\prime \prime }}$ resolution.

It should be noted that variations in the measured line widths are present in the literature. They are partly due to real spatial variations, but also to many other effects, such as the differences in the instrumental broadening, the spatial resolution, the exposure times, opacity effects, etc. We note that considering its spatial resolution, sensitivity and instrumental broadening, the best instrument, until the launch of IRIS, was probably HRTS. The FWHM of the HRTS instrument was about 0.07 Å (Dere and Mason 1993), i.e., similar to the value of the Skylab NRL slit spectrometer (about 0.06 Å, cf. Doschek et al. 1976) which however did not have a stigmatic slit. The FWHM of the SUMER instrument was about 2.3 pixels, equal to about 0.1 Å (see, e.g., Chae et al. 1998), while the spatial resolution was about 2$''$. The second Skylark rocket had an excellent FWHM resolution of 0.03 Å (Boland et al. 1975), but only measured the line widths of a few lines, mostly at longer wavelengths (compared to the other instruments). The excellent Skylab NRL slit spectrometer observations were performed near the limb, where some additional opacity effect were probably present.

The sensitivity of the HRTS instrument was such that exposures of only 1s could be used to record the strongest lines. Dere and Mason (1993) analysis used a combination of the 1, 2.8, 8, and 20 s exposures. On the other hand, typical quiet Sun SUMER exposures were much longer. For example, Peter (2001) used the reference spectra obtained with 115 s exposures, while Chae et al. (1998) used data with 400–500 s exposures. Some additional broadenings of the lines is clearly expected for longer exposures, given the dynamical nature of the TR lines. Indeed Chae et al. (1998) found averaged differences of about $4\,\hbox {km s}^{-1}$ between the 20 and 300 s exposures.

**Fig. 98:**
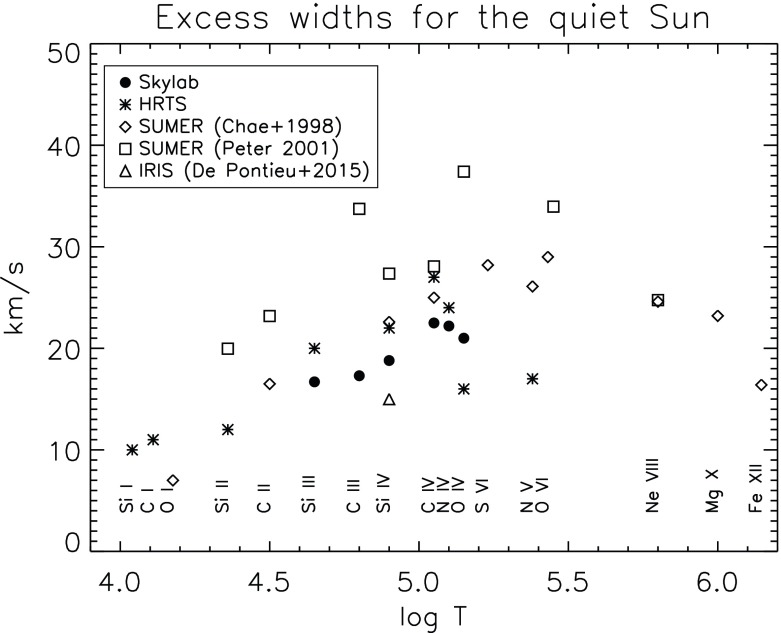
Nonthermal line widths as a function of temperature for the quiet Sun, as obtained from the Skylab NRL slit spectrometer (Feldman et al. 1988), from HRTS by Dere and Mason (1993), SoHO SUMER by Chae et al. (1998) and Peter (2001), and IRIS by De Pontieu et al. (2015)

The average excess broadening varies with the temperature of formation of the line, with a peak in the middle of the transition region around $2 \times 10^5\,\hbox {K}$ of about $25\,\hbox {km s}^{-1}$ for the quiet Sun. An example is shown in Fig. 98, with measurements from the Skylab NRL slit spectrometer (see the review by Feldman et al. 1988), HRTS (Dere and Mason 1993), SoHO SUMER (Chae et al. 1998; Peter 2001), and the last IRIS measurement from De Pontieu et al. (2015). We note that the Skylab NRL slit spectrometer results reported by Doschek et al. (1976) and Mariska et al. (1978) are consistent. We also note that there are several of such plots in the literature, however the actual formation temperature of a line is a non-trivial issue. Due to the emission measure structure in the transition region, the formation temperature of an ion can in fact be quite different from the value at peak ion abundance in ionization equilibrium, which would result in quite different plots.

The data points in Fig. 98 are plotted at an approximate effective temperature of formation of the lines, and not at the temperature of peak ion abundance in ionization equilibrium. This was obtained by us from average quiet Sun radiances obtained from HRTS spectra. The actual formation temperatures within each observation could be slightly different, but the trends would remain. The observed line widths reported by Peter (2001) have been converted into excess widths adopting the temperature of peak ion abundance as reported by Peter (2001), for consistency.

Another issue to keep in mind when interpreting line width observations is that lines formed at lower temperatures such as the dipole-allowed transitions of O i, Si ii, C ii, Si iii are often optically thick, which tends to increase the width of a line. In fact, the widths of different lines from the same ion often differ.

**Fig. 99:**
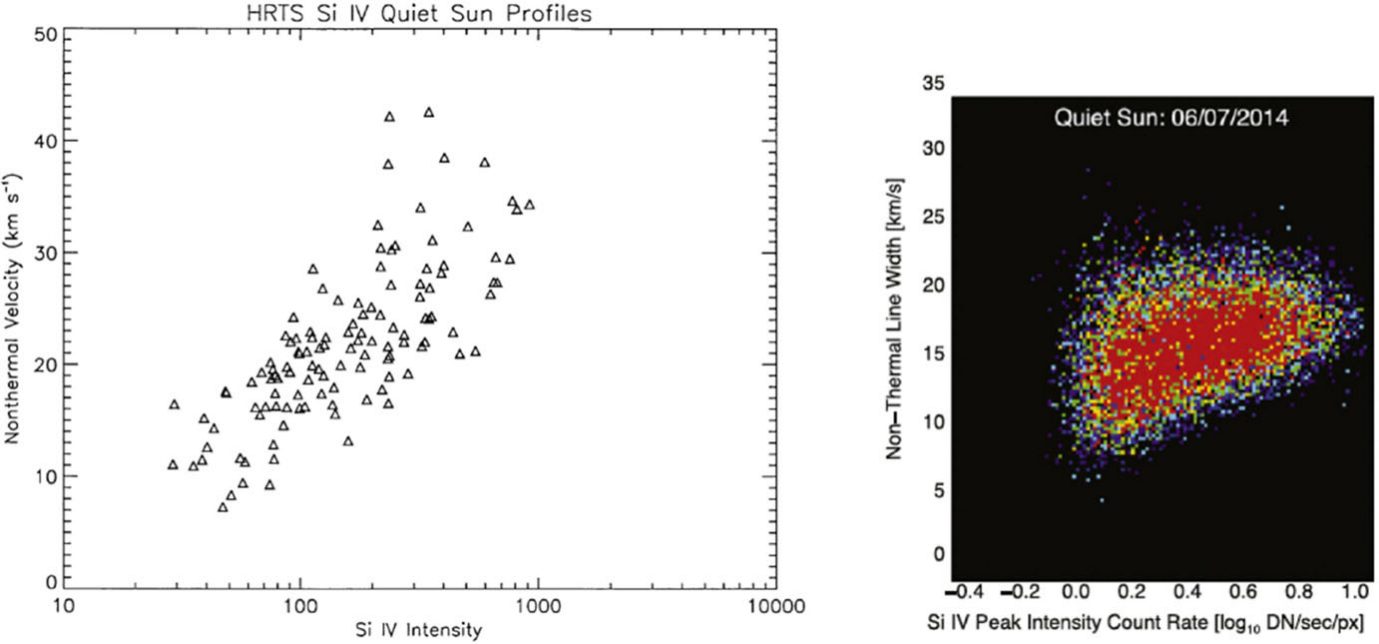
Nonthermal line widths of the Si iv 1403 Å line as a function of its intensity for the quiet Sun, as obtained from HRTS by Dere and Mason (1993) on the left, and IRIS (De Pontieu et al. 2015), on the right. Note the different scales

Also, we stress that the values often found in the literature are average values. For example, the quiet Sun excess broadening in Si iv observed with HRTS varied from about 10 to $40\,\hbox {km s}^{-1}$, when the radiance changed by over an order of magnitude (see Dere and Mason 1993 and Fig. 99).

We recall that IRIS observations at $0.33{^{\prime \prime }}$ (slit width) resolution and short exposure times (seconds) have shown (De Pontieu et al. 2015) that the non-thermal broadening in Si iv in the quiet Sun is still of the same order, although we note that values are overall smaller ($10{-}20\,\hbox {km}\,\hbox {s}^{-1}$) than those measured by HRTS, as Fig. 99 shows. So, we find that the higher IRIS spatial resolution does indeed produce somewhat lower non-thermal broadenings, in contrast to the conclusions by De Pontieu et al. (2015).

It is in fact well established that the excess broadening is correlated with the intensity of the lines, for the lines formed in the middle of the transition region such as Si iv. There is very little excess broadening in the supergranular cell centres, while the largest values are at the cell boundaries. Interestingly, the network boundaries are typically the places where all sorts of explosive events occur (where widths are much larger). Lines formed at lower and higher temperature have a much weaker correlation between intensities and excess widths.

Coronal holes on average have very similar characteristics. Active regions have similar, or just a bit larger, excess broadening than the quiet Sun.

Interestingly, observations of several quiescent prominences with the Skylab NRL slit spectrometer have shown that in several (but not all) cases the excess broadening was almost non-existent, of the order of just a few $\hbox {km s}^{-1}$ (Feldman and Doschek 1977a).

**Fig. 100:**
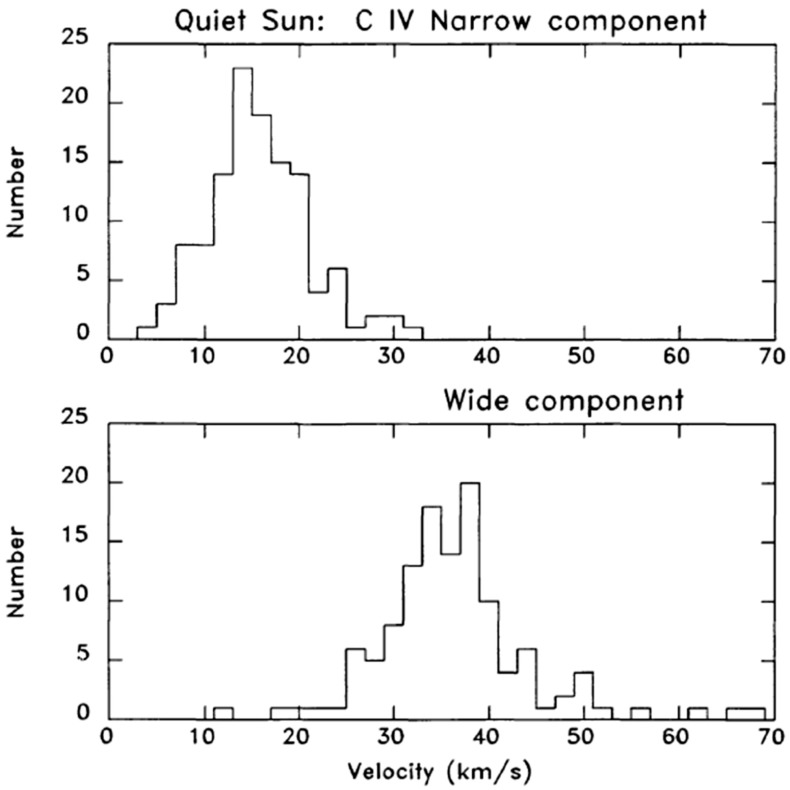
Distribution of the nonthermal line widths of the core and broad component of the C iv line for the quiet Sun, as obtained from HRTS by Dere and Mason (1993)

Earlier observations showed nearly Gaussian line profiles, although distortions of the profiles are very common, especially in TR lines, due to the intrinsic transient nature of the emission and the ubiquity of explosive events, when very large red- and blue-wing asymmetries are observed. In the more quiescent observations, it was however found, especially from HRTS, that the core of the lines was nearly Gaussian, but a much broader and weaker component, of the order of $50\,\hbox {km s}^{-1}$ was also present. Figure 100 shows the distributions of the nonthermal line widths of the core and broad component of one of the C iv lines for the quiet Sun, as obtained from HRTS by Dere and Mason (1993).

**Fig. 101:**
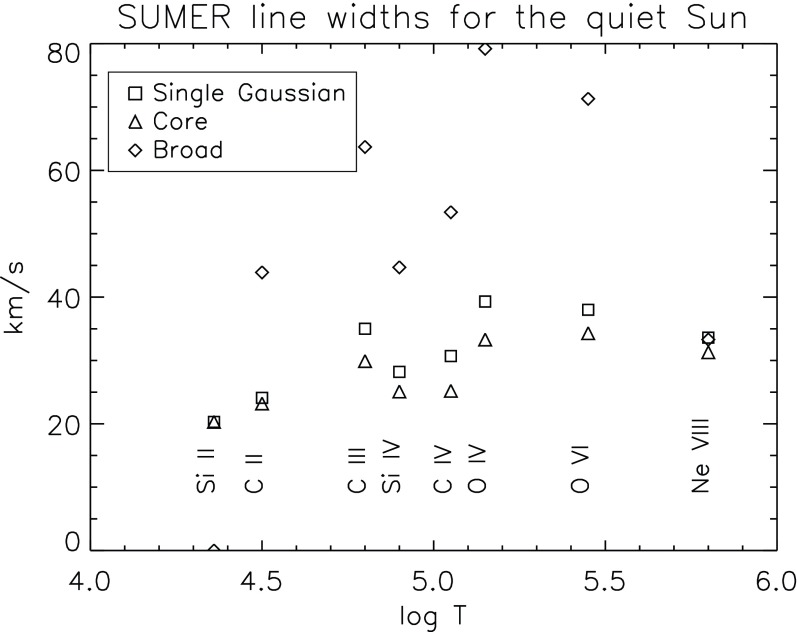
Observed line widths of the core and broad components in the quiet Sun from SoHO SUMER (Peter 2001)

Traditionally, the profiles of TR lines have therefore been fitted with two Gaussian profiles. Figure 101 shows the variation of the core and broad components in TR lines as measured with SoHO SUMER (see, e.g., Peter 2001). It is interesting to note that the contribution of the broad component changes with the temperature of formation of the line. The different behaviour of the narrow and broad components led to the interpretation that they originate in two different magnetic structures: the narrow one in closed magnetic loops and the broad one in open coronal funnels (Peter 2001).

An alternative possibility is that TR line profiles reflect a non-thermal distribution of the velocities of the ions. Dudík et al. (2017b) showed that IRIS line profiles in an active region are better fitted with a $\kappa$-distribution rather than a double Gaussian, although an extra non-thermal broadening is still needed to explain the profiles.

Another interesting fact is the lack of a clear center-to-limb variation in the average values of the excess widths in TR lines, which indicates that if the interpretation is in terms of Doppler motions, they are nearly isotropic. Some center to limb changes in the broadening have been observed, but were mostly attributed to optical depth effects.

Interestingly, broadening in cooler lines (e.g., C ii, Si iii, up to C iv) is observed to increase with height when observations at the limb are performed (see,e.g., Doschek et al. 1977a; Nicolas et al. 1977; Mariska et al. 1978, 1979). For example, the excess velocity in the resonance lines of C iv $12{^{\prime \prime }}$ above the visible limb was found to be about $33\,\hbox {km s}^{-1}$, i.e., twice that which was observed on-disk (Dere et al. 1987).

Systematic differences in the excess line widths of allowed and intersystem TR lines have been noted from Skylab and HRTS observations (Brueckner et al. 1977; Kjeldseth Moe and Nicolas 1977; Dere and Mason 1993). The widths of the allowed oxygen lines appear to be about 50% larger than those of the intersystem lines. This was also noted by Doschek and Feldman (2004) using SoHO SUMER observations of the quiet Sun. In principle, the larger widths of the allowed lines could be due to opacity effects. However, center-to-limb measurements of TR lines normally indicate very small opacities in these lines (see, e.g., Doyle and McWhirter 1980). Estimates of the opacities based on oscillator strengths confirm this (Doschek and Feldman 2004). Doschek and Feldman (2004) also discussed various other possible causes for the differences in the line widths, without finding an explanation. The allowed lines are more sensitive to higher densities and temperatures, so the steep gradients would cause some effects. Standard DEM results have been used to show that this effect is too small. If the plasma had high-density regions, the intersystem lines would be depressed, compared to the allowed ones. If the same plasma was more turbulent, the net effect would be to increase and broaden the allowed lines, compared to the intersystem lines. However, it is not very clear if such high densities components exist (see, e.g., Polito et al. 2016a).

Dere and Mason (1993) suggested, however, that the broad wings in the intersystem lines are not observed because the lines are weak in the spectra. Indeed recent IRIS spectra of the same lines in active regions do indicate that allowed lines such as Si iv and intersystem lines from O iv have very similar profiles and widths (see, e.g., Polito et al. 2016a; Dudík et al. 2017b). We note that IRIS, with its very small instrumental width (one or two pixels) and its good sampling of line profiles, is an excellent instrument to measure line profiles. Recently, Doschek et al. (2016) reviewed earlier Skylab observations, and recalled the high-density interpretation, but when comparing IRIS line widths also found (cf. their Fig. 8) similar line profiles for the O iv and Si iv lines.

### Coronal lines

Early observations of the green and red forbidden visible lines showed excess line widths ($18{-}20\,\hbox {km s}^{-1}$) (see, e.g., Billings and Lehman 1962). Rocket observations of the full-Sun EUV spectrum also showed significant excess broadening in coronal lines, of the order of $30\,\hbox {km s}^{-1}$ (Feldman and Behring 1974). Skylab observations above the limb (around $10{-}20{^{\prime \prime }}$) with the NRL slit spectrograph of the coronal forbidden lines also showed excess line widths of the order of $20\,\hbox {km s}^{-1}$ (see, e.g., Doschek and Feldman 1977b; Cheng et al. 1979). The excess line widths above active regions were somewhat smaller.

The SMM-UVSP measurements indicated somewhat lower values of $15\,\hbox {km s}^{-1}$ or less in the weak forbidden coronal lines from Si viii, Fe x, Fe xi, and Fe xii (Mason 1990).

The excess widths of the coronal lines increase as a function of height above the limb. Hassler et al. (1990) used a rocket spectrum to show this behaviour in the Mg x lines, with broadenings up to $30\,\hbox {km s}^{-1}$.

SoHO CDS off-limb observations of the quiet corona showed a decrease in the line widths of Mg x at larger heights (Harrison et al. 2002). However, it turned out that there was an instrumental variation that was not taken into account (Wilhelm et al. 2005). With a correction obtained from on-disk observations, the widths were found to be constant.

SoHO SUMER off-limb observations of quiet regions have shown little variations in the excess line widths up to 1.45 $R_\odot$ (see, e.g., Doyle et al. 1998; Doschek and Feldman 2000; Wilhelm et al. 2004b). There is, however, a scatter of values, which ultimately depend on the assumed ion temperatures. For example, Seely et al. (1997) reported $10\,\hbox {km s}^{-1}$ at $100{-}200{^{\prime \prime }}$ above the limb, i.e., a value smaller than that reported by Hassler et al. (1990). Landi and Feldman (2003) found instead relatively constant excess widths of $25{-}30\,\hbox {km s}^{-1}$ up to 1.3 $R_\odot$.

Interestingly, changes in the solar cycle appear to affect the excess line widths: Landi (2007), assuming that all the excess line widths for a range of ions were the same, found that the ion temperatures were closer to the electron temperatures during solar minimum conditions, while differences increased with the solar activity.

SoHO LASCO C1 coronagraph observations showed that the width of the Fe xiv green line during the 1996 solar minimum was roughly constant up to 1.3 $R_\odot$ and then decreased (Mierla et al. 2008).

Hinode/EIS observations of the quiet Sun have indicated a small decrease with height (see, e.g., Hahn and Savin 2014).

#### Coronal lines in active regions

Ground-based observations with the coronagraph at the Norikura Solar Observatory (Japan) over the years have shown a range of excess broadenings ($14{-}26\,\hbox {km s}^{-1}$) and some variations along loop structures (Hara and Ichimoto 1999) or with height above the solar limb (see, e.g., Singh et al. 2006, and references therein). In their review, Singh et al. (2006) report that the broadening of the red Fe x line tends to increase up to about $250{^{\prime \prime }}$ above the limb, but then remains unchanged. On the other hand, the width of the green Fe xiv line decreases up to about $300{^{\prime \prime }}$, and then remains unchanged. The widths of the Fe xi, Fe xiii lines show an intermediate behaviour. The difference in the behaviour of the lines is puzzling. We should point out, however, that such observations were carried out above active regions and not in quiet Sun areas. Therefore, the various lines show emission that is not cospatial, and in fact it is likely to have quite different characteristics (see, e.g., Del Zanna and Mason 2003).

Off-limb observations of the coronal forbidden lines with the Skylab NRL instrument showed excess line widths of about $10{-}25\,\hbox {km\,s}^{-1}$ in active regions that were either similar or smaller than those of quiet Sun regions (see, e.g., Cheng et al. 1979).

Regarding on-disk observations of active regions, SMM–XRP FCS observations of X-ray lines (Mg XI, Ne IX, O VIII) formed around 4 MK indicated much higher values, of the order of $40{-}60\,\hbox {km s}^{-1}$ (Acton et al. 1981; Saba and Strong 1991b) in the cores of active regions. We recall that the FCS instrument had an aperture of about 14$''$, i.e., had some spatial resolution. The broadest line profiles were found along the magnetic neutral line. It is interesting to note that these excess widths did not significantly change with the location of the active regions, i.e., no centre-to-limb effects were observed (Saba and Strong 1991a), which suggests that the widths are not due to a superposition of up- and down-flowing motions. The widths of these X-ray lines are significantly larger than most of the reported widths of lines formed at similar temperatures observed in the EUV, UV or visible. This is puzzling and it is not easy to explain. Perhaps such large widths are associated with the increases (up to about $40{-}60\,\hbox {km s}^{-1}$) which have been recently observed by Hinode EIS in active regions before the occurrence of large flares (Harra et al. 2009), although in this case the widths were observed in Fe xii, which is formed at a lower temperature, around 1.5 MK.

Further on-disk observations of widths of coronal lines formed around 1–3 MK in active region loops have been provided by Hinode EIS. Doschek et al. (2007a) found that the excess width in Fe xii is somewhat smaller (by $6\,\hbox {km s}^{-1}$) in coronal loops than in the surrounding regions.

Imada et al. (2009) analysed Hinode EIS observations of an active region quiescent core observed off the limb. By assuming that lines from different ions have the same excess widths, they obtained an ion temperature of 2.5 MK and an excess width of about $13\,\hbox {km s}^{-1}$.

Brooks and Warren (2016) performed a statistical study of excess widths in coronal lines formed in the 1–4 MK range, using observations with Hinode EIS of quiescent AR cores. They found values around $18\,\hbox {km s}^{-1}$, in agreement with previous results, and no significant trends with the temperature of formation of the line. The authors conclude that these measurements are inconsistent with all the current models.

#### Excess widths in active region coronal outflows

Excess widths in lines formed around 1–3 MK have been discovered from Hinode EIS in the so called coronal outflow regions (see, e.g., Del Zanna 2008a, c; Doschek et al. 2008; Harra et al. 2008). The non-thermal broadening and the blue-shifts are small ($10{-}20\,\hbox {km s}^{-1}$), hence were not previously clearly observed. Del Zanna (2008c) showed that only lines formed at temperatures higher than 1 MK present this behaviour, and that higher-temperature lines had stronger blue-shifts. Del Zanna (2008c) and Del Zanna et al. (2011a) indicated that the line profiles were mostly symmetric, as in the case of plage (AR moss) areas (see, e.g., Tripathi and Klimchuk 2013).

**Fig. 102:**
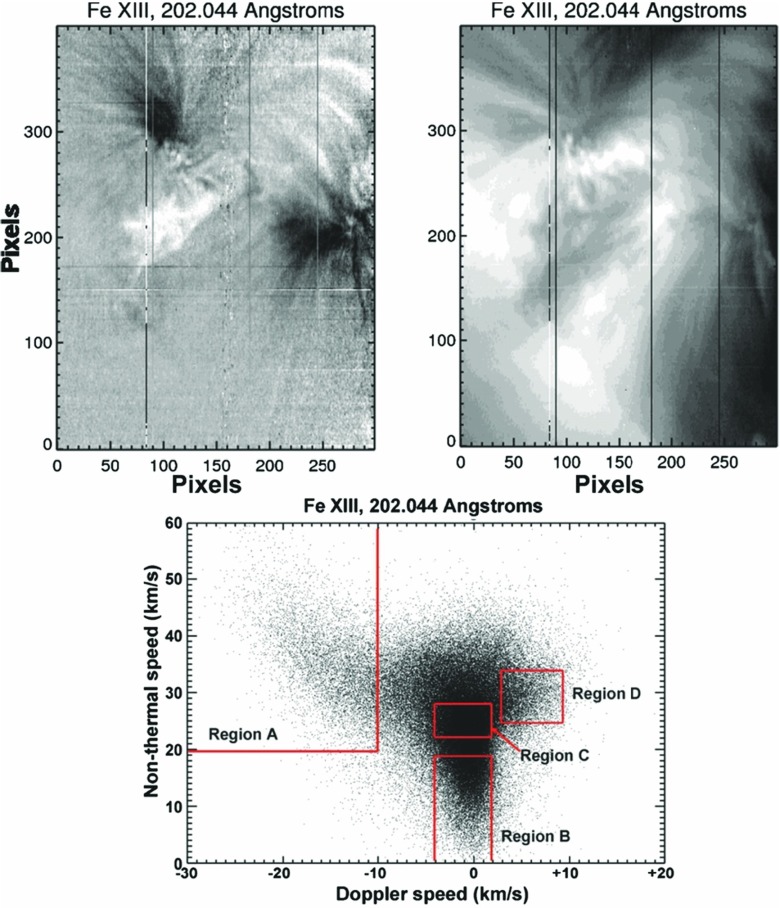
Nonthermal line widths versus blue-shifts in a Hinode EIS Fe xiii line (bottom panel) in an active region. The values were obtained with single Gaussian fitting. The values of the Doppler flows and the radiance in the line are shown in the upper left and right plots, respectively. The largest non-thermal widths are in region ‘A’, which corresponds mainly with the dark (blue-shifted) regions in the Doppler map Image reproduced with permission from Doschek (2012), copyright by AAS

On the other hand, asymmetric line profiles in some of the coronal lines and some outflow regions have been reported by several authors (see, e.g., Hara et al. 2008; De Pontieu et al. 2009; Peter 2010; Bryans et al. 2010), with large blue-shifts reaching values of the order of $100\,\hbox {km s}^{-1}$. The large blue-shifts were found to be correlated with high-speed chromospheric jets or type II spicules by De Pontieu et al. (2009), and were interpreted as the signatures of coronal heating in the chromosphere. However, such observations and subsequent interpretations have been strongly debated in the literature (see, e.g., Reale 2014), and more evidence was later provided to show that indeed line profiles are symmetric in most places (Brooks and Warren 2012b; Doschek 2012). There is also a growing consensus that these excess widths are the result of a superposition, along the line of sight, of upflows with different velocities. Figure 102 (bottom panel, region A, from Doschek 2012) shows the clear correlation that exist between nonthermal line widths and blue-shifts, for one Hinode EIS coronal line from Fe xiii (other lines formed in the 2–3 MK range show similar characteristics).

The coronal outflow regions are rooted in the centre of sunspots and in the middle of plage regions but then have a significant expansion in the corona (Del Zanna 2008a, c). These basic observational results have later been confirmed by a number of authors. A physical explanation for the excess widths and outflows was put forward by Del Zanna et al. (2011a): interchange reconnection between active region core loops and the surrounding unipolar regions occurs in the outer corona and drives a pressure imbalance (a rarefaction wave) which later produces effects observed in the lower corona. Hydrodynamical modelling of the rarefaction wave gave outflow velocities close to the observed ones, increasing with the temperature of formation of the line (Bradshaw et al. 2011). The process continues for a long time, but is intrinsically intermittent. The temporal and spatial (along the line of sight) superposition of different rarefaction waves would explain the broadening.

#### Coronal lines in coronal holes

Coronal holes are the places where one would expect broadening of coronal lines caused by the Alfvén waves which are expected to be present, both on a theoretical and observational basis. In fact, counter-propagating Alfvén waves are expected to be present in the outer corona and with non-linear processes (turbulent dissipation) could provide energy to the solar wind (see, e.g., Parker 1965; Hollweg 1986; Velli 1993; Verdini et al. 2010; van Ballegooijen and Asgari-Targhi 2016, but note that there is an extensive literature on this topic). The fast solar wind, streaming from coronal holes, is strongly Alfvénic as observed in-situ (see, e.g., Cranmer et al. 2007). Pure Alfvén waves are transverse, i.e., the oscillations are perpendicular to the direction of the magnetic field, which is mostly radial in the polar coronal holes. Therefore, outward propagating Alfvén waves would cause a broadening of the spectral lines above polar coronal holes, as indeed observed. The non-thermal widths of the lines in the coronal holes are larger than in equatorial regions. If the excess broadening decreases with height, then it would be a signature of damping of the Alfvén waves, which would resolve a long-standing puzzle about the heating of the fast solar wind.

SoHO SUMER off-limb observations of coronal hole regions have shown that excess line widths sometimes increase, with a tendency to flatten off (see, e.g., Tu et al. 1998; Banerjee et al. 1998; Doyle et al. 1999; Doschek et al. 2001; Wilhelm et al. 2004b). Large uncertainties are often present in the data.

Hinode/EIS observations of coronal holes have shown an increase in the excess line widths, then some decrease after 1.15–1.2 $R_\odot$ above the limb (see, e.g., Banerjee et al. 2009; Hahn et al. 2012; Bemporad and Abbo 2012; Hahn and Savin 2013).

Similar results have been obtained from ground-based observations. Those taken at the National Solar Observatory (USA) of coronal hole regions off-limb show large non-thermal broadenings increasing with height and ranging from $40\,\mathrm{to}\,60\,\hbox {km s}^{-1}$ up to 1.16 $R_\odot$ in the red line from Fe x (Hassler and Moran 1994). Ground-based observations with the coronagraph at the Norikura Solar Observatory (Japan) above coronal holes show the same behaviour in the iron forbidden lines: the width of the red Fe x line increases with height, while that of the green Fe xiv line decreases (see, e.g., Prasad et al. 2013).

### Flare lines

It is well established that flare lines, i.e., lines formed at 10 MK or more, show significant excess widths during the impulsive phase of flares, in particular a few minutes before the onset of the HXR bursts and also show blue-shifted components, which are signatures of the chromospheric evaporation. These excess widths were observed with, e.g., the NRL SOLFLEX instrument (see, e.g., Doschek et al. 1979) and the SMM BCS (see, e.g., Antonucci et al. 1982; Antonucci and Dodero 1995). One of the limitations of such measurements was the limited spatial information about the location of the non-thermal broadenings. With SMM/UVSP, which had much better spatial resolution than the X-ray instruments, excess widths and asymmetric line profiles (with blue-shifts of the order of $200\,\hbox {km s}^{-1}$) were observed during the early stages of flares at the footpoint regions in the Fe xxi flare line (Mason et al. 1986).

**Fig. 103:**
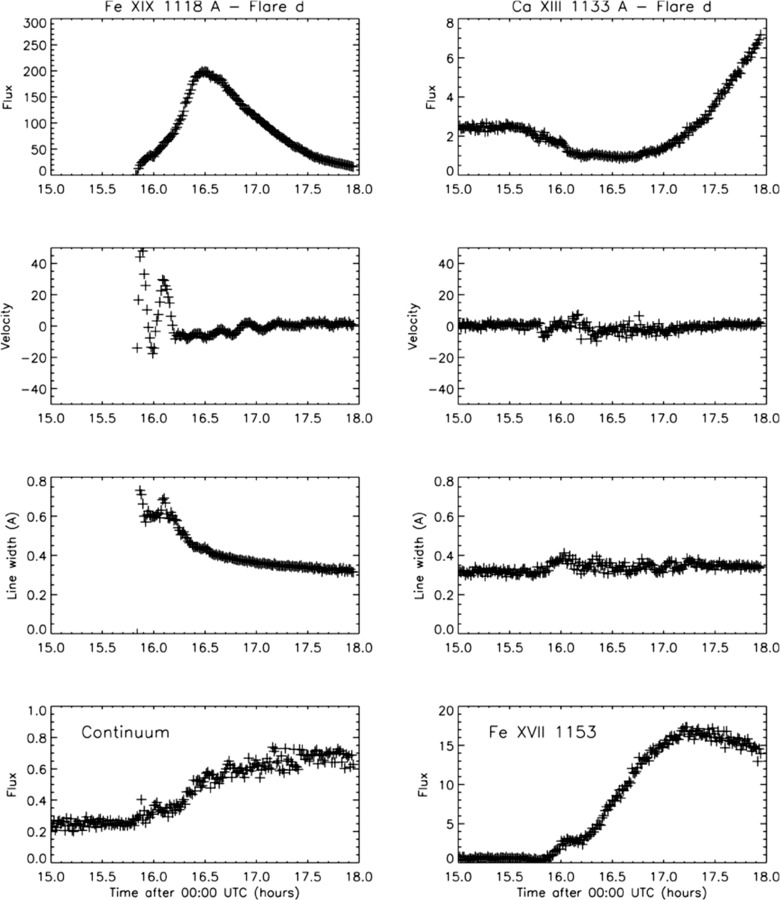
Nonthermal line widths in the Fe xix SUMER flare line (third plot form the top, left column) Image reproduced with permission from Feldman et al. (2004), copyright by AAS

Several spatially-resolved observations of flares were obtained with SoHO SUMER in flare lines while the slit was positioned off the limb, normally in a sit-and-stare mode. Most studies focused on extremely interesting Doppler flows and damped oscillations observed in the flare lines. However, a few published studies also presented measurements of the excess widths as a function of time. For example, Kliem et al. (2002) reported a significant excess width of almost $100\,\hbox {km s}^{-1}$ in the Fe xxi flare line during the peak phase of a flare. Similar results were obtained by Landi et al. (2003) from SUMER observations of an M7 class flare. The widths during the initial phases were difficult to estimate because the line profiles were broadened by large ($600\,\hbox {km s}^{-1}$) systematic bulk motions, as described by Innes et al. (2001). Feldman et al. (2004) measured excess widths during the initial phases of several flares, in the Fe xix SUMER flare line at 1118 Å. At flare onset, nonthermal mass motions were found to be between 50 and $100\,\hbox {km s}^{-1}$, as shown in Fig. 103.

SoHO/CDS allowed spatially-resolved measurements of non-thermal broadenings on-disk in the Fe xix flare line. It was a challenge to find an observation where the Fe xix flare line was visible during the impulsive phase of a flare, when the CDS slit was at the right time and right place. One such observation is the M1-class flare described in Del Zanna et al. (2006a). During the impulsive phase of the flare, large excess widths (and totally blueshifted line profile) were observed in the Fe xix line, at the kernels of chromospheric evaporation. The line intensity was very weak, at the limit of detection. The authors also showed that the line width, as well as the blue-shift, decreased over time and quickly disappeared during the peak phase. Similar (but progressively smaller) blueshifted line profiles were found in lines formed at lower temperatures (1–3 MK). On the other hand, the bright post-flare loops that were formed during the impulsive phase did not show any appreciable non-thermal broadenings. Large non-thermal widths and a blue-shifted Fe xix line profile were also found in a sit-and-stare CDS observation by Brosius (2003) during the impulsive phase of a flare. In contrast, most other CDS studies found that the Fe xix line profile was asymmetric, with an enhancement in the blue wing (Teriaca et al. 2003; Milligan et al. 2006).

The hydrodynamic response of the atmosphere during chromospheric evaporation has been studied for a long time with 1-D modelling, which usually predicts that line profiles should be totally blueshifted with increasing velocities in lines formed at higher temperatures, as seen in the CDS observations reported by Del Zanna et al. (2006a).

As we explain below, there is now convincing evidence that the flare lines are broad but blueshifted. However at the time the vast majority of the community thought that the asymmetric line widths were real. A possible explanation for this was that the asymmetric excess widths were composed of a superposition of multi-thread loops activated at different times (see, e.g., Doschek and Warren 2005).

As in the CDS case, most literature based on Hinode EIS observations of the flare Fe xxiii and Fe xxiv lines reported asymmetric line profiles, with strong blue-shifted components (see, e.g., Milligan and Dennis 2009; Young et al. 2013). As in the CDS case, the observations are challenging because the EIS slit had to be at the right place at the right time and the flare lines are very weak during the impulsive phase. Furthermore, these EIS flare lines are all blended to some degree, as discussed e.g., in Del Zanna (2008b). Del Zanna et al. (2011b) however reported, as in the CDS case, weak but totally blueshifted and broad Fe xxiii emission in kernels during the impulsive phase of a small B-class flare, which behaved like a textbook standard model case. Similarly, Hinode EIS sit-and-stare Fe xxiii observations reported by Brosius (2013) of a C1-class flare also showed large widths and blue-shifts, although at some times two components were observed.

As an aside, Del Zanna et al. (2011b) clearly showed that lower-temperature lines formed around 3 MK appear to be broader, but in reality their line profiles are composed of a superposition of a stationary component which is produced by foreground emission (which is always present in active region at such temperatures), and a blue-shifted component which only appears during the impulsive phase in the kernels of chromospheric evaporation. This is an example of the complexity and richness of information which is present in any line profile.

**Fig. 104:**
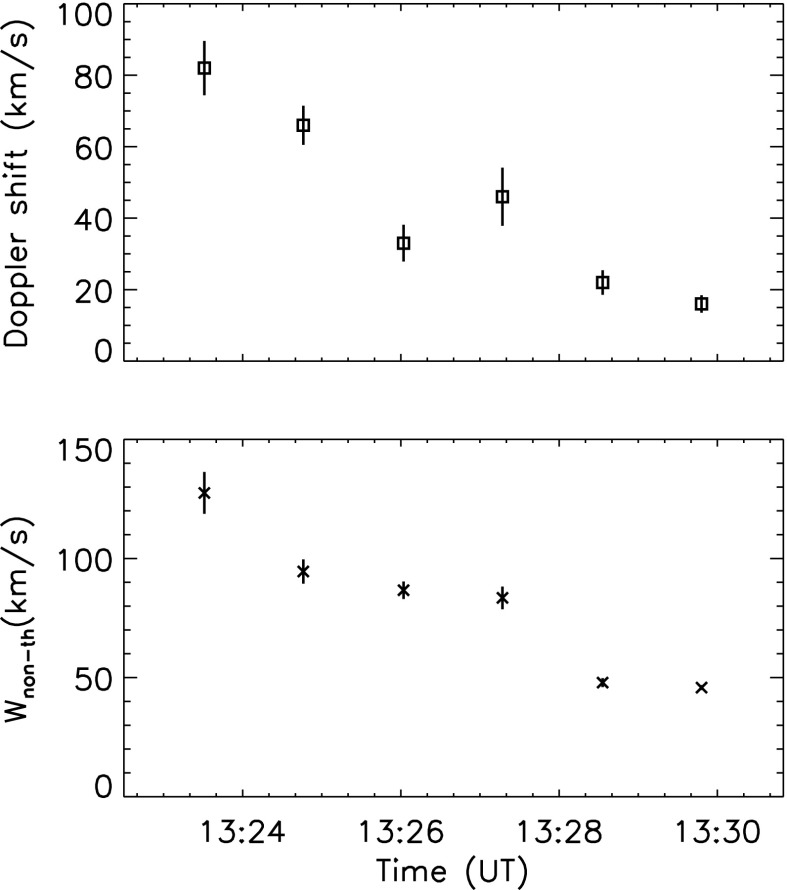
Nonthermal line widths and blue-shifts in the IRIS Fe xxi line as a function of time during the impulsive phase of a flare, at one kernel location in a ribbon Adapted from Polito et al. (2015)

With the launch of IRIS, new observations of the Fe xxi flare line at high spatial resolution and high cadence have been possible. In all cases the line was found to have strong non-thermal broadening and a totally blueshifted line profile during the impulsive phase at the kernels of chromospheric evaporation (see, e.g., Polito et al. 2015; Young et al. 2015; Tian et al. 2015), as in the Fe xix CDS observations reported by Del Zanna et al. (2006a). One example from Polito et al. (2015) is shown in Fig. 104. There is a remarkable agreement between the decrease of the excess width and the blue-shift in the line profile. This confirms earlier work with SoHO/CDS and the Fe xix line by Del Zanna et al. (2006a), although CDS had a much lower temporal, spectral and spatial resolution.

The above differences are puzzling. It is possible that different sub-resolution strands are activated in different ways, as suggested by the observations reported by Brosius (2013). On the other hand, the large differences in spatial resolution between EIS and IRIS could cause such differences, as shown by Polito et al. (2016b). Polito et al. (2016b) found a rare occurrence, a flare simultaneously observed at the same place by Hinode EIS and IRIS. The EIS flare lines showed asymmetric line profiles while the IRIS Fe xxi was totally blueshifted. However, the IRIS data showed that within the EIS effective spatial resolution (3–4$''$), both a stationary and a blueshifted component were present. This provides a simple explanation as to why the profiles of EIS flare lines are often asymmetric.

## Measuring chemical abundances from XUV spectroscopy

### Introduction

Pottasch (1963) used full-Sun EUV observations and developed an approximate emission measure method (described below) to investigate the abundances of elements. He found that the abundances of elements such as magnesium and iron were greatly enhanced compared to their photospheric values. These early results had large uncertainties (about a factor of 2), mainly because of the use of approximate methods to estimate the cross-sections for electron impact excitation.

During the 1980s, it became well established (cf. Meyer 1985) that the coronal abundances were variable and different from the photospheric values. Patterns in the variations also emerged, the most famous one being the correlation between the abundance of an element and its first ionization potential (FIP). The low-FIP ($\le 10\,\hbox {eV}$) elements are more abundant than the high-FIP ones, relative to the photospheric values (the FIP bias).

Support for the presence of a FIP bias later came from the in-situ measurements of the chemical composition of the solar wind, where it was clear that the fast wind streaming from the coronal holes has a near-photospheric composition, while the slow wind has a variable composition, on average showing a FIP bias (see, e.g., von Steiger et al. 2000).

There is an extended literature on measurements of solar chemical abundances, and only a few key issues are discussed here. We point out that over the years several review articles have been written, see e.g., Meyer (1985), Feldman (1992a), Mason (1995), Bochsler (2007), Feldman and Laming (2000), Feldman and Widing (2002, 2003, 2007), Asplund et al. (2009), Lodders et al. (2009), Schmelz et al. (2012) and Del Zanna and Mason (2013, 2014). Various articles can also be found in Wimmer-Schweingruber (2001), the proceedings of a joint SOHO/ACE workshop on solar and galactic composition in 2001. There is also a recent *Living Review* by Laming (2015), which also discusses stellar observations and the theoretical aspects of abundance variations in the solar and stellar atmospheres.

We focus here on remote-sensing measurements of abundances in the low corona from XUV spectroscopy. We note that accurate abundance measurements are important in many respects. As described in Laming (2015), the chemical fractionation is intimately related to the processes that heat the solar corona, so FIP bias variations provide important diagnostics.

As briefly mentioned in this chapter, there are clear correlations between remote-sensing and the in-situ abundance measurements. Accurate measurements of remote-sensing abundances will be particularly important for Solar Orbiter, because they can be used to help identifying the source regions of the solar wind that will be measured close to the Sun (at 0.3 AU). There is now a growing consensus that element fractionation is perhaps the only plasma characteristic that does not change, along open field lines from source regions into the heliosphere. Once the FIP fractionation has occurred in the chromosphere, it is not going to change in the low corona, unlike other characteristics such as the temperature, ionization state, particle distributions, etc. In the outer corona, other effects such as gravitational settling can occur and modify the abundances until the plasma becomes collisionless.

The abundances of several elements such as Helium, Neon and Argon cannot be measured in the photosphere, since they do not produce any absorption lines. Therefore, remote-sensing measurements are one direct way to measure the solar abundances of these elements.

Knowing the chemical abundances is also important for modelling the energy budget in coronal structures. In fact, the radiative losses in the chromosphere–corona are a major sink of energy, and depend directly on the absolute abundances.

### Diagnostic methods to measure elemental abundances using spectral lines

Several diagnostic methods have been developed to measure chemical abundances in the low corona. The most common ones rely on some sort of EM/DEM analysis. We recall that for optically thin lines the observed radiance is proportional to the chemical abundance of the element *Ab*(*Z*):

124 $$\begin{aligned} {I(\lambda _{ij})}= {Ab(Z)} {\int \limits _T ~{C(T,\lambda _{ij},N_e)} ~{ DEM} (T) ~ dT}, \end{aligned}$$

so, for example, once a ${ DEM}(T)$ is obtained from, e.g., lines of the same element, the relative abundances of the other elements are directly obtained.

However, what is really needed is the ‘absolute’ abundance of an element, i.e., its abundance relative to hydrogen. One way to establish this is when hydrogen lines are also observed. Hydrogen lines are normally formed in the chromosphere, so radiative transfer effects should be taken into account. However, in off-limb observations the observed hydrogen emission is formed at coronal temperatures, and lines are optically thin, so direct abundance measurements can be obtained (see, e.g., Laming and Feldman 2001). The lines are very weak and can be affected by instrumental stray light and resonance scattering higher up in the corona. Gravitational settling also affects measurements higher up.

Several EM/DEM methods have been developed and used to measure relative elemental abundances. Whenever the plasma is nearly isothermal, the EM loci method (see Sect. 7.2) is often used instead, adjusting the relative abundances so all the EM loci curves cross at one point.

Various approximate methods have also been developed. One method for example is to define an average emission measure $\langle { EM}\rangle$ for each observed line, while another is to define an averaged $\langle { DEM}\rangle$ for each observed line. These approximations and their limitations are described below.

#### The Pottasch approximation

Following Pottasch (1963), many authors have approximated the integral by assuming an averaged value of the *G*(*T*) [i.e., $A_b(Z) ~ C(T)$]:

125 $$\begin{aligned} I_{th} = A_b(Z) ~\langle C(T)\rangle ~ {\int \limits _h ~N_e N_H dh} \end{aligned}$$

A *line emission measure* ${ EM}_L$ can therefore be defined, for each observed line of intensity $I_{\mathrm{ob}}$:

126 $$\begin{aligned} { EM}_L \equiv {I_{\mathrm{ob}} \over A_b(Z) \langle C(T)\rangle } \quad ~ ({\mathrm{cm}}^{-5}) \end{aligned}$$

The approximation applied by Pottasch (1963) was to take $C(T)=0$ when *C*(*T*) is less than one-third of its maximum value, and equal to a constant value otherwise. For $T_1, T_2$ such that $C(T_1)=C(T_2)={1\over 3} C(T_{\max })$, define:

127 $$\begin{aligned} C_{P}(T)= \left\{ \begin{array}{lll} C_{0} &{} \quad T_1 \le T \le T_2 &{} \\ 0 &{} \quad T < T_1, T > T_2 &{} \\ \end{array} \right. \end{aligned}$$

and requiring that

128 $$\begin{aligned} \langle C(T)\rangle = {{\int C(T) dT} \over {|T_2-T_1|}} \end{aligned}$$

which gives $$\langle C(T)\rangle = 0.7 ~C(T_{\max })$$.

**Fig. 105:**
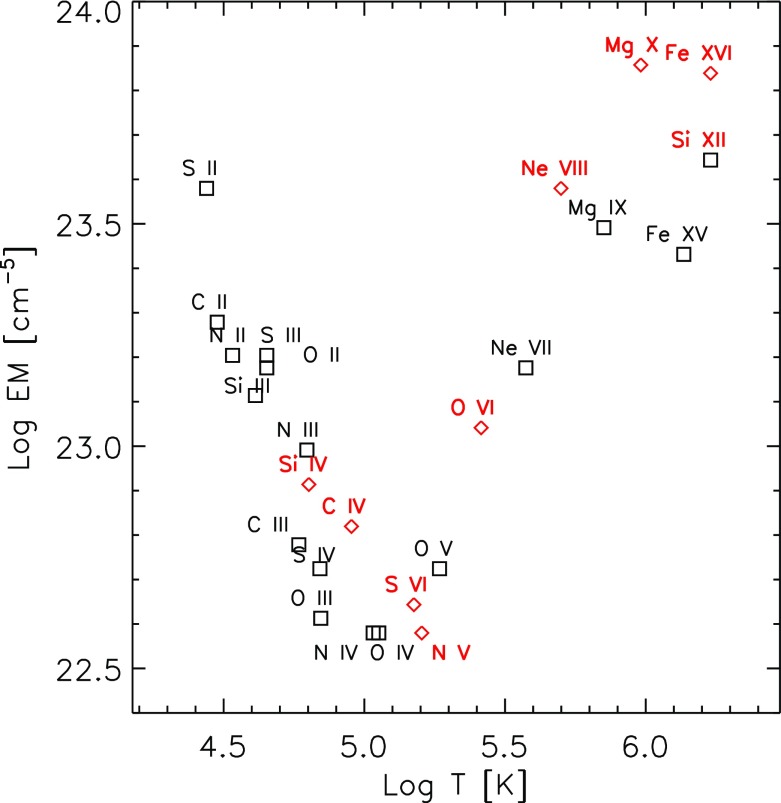
The line emission measures as obtained by Pottasch (1963) with older atomic data. Lines from ions of the Li- and Na-like sequences, such as C iv and Si iv, are shown in red. These are clearly at odds with the other ones

Pottasch (1963) and following authors have produced figures of the *line emission measures*, multiplied by the corresponding abundance value:

129 $$\begin{aligned} A_b(Z) ~{ EM}_L = {I_{\mathrm{ob}} \over 0.7 ~ C(T_{\max })} \end{aligned}$$

plotted at the temperature $T_{\max }$ corresponding to the peak value of the contribution function of the spectral line. An example is given in Fig. 105, obtained from the original data points published by Pottasch (1963). The relative abundances of the elements are derived in order to have all the *line emission measures* of the various ions lie along a common smooth curve.

#### The Jordan and Wilson (1971) approximation

Jordan and Wilson (1971) adopted the Pottasch (1963) approach but proposed a different approximation, assuming that *C*(*T*) has a constant value over a narrow temperature interval $\varDelta { log} T=0.3$ around $C(T_{\max })$:

130 $$\begin{aligned} C_{J}(T)= \left\{ \begin{array}{ll} C_{0} &{} \quad |\log \,T - \log \,T_{\max }| < 0.15 \\ 0 &{} \quad |\log \,T - \log \,T_{\max }| > 0.15\\ \end{array} \right. \end{aligned}$$

and requiring that

131 $$\begin{aligned} \int C(T) dT = \int C_{J}(T) dT \end{aligned}$$

so that

132 $$\begin{aligned} C_{0}={\int C(T) ~dT \over T_{\max }(10^{+0.15} - 10^{-0.15})} = {\int C(T) ~dT \over 0.705 ~T_{\max }} \end{aligned}$$

and thus deducing the relative *line emission measure* ${ EM}_L$, sometimes indicated by EM(0.3), to make it clear that the contributions are calculated over a temperature interval $\varDelta$ log $T=0.3$. This approximation has been used by various authors to derive element abundances. As an example, values of EM(0.1) (at intervals of $\varDelta \log T=0.1$) relative to a Hinode EIS spectrum of an active region core are displayed in Fig. 54 as triangles. The EM loci are upper limits and can be quite different (as Fig. 54 shows) from the actual EM values, which should be obtained once the full DEM has been calculated.

#### The Widing and Feldman (1989) approximation

A different approach was proposed by Widing and Feldman (1989). The idea is to extract from the integral an averaged value of the DEM of the line:

133 $$\begin{aligned} { DEM}_L \equiv \left\langle N_e N_H {{ dh} \over { dT}} \right\rangle \quad (\mathrm{cm}^{-5}\,\mathrm{K}^{-1}) \end{aligned}$$

such that for each line of observed intensity $I_{\mathrm{ob}}$:

134 $$\begin{aligned} { DEM}_L \equiv {I_{\mathrm{ob}} \over A_b(Z) {\int \nolimits _T ~{C(T) { dT}}}} \end{aligned}$$

A plot of the $A_b(Z) ~ { DEM}_L= I_{\mathrm{ob}}/{\int \nolimits _T ~{C(T) { dT}}}$ values displayed at the temperatures $T_{\max }$ gives the ${ DEM}_L$ values of the observed lines.

This method has been widely used in the literature to obtain relative element abundances, adjusting the abundances in order to have a continuous sequence of the $A_b(Z)~{ DEM}_L$ values. In reality, the approximation only works when a smooth continuous distribution of the plasma temperature is present. In fact, given two elements $X_1$ and $X_2$, the ratio of the observed intensities can be written:

135 $$\begin{aligned} {I_1 \over I_2} = {{A_b(X_1) ~\int \nolimits _T~ {C_1(T, N_{_e})} ~{ DEM} (T) ~dT} \over {A_b(X_2) ~\int \nolimits _T~ {C_2(T, N_{_e})} ~{ DEM} (T) ~dT}} \end{aligned}$$

from which the relative element abundance $A_b(X_1)/A_b(X_2)$ can be deduced, from the observed intensity ratio $I_1/I_2$, once the DEM distribution is known. Only when the two lines have similar *C*(*T*) and the DEM distribution is relatively flat would one expect that the DEM factors out from the integrals:

136 $$\begin{aligned} {\int \nolimits _T~ {C_1(T, N_{_e})} ~{ DEM} (T) ~{ dT} \over \int \nolimits _T~ {C_2(T, N_{_e})} ~{ DEM} (T) ~{ dT}} = {\int \nolimits _T~ {C_1(T, N_{_e})} ~{ dT} \over \int \nolimits _T~ {C_2(T, N_{_e})} ~{ dT}} \end{aligned}$$

If the above equality holds, then it is possible to deduce the relative abundances directly from the observed intensities and the contribution functions, because:

137 $$\begin{aligned} {A_b(X_1) \over A_b(X_2)} = {{I_1 \cdot ~\int \nolimits _T~ {C_2(T, N_{_e})} ~{ dT}} \over {I_2 \cdot ~\int \nolimits _T~ {C_1(T, N_{_e})} ~{ dT}}} = {{ DEM}_L(X_2) \over { DEM}_L(X_1)}, \end{aligned}$$

i.e., the *DEM* method and the ${ DEM}_L$ method are equivalent. Of course, if two lines have exactly the same contribution function, then the ${ DEM}_L$ method should produce the same results as the *DEM* method. However, the contribution functions of lines from two ions are never exactly the same, and any small difference can be amplified if the *DEM* is a steep function where the two lines differ. In such a case, the *DEM* and ${ DEM}_L$ methods can be produce very different results.

**Fig. 106:**
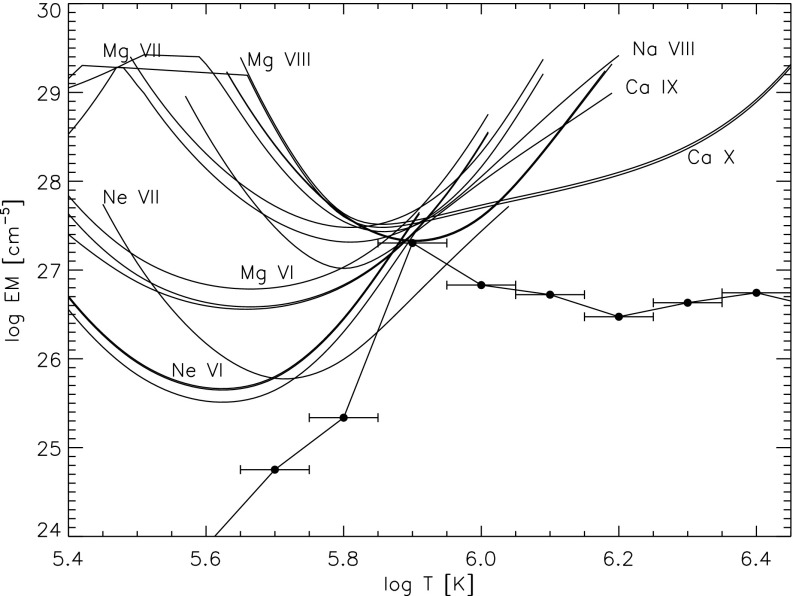
EM loci $I_{\mathrm{ob}}/(G(T))$ curves obtained from the coronal hole plume observed by Skylab (Widing and Feldman 1989), assuming photospheric abundances. The data indicate an isothermal distribution at $\log T\,(\mathrm{K})=5.9$ and are consistent with no FIP effect being present. The emission measure *EM*(0.1) values are also shown (filled circles) Image reproduced with permission from Del Zanna et al. (2003), copyright by ESO

There are several features on the Sun where the plasma distribution appears to be nearly isothermal, and the ${ DEM}_L$ approximation can produce significant errors in the derived abundances. For example, several abundance measurements were obtained from the Skylab slit-less spectrometer on active region loop legs or coronal hole plumes. In these cases, it has been shown by, e.g., Del Zanna et al. (2001b) and Del Zanna (2003) that the method can seriously overestimate the relative chemical abundances, whenever the DEM distributions are close to isothermal. As an example, Fig. 106 shows the EM loci curves obtained from the coronal hole plume observed by Skylab (Widing and Feldman 1989), which are consistent with photospheric abundances, and not an increased Mg/Ne ratio by a factor of 10 as obtained with the Widing and Feldman (1989) approximation.

### Absolute elemental abundances using the continuum

One way to get absolute abundances is to measure both line radiances and the continuum. As already pointed out by Woolley and Allen (1948), the visible lines allow a direct measurement of the absolute (i.e., relative to hydrogen) values of the chemical abundances, by comparing line radiances with the visible continuum, whenever observed. This is because the continuum is due to the Thomson scattering of the photospheric radiation by the coronal electrons. Given that this Living Review is focused on XUV diagnostics, we do not discuss this diagnostic further here.

Another possibility which has been exploited in the X-rays is to use the X-ray line to continuum measurements (see, e.g., Veck and Parkinson 1981). The X-ray continuum emissivity at some temperatures and wavelengths is dominated by free–free radiation, that is mostly by hydrogen, hence the continuum is directly proportional to the hydrogen density. These types of measurements are normally limited to the X-rays when free–free emission becomes significant during solar flares (at temperatures of the order of 10 MK). However, some free–bound emission, which depends on the chemical abundances of several elements, is also present (see Fig. 18) and needs to be taken into account in the analysis. We provide several examples below on the use of this diagnostic.

Finally, we point out that the same measurements can be carried out in the UV using the free–free emission during a flare. Results from SUMER have been obtained by Feldman et al. (2003).

### Overview of abundance measurements

It has become commonly accepted in the literature that the solar corona has a FIP bias of about 4, while coronal holes have near photospheric abundances. Results on active regions and flares have been controversial, and several issues are still very much debated in the literature.

We start by noting that several issues have complicated the interpretation of the measurements. First, most of the remote-sensing and in-situ measurements are about *relative abundances*, while only occasionally have results been obtained relative to hydrogen. Indeed it is still debated whether the low-FIP elements are *enhanced* in the corona, or high-FIP ones are *depleted*, or if a ‘hybrid’ solution (cf. Fludra and Schmelz 1999) applies. Second, the FIP effect is normally measured *relative to a standard set of photospheric abundances*. Third, it has not been possible to measure directly the solar photospheric abundances for some important elements (such as the high-FIP He, Ar, Ne). Fourth, results for the solar photospheric abundances have been changing (and debated) considerably in the literature, especially in the past few years, when 3D time-dependent hydrodynamic simulations of the solar convection zone have been introduced. Measurements of the FIP bias have therefore been changing as a consequence. Fifth, several measurements had little or no spatial resolution, many were full Sun. Finally, several approximate or inaccurate methods have been used.

A major problem of full-Sun measurements is related to the fact that while most of the contributions to the irradiance of lines formed up to 1 MK comes from QS areas, a significant contribution for the higher-temperature lines comes from the hot loops in active regions, so relative abundances in lines formed at different temperatures refer to different solar regions. One example of an often-cited result based on irradiance measurement is the Laming et al. (1995) study. They used the excellent grazing-incidence spectrum published by Malinovsky and Heroux (1973) and an emission measure analysis. They found near photospheric abundances when considering lines formed in the transition region, up to 1 MK. However, they found a FIP bias of 3–4 at temperatures above 1 MK. Malinovsky and Heroux (1973) presented a calibrated spectrum covering the 50–300 Å range with a medium resolution (0.25 Å), taken with a grazing-incidence spectrometer flown on a rocket on 1969 April 4, when the Sun was ‘active’, and significant contributions from active regions were present. Laming et al. (1995) results therefore seem to be consistent with the overall picture that we discuss below, i.e., that the quiet Sun has nearly photospheric abundances, while the hot loops in active regions show a FIP bias of 3–4.

On the other hand, there is a significant body of literature where observations have been interpreted in a different way: that the lower TR and the corona above have different FIP biases (see also Feldman and Laming 1994; Feldman 1998). This is the interpretation that Laming et al. (1995) made, in terms of unresolved fine structures, ufs, small TR loops.

Due to the nature of the Skylab NRL slitless spectrometer, only small-size bright features could be readily observed with this instrument. Many of the abundance results were obtained from nearby lines of Mg vi (low-FIP) and Ne vi (high-FIP).

SOHO CDS could resolve lines from a sequence of magnesium [Mg v, Mg vi, Mg vii, Mg viii] and neon [Ne iv, Ne v, Ne vi, and Ne VII] ions before degradation due to the temporary SOHO loss, so the FIP effect could be studied in terms of Mg/Ne relative abundances at transition region temperatures. SOHO SUMER also offered several abundance diagnostic ratios, but again mostly in transition-region lines.

On the other hand, some diagnostics in coronal lines have been available with Hinode EIS. As described in Del Zanna (2012b), Hinode EIS observes several lines formed around 3 MK, from the low-FIP Fe, Si, Mg, Ni, Ca and the high-FIP Ar. Strong lines from S are also present. They are very useful because S abundances normally show the same FIP effects as those of the high-FIP elements.

Finally, we note that there are many studies of solar abundances that are not strictly measurements of the FIP effect. For example, see Young (2005b) on the O/Ne ratio in the quiet Sun and Schmelz et al. (2005) on the O/Ne ratio in active regions. In what follows, we focus mainly on results relating to the FIP effect.

### Abundances in coronal holes and plumes

Feldman and Widing (1993) obtained near photospheric Mg/Ne abundances (FIP bias about 1–2) from the rims of the limb brightening in coronal holes imaged by the Skylab NRL slitless spectrometer in Mg vi and Ne vi lines.

No significant FIP effect was observed using SOHO CDS observations in coronal holes (Del Zanna et al. 2001b, 2003). Del Zanna and Bromage (1999b) found indications of a small FIP bias between cell centres and supergranular network areas, with the cell centres showing enhanced Mg/Ne.

Feldman et al. (1998b) used SOHO SUMER observations above the north polar coronal hole at a radial distance $R \le 1.03$ $R_\odot$ and found no significant FIP effect.

Doschek et al. (1998b) used SOHO SUMER observations of the off-limb corona to measure the Si/Ne abundance using primarily lines from Si vii, Si viii, Ne vii and Ne viii. They found that the Si/Ne abundance ratio in inter-plume polar coronal hole regions was about a factor of 2 greater than the photospheric value.

Finally, Laming and Feldman (2001, 2003) measured the He abundance using ratios of He/H lines observed with SoHO SUMER off limb. The authors obtained a value similar to the He abundance measured in-situ in the fast solar wind.

The main features within coronal holes are plumes. It is important to assess if plumes have non-photospheric abundances, as in that case this would be a strong indication that plumes are probably not the sources of the fast solar wind, since in-situ measurements indicate nearly photospheric abundances for this type of wind (see the Living Review by Poletto 2015).

The legs of coronal hole plumes are very bright in the transition-region low-FIP Mg vi, Mg vii and high-FIP Ne vi, Ne vii lines, which have been observed with, e.g., Skylab, SoHO CDS and SUMER. Widing and Feldman (1992) analysed one Skylab observation of a plume and found an FIP effect of 10, using the Widing and Feldman (1989) approximation. As discussed above (cf. Fig. 106), the isothermal nature of the plumes led to an overestimation of the FIP effect. Indeed, using the EM loci method and SoHO CDS observations, plumes were found by Del Zanna et al. (2001b), Del Zanna et al. (2003) to have near-photospheric abundances.

Young et al. (1999) found a small FIP bias of only 1.5 at the base of a coronal hole plume, from the Mg and Ne lines observed by CDS. Curdt et al. (2008) pointed out that the ratio of the Ne viii versus Mg viii as observed by SUMER varies across plume structures, but could not perform a temperature analysis (using lines from other ionisation stages) to be able to measure the FIP bias. Recently, Guennou et al. (2015) monitored coronal hole plumes over a few hours using Hinode EIS, and found no significant variation in the plume abundances with time. The values obtained were consistent with photospheric abundances, confirming the CDS results. However, we point out that only a weak an blended line from a mid-FIP element (S viii 198.5 Å) was used to measure the FIP bias relative to Fe and Si.

### Abundances in the quiet Sun

Feldman and Widing (1993) obtained near photospheric Mg/Ne abundances ($\hbox {FIP bias} = 1{-}2$) from the rims of the QS limb brightening in Skylab NRL slitless spectrometer, i.e., basically no difference with the coronal hole case.

Skylab observations of the Mg and Ne lines in small quiet Sun brightenings have generally shown photospheric abundances (cf. Sheeley Jr 1996).

**Fig. 107:**
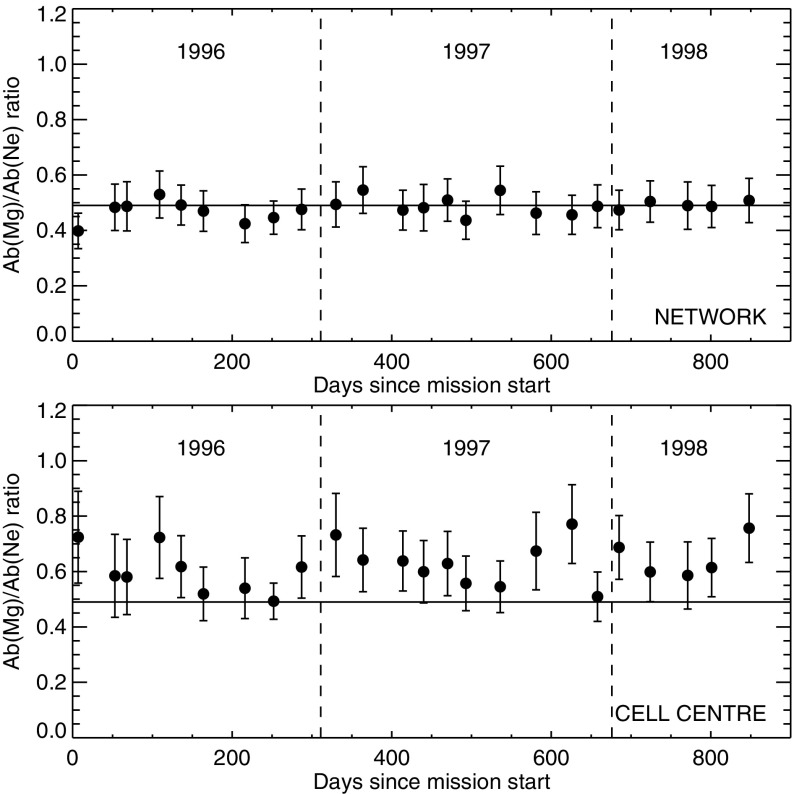
The Mg/Ne abundance ratios for the network (upper panel) and cell centre (lower panel) quiet Sun regions as a function of time, as obtained from SoHO CDS by P. R. Young (a revision of the analysis presented in Young 2005a). The black horizontal line denotes the photospheric Mg/Ne abundance ratio

SOHO CDS results in terms of Mg/Ne abundance in the transition region have generally shown that the quiet Sun has nearly photospheric abundances (Del Zanna and Bromage 1999b). These authors found indications of a small FIP bias between cell centres and supergranular network areas, with the cell-centre regions showing a Mg/Ne enhancement of about a factor of 2.5 compared to the coronal hole network. Similar results were obtained by Young (2005a), where it was further shown that no significant variations with the solar cycle were present (see Fig. 107).

Feldman et al. (1998b) used SOHO SUMER observations in 1996 above a quiet Sun region at a radial distance $R \le 1.03$ $R_\odot$ and found a FIP effect of about 4. A similar FIP bias was found using SUMER by other authors (e.g., Laming et al. 1999), although in several other cases the FIP bias was found to be much smaller, about a factor of 2 (see, e.g., Warren 1999; Widing et al. 2005). The differences were ascribed to local variations or changes due to the solar cycle.

Doschek et al. (1998b) used SOHO SUMER observations of the off-limb corona to measure the Si/Ne abundance using primarily lines from Si vii, Si viii, Ne vii and Ne viii. They found photospheric abundances.

The discrepancy among the SUMER results is puzzling and appears to be related to the choice of spectral lines/ions and potential issues with the atomic data. We note that Feldman et al. (1998b) obtained measurements relative to the Li-like O vi 1032 Å line, and noted that “the Ne viii, Na ix, Mg x lines, all of which belong to the Li-like isoelectronic sequence, appear to indicate a systematic lower effective FIP bias value than the rest of the lines”. As the emission measures of lines from Li-like and Na-like ions are often different than those from other sequences (see Sect. 7.3), it is therefore likely that, depending on the choice of lines, different results could be obtained. The quiet Sun off-limb measurement are ideal to measure abundances because the temperature is nearly isothermal (see Sect. 12.2). It was noted by Landi et al. (2002b) that the SUMER lines of different isoelectronic sequences in such off-limb observations have different emission measures. The authors suggested a FIP bias of 4. On the other hand, a re-analysis of the same observation by Del Zanna and DeLuca (2018) using more recent atomic data (CHIANTI v.8) and a different selection of ions indicates abundances close to the photospheric ones recommended by Asplund et al. (2009). Due to discrepancies found, it was suggested by Landi et al. (2002b) and Widing et al. (2005) that some of the atomic data available at the time were not accurate. This affected, e.g., the complex coronal iron ions, which were significantly improved in CHIANTI v.8, and the forbidden lines in various ions, which still do not have accurate enough atomic data, as discussed in Del Zanna and DeLuca (2018).

Another issue to be considered is the photospheric abundance of oxygen, which was used as a reference by Feldman et al. (1998b), and which has been modified in recent times. Interestingly, Widing et al. (2005) analysed another SUMER observation in 1999, and found a smaller FIP bias of about 2 between Mg, Si and O, from the ratios of Mg ix, Si viii and O vi lines. However, a comparison against the H i Ly $\beta$ indicated nearly photospheric abundances for Mg and Si, with a depletion of O by almost a factor of 2. This differs from the result of Feldman et al. (1998b), where the relative O/H ratio was found to be close to photospheric.

Finally, we note that Laming and Feldman (2001, 2003) found the He abundance to be close to its ‘photospheric’ value (He/H about 5% by number), using ratios of He/H lines observed with SOHO SUMER off the limb.

#### Abundances in the quiet Sun outer corona

Raymond et al. (1997) used SOHO UVCS measurements (off limb) and found that high-FIP elements were depleted by an order of magnitude (compared with photospheric abundances) in the core of a quiescent equatorial streamer, during solar minimum. On the other hand, low-FIP ions were also depleted by about a factor of 3. The abundances along the edges of streamer legs were similar to those measured in the slow solar wind, which is a possible indication that they are the source regions for the slow wind. Several abundance studies based on SOHO UVCS observations have followed, but have found similar results.

It has been noted from the SUMER and UVCS observations by e.g., Feldman et al. (1998b) and Raymond et al. (1997) that gravitational settling is an important effect in the outer corona, which complicates any interpretation of the observations. Another complication in the analysis is the process of photo-excitation, which becomes important in lower density plasma and affects different lines in different ways.

Since the present Living Review does not discuss photo-excitation in detail or the outer corona in general, further analyses of UVCS observations that are available in the literature are not reviewed here.

Finally, it is worth mentioning that CHASE observations in the outer corona of the ratio of He ii at 304 Å to the H Lyman-$\alpha$ at 1218 Å suggested a helium abundance of $0.079\pm 0.011$ for the quiet Sun (Gabriel et al. 1995).

### Abundances in active regions

#### Abundances in hot (3 MK) core loops: the $\pi$ factor

The abundance measurements of the hot core loops in quiescent active regions have recently been reviewed in Del Zanna and Mason (2014). Here we give a summary of the main results. The hot core loops are nearly isothermal around 3 MK so FIP measurements require that lines formed at these temperatures are observed.

There are several results on the FIP bias in terms of the Fe versus the O, Ne abundances from X-ray spectra obtained by, e.g., the P78-1 SOLEX B spectrometer (McKenzie and Feldman 1992) and the SMM/FCS (Saba and Strong 1993; Schmelz et al. 1996). Significant variability in the FIP bias was reported. However, a re-analysis of the same SMM/FCS observations by Del Zanna and Mason (2014), selecting only quiescent active region cores and using the most recent atomic data and the DEM analysis, has shown that in most cases the apparent variability can be explained by slightly different temperature distributions. The FIP effect for almost all the active region cores, independently of their age and size, was remarkably consistent at around a value of $\pi$.

Del Zanna (2013a) analysed several Hinode EIS observations of quiescent active region core loops, finding a remarkable constancy of the FIP effect, around a factor of $\pi$, in excellent agreement with the FIP bias obtained from SMM/FCS X-ray observations.

Table 20 shows a summary of some of the abundance measurements in quiescent active region cores.

**Table 20:** Abundance measurements relative to iron in quiescent active region cores

Fe/El.	(FIP)	E68	D75	E	P77	W94	M94	D13	DM14	Phot. A09	Coronal F92
Fe/Ne	(21.6)	2.5	1.7	–	1.0	1.1	–	–	1.2	$0.37^\dagger$	1.05
Fe/Ar	(15.8)	–	–	13.2	–	–	25	50	–	$12.6^\dagger$	33
Fe/O	(13.6)	0.28	0.26	–	0.16	0.16	–	–	0.2	0.065	0.16
Fe/S	(10.4)	–	–	–	–	–	4.0	6.8	–	2.4	6.8
Fe/Si	(8.1)	–	–	–	–	–	–	1.0	–	1.0	1.0
Fe/Mg	(7.6)	–	–	–	1.0	1.05	–	0.8	–	0.8	0.9
Fe/Ni	(7.6)	6.0	–	–	17.8	–	8.3	29.5	–	19.1	18.2
Fe/Ca	(6.1)	–	–	11.2	–	–	–	13.5	–	14.5	14.8

The abundances are listed with decreasing FIP of the element (eV, the FIP of Fe is 7.9). Previous values from quiescent active region observations: E68: Evans and Pounds (1968); D75: Davis et al. (1975); E: Mason (1975b) for Ca and Young et al. (1997) for Ar; P77: Parkinson (1977); W94: Waljeski et al. (1994); M94: Monsignori Fossi et al. (1994); D13: Del Zanna (2013a),obtained from Hinode EIS in the EUV; DM14: Del Zanna and Mason (2014), obtained from a sample of quiescent active region cores observed by SMM/FCS. For reference, we show the Asplund et al. (2009) [A09] photospheric abundances ($^\dagger$ the Ne and Ar values were obtained with indirect measurements) and the ‘coronal’ abundances of Feldman et al. (1992) [F92], which show an average FIP bias of a factor of 4

Using the Hinode EIS absolute in-flight calibration (Wang et al. 2011; Del Zanna 2013b) and density measurements from Fe xiv, Del Zanna (2013a) measured the resulting path lengths and showed that it is the low-FIP elements that must be *enhanced* at least by a factor of 3, otherwise the path lengths would be unreasonably larger than the size of the observed structures.

If one assumes a filling factor of one, the EUV and X-ray measurements of the AR cores are therefore consistent with the abundances of the high-FIP elements being photospheric, and those of the low-FIP elements increased by about a factor of 3.

This FIP effect is consistent with the Ca/H abundance obtained by Mason (1975b) from the Ca xv intensity above an active region (a coronal condensation) observed during the total solar eclipse in 1952 in Kharthoum.

#### Abundances in plage and sunspots

Feldman et al. (1990) studied HRTS UV observations of low charge states of C and Si and found an increased Si/C abundance in plage areas, compared to a sunspot. The increase was about a factor of 3. A comparison with the H i $\hbox {L}\alpha$ indicated that the sunspot had photospheric abundances. Abundance variations from the HRTS data were also studied by Doschek et al. (1991), where increased Si/C abundances in active regions were confirmed.

#### TR brightenings

Young and Mason (1997) studied the relative Mg/Ne abundance using SOHO CDS observations of active region brightenings, some of which were associated with emerging flux regions. They found variable abundances. The brightenings associated with emerging flux regions in the core of an active region showed near photospheric abundances, while those associated with long-lasting fans of loop showed an increased Mg/Ne abundance.

The fact that abundance variations in transition-region lines seemed to be associated with the emergence of the magnetic field was previously pointed out by Sheeley Jr (1995) using Skylab observations.

#### Abundances in warm (1 MK) loops

The legs of AR ‘warm’ 1 MK loops were clearly observed by the Skylab NRL slitless spectrometer in lines of the low-FIP Mg and high-FIP Ne formed at low temperatures (Mg vi, Mg vii, Mg viii and Ne vi, Ne vii), hence there is ample literature on the FIP effect in terms of Mg/Ne relative abundances. A particularly interesting result is the fact that the FIP seems to increase with the age of an active region (Widing and Feldman 2001), from being non-existent at birth to a typical increase of a factor of 4–5 in 2–3 days. One issue, however, is that the above results were obtained using the Widing and Feldman (1989) approximation, which assumes that a smooth emission measure distribution exists.

However, AR warm loops are normally consistent with the plasma being nearly isothermal (across their cross-section, not along the length), so the FIP bias obtained using the Widing and Feldman (1989) method may be overestimated (Del Zanna 2003). In one case, the EM loci method applied by Del Zanna (2003) to Skylab observations indicated an FIP bias 4 times smaller, but the FIP bias was still about a factor of 4.

**Fig. 108:**
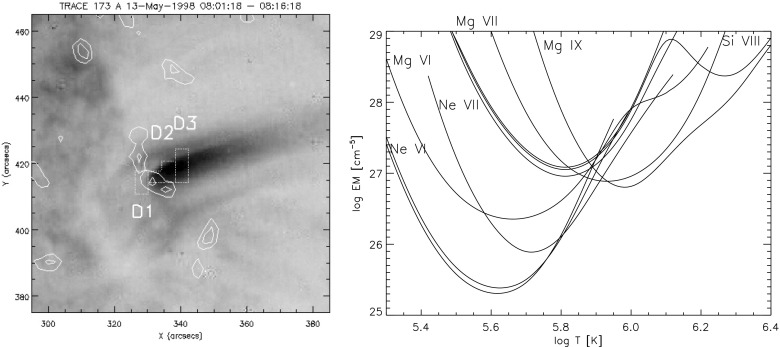
Left: TRACE 171 Å image of the leg of a warm loop, with contours of the photospheric magnetic fields and three areas selected. Right: EM loci curves for region D2, using photospheric abundances Adapted from Del Zanna (2003)

Results based on SOHO CDS spectra indicated that the Mg/Ne abundances in the legs of quiescent 1 MK loops have either an FIP effect of about a factor of 4 (cf. Del Zanna and Mason 2003), or have near photospheric abundances (Del Zanna 2003). Figure 108 shows one of the many warm loops that showed photospheric abundances.

We note that Del Zanna et al. (2011a) proposed that a significant fraction of the warm loops are formed by interchange reconnection of the hot core loops, which show a FIP bias of a factor of 3–4.

#### Abundances in the unresolved 1–2 MK AR emission

A FIP bias of about 2 was found using Hinode EIS off-limb observations of the unresolved diffuse 2 MK emission in active regions (Del Zanna 2012b). Similar results were found in several active regions (Del Zanna 2013a).

Baker et al. (2013) have reported similar relatively small FIP biases (2–3) in the core of a young active region, obtained from lines formed around 1.5 MK, S x and Si x. It is however unclear if this bias is associated with the unresolved 1–2 MK emission or to the warm loops.

#### Abundances in coronal outflows

The so-called coronal outflows in active regions have been discovered with Hinode EIS. The coronal lines observed by Hinode EIS show persistent blue-shifts that are progressively stronger in lines formed at $T > 1$ MK and are located exactly above regions of strong magnetic field (sunspots umbrae and plage) (Del Zanna 2008c). It was also shown that they are typically persistent over several days and have a large spatial expansion. Line profiles are generally symmetric but broadened.

Several other studies (Doschek et al. 2008; Harra et al. 2008; Hara et al. 2008; Baker et al. 2009) have been carried out, giving a consistent picture, although some details have been debated in the literature (e.g., the asymmetry of the line profiles). Del Zanna et al. (2011a), Bradshaw et al. (2011) developed a physical model which is able to explain the general characteristics of these outflows, triggered by interchange reconnection between the magnetically closed hot (3 MK) core loops and the surrounding open magnetic field. The model predicts that part of the outflows would contribute mass and momentum into the slow solar wind, and the reconnection would create some of the warm (1 MK) loops.

Brooks and Warren (2011) found a rather large (3–5) FIP bias (in terms of the S x versus Si x abundance) in the coronal outflows associated with an active region. Such FIP biases are close to those observed in the slow solar wind by in-situ instruments, which is suggestive that indeed coronal outflows are directly contributing to the solar wind (see also Brooks and Warren 2012a; Brooks et al. 2015).

Further measurements of this kind will be very useful in conjunction with the in-situ abundance measurements of Solar Orbiter.

### Abundances in flares

There is quite an extensive literature on abundances in solar flares, although some results are contradictory. It remains to be seen if appropriate methods and updated atomic data will lead to a revision of the previous results, as in the case of quiescent active regions cores.

The FIP bias of 3–5 MK plasma can be measured from the Fe, Mg versus the H-like and He-like O, Ne X-ray lines. One of the first studies was that of Veck and Parkinson (1981), where X-ray spectra from OSO-8 of solar flares were analysed. Both H-like, He-like lines and the continuum were measured.

McKenzie and Feldman (1992) analysed many flares observed with the P78-1 SOLEX B spectrometer and found a variable FIP effect, up to a factor of 4. Doschek et al. (1985) used X-ray spectra from the P78-1 Bragg crystal spectrometers to obtain the following relative abundances: $\hbox {Ar/Ca}=0.65, \hbox {K/Ca}=0.1$, and $\hbox {Ca/Fe}=0.1$. Sterling et al. (1993) analysed 25 flares observed with the NRL SOLFLEX Bragg crystal spectrometer and obtained an average Ca abundance of $5\times 10^{-6}$.

Several results have been published based on the Skylab spectroheliograms. For example, Feldman and Widing (1990) used TR lines from O, Ne, Ar, and Mg during the peak phase of an impulsive flare to find that the relative O/Mg was close to its photospheric value. The Ar/Mg and Ne/Mg abundances were close to galactic values and have often been used to estimate the photospheric abundances of Ar and Ne.

Widing and Feldman (2008) re-analysed a Skylab observation of an arcade of post-flare loops. EUV lines formed around 3–5 MK from Ca, Ni, and Ar indicated a FIP bias between 1.7 and 4.6, i.e., unexpectedly large for a flare. The authors suggested that perhaps some filament material contributed to the arcade.

Many results also exist from SMM. Schmelz (1993) analysed two flares observed by SMM/FCS. Whilst one showed coronal abundances, the second one had anomalous Ne and S abundances. Schmelz and Fludra (1993) found a FIP bias of about 4 from two flares observed with FCS and BCS, with the abundances of high-FIP elements lower than the photospheric values (but anomalous Ne and S abundances were found). Fludra and Schmelz (1995) analysed two solar flares observed with the SMM FCS and BCS instruments, finding generally that the high-FIP elements were depleted, compared to their photospheric values, while low-FIP elements such as Ca were slightly enhanced. Anomalous Ne and Ar abundances were also reported.

**Fig. 109:**
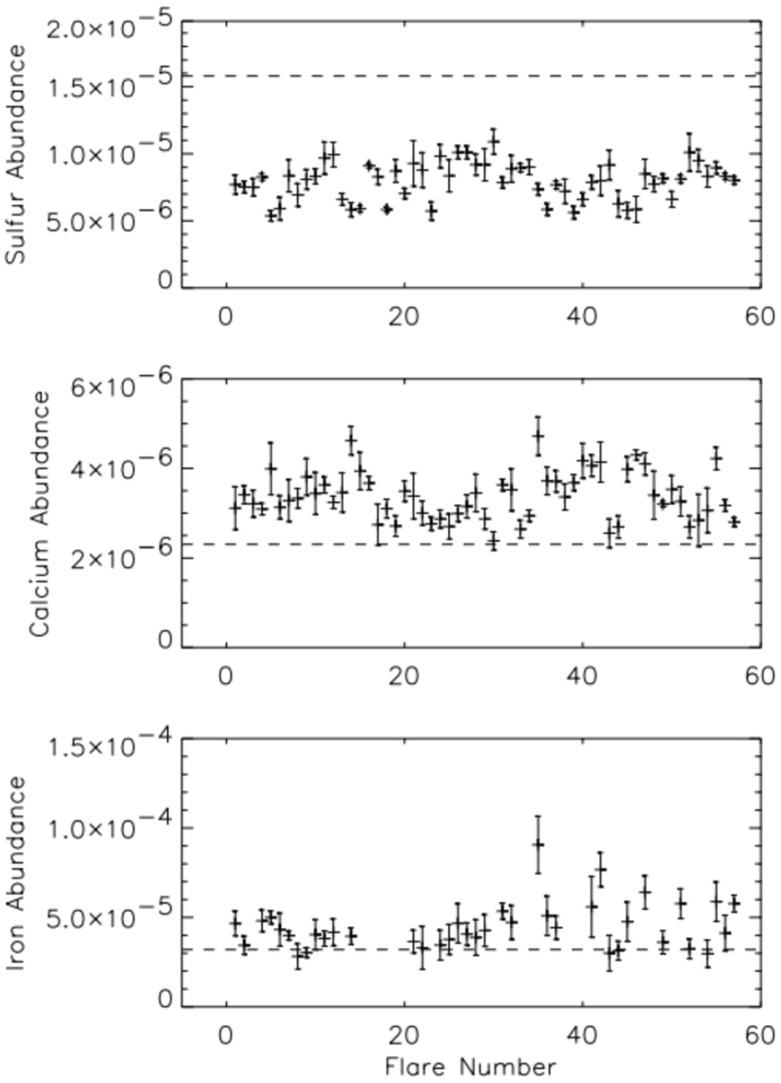
The absolute abundances of Ca, S, and Fe from several flares observed with Yohkoh BCS. The dashed lines were the photospheric values known at the time. Note that the photospheric abundance of S has been significantly revised to $1.3\times 10^{-5}$ by Asplund et al. (2009) Image reproduced with permission from Fludra and Schmelz (1999), copyright by ESO

Line-to-continuum measurements of the He-like Fe, S, and Ca during flares in the X-rays were possible with Yohkoh BCS. Fludra and Schmelz (1999) obtained absolute abundances for many flares, which showed a remarkable constancy, as shown in Fig. 109. There are several similar studies, but often limited to one element. For example, Bentley et al. (1997) measured an average Ca abundance for 177 flares to be $3.6\times 10^{-6}$. Overall, the Yohkoh BCS results indicate a small increase in the Ca abundance, and a S abundance still lower than the revised photospheric abundance of $1.3\times 10^{-5}$ (Asplund et al. 2009). The Fe abundance is pretty much photospheric.

Landi et al. (2007) analysed SOHO SUMER observations of a flare and obtained a Ne abundance of $1.3\times 10^{-4}$ using line versus continuum measurements. This Ne abundance is in agreement with older estimates but significantly higher than the value recommended by Asplund et al. (2009) in their controversial revision of the photospheric abundances.

**Fig. 110:**
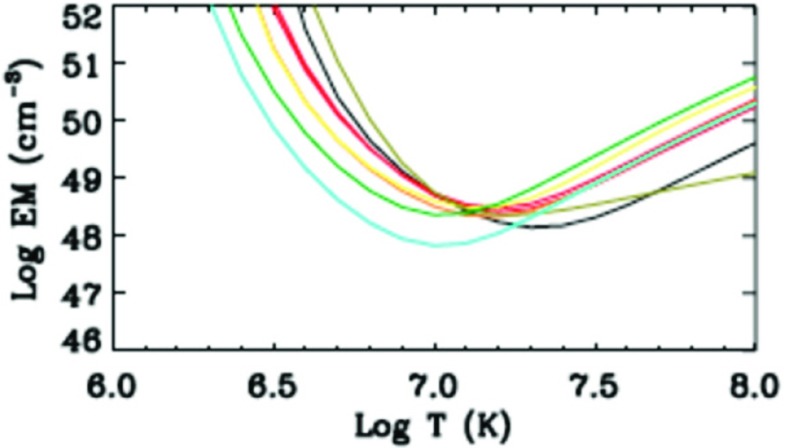
EM loci curves during the peak phase of a flare observed with RESIK Image reproduced with permission from Chifor et al. (2007), copyright by ESO

Feldman et al. (2005) also used SOHO SUMER observations of H, He lines together with the continuum to measure the He abundance, which on average was found to be 12.2%. On the other hand, Andretta et al. (2008) used SOHO CDS and ground-based observations of another (C-class) flare and obtained a He chromospheric abundance of 7.5%. This value is closer to the He abundance obtained from helioseismology, $8.5\% \pm 0.2$ (Asplund et al. 2009) (Fig. 110).

The RESIK crystal spectrometer on-board the CORONAS-F spacecraft has allowed line-to-continuum analyses of solar flares and measurements of the FIP effect in terms of, e.g., Si versus Ar and S abundances. A study by Chifor et al. (2007) found abundances close to the photospheric ones of Asplund et al. (2009) and Ar and S abundances consistent with earlier measurements from SMM. Several similar studies have since been carried out, in a series of papers on K, Cl, Ar, S by J. Sylwester and collaborators, with a variety of results (K enhanced by a large factor, Si by a factor of 2). However, in the latest revised analysis (Sylwester et al. 2014) the results broadly agree with the Chifor et al. (2007) ones, i.e., with abundances close to the Asplund et al. (2009) recommended values, with the exception of K and Ar, as can be seen from Table 21, where a few measurements are listed.

It is interesting to see a relatively good consistency in the results listed in Table 21, obtained from OSO-8, SMM, Yohkoh BCS, and RESIK. The relative abundances of Ar/Ca and Ca/Fe measured by Doschek et al. (1985) with P78-1 are in good agreement, while the K abundance is half-way between the Chifor et al. (2007) and (Sylwester et al. 2014) RESIK results. The NRL SOLFLEX Ca abundance obtained by Sterling et al. (1993) is also in good agreement with the measurements reported in the table.

**Table 21:** Abundance measurements relative to hydrogen during flares from X-ray He-like lines

Element	VP1981	FS1995	FS1999	C$+$2007	S$+$2014	Photospheric
O	–	$2.5 \times 10^{-4}$	–	–	–	$4.9 \times 10^{-4}$
Ne	–	$3.8{-}8.6 \times 10^{-5}$	–	–	–	($8.5 \times 10^{-5}$)
K	–	–	–	$1.2 \times 10^{-7}$	$7.2 \times 10^{-7}$	$1.1 \times 10^{-7}$
Ar	$2.4 \times 10^{-6}$	–	–	$1.5 \times 10^{-6}$	$3.8 \times 10^{-6}$	($2.5 \times 10^{-6}$)
S	$8.1 \times 10^{-6}$	$9.7{-}14 \times 10^{-6}$	7.8–$8.2 \times 10^{-6}$	$7.9 \times 10^{-6}$	$8.7 \times 10^{-6}$	$1.3 \times 10^{-5}$
Mg	–	$4.0{-}4.9 \times 10^{-5}$	–	–	–	$4.0 \times 10^{-5}$
Si	$4.5 \times 10^{-5}$	$3.3{-}4.2 \times 10^{-5}$	–	$3.2 \times 10^{-5}$	$3.6 \times 10^{-5}$	$3.2 \times 10^{-5}$
Ca	$3.2 \times 10^{-6}$	$4.2{-}4.4 \times 10^{-6}$	$2.9{-}3.1 \times 10^{-6}$	–	–	$2.2 \times 10^{-6}$
Fe	–	$3.8{-}4.8 \times 10^{-5}$	$3.9{-}4.1 \times 10^{-5}$	–	–	$3.2 \times 10^{-5}$

VP1981: OSO-8 measurements by Veck and Parkinson (1981); FS1995: SMM results by Fludra and Schmelz (1995); FS1999: results from Yohkoh BCS by Fludra and Schmelz (1999); C+2007: results from RESIK by Chifor et al. (2007); S+2014: results from RESIK by Sylwester et al. (2014). Photospheric abundances are the Asplund et al. (2009) recommended values (those in brackets are derived, not measured)

Direct measurements of inner-shell Fe lines formed during large solar flares observed by the crystal spectrometers aboard Yohkoh, SMM and P78-1 were thought to be consistent with the coronal Fe abundance being equal to the photospheric value, within a factor of two, according to Phillips et al. (1995). Phillips (2012) revised observations of the Fe K$\alpha$ lines observed by P78-1 to obtain an iron abundance about 1.6 times photospheric.

RHESSI observations of flares offer in principle the opportunity to obtain the iron abundance from the line-to-continuum measurements in the X-rays. The 6.65 keV Fe lines are observed as a small ‘bump’ in the RHESSI spectra. Phillips and Dennis (2012) revised a previous analysis to obtain an iron abundance from 20 flares of $8.1\times 10^{-5}$, or 2.6 times the photospheric abundance.

As shown by Del Zanna and Woods (2013), it is possible to use SDO/EVE spectra to obtain information on the FIP effect during large flares. The relative abundance of high-temperature high-FIP argon lines versus low-FIP elements (iron, calcium) was measured. Several flares were studied and in all cases near photospheric abundances were found.

Warren (2014) analysed the SDO/EVE line-to-continuum emission of 21 flares to obtain nearly photospheric iron abundances in all cases.

Finally, measurements of an inverse FIP effect have recently been reported in solar observations. They have been obtained with Hinode EIS, using lines from Ar and Ca, during a solar flare but close to a sunspot (Doschek et al. 2015; Doschek and Warren 2016). These measurements are very interesting because it is the first time that an inverse FIP effect has been reported on the Sun. On the other hand, inverse FIP effects are common in active stars (see, e.g., the review by Laming 2015).

### Conclusions on the abundance measurements

In conclusion, there is little evidence that at transition region temperatures the coronal holes and the quiet Sun present any significant FIP bias.

Some of the SUMER results on the measurements of the quiet Sun above 1 MK appear to be affected by the choice of spectral lines and the atomic data, which still need further improvement.

The 3 MK hot loops in the cores of the active regions generally show a FIP bias of about a factor of three, consistently using EUV and X-Ray measurements.

The situation with regard to the warm 1 MK loops is not clear, in the sense that the FIP bias is not as large as previously thought on the basis of the Skylab observations and the approximate methods previously used.

We end by noting that results from in-situ measurements of the solar wind have similar contradictions and large uncertainties as the remote-sensing ones. However, the overall picture that is emerging is that the fast solar wind has nearly photospheric abundances, while the slow solar wind has abundances that vary with the solar cycle. They are intrinsically very variable, but still normally between the photospheric and the abundances of the hot core loops (FIP bias of 3).

## Conclusions and future

There has been tremendous progress in the plasma diagnostics for XUV spectroscopy since the early rocket observations, in particular in the determination of the physical state of the emitting plasma. Great strides have been made with high cadence imaging instruments, such as SDO/AIA. A better knowledge of the contribution of spectral lines to the Hinode/XRT and SDO/AIA channels, for example using Hinode/EIS spectra has been very fruitful. A very powerful tool is provided by combining imaging and spectroscopic observations, in particular with the combination of SDO, Stereo, Hinode and IRIS instruments.

XUV spectrometers have produced a large amount of data in the past couple of decades, however improvements in terms of spectral resolution, spatial resolution and radiometric calibration have been modest, when compared to earlier observations, for example Skylab and HRTS. More recent spectrometers, such as IRIS, have provided much improved spatial and spectral resolution, with high cadence, but only over a limited wavelength range and FOV.

One of the most significant advances has been with regard to the atomic data calculations, e.g., from the APAP team, reaching an accuracy of 10–20% for the strongest lines, especially when lines from the same ion are considered. However, a significant amount of work is still needed before accurate atomic data are available for all astrophysically-important ions. Together with these new atomic data, another major advance has been the identification of all the strongest lines in the XUV. However, a significant fraction of the weaker EUV and UV lines is remain unidentified. These new atomic data have been consolidated in the recent release of CHIANTI v8.

Some important spectral regions for solar diagnostics have not received enough attention in the past few decades. In particular, the X-ray wavelength range. In this regard, we look forward to seeing data for the first X-ray spectra (after SMM) in the 6–20 Å region with the forthcoming MAGIXS sounding rocket.

Our review of the status of atomic data and observations has shown that several outstanding issues still remain.

High-cadence observations of the transition region with IRIS and of the corona with, e.g., SDO/AIA have clearly shown variability and dynamics on short timescales, likely leading to the consideration of non-equilibrium atomic processes, for example, time-dependent ionisation/recombination. More studies in this regard will be needed for future analyses.

However, emission measure analyses of ‘quiescent’ features normally suggest that equilibrium ionization holds, at least for the majority of ions. One outstanding issue relates to the frequently enhanced intensities of lines from the Li-like and Na-like ions, which produce the strongest UV solar lines. The EUV Helium lines are also often enhanced. These enhancements have not to-date been adequately explained.

Other non-equilibrium effects such as departures from Maxwellian electron distributions might be common in the corona, but are still hard to diagnose with the current instrumentation. Much work in this regard has been carried out to model them, but further refinements are needed.

Regarding electron densities, there is now better agreement in the various measurements of different solar regions, although simultaneous measurements along single structures such as coronal loops are still beyond the current instrumental capabilities. Electron densities in the transition-region flare plasma can reach high values, but measurements based on high-temperature lines are still uncertain. Further measurements of diagnostic line ratios from flare ions are needed.

Regarding electron temperatures, relatively few direct measurements based on ratios of lines from the same ion exist. Measurements of the electron temperatures in the outer corona are particularly important for solar wind modelling. We therefore look forward to off-limb Mg ix observations from the SPICE spectrometer on-board Solar Orbiter.

Regarding elemental abundances and the FIP effect, we have presented an overview of the many results available in the literature, highlighting that in several cases significantly different FIP biases have been obtained. We have pointed out that such differences can partly be explained by the use of different diagnostic techniques, a different selection of the spectral lines and the limitations of the atomic data which was used at the time of the analyses. Most of the discordant results concern the quiet Sun, while a consensus on a FIP bias of about 3–4 in the hot (3 MK) active region cores is emerging. Coronal holes and coronal hole plumes appear to have close to photospheric abundances. However, puzzling results have also been obtained on the ‘warm’ 1 MK loops, and further analyses would be useful. The connection between elemental abundances as measured remotely with those measured in-situ is still extremely hard to study, but will feature prominently in the future with Solar Orbiter observations closer to the Sun. Further advances will be obtained by linking more realistic models with time-dependent plasma processes.

## Footnotes

1 The indexing of a rate or cross-section is indicated either with a *ji* or *j*, *i* in this manuscript.

2 open.adas.ac.uk.

3 http://physics.nist.gov.

4 http://kappa.asu.cas.cz.

5 http://hea-www.harvard.edu/PINTofALE/.

6 http://www.astro.gla.ac.uk/~iain/demreg/.
